# Profiling health professionals’ personality traits, behaviour styles and emotional intelligence: a systematic review

**DOI:** 10.1186/s12909-023-04003-y

**Published:** 2023-02-18

**Authors:** C. Louwen, D. Reidlinger, N. Milne

**Affiliations:** Faculty of Health Sciences and Medicine, Bond Institute of Health and Sport, Bond University, Robina, Gold Coast, QLD 4226 Australia

**Keywords:** Personality traits, Behaviour styles, Emotional intelligence, Health professionals

## Abstract

**Background:**

Non-cognitive traits have been theorised to predict characteristics, career choice and outcomes of health professionals and could represent a homogenous group. This study aims to profile and compare personality traits, behaviour styles and emotional intelligence of health practitioners across a variety of professions.

**Methods:**

Empirical literature was systematically reviewed. A two-concept search strategy was applied to four databases (CINAHL, PubMed, Embase, ProQuest). Title/abstract and full text articles were screened against inclusion and exclusion criteria. Methodological quality was assessed using Mixed Methods Appraisal Tool. Data was synthesised narratively and meta-aggregated where feasible.

**Results:**

Three hundred twenty-one studies representing 153 assessment tools of personality (*n* = 83 studies), behaviour (*n* = 8), and emotional intelligence (*n* = 62) were included. Most studies (*n* = 171) explored personality (medicine, nursing, nursing assistants, dentistry, allied health, paramedics), revealing variation in traits across professions. Behaviour styles were least measured with only ten studies exploring these across four health professions (nursing, medicine, occupational therapy, psychology). Emotional intelligence (*n* = 146 studies) varied amongst professions (medicine, nursing, dentistry, occupational therapy, physiotherapy, radiology) with all exhibiting average to above-average scores.

**Conclusion:**

Personality traits, behaviour styles and emotional intelligence are all key characteristics of health professionals reported in the literature. There is both heterogeneity and homogeneity within and between professional groups. The characterisation and understanding of these non-cognitive traits will aid health professionals to understand their own non-cognitive features and how these might be useful in predicting performance with potential to adapt these to enhance success within their chosen profession.

**Supplementary Information:**

The online version contains supplementary material available at 10.1186/s12909-023-04003-y.

## Background

Information relating to personality traits, behaviour styles and emotional intelligence of qualified health professionals has been gaining interest in the empirical literature. These non-cognitive traits have been explored to determine if they predict characteristics and outcomes of health professionals and their practice [1]. It has been theorised that practitioners from each health profession, based on their choice of career and occupational requirements, could represent homogenous groups in terms of non-cognitive traits [2, 3].

Personality has been investigated within several health professional fields and is believed to be stable over time [4–7], influential in professional practice [3] and to precede professional/speciality choice [4, 7]. Personality is expressed as enduring patterns of feelings, thoughts and behaviours by an individual in different environments [8], and understandings about personality have been helpful in guiding clinicians’ vocational choices where some have matched occupational requirements, routines and rewards to personality traits [3]. Personality traits are known to influence an individual’s perspective, attitudes and behaviours which in turn influence how an individual approaches a situation or conflict [9]. Exploring the similarities and differences of personality across health professions may aid in understanding profession-specific strengths and weaknesses, foster mutual understanding, inform professional practice support strategies, and improve understandings to enhance interprofessional practice [1, 10].

Whilst research into personality traits of qualified health professionals is continuing to emerge, empirical studies that explore behaviour styles which are underpinned by personality traits [8] are still limited. Behaviour is known to be developed based on temperament and informs the ways in which we describe ourselves and others [1, 8]. Behaviour is also dependent on the influence of external factors and internal processing of information [1], which leads to a coordinated response (actions or inactions) of an individual to the external and/or internal stimulus [11]. Therefore, despite humans having preferred behaviours underpinned by their beliefs, values and physiological systems, it is thought that through cognitive reasoning one may be able to influence and change the expression of behaviour over time and contexts [12, 13].

In addition to personality and behaviour, emotional intelligence (EI) represents an assortment of non-cognitive skills and capabilities including empathy, professionalism and integrity, and each of these attributes influence an individual’s ability to cope with environmental demands [14]. Higher levels of EI have been associated with increased professional success and better workplace performance [15, 16]. Those with higher EI show increased individual cognitive-based performance [15], higher interpersonal skills with less conflict [17], increased facilitation of intellectual development [16]; and improved quality of work and productivity [18]. EI has been defined as an individual’s ability to monitor their own and others’ feelings, discriminating them and utilising this information to guide thinking and actions [19]. The application of EI therefore requires self-awareness in order to improve EI through practice and feedback [20]. Increased insight into one’s EI has been shown to be integral to enhancing one’s ability to work effectively with colleagues and clients and can result in enhanced patient-centred care, due to increased ability to manage and read emotions [18, 21]. Brewer [22] identified that enhancement of EI is directly associated with an individual’s capacity to develop skills and competency across five domains of self-regulation, self-awareness, empathy, motivation and social skills [22].

Standardised tools for personality, behaviour and emotional intelligence are utilised to allow individuals to have a better understanding of their own and others’ non-cognitive traits and underlying reasons for their behaviour. There is a wide variety of tools available, with the Myers-Briggs Type Indicator (MBTI) [23] being one validated and reliable tool commonly used to study an individual’s personality traits. Behaviour styles is the least measured non-cognitive trait in the empirical literature, with the DiSC behaviour profiling assessment tool [24], being utilised to understand health professional team interactions and performance [25]. There is a vast array of EI measures, with the Mayer-Salovey-Caruso Emotional Intelligence Test (MSCEIT) and Schutte Self Report Emotional Intelligence Test most utilised [26, 27]. These tools have been applied in health and education settings to identify and describe an individual’s motivators for success; preferred behaviours; influences on career pathway development and job satisfaction [28]. Additionally, they may optimise the success of both individuals and professional teams [18, 29].

Frequently within the literature non-cognitive traits are also explored relative to burnout, acknowledging that health professionals often experience higher levels of burnout due to the emotionally challenging and physically demanding nature of healthcare [30, 31]. Burnout is a measure of physical and psychological fatigue from occupational and professional demands and is characterised by high levels of emotional exhaustion and depersonalisation, and low levels of personal accomplishment [32]. Although the causes of burnout are complex and unclear, burnout and stress are symptomatically similar [33]. Therefore, given non-cognitive traits inform how individuals engage with and cope in different environments, it is understandable that non-cognitive traits are an influencing factor on burnout. Personality has been central in determining burnout, with personality influencing behaviour and performance [30], whilst individuals with higher emotional intelligence and ability to regulate their emotions are shown to have more problem-focused coping styles and hence are less vulnerable to burnout [34].

Although literature on this topic exists, to date there has been no systematic review that synthesises the evidence to profile personality traits, behaviour styles and / or EI capabilities of practitioners across the health professions. This information could be used by educators of health professional students to better understand the personality traits, behaviour styles and EI capabilities of those who have successfully qualified as a health professional. It could also be valuable information for developing strategies to improve performance of student health practitioners beyond their technical skills. Further information about health practitioners’ personality traits, behaviour styles and EI capabilities could also be useful for the higher education sector when establishing the inherent requirements of their entry level health profession programs, and for future students to make decisions about entry into these programs and professional pathways. The purpose of this systematic review was to profile the personality traits, behaviour styles and EI capabilities of qualified health practitioners.

## Methods

The systematic review protocol was developed utilising the Preferred Reporting Items for Systematic and Meta-Analysis Protocols (PRISMA-P) [35], and registered on the International Prospective Register of Systematic Reviews (PROSPERO) (Registration number CRD42020155113).

### Search strategy and key themes

The search strategy was developed by initially running primary searches in key databases using keywords capturing the research question. The identification of relevant articles guided the refinement and formation of the final search strategy and key concepts. There were two key concepts derived within the search strategy, which included: (i) non-cognitive traits and (ii) health professionals. Interventions and outcomes included tools utilised to profile personality traits, behaviour styles and emotional intelligence. The health professionals’ key concept was inclusive of allied health (e.g., physiotherapy, occupational therapy, and speech pathology), nursing, medicine, and dentistry. A full list of search terms for concepts one and two can be found in Additional file 1.

The search terms were further refined using filters dependant on the database searched. The strategy was used to search CINAHL, PubMed, EMBASE (via OVID) and ProQuest Central databases (which provides access to 47 databases across all major subject heading areas including health and medical, social, science, business, arts, humanities, religion, education and technology [36]). The Polyglot Search Translator [37] was utilised to input initial search string (PubMed) of key concepts to adapt the search strategy to the remaining three database search requirements. The search terms and filters used in the systematic search by database is outlined in Additional file 2. Filters utilised for each database were selected based on the available filters for each database. The authors attempted to maintain consistency across all filters selected, though variation is evident due to the construct of each database. To ensure retrieval of studies of relevance to modern health professionals, databases were searched from 1980 onwards. An age limit filter was not used in the search strategy and instead studies of individuals under 18 years of age were excluded when applying the eligibility criteria (Table 1) during the screening process.

**Table 1:** Inclusion and exclusion criteria

	Inclusion Criteria	Exclusion Criteria
**Publication Criteria**	Published in English language Studies with full text	Not published in English language Studies without full text
**Publication Dates**	January 1980 – September 2022	Not published between January 1980 – September 2022
**Research**	All empirical study designs	Single case study designs
**Intervention and Outcome**	Quantitative and qualitative studies reporting baseline characteristics of personality, behaviour and/or emotional intelligence of health professionals using valid tools	Studies not explicitly reporting on behaviour style, personality trait and/or emotional intelligence Studies not reporting a validated measure of behaviour, personality and/or emotional intelligence Studies without baseline data of these characteristics
**Population**	Studies profiling qualified health professionals in allied health (physiotherapist, occupational therapist, speech pathologist, dietitian, psychologist, podiatrist, osteopath, chiropractor), medicine, nursing and dentistry	Studies that profile other health science professions which are not typically registered health professionals, or students of registered health profession Studies of individuals under the age of 18 years

### Selection and screening process

Search results were exported into Covidence (Covidence online systematic review platform, Veritas Health Innovation Ltd., Melbourne, Australia, www.covidence.org) which was used to store all references, identify duplicates, complete title and abstract screening; and determine the number of records for data synthesis.

**Fig. 1:**
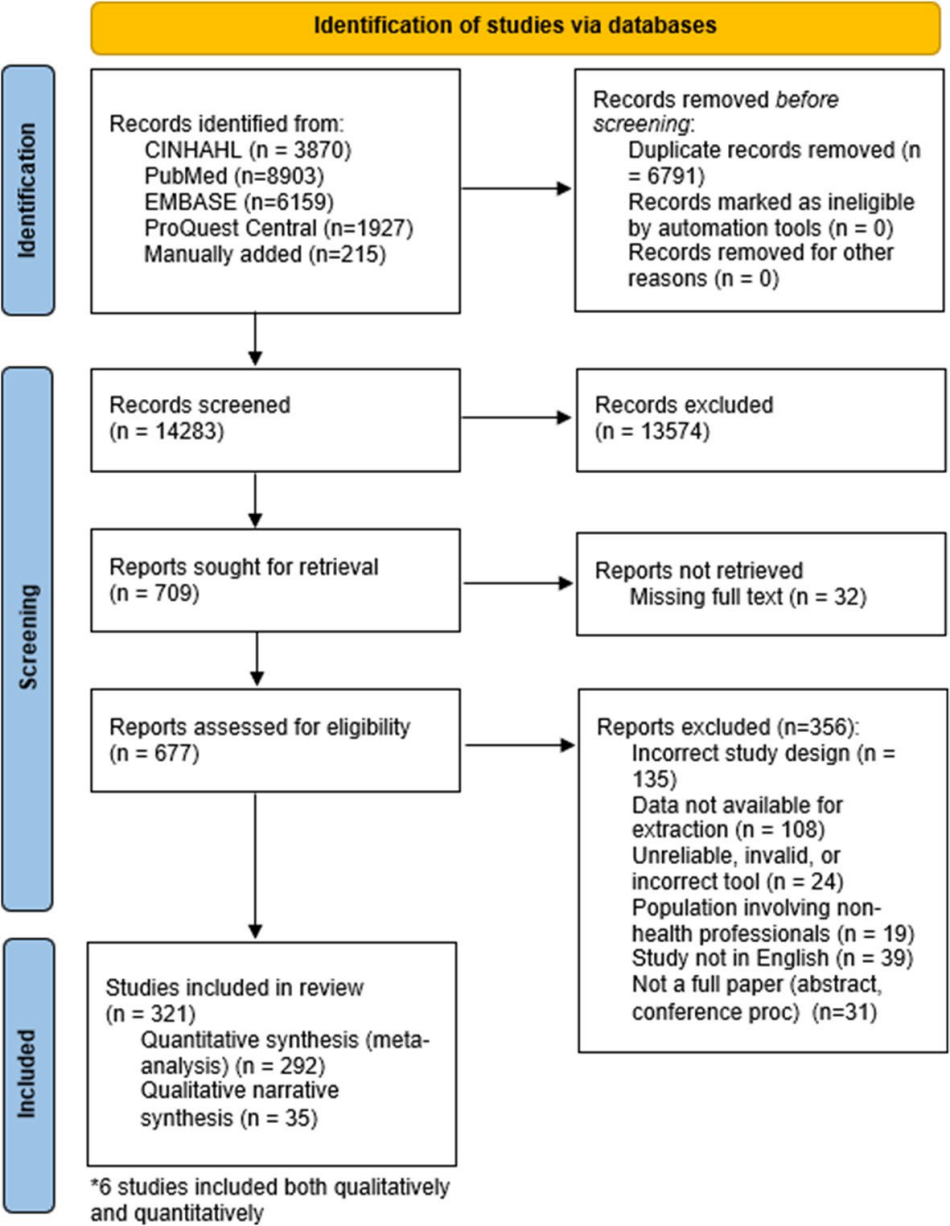
PRISMA flow diagram describing process of study selection [38]

Utilising the inclusion criteria (Table 1), two reviewer pairs (GM/KS, CL/NM) independently screened titles and abstracts for possible inclusions, with a third reviewer managing conflicts (DR). For records that appeared to meet the inclusion criteria, or those that were not clear, full-text records were obtained. Three reviewers (CL, DR, NM) independently screened full text against the eligibility criteria. Any discrepancies of inclusion were resolved by discussion or reference to third reviewer (NM or DR) to reach consensus. Reasons for exclusions were documented (Fig. 1).

### Critical appraisal

All included papers were critically appraised using the Mixed Methods Appraisal Tool (MMAT) [39]. The MMAT comprises five categories dependent on study design and appraises three methodological domains: qualitative, quantitative, and mixed methods. The tool utilises a dichotomous scoring scale, whereby items scored as ‘no’ were awarded zero points and items scored as ‘yes’ were awarded one point. Each study was appraised according to the relevant methodological domain. Scores were converted to a percentage based on the number of ‘yes’ responses compared to the total number of questions. Studies were considered to have high methodological quality when the total percentage score was equal to or above 75% [40], whilst studies below 75% were deemed to have a lower methodological quality [41].

Two independent reviewers completed the critical appraisal process (CL, NM) for each search stage (initial search (CL, NM) and updated search inclusive of MeSH terms (CT, DR). The level of agreement between the critical appraisers was examined by a Kappa analysis using SPSS, version 24 [42]. Consensus between the two appraisers was determined by a third reviewer (DR, CH) when discrepancies in scores were evident and could not be resolved through a process of discussion.

### Data extraction and synthesis

A standardised template agreed prior to data extraction was used to guide extraction of study characteristics and outcome data by a single reviewer (CL), with a second reviewer (NM) validating the data extracted from the includes studies. Data extraction included: (a) methods (study authors, title, aims/objective, location, study design); (b) participants (N = number, profession, age (mean or median; range), gender); (C) non-cognitive traits (health professional behaviour pattern, personality and emotional intelligence assessment measures); and (d) outcomes. Data was recorded in an Excel spreadsheet [43].

The extracted data was synthesised and meta-aggregated for quantitative analysis, and narratively synthesised reporting on emerging concepts and key findings for qualitative analysis. Meta-aggregation was conducted for each of the outcomes assessed utilising the Exploratory Software for Confidence Intervals (ESCI) Meta-Analysis software [44] for mean and standard deviation (SD). Where the SD data was not available the SD was calculated utilising the Cochrane calculator [45] from either *p*-values or 95% confidence intervals (CI). If the *p*-value was not available, the highest SD available from other included studies using the same measure was imputed using methods consistent with previous studies [46], and this was required for 31 studies. Where studies only included mean and inter quartile range (IQR), the mean and SD was calculated utilising sample size, median and IQR using methods consistent with previous published studies [47, 48], and this was required for two studies. Where published results did not provide global totals or subscale totals, mean and SD calculations were reported according to the intended tool’s purpose.

## Results

### Literature search and selection

The results of the literature search, screening and selection process are outlined according to the Preferred Reporting Items for Systematic Reviews and Meta-Analyses (PRISMA) format in Fig. 1.

### Participants

Of the 321 studies included 112,691 participants from 53 different countries were reported, ranging from 10 to 5148 participants in each study. Most participants were nurses (*n* = 64,250), followed by doctors (*n* = 36,029), allied health (*n* = 5068), dentists (*n* = 4139), unidentified health professionals (*n* = 2247), paramedics (*n* = 744), nursing assistants (*n* = 177 and pathologists (*n* = 37). The 5068 allied health participants included occupational therapists (*n* = 1944), dietitians (*n* = 776), physiotherapists (*n* = 684), pharmacists (*n* = 298), psychologists (*n* = 118), radiologists (*n* = 117), social workers (*n* = 8) and non-defined health professionals (*n* = 1123).

### Methodological quality of included studies

The level of agreement between the two initial critical appraisers using the MMAT was 74.05% (Cohen’s Kappa (K) = 0.841, *p* = < 0.01), and in the updated search 88.89% (Cohen’s Kappa (K) 0.602, *p* = < 0.05) indicating a high level of agreement. Following a consensus process, 100% agreement was achieved for all studies critically appraised. Two hundred and seventeen studies achieved YES responses for more than 75% of the questions, indicating that they were of high methodological quality and 104 of the included studies did not meet this threshold and were therefore not considered high methodological quality studies as interpreted according to Radomski, Wozney [40] and Horswood, Baker [41]. Papers of high quality consistently demonstrated complete outcome data utilising an appropriate outcome measure that was administered as intended. Lower quality papers were generally not able to demonstrate if the sample was representative of the target population, with uncertainty regarding confounder accountability or risk of nonresponse bias.

### Tools used to assess personality traits, behaviour styles and emotional intelligence across the health professionals

The 321 included studies used 148 different outcome measure tools (with some tools having multiple versions, or reporting data in different formats), across the measures of personality traits (*n* = 84 tools), behaviour styles (*n* = 8), and emotional intelligence (*n* = 56). Eighty-four of these tools measured personality traits inclusive of 281 different personality trait subscales; eight measured behaviour styles across seven categories; 56 measured emotional intelligence including 102 subscale items (Additional file 3).

### Profiling the personality traits, behavioural styles and emotional intelligence of health professionals

Relevant results from each included study were tabulated and are presented in the data extraction table (Additional file 4). The meta-aggregation included 292 studies, and qualitative synthesis included 35 studies, noting 6 studies were both qualitatively and qualitatively analysed (Fig. 1). Results from each of the studies have been synthesised according to the measured factor, subscale/category and the health professional population in the following sections.

### Personality traits

Personality was the most measured factor with 171 studies (*n* = 65,581). Ninety-eight of these studies were in nursing [1, 9, 49–142], 52 in medicine [1, 30, 58, 90, 94, 100, 143–185], 14 in allied health [10, 28, 89, 178, 186–196], seven in dentistry [197–203], three were unidentified health professional groups [94, 204, 205], three in paramedics [206–208], one in nursing assistants [209], and one in pathologists [210]. Data from 143 of these studies were meta-aggregated (Table 2) and 20 were narratively synthesised by profession and personality trait (presented below).

**Table 2:** Meta-aggregated results for personality traits of health professionals (*n* = 143 studies)

Profession	Personality Traits	Tool	N	Mean	SD	Practical Interpretation
**Medicine**	*Ability to cope and tolerance of negative emotion* [175]	Resiliency Assessment Scale	655	14.58	3.28	*Information not available for interpretation*
	*Abstractedness* [154]	Cattell’s Sixteen Personality Factor Form C	231	5.47	0.00	Doctor exhibit slightly lower abstractedness than population norm.
	*Agreeableness* [100, 150, 154–157, 159, 162, 165, 166, 168–174, 177, 180, 184]	Big Five Inventory	1475	3.80	0.08	Doctors score 3.80 out of 5, where higher scores = higher association with personality trait.
		Big Five Inventory (BFI) *Subscale Sum Score*	563	36.28	0.00	*Information not available for interpretation*
		Five Factor Model of personality (FFM)	4068	3.67	0.33	Doctors score 3.67 out of 5, where higher score = higher association to personality trait.
		Mini-markers of the Big Five Factor Structure personality scale	70	7.30		*Information not available for interpretation*
		NEO Five-Factor Inventory *Sten Score*	50	8.30	1.50	Doctors exhibit average levels of agreeableness.
		NEO Five-Factor Inventory *5 point scale*	872	34.43	2.72	Doctors score 34.43 out of 60, where higher score = higher association to personality trait.
		NEO Personality Inventory Revised *5 point scale*	32	3.87	0.50	Doctors score 3.87 out of 5, where higher score = higher association to personality trait.
		NEO Personality Inventory Revised	984	128.06	25.66	Doctors exhibit above level when compared to population norm.
		Ten-Item Personality Inventory (TIPI)	64	8.74	1.82	*Information not available for interpretation*
	*Apprehension* [154]	Cattell’s Sixteen Personality Factor Form C	231	6.06	1.55	Doctors exhibit higher agreeableness than population norm.
	*BIS* [182]	Behavior Inhibitory System/Behavior Approach System (BIS/BAS)	801	19.90	3.20	Doctors score 19.9 out of 28
	*BAS Drive* [182]	Behavior Inhibitory System/Behavior Approach System (BIS/BAS)	801	12.20	2.10	Doctors score 12.20 out of 16, indicating a higher degree of drive.
	*BAS Fun Seeking* [182]	Behavior Inhibitory System/Behavior Approach System (BIS/BAS)	801	11.80	2.10	Doctors score 11.80 out of 16, where higher score = higher association to the personality trait.
	*BAS Reward Response* [182]	Behavior Inhibitory System/Behavior Approach System (BIS/BAS)	801	11.40	1.80	Doctors score 11.80 out of 16, where higher score = higher association to the personality trait.
	*Coherence* [124, 211]	Sense of Coherence	553	5.05	0.05	Doctors score 5.05 out of 7, where higher score = higher association to personality trait.
	*Control* [212, 229]	Basic Character Inventory (BCI)	129	3.90	1.74	Doctors score 3.90 out of 9, where higher score = more association with personality trait
		Eysenck Personality Questionnaire - Revised (EPQ-R)	255	11.29	0.37	Information not available for interpretation
	*Conscientiousness* [100, 147, 150, 155–157, 162, 165, 166, 168–174, 177, 180, 184]	Big Five Inventory	1572	4.08	0.27	Doctors score 4.08 out of 5, where higher score = higher association to personality trait.
		Big Five Inventory (BFI) *Subscale Sum Score*	563	38.63	3.95	*Information not available for interpretation*
		NEO Five-Factor Inventory *Sten Score*	50	8.60	1.10	Doctors exhibit average levels of Conscientiousness.
		NEO Five-Factor Inventory *5 point scale*	872	34.75	2.74	Doctors score 34.75 out of 60, where higher score = higher association to personality trait.
		NEO Personality Inventory Revised *5 point scale*	32	4.00	0.40	Doctors score 4.00 out of 5, where higher score = higher association to personality trait.
		NEO Personality Inventory Revised (NEO-PI-R)	984	125.67	42.04	Doctors exhibit above level when compared to population norm.
		Five Factor Model of personality (FFM)	4068	3.83	0.13	Doctors score 3.83 out of 5, where higher score = higher association to personality trait.
		Mini-markers of the Big Five Factor Structure personality scale	70	6.50		*Information not available for interpretation*
	*Cooperativeness* [1, 148, 149, 151, 163]	Temperament and Character Inventory	907	80.80	0.00	Doctors exhibit high cooperativeness, when compared to population norms.
		Temperament and Character Inventory - mean score	545	3.98	0.13	Doctors score 3.98 out of 5, where higher score = higher association to personality trait.
	*Cynicism* [176]	Minnesota Multiphasic Personality Inventory (MMPI)	440	8.67	1.45	*Information not available for interpretation*
	*Dogmatism* [161]	Rokeach Dogmatism Scale (D-20)	22	12.50		Doctors scored 12.50 out of 60, where high score = higher association with personality trait.
	*Dominance* [154]	Cattell’s Sixteen Personality Factor Form C	231	6.00	0.00	Doctors exhibit higher dominance than population norm.
	*Emotional Stability* [154, 155, 171]	Big Five Inventory	636	3.87	0.67	Doctors score 3.87 out of 5, where higher scores = higher association with personality trait.
		Cattell’s Sixteen Personality Factor Form C	231	5.34	1.83	Doctor exhibit slightly lower emotional stability than population norm.
		Mini-markers of the Big Five Factor Structure personality scale	70	6.10		*Information not available for interpretation*
	*Expressive* [211]	Personal Attribute Questionnaire	504	4.87	0.05	Doctors score 4.87 out of 6 expressiveness, suggesting they utilise socially desirable expressiveness and communal traits.
	*Extroversion* [90, 100, 150, 152, 153, 155–158, 160, 162, 165, 168–174, 176, 177, 180, 184, 204, 212]	NEO Five-Factor Inventory	50	9.70	0.70	Doctors exhibit high levels of extroversion.
		NEO Personality Inventory Revised	32	3.61	0.60	Doctors exhibit same extroversion levels as population norms.
		Eysenck Personality Questionnaire	149	14.30	4.49	*Information not available for interpretation*
		Big Five Inventory	1475	3.47	0.80	Doctors score 3.47 out of 5, where higher scores = higher association with personality trait.
		Big Five Inventory (BFI) *Subscale Sum Score*	563	25.95	0.00	*Information not available for interpretation*
		Eysenck Personality Questionnaire - Revised (EPQ-R)	255	12.20	0.31	*Information not available for interpretation*
		Eysenck Personality Questionnaire - Revised Short Scale	1244	5.55	1.13	Doctors scored 5.55 out of 12, where higher score = higher association with personality trait.
		NEO Five-Factor Inventory *5 point scale*	872	31.41	5.08	Doctors score 31.41 out of 60, where higher score = higher association to personality trait.
		NEO Personality Inventory Revised (NEO-PI-R)	984	118.35	20.30	NEO Personality Inventory Revised (NEO-PI-R)
		Basic Character Inventory (BCI)	129	5.70	2.61	Basic Character Inventory (BCI)
		Five Factor Model of personality (FFM)	4068	3.43	0.13	Five Factor Model of personality (FFM)
		Mini-markers of the Big Five Factor Structure personality scale	70	5.70		Mini-markers of the Big Five Factor Structure personality scale
		Minnesota Multiphasic Personality Inventory (MMPI)	440	16.85	3.54	Minnesota Multiphasic Personality Inventory (MMPI)
		Myers-Briggs Type Indicator (MBTI)	98	9.50		Myers-Briggs Type Indicator (MBTI)
	*Feeling* [160]	Myers-Briggs Type Indicator (MBTI)	98	10.22	1.70	*Information not available for interpretation*
	*Harm avoidance* [1, 148, 149, 151, 163]	Temperament and Character Inventory	907	54.31	0.00	Doctors exhibit average harm avoidance, when compared to population norms.
		Temperament and Character Inventory - mean score	545	2.71	0.00	Doctors score 2.71 out of 5, where higher score = higher association to personality trait.
	*Inadequacy* [176]	Minnesota Multiphasic Personality Inventory (MMPI)	440	4.55	4.89	*Information not available for interpretation*
	*Intellectual Interest* [176]	Minnesota Multiphasic Personality Inventory (MMPI)	440	8.67	1.45	*Information not available for interpretation*
	*Introversion* [160]	Myers-Briggs Type Indicator (MBTI)	98	11.47	0.00	*Information not available for interpretation*
	*Intuition* [160]	Myers-Briggs Type Indicator (MBTI)	98	10.14	1.43	*Information not available for interpretation*
	*Instrumentality* [211]	Personal Attribute Questionnaire	504	4.15	0.05	Doctors score 4.15 out of 6 for instrumentality, suggesting they are independent and decisive.
	*Judgement* [160]	Myers-Briggs Type Indicator (MBTI)	98	14.38	2.04	*Information not available for interpretation*
	*Liveliness* [154]	Cattell’s Sixteen Personality Factor Form C	231	4.61	0.68	Doctor exhibit lower liveliness than population norm.
	*Masculinity/femininity* [176]	Minnesota Multiphasic Personality Inventory (MMPI)	440	29.54	3.36	*Information not available for interpretation*
	*Machiavelism* [58]	MACH-IV Test of Machiavellianism	199	52.88	1.18	Doctors score 52.88 out of 100, where higher scores = higher Machiavellianism.
	*Narcissism* [58, 185]	Narcissistic Personality Inventory	199	11.66	2.36	Doctors score 11.66 out of 40, where higher scores = higher narcissism.
	*Neuroticism* [90, 100, 147, 150, 152, 153, 155–158, 162, 165–170, 172–174, 176–178, 180, 184, 212]	Big Five Inventory	936	2.37	0.32	Doctors exhibit lower levels of neuroticism.
		Big Five Inventory (BFI) *Subscale Sum Score*	563	19.59	0.79	*Information not available for interpretation*
		NEO Five-Factor Inventory *Sten Score*	50	8.40	1.20	Doctors exhibit average levels of neuroticism.
		Eysenck Personality Questionnaire - Revised (EPQ-R)	255	8.83	0.00	Doctor exhibit lower level of neuroticism
		Eysenck Personality Questionnaire - Revised Short Scale	1251	2.90	0.64	Doctors scored 2.90 out of 12, where higher score = higher association with personality trait.
		NEO Five-Factor Inventory *5 point scale*	872	24.73	8.60	Doctors score 24.73 out of 60, where higher scores = higher association with personality trait.
		NEO Personality Inventory Revised *5 point scale*	32	3.23	0.50	Doctors exhibit lower levels of neuroticism compared to population norms.
		NEO Personality Inventory Revised (NEO-PI-R)	984	64.89	8.78	Doctors exhibit lower levels when compared to population norm.
		Eysenck Personality Questionnaire	333	3.34	4.52	Doctors exhibit lower levels of neuroticism.
		Basic Character Inventory (BCI)	129	3.10	2.03	Doctors score 3.10 out of 9, where higher score = more association with personality trait
		Five Factor Model of personality (FFM)	4068	2.67	2.67	Doctors score 2.67 out of 5, where higher score = higher association to personality trait.
		Mini-International Personality Item Pool (Mini-IPIP)	22	10.31	2.80	Doctors demonstrate lower levels of neuroticism.
		Minnesota Multiphasic Personality Inventory (MMPI)	440	11.59	3.54	*Information not available for interpretation*
	*Novelty Seeking* [1, 148, 149, 151, 163]	Temperament and Character Inventory	907	52.38	1.00	Doctors exhibit average novelty seeking, when compared to population norms.
		Temperament and Character Inventory - mean score	550	2.66	0.05	Doctors score 2.66 out of 5, where higher scores = higher association with personality trait.
	*Openness* [100, 150, 154–157, 162, 165, 166, 168–175, 177, 180, 184]	Big Five Inventory	1475	3.54	0.09	Doctors score 3.54 out of 5, where higher scores = higher association with personality trait.
		Big Five Inventory (BFI) *Subscale Sum Score*	563	35.13	0.00	*Information not available for interpretation*
		NEO Five-Factor Inventory *Sten Score*	50	8.70	1.10	Doctors exhibit average levels of openness.
		NEO Five-Factor Inventory *5 point scale*	872	30.00	7.11	Doctors score 29.63 out of 60, where higher scores = higher association with personality trait.
		NEO Personality Inventory Revised *5 point scale*	32	4.11	0.60	Doctors exhibit higher openness than population norms.
		NEO Personality Inventory Revised (NEO-PI-R)	984	118.52	19.45	Doctors exhibit above level when compared to population norm.
		Cattell’s Sixteen Personality Factor Form C	231	5.23	0.00	Doctor exhibit lower openness than population norm.
		Five Factor Model of personality (FFM)	4068	3.32	0.13	Doctors score 3.32 out of 5, where higher score = higher association to personality trait.
		Mini-markers of the Big Five Factor Structure personality scale	70	6.80		*Information not available for interpretation*
		Resiliency Assessment Scale	655	15.52	2.77	*Information not available for interpretation*
	*Optimism* [175]	Resiliency Assessment Scale	655	13.85	3.40	*Information not available for interpretation*
	*Perfectionism* [154]	Cattell’s Sixteen Personality Factor Form C	231	5.61	0.68	Doctors exhibit slightly higher perfectionism than population norm.
	*Persistence* [1, 148, 149, 151, 163, 175]	Temperament and Character Inventory	907	71.16	0.00	Doctors exhibit high persistence, when compared to population norms.
		Temperament and Character Inventory - mean score	547	3.54	0.05	Doctors score 3.54 out of 5, where higher score = higher association with personality trait.
		Resiliency Assessment Scale	655	14.76	3.28	*Information not available for interpretation*
	*Perception* [160]	Myers-Briggs Type Indicator (MBTI)	98	7.58	2.05	*Information not available for interpretation*
	*Psychotism* [58, 90, 153, 158, 176]	Levenson Self-Report Psychopathy Scale	199	44.08	1.21	Doctors score 44.08 out of 104, where higher scores = higher psychopathy.
		Eysenck Personality Questionnaire - Revised Short Scale	1076	2.86	0.64	Doctors scored 2.86 out of 12, where higher score = higher association with personality trait.
		Eysenck Personality Questionnaire - Revised (EPQ-R)	255	5.85	0.46	*Information not available for interpretation*
		Eysenck Personality Questionnaire	149	3.00	1.50	Doctors exhibit lower levels of psychoticism.
		Minnesota Multiphasic Personality Inventory (MMPI)	440	3.88	2.67	*Information not available for interpretation*
	*Reasoning* [154]	Cattell’s Sixteen Personality Factor Form C	231	6.63	0.10	Doctors exhibit higher reasoning than population norm.
	*Reward Dependence* [1, 148, 149, 151, 163]	Temperament and Character Inventory	907	67.58	2.89	Doctors exhibit high reward dependence, when compared to population norms.
		Temperament and Character Inventory - mean score	549	3.46	0.00	Doctors score 3.46 out of 5, where higher score = higher association with personality trait.
	Resilience [175]	Resiliency Assessment Scale	655	75.52	16.84	*Information not available for interpretation*
	*Risk Attitude / Readiness* [143, 159]	Jackson Personality Inventory-revised	829	2.90		Doctors scored 2.90 out of 6, where higher scores = more risk prone.
		Ten-Item Personality Inventory (TIPI)	64	−0.48	3.19	*Information not available for interpretation*
	*Rule-consciousness* [154]	Cattell’s Sixteen Personality Factor Form C	231	5.40	0.00	Doctor exhibit slightly lower rule-consciousness than population norm.
	*Self-directedness* [1, 148, 149, 151, 163]	Temperament and Character Inventory	907	77.81	0.80	Doctors exhibit high self-directedness, when compared to population norms.
		Temperament and Character Inventory - mean score	543	3.79	0.04	Doctor score 3.79 out of 5, where higher score = higher association to personality trait.
	*Self-reliance* [154]	Cattell’s Sixteen Personality Factor Form C	231	7.10	0.00	Doctors exhibit higher self-reliance than population norm.
	*Self-transcendence* [1, 148, 149, 151, 163]	Temperament and Character Inventory	907	41.28	0.45	Doctors exhibit average self-transcendence, when compared to population norms.
		Temperament and Character Inventory - mean score	550	2.64	0.00	Doctor score 2.64 out of 5, where higher score = higher association to personality trait.
	*Sensing* [160]	Myers-Briggs Type Indicator (MBTI)	98	15.82	1.38	*Information not available for interpretation*
	*Sensitivity* [154]	Cattell’s Sixteen Personality Factor Form C	231	5.67	0.00	Doctors exhibit higher sensitivity than population norm.
	*Shrewdness* [154]	Cattell’s Sixteen Personality Factor Form C	231	5.67	0.38	Doctors exhibit higher shrewdness than population norm.
	*Social boldness* [154]	Cattell’s Sixteen Personality Factor Form C	231	4.90	0.82	Doctor exhibit lower social boldness than population norm.
	*Tension* [154]	Cattell’s Sixteen Personality Factor Form C	231	6.29	1.90	Doctors exhibit higher tension than population norm.
	*Thinking* [160]	Myers-Briggs Type Indicator (MBTI)	98	13.75	1.66	*Information not available for interpretation*
	*Tolerance of Failure / Viewing Life as a challenge* [175]	Resiliency Assessment Scale	655	14.82	3.33	*Information not available for interpretation*
	*Type D* [175]	Type D Personality Scale (DS-14)	655	21.05	10.58	*Information not available for interpretation*
	*Vigilance* [154, 159]	Cattell’s Sixteen Personality Factor Form C	231	5.34	0.75	Doctor exhibit lower vigilance than population norm.
		Ten-Item Personality Inventory (TIPI)	64	16.31	2.05	*Information not available for interpretation*
	*Warmth* [154]	Cattell’s Sixteen Personality Factor Form C	231	4.90	0.00	Doctor exhibit lower warmth than population norm.
**Nursing**	*Abstractedness* [52, 106]	Cattell’s Sixteen Personality Factor	159	12.13	0.04	*Information not available for interpretation*
		16F Personality Indicator	69	5.41	0.12	Nurses personality trait of abstraction is slightly lower than the general population.
	*Abasement* [138]	Edwards Personal Preference Schedule (EPPS)	445	12.89	5.25	*Information not available for interpretation*
	*Achievement* [138]	Edwards Personal Preference Schedule (EPPS)	445	15.25	4.18	*Information not available for interpretation*
	*Achievement* via *conformance* [57]	California Psychological Inventory	36	31.00	3.66	*Information not available for interpretation*
	*Achievement* via *independence* [57]	California Psychological Inventory	36	22.67	3.24	*Information not available for interpretation*
	*Affiliation* [138]	Edwards Personal Preference Schedule (EPPS)	445	15.10	3.56	*Information not available for interpretation*
	*Agreeableness* [9, 53–55, 60, 61, 64, 70, 74, 75, 77–80, 82, 86, 96, 100, 102–105, 108, 112–114, 119, 123, 126, 127, 129–132, 136, 140]	Big Five Inventory	2977	3.96	1.45	Doctors score 3.96 out of 5, where higher scores = higher association with personality trait.
		Big Five Inventory *Sum Score*	1695	34.55	4.14	Nurses scored 34.55 out 45, where higher scores = higher association with personality trait.
		Big Five Inventory - Short Form	207	4.61	0.90	Nurses scored 4.61 out of 7, where higher scores = higher association with personality trait.
		Chinese Big Five Personality Inventory Brief Version	471	38.62	5.39	Nurses scored 38.62 out of 48, where higher score = higher association with personality trait.
		NEO Five-Factor Inventory *5 point scale*	1215	39.34	9.52	Nurses score 9.34 out of 60, where higher score = higher association to personality trait.
		NEO Five-Factor Inventory *Original*	99	51.06	6.46	*Information not available for interpretation*
		NEO Five-Factor Inventory (NEO FFI) *Sten score*	954	5.76	0.08	Nurses exhibit average levels of agreeableness.
		NEO Personality Inventory *240 items*	203	44.43	8.15	Nurses demonstrate a medium level of agreeableness.
		Five-Factor Personality Inventory	237	3.15	0.41	Nurses score 3.15 out of 5, where higher score = higher association with personality trait.
		Five-Factor Inventory	1246	20.32	2.92	Nurses scored 20.32 out of 30, where higher scores = higher association with personality trait.
		NEO Personality Inventory Revised	319	3.76	0.27	Nurses scored 3.76 out of 5, where higher score = higher association with personality trait.
		Ten-Item Personality Inventory	420	7.05	4.14	*Information not available for interpretation*
		Ten-Item Personality Inventory - Japanese (TIPI-J)	211	4.78	0.96	*Information not available for interpretation*
		Revised Short-Form Personality 5-Factor Model	138	14.44	2.62	Nurses scored 14.44 out of 21, where higher score = higher association with personality trait.
		International Personality Item Pool	41	4.36	0.57	Nurses score 4.36 out of 5, where higher score = higher association with personality trait.
		NEO Personality Inventory - 3	72	125.90	17.50	Nurses exhibit higher levels of agreeableness than population norms.
		HEXACO Personality Inventory-Revised	138	2.93	0.00	Nurses scored 2.93 out of 5, where higher score = higher association with personality trait.
		10-Item Big Five Inventory (BFI-10)	518	3.41	0.81	Nurses scored 3.41 out of 5, where higher score = higher association with personality trait.
		Big Five Personality Scale (BFPS)	176	35.88	4.88	Nurses demonstrate higher than average levels of agreeableness.
	*Anger Control* [76, 138]	Trait Anger–Anger Expression Scales	370	22.86	4.27	Nurses score 22.86 out of 32, where higher score indicate a greater control of anger.
		Edwards Personal Preference Schedule (EPPS)	445	10.78	3.80	*Information not available for interpretation*
	*Anger-In* [76]	Trait Anger–Anger Expression Scales	370	16.24	3.48	Nurses score 16.24 out of 32, where higher score indicate a greater suppression of anger.
	*Anger-Out* [76]	Trait Anger–Anger Expression Scales	370	15.38	3.13	Nurses score 15.38 out of 32, where higher score indicate an easy expression of anger.
	*Anxiety* [52, 106]	16F Personality Indicator	140	5.55	0.56	Nurses personality trait of anxiety is the same as the general population.
	*Apprehension* [52, 106]	16F Personality Indicator	69	6.25	0.00	Nurses’ personality trait of abstraction is slightly lower than the general population.
		Cattell’s Sixteen Personality Factor	159	9.58	0.00	*Information not available for interpretation*
	*Assertiveness* [50]	16F Personality Indicator	71	6.14	1.11	Nurses personality trait of assertiveness is higher than the general population.
	*Autonomy* [76, 117, 138]	Sociotropy– autonomy Scale	392	75.16	2.05	Nurses score 75.16 out of 120, where higher score indicates high level of autonomy.
		Edwards Personal Preference Schedule (EPPS)	445	11.85	4.17	*Information not available for interpretation*
	*Boldness* [52]	16F Personality Indicator	69	5.31	0.00	Nurses personality trait of boldness is slightly lower than the general population.
	*Capacity for status* [57]	California Psychological Inventory	36	22.36	2.72	*Information not available for interpretation*
	*Communality* [57]	California Psychological Inventory	36	25.69	2.21	*Information not available for interpretation*
	*Compassionate* [88]	Myers-Briggs Type Indicator	882	3.55	0.56	Nurses scored 3.55 out of 5, where higher scores = higher association with personality trait.
	*Conscientiousness* [9, 53–55, 60, 61, 64, 70, 74, 75, 77–80, 82, 86, 96, 100, 102–105, 112–114, 119, 123, 127, 129–132, 136, 140, 213]	Big Five Inventory	2977	3.94	1.46	Doctors score 3.94out of 5, where higher scores = higher association with personality trait.
		Big Five Inventory *Sum Score*	1695	36.93	3.62	Nurses scored 36.93 out 45, where higher scores = higher association with personality trait.
		Big Five Inventory - Short Form	522	3.85	0.00	Nurses scored 3.85 out of 7, where higher scores = higher association with personality trait.
		Chinese Big Five Personality Inventory Brief Version	471	37.93	6.50	Nurses scored 37.93 out of 48, where higher score = higher association with personality trait.
		NEO Five-Factor Inventory *5 point scale*	1597	40.56	9.14	Nurses score 40.56 out of 60, where higher score = higher association to personality trait.
		NEO Five-Factor Inventory *Original*	99	49.22	8.60	*Information not available for interpretation*
		NEO Five-Factor Inventory (NEO FFI) *Sten score*	954	6.40	0.00	Nurses exhibit average levels of Conscientiousness.
		NEO Personality Inventory *240 items*	203	44.25		Nurses demonstrate a medium level of conscientiousness.
		Five-Factor Personality Inventory	237	3.31	0.45	Nurses score 3.31 out of 5, where higher score = higher association with personality trait.
		16F Personality Indicator	71	5.53	0.39	Nurses personality trait of conscientiousness is the same as the general population.
		Five-Factor Inventory	1246	20.25	2.40	Nurses scored 20.52 out of 30, where higher scores = higher association with personality trait.
		NEO Personality Inventory Revised	319	3.75	0.34	Nurses scored 3.75 out of 5, where higher score = higher association with personality trait.
		Ten-Item Personality Inventory	420	9.26	4.98	*Information not available for interpretation*
		Ten-Item Personality Inventory - Japanese (TIPI-J)	211	3.74	0.99	*Information not available for interpretation*
		Revised Short-Form Personality 5-Factor Model	138	20.92	3.92	Nurses scored 20.92 out of 28, where higher score = higher association with personality trait.
		International Personality Item Pool	41	4.10	0.55	Doctors scores 4.10 out of 5, where higher scores = higher association with personality trait.
		HEXACO Personality Inventory-Revised	138	3.67	0.00	Nurses scored 3.67 out of 5, where higher score = higher association with personality trait.
		10-Item Big Five Inventory (BFI-10)	518	4.08	0.77	Nurses scored 4.08 out of 5, where higher score = higher association with personality trait.
	*Control* [50, 212]	16F Personality Indicator	71	5.81	0.00	Nurses personality trait of control is higher than the general population.
		Basic Character Inventory (BCI)	16	2.90	2.25	Nurses score 2.9 out of 9, where higher score = more association with personality trait
	*Conformity* [115]	PROSCAN	76	4.67	1.13	Nurses scored 4.67 out of 5, where higher scores = higher association with personality trait.
	*Cooperativeness* [1, 66, 141]	Temperament and Character Inventory (TCI-R 140)	411	80.13	0.00	Nurses exhibit High cooperativeness, when compared to population norms.
		Temperament and Character Inventory (TCI-240)	287	32.40	4.50	Information not available for interpretation
	*Change* [138]	Edwards Personal Preference Schedule (EPPS)	445	16.62	4.63	*Information not available for interpretation*
	*Dominance* [52, 57, 106, 115, 138]	16F Personality Indicator	69	5.74	1.24	Nurses personality trait of dominance is higher than the general population.
		California Psychological Inventory	36	31.52	4.58	*Information not available for interpretation*
		Cattell’s Sixteen Personality Factor	159	10.28	1.52	*Information not available for interpretation*
		Edwards Personal Preference Schedule (EPPS)	445	12.65	4.71	*Information not available for interpretation*
		PROSCAN	76	3.51	1.40	Nurses scored 3.51out of 5, where higher scores = higher association with personality trait.
	*Deference* [138]	Edwards Personal Preference Schedule (EPPS)	445	13.41	3.33	*Information not available for interpretation*
	*Easy Going* [88]	Myers-Briggs Type Indicator	882	3.92	0.55	Nurses scored 3.92 out of 5, where higher scores = higher association with personality trait.
	*Ego strength* [50]	16F Personality Indicator	71	5.48	0.15	Nurses personality trait of ego strength is the same than the general population.
	*Emotional Stability* [9, 60, 61, 82, 86, 103, 104, 106]	Five-Factor Personality Inventory	237	2.96	0.51	Nurses score 2.96 out of 5, where higher score = higher association with personality trait.
		Five-Factor Inventory	1246	18.60	4.53	Nurses scored 18.60 out of 30, where higher scores = higher association with personality trait.
		NEO Personality Inventory Revised	173	3.37	0.67	Nurses scored 3.37 out of 5, where higher score = higher association with personality trait.
		Ten-Item Personality Inventory	420	7.46	4.65	*Information not available for interpretation*
		International Personality Item Pool	41	3.35	0.65	Nurses scored 3.35 out of 5 where higher score = higher association with personality trait.
		Cattell’s Sixteen Personality Factor	159	9.75	0.00	*Information not available for interpretation*
		HEXACO Personality Inventory-Revised	138	2.89	0.19	Nurses scored 2.89 out of 5, where higher score = higher association with personality trait.
	*Endurance* [138]	Edwards Personal Preference Schedule (EPPS)	445	15.97	4.59	*Information not available for interpretation*
	*Expressive* [50]	16F Personality Indicator	71	5.60	0.00	Nurses personality trait of ego expressive is the same than the general population.
	*Extroversion* [9, 53–55, 60–62, 64, 70, 72, 74, 75, 77–80, 82, 84, 86, 90, 93, 96, 100, 102–105, 108, 109, 112–115, 119, 123, 126, 127, 129–132, 136, 140, 142, 212, 213]	Five-Factor Inventory	1246	18.95	2.44	Nurses scored 18.95 out of 30, where higher scores = higher association with personality trait.
		NEO Personality Inventory Revised	319	3.26	0.39	Nurses scored 3.26 out of 5, where higher score = higher association with personality trait.
		Ten-Item Personality Inventory	420	5.72	2.87	*Information not available for interpretation*
		Ten-Item Personality Inventory - Japanese (TIPI-J)	211	4.42	1.38	*Information not available for interpretation*
		Revised Short-Form Personality 5-Factor Model	138	14.54	2.86	Nurses scored 14.54 out of 21, where higher score = higher association with personality trait.
		NEO Personality Inventory - 3	72	121.80	19.90	Nurses demonstrate higher than population norm, indicating friendly individuals who engage with conversation easily.
		Eysenck Personality Questionnaire	320	14.65	3.72	*Information not available for interpretation*
		Big Five Inventory	2977	3.33	0.74	Doctors score 3.33 out of 5, where higher scores = higher association with personality trait.
		Big Five Inventory *Subscale Sum Score*	1695	28.73	7.14	Nurses scored 28.73 out 40, where higher scores = higher association with personality trait.
		Big Five Inventory - Short Form	522	4.75	0.00	Nurses scored 4.75 out of 7, where higher scores = higher association with personality trait.
		Chinese Big Five Personality Inventory Brief Version	471	29.93	6.44	Nurses scored 29.93 out of 48, where higher score = higher association with personality trait.
		NEO Five-Factor Inventory *5 point scale*	1938	38.32	6.33	Nurses score 38.32 out of 60, where higher score = higher association to personality trait.
		NEO Five-Factor Inventory *Original*	99	115.97	20.62	Extroversion is the highest personality trait of five factor model demonstrated by nurses.
		NEO Five-Factor Inventory (NEO FFI) *Sten score*	954	5.84	0.13	Nurses exhibit average levels of extraversion
		NEO Personality Inventory *240 items*	203	44.70	6.96	Nurses demonstrate a medium level of extraversion.
		Five-Factor Personality Inventory	237	3.18	0.46	Nurses score 3.18 out of 5, where higher score = higher association with personality trait.
		16F Personality Indicator	140	6.20	0.60	Nurses personality trait of extroversion is higher than the general population.
		Eysenck Personality Questionnaire - Revised Short Scale	823	5.64	0.19	Nurses scored 5.64 out of 12, where higher score = higher association with personality trait.
		Eysenck Personality Questionnaire - Revised	96	14.32	0.00	*Information not available for interpretation*
		HEXACO Personality Inventory-Revised	138	3.46	0.04	Nurses scored 3.46 out of 5, where higher score = higher association with personality trait.
		10-Item Big Five Inventory (BFI-10)	518	3.49	1.00	Nurses scored 3.49 out of 5, where higher score = higher association with personality trait.
		Basic Character Inventory (BCI)	16	6.10	2.53	Doctors score 2.53 out of 9, where higher score = more association with personality trait
		Big Five Personality Scale (BFPS)	176	28.64	5.02	Nurses exhibit lower than average extraversion.
		International Personality Item Pool (IPIP)	311	3.54	0.76	Nurses scored 3.54out of 5, where higher scores = higher association with personality trait.
		Personality-trait questionnaire	20	16.55	2.37	Nurses scored 16.55 out of 20, where higher scores = higher association
		PROSCAN	76	3.95	1.44	Nurses scored 3.95 out of 5, where higher scores = higher association with personality trait.
	*Extroversion/Introversion* [50]	Myers-Briggs Type Indicator	71	104.51	0.00	Nurses demonstrate slightly more introversion than extroversion.
	*Exhibition* [138]	Edwards Personal Preference Schedule (EPPS)	445	12.14	3.78	*Information not available for interpretation*
	*Femininity* [57, 65]	California Psychological Inventory	70	18.82	6.48	*Information not available for interpretation*
	*Flexibility* [57]	California Psychological Inventory	36	11.14	3.57	*Information not available for interpretation*
	*Forthrightness* [50]	16F Personality Indicator	71	4.18	0.85	Nurses personality trait of forthrightness is lower than the general population.
	*Friendliness* [62, 107]	NEO Five-Factor Inventory (NEO FFI) 5 point scale	338	45.16	0.54	Nurses score 45.16 out of 60, where higher score = higher association to personality trait.
	*Friendliness* [62]	NEO Five-Factor Inventory *5 point scale*	96	45.81	5.90	Nurses score 45.81 out of 60, where higher score = higher association to personality trait.
	*Good Impression* [57, 65]	California Psychological Inventory	70	19.64	1.81	*Information not available for interpretation*
	*Hardiness* [53, 63, 83, 97, 101, 122, 128, 133, 134, 139]	Hardiness of Personality Inventory	287	77.10	7.80	Nurses scored 77.10 out of 100, where higher scores = higher hardiness.
		Hardiness Test	80	72.00	0.00	Nurses scored 72. 00 out of 100, where higher scores = higher hardiness and is a negative predictor of burnout.
		Hardiness Scale	253	74.31	16.89	Nurses scores 74.31 out of 100, where higher scores = higher hardiness and is linked to lower stress and emotional exhaustion.
		The Short Hardiness Inventory	192	33.85	8.50	Nurses scored 33.85 out of 45, where higher scores = higher hardiness.
		Third Generation Personal Views (Hardiness) questionnaire	62	75.92	2.08	*Information not available for interpretation*
		Abridged Hardiness Scale	107	0.00	2.17	Information not available for interpretation
		Dispositional Resilience (hardiness) Scale-Revised (DRS15R)	3607	31.55	0.04	Information not available for interpretation
	*Harm Avoidance* [1, 66, 141]	Temperament and Character Inventory (TCI-R 140)	411	54.24	0.00	Nurses exhibit average harm avoidance, when compared to population norms.
		Temperament and Character Inventory (TCI-240)	287	15.30	6.20	*Information not available for interpretation*
	*Heterosexuality* [138]	Edwards Personal Preference Schedule (EPPS)	445	12.77	6.35	*Information not available for interpretation*
	*Honesty/Humility* [104]	HEXACO Personality Inventory-Revised	138	3.84	0.22	Nurses scored 3.84 out of 5, where higher score = higher association with personality trait.
	*Imagination* [50]	16F Personality Indicator	71	5.23	0.64	Nurses personality trait of imagination is lower than the general population.
	*Independence* [50, 52]	16F Personality Indicator	140	5.17	0.82	Nurses personality trait of independence is lower than the general population.
		California Psychological Inventory	34	18.51	0.47	*Information not available for interpretation*
	*Intelligence* [50, 57, 65]	California Psychological Inventory	70	37.61	8.78	*Information not available for interpretation*
		16F Personality Indicator	71	6.82	0.26	Nurses personality trait of intelligence is higher than the general population.
	*Intraception* [138]	Edwards Personal Preference Schedule (EPPS)	445	16.42	4.10	*Information not available for interpretation*
	*Judgement/Perception* [50]	Myers-Briggs Type Indicator	71	95.27	6.36	Nurses exhibit more judgement than perception but is similar to population norms.
	*Lie* [72, 84, 142]	Eysenck Personality Questionnaire - Revised Short Scale	116	6.80	2.00	Nurses scored 6.80 out of 12, where higher score = higher association with personality trait.
		Eysenck Personality Questionnaire - Revised	96	12.20	0.00	*Information not available for interpretation*
		Personality-trait questionnaire	23	14..38	1.66	Nurses scored 14.38 out of 23, where higher scores = higher association
	*Liveliness* [52, 106]	16F Personality Indicator	69	5.10	0.00	Nurses personality trait of liveliness is lower than the general population.
		Cattell’s Sixteen Personality Factor	159	10.87	0.33	*Information not available for interpretation*
	*Logic* [115]	PROSCAN	76	3.66	1.14	Nurses scored 3.66 out of 5, where higher scores = higher association with personality trait.
	*Machiavelism* [58]	MACH-IV Test of Machiavellianism	49	53.50	8.36	Nurses scored 53.50 out of 100, where higher scores = higher levels of Machiavellianism.
	*Narcissism* [58]	Narcissistic Personality Inventory	49	11.10	5.57	Nurses score 11.10 out of 40, where higher scores = higher narcissism.
	*Neuroticism* [9, 49, 53–55, 60–62, 64, 70, 72, 74, 75, 77–80, 82, 84, 86, 90, 93, 96, 100, 102–105, 107, 108, 112–114, 119, 123, 125–127, 129–132, 135, 136, 140, 142, 212]	Big Five Inventory	3243	2.66	0.15	Doctors score 2.66out of 5, where higher scores = higher association with personality trait.
		Big Five Inventory *Subscale Sum Score*	1956	21.80	4.02	Nurses scored 21.93 out 45, where higher scores = higher association with personality trait.
		Big Five Inventory - Short Form	207	4.51	1.29	Nurses scored 4.51 out of 7, where higher scores = higher association with personality trait.
		Chinese Big Five Personality Inventory Brief Version	471	25.32	8.71	Nurses scored 25.32 out of 48, where higher score = higher association with personality trait.
		NEO Five-Factor Inventory *Sten score*	1058	4.50	0.36	Nurses exhibit low levels of neuroticism.
		NEO Five-Factor Inventory *5 point scale*	1574	25.10	3.75	Nurses score 25.10 out of 60, where higher score = higher association to personality trait.
		NEO Five-Factor Inventory *Original*	99	77.61	20.62	*Information not available for interpretation*
		NEO Personality Inventory *240 items*	203	30.30	12.12	Nurses demonstrate a lower level of neuroticism.
		Eysenck Personality Questionnaire - Revised Short Scale	823	7.62	1.26	Nurses scored 7.62 out of 12, where higher score = higher association with personality trait.
		Eysenck Personality Questionnaire - Revised	96	14.69	0.00	*Information not available for interpretation*
		NEO Personality Inventory Revised	146	2.30	0.60	Nurses scored 2.30 out of 5, where higher score = higher association with personality trait.
		Revised Short-Form Personality 5-Factor Model	138	17.25	2.82	Nurses scored 17.25 out of 28, where higher score = higher association with personality trait.
		10-Item Big Five Inventory (BFI-10)	518	2.73	0.94	Nurses scored 2.73 out of 5, where higher score = higher association with personality trait.
		Eysenck Personality Questionnaire	320	14.30	4.49	*Information not available for interpretation*
		Basic Character Inventory (BCI)	16	2.80	1.88	Doctors score 2.80 out of 9, where higher score = more association with personality trait
		Big Five Personality Scale (BFPS)	176	25.57	4.16	Nurses demonstrate lower than average levels of neuroticism.
		Neuroticism Scale	139	18.34	5.80	Information not available for interpretation
		Ten-Item Personality Inventory - Japanese (TIPI-J)	211	2.45	1.19	*Information not available for interpretation*
		Personality-trait questionnaire	23	17.40	3.51	Nurses scored 17.40 out of 23, where higher scores = higher association
	*Nurturance* [138]	Edwards Personal Preference Schedule (EPPS)	445	16.65	4.31	*Information not available for interpretation*
	*Novelty Seeking* [1, 66, 141]	Temperament and Character Inventory (TCI-R 140)	411	54.48	0.24	Nurses exhibit average novelty seeking, when compared to population norms.
		Temperament and Character Inventory (TCI-240)	287	18.10	4.40	*Information not available for interpretation*
	*Openness* [9, 53–55, 59–62, 64, 70, 72, 74, 75, 77–80, 82, 84, 86, 90, 93, 96, 100, 102–105, 107, 108, 112–114, 119, 123, 126, 127, 129–132, 136, 140]	Big Five Inventory	3398	4.57	1.52	Doctors score 4.57 out of 5, where higher scores = higher association with personality trait.
		Big Five Inventory *Subscale Sum Score*	1695	33.67	1.92	Nurses scored 33.67 out 50, where higher scores = higher association with personality trait.
		Big Five Inventory - Short Form	522	4.81	0.00	Nurses scored 4.81 out of 7, where higher scores = higher association with personality trait.
		Chinese Big Five Personality Inventory Brief Version	471	33.03	7.33	Nurses scored 33.03 out of 48, where higher score = higher association with personality trait.
		NEO Five-Factor Inventory *5 point scale*	1949	33.67	8.68	Nurses score 33.67 out of 60, where higher score = higher association to personality trait.
		NEO Five-Factor Inventory *Original*	99	115.70	16.25	*Information not available for interpretation*
		NEO Five-Factor Inventory (NEO FFI) *Sten score*	954	5.52	0.00	Nurses exhibit average levels of openess.
		NEO Personality Inventory *240 items*	203	24.00	8.72	Nurses demonstrate a lower level of openness.
		Five-Factor Personality Inventory	237	3.31	0.52	Nurses scored 3.31 out of 5, where higher scores = higher association with personality trait.
		16F Personality Indicator	69	5.62	0.22	Nurses personality trait of Openness is higher than the general population.
		Five-Factor Inventory	1246	18.71	2.35	Nurses scored 18.71 out of 30, where higher scores = higher association with personality trait.
		NEO Personality Inventory Revised	319	3.38	0.48	Nurses scored 3.76 out of 5, where higher score = higher association with personality trait.
		Ten-Item Personality Inventory	420	6.96	3.73	*Information not available for interpretation*
		Ten-Item Personality Inventory - Japanese (TIPI-J)	211	3.79	1.06	*Information not available for interpretation*
		Revised Short-Form Personality 5-Factor Model	138	17.46	3.44	Nurses scored 17.46 out of 28, where higher score = higher association with personality trait.
		International Personality Item Pool	41	3.63	0.50	Nurses scored 3.63 out of 5, where higher scores = higher association with personality trait.
		NEO Personality Inventory - 3	72	119.90	19.20	Nurses demonstrate higher openness than population norm (107.7).
		Cattell’s Sixteen Personality Factor	159	11.52	0.25	*Information not available for interpretation*
		HEXACO Personality Inventory-Revised	138	3.37	0.06	Nurses scored 3.37 out of 5, where higher score = higher association with personality trait.
		10-Item Big Five Inventory (BFI-10)	518	3.53	0.98	Nurses scored 3.53 out of 5, where higher score = higher association with personality trait.
		Big Five Personality Scale (BFPS)	176	36.20	5.47	Nurses demonstrate higher than average openness.
	*Orderly* [88, 138]	Myers-Briggs Type Indicator	882	4.03	0.47	Nurses scored 4.03 out of 5, where higher scores = higher association with personality trait.
		Edwards Personal Preference Schedule (EPPS)	445	13.63	5.02	*Information not available for interpretation*
	*Pace* [115]	PROSCAN	76	4.86	1.15	Nurses scored 4.86 out of 5, where higher scores = higher association with personality trait.
	*Perfectionism* [52, 106]	16F Personality Indicator	69	5.16	0.35	Nurses’ personality trait of perfectionism is lower than the general population.
		Cattell’s Sixteen Personality Factor	159	12.93	0.67	*Information not available for interpretation*
	*Persistence* [1, 66, 141]	Temperament and Character Inventory (TCI-R 140)	411	70.98	0.00	Nurses exhibit high persistence, when compared to population norms.
		Temperament and Character Inventory (TCI-240)	287	5.70	1.80	*Information not available for interpretation*
	*Poise* [50]	16F Personality Indicator	71	6.02	0.23	Nurses’ personality trait of poise is higher than the general population.
	*Privateness* [52]	16F Personality Indicator	69	5.90	0.00	Nurses’ personality trait of privateness is higher than the general population.
	*Process* [50]	16F Personality Indicator	71	5.69	0.00	Nurses’ personality trait of process is higher than the general population.
	*Proactive* [110, 118, 121, 214]	Proactive Coping Scale	314	56.76	7.74	*Information not available for interpretation*
		Proactive Personality Scale (PPS)	1620	4.50	0.76	*Information not available for interpretation*
	*Psychotism* [57, 58, 72, 84, 90, 142]	California Psychological Inventory	36	16.56	2.24	*Information not available for interpretation*
		Levenson Self-Report Psychopathy Scale	49	47.30	8.70	Nurses scored 47.30 out of 104, where higher score = higher level psychopathy.
		Eysenck Personality Questionnaire - Revised Short Scale	116	6.40	1.40	Nurses scored 6.40 out of 12, where higher score = higher association with personality trait.
		Eysenck Personality Questionnaire - Revised	96	10.19	0.00	*Information not available for interpretation*
		Eysenck Personality Questionnaire	320	3.35	2.23	*Information not available for interpretation*
		Personality-trait questionnaire	25	21.41	0.86	Nurses scored 21.41 out of 25, where higher scores = higher association
	*Radicalism* [50]	16F Personality Indicator	71	5.71	0.76	Nurses’ personality trait of radicalism is higher than the general population.
	*Reasoning* [52]	16F Personality Indicator	69	6.10	0.00	Nurses’ personality trait of privateness is higher than the general population.
	*Reasoning* [106]	Cattell’s Sixteen Personality Factor	159	1.96	0.00	*Information not available for interpretation*
	*Receptive* [88]	Myers-Briggs Type Indicator	882	3.95	0.48	Nurses scored 3.95 out of 5, where higher scores = higher association with personality trait.
	*Resilience* [81]	Resilience Scale for Adults	140	134.00	71.81	Nurses demonstrate high resilience protective factors.
	*Responsibility* [62, 107, 108]	NEO Five-Factor Inventory *5 point scale*	338	47.02	6.10	Nurses scored 47.02 out of 60, where higher score = higher association to personality trait.
		California Psychological Inventory	36	31.92	4.28	*Information not available for interpretation*
		Big Five Personality Scale (BFPS)	176	35.25	5.39	Nurses demonstrate higher than average responsibility.
	*Reward Dependence* [1, 66, 141]	Temperament and Character Inventory (TCI-R 140)	411	69.69	0.00	Nurses exhibit high reward dependence, when compared to population norms.
		Temperament and Character Inventory (TCI-240)	287	15.20	3.20	*Information not available for interpretation*
	*Rule-consciousness* [52, 106]	16F Personality Indicator	69	5.48	0.00	Nurses’ personality trait of rule-consciousness is the same as the general population.
		Cattell’s Sixteen Personality Factor	159	8.85	0.00	*Information not available for interpretation*
	*Self-acceptance* [57]	California Psychological Inventory	36	23.14	3.34	*Information not available for interpretation*
	*Self-control* [52, 57]	16F Personality Indicator	69	5.97	0.40	Nurses’ personality trait of self-control is lower than the general population.
		California Psychological Inventory	36	33.06	6.03	*Information not available for interpretation*
	*Self-directedness* [1, 66, 141]	Temperament and Character Inventory (TCI-R 140)	411	74.96	0.00	Nurses exhibit high self-directedness, when compared to population norms.
		Temperament and Character Inventory (TCI-240)	287	33.50	6.00	*Information not available for interpretation*
	*Self-reliance* [50, 52, 106]	16F Personality Indicator	69	6.25	0.00	Nurses’ personality trait of self-reliance is higher than the general population.
		Cattell’s Sixteen Personality Factor	159	12.07	0.45	*Information not available for interpretation*
		16F Personality Indicator	71	5.73	0.00	Nurses’ personality trait of self-sufficiency is higher than the general population.
	*Self-transcendence* [1, 66, 141]	Temperament and Character Inventory (TCI-R 140)	411	47.02	0.00	Nurses exhibit average self-transcendence, when compared to population norms.
		Temperament and Character Inventory (TCI-240)	287	17.90	5.40	*Information not available for interpretation*
	*Sense of well-being* [57]	California Psychological Inventory	36	38.36	3.22	*Information not available for interpretation*
	*Sensing/Intuition* [50]	Myers-Briggs Type Indicator	71	92.09	11.79	Nurses’ exhibit more sensing than intuition.
	*Sensitivity* [50, 52, 106]	16F Personality Indicator	140	6.22	0.45	Nurses’ personality trait of sensitivity is higher than the general population.
		Cattell’s Sixteen Personality Factor	159	11.02	0.00	*Information not available for interpretation*
	*Shrewdness* [106]	Cattell’s Sixteen Personality Factor	159	9.02	1.08	*Information not available for interpretation*
	*Sociability* [57]	California Psychological Inventory	36	27.58	3.97	*Information not available for interpretation*
	*Social boldness* [106]	Cattell’s Sixteen Personality Factor	159	11.01	0.49	*Information not available for interpretation*
	*Social pressure* [57] *B* [65]	California Psychological Inventory	70	29.71	8.94	*Information not available for interpretation*
	*Socialisation* [57]	California Psychological Inventory	36	37.75	4.86	*Information not available for interpretation*
	*Sociotrophy* [76, 117]	Sociotropy– autonomy Scale	392	66.12	7.01	Nurses scored 66.12out of 120, where higher score = higher sociotrophy.
	*Stability* [52]	16F Personality Indicator	69	4.94	0.19	Nurses’ personality trait of stability is lower than the general population.
	*Succourance* [138]	Edwards Personal Preference Schedule (EPPS)	445	13.20	4.19	*Information not available for interpretation*
	*Suspiciousness* [50]	16F Personality Indicator	71	5.80	0.00	Nurses’ personality trait of Suspiciousness is higher than the general population.
	*Tension* [50, 52, 106]	16F Personality Indicator	140	6.20	0.17	Nurses’ personality trait of tension is higher than the general population.
		Cattell’s Sixteen Personality Factor	159	10.31	0.00	*Information not available for interpretation*
	*Thinking/Feeling* [50]	Myers-Briggs Type Indicator	71	117.45	0.00	Nurses exhibit higher feelings preference than thinking.
	*Tolerance* [57, 65]	California Psychological Inventory	70	22.67	4.45	*Information not available for interpretation*
	*Tough-mindedness* [52]	16F Personality Indicator	69	6.36	0.17	Nurses’ personality trait of tough-mindedness is higher than the general population.
	*Trait Anger* [76]	Trait Anger–Anger Expression Scales	370	20.58	4.46	Nurses scored 20.58 out of 40, where higher scores = higher levels of anger.
	*Venturesomeness* [50]	16F Personality Indicator	71	5.61	0.21	Nurses’ personality trait of venturesomeness is higher than the general population.
	*Vigilance* [52, 106]	16F Personality Indicator	69	5.55	0.00	Nurses personality trait of vigilance is lower than the general population.
		Cattell’s Sixteen Personality Factor	159	9.31	1.33	*Information not available for interpretation*
	*Warmth* [50, 106]	16F Personality Indicator	140	5.45	0.57	Nurses’ personality trait of warmth is slightly lower than the general population.
		Cattell’s Sixteen Personality Factor	159	10.07	0.00	*Information not available for interpretation*
	*Well-being* [65]	California Psychological Inventory	34	31.02	1.90	*Information not available for interpretation*
	*Work orientation* [65]	California Psychological Inventory	34	28.01	0.51	*Information not available for interpretation*
		Type D Scale-14	450	1.29	0.45	Higher levels of Type D, which is a risk factor for negative psychophysical health
		Core Self-Evaluations Scale *mean*	142	3.50	0.52	Nurses scored 3.50 out of 5, where higher scores = higher association with personality trait.
		Core Self-Evaluations Scale *sum*	584	40.50	5.30	*Information not available for interpretation*
	*Proactive* [85]	Proactive Personality Scale	464	5.02	0.78	*Information not available for interpretation*
	*Global Score* [68, 120, 215]	Core Self-Evaluations Scale (CSES) - sum	1131	42.26	6.79	Nurses score 42.26 out of 65, where higher scores indicated higher association to personality traits.
**Allied Health**	*Cooperativeness* [187, 188]	Temperament and Character Inventory	1123	83.38	0.00	Allied Health clinicians exhibit very high cooperativeness, when compared to population norms.
	*Harm avoidance* [187, 188]	Temperament and Character Inventory	1123	54.20	0.00	Allied Health clinicians exhibit average harm avoidance, when compared to population norms.
	*Novelty Seeking* [187, 188]	Temperament and Character Inventory	1123	55.53	0.00	Allied Health clinicians exhibit high novelty seeking, when compared to population norms.
	*Persistence* [187, 188]	Temperament and Character Inventory	1123	72.38	0.00	Allied Health clinicians exhibit very high persistence, when compared to population norms.
	*Reward Dependence* [187, 188]	Temperament and Character Inventory	1123	71.87	0.00	Allied Health clinicians exhibit very high reward dependence, when compared to population norms.
	*Self-directedness* [187, 188]	Temperament and Character Inventory	1123	77.39	0.00	Allied Health clinicians exhibit very high self-directedness, when compared to population norms.
	*Self-transcendence* [187, 188]	Temperament and Character Inventory	1123	44.98	0.00	Allied Health clinicians exhibit low self-transcendence, when compared to population norms.
**Dentistry**	*Agreeableness* [201]	NEO Five-Factor Inventory (NEO FFI) 5 point scale	1298	42.10	5.90	Dentists score 42.10 out of 60, where higher score = higher association to personality trait.
	*Ambition* [203]	Personality Styles and Disorder Inventory (PSDI)	580	45.47	8.27	Scores differ to population norms.
	*Assertiveness* [203]	Personality Styles and Disorder Inventory (PSDI)	580	49.79	9.48	Scores similar to population norms.
	*Charming* [203]	Personality Styles and Disorder Inventory (PSDI)	580	49.79	9.48	Scores similar to population norms.
	*Conscientiousness* [201, 203]	NEO Five-Factor Inventory (NEO FFI) 5 point scale	1298	45.90	6.00	Dentists score 45.90 out of 60, where higher score = higher association to personality trait.
		Personality Styles and Disorder Inventory (PSDI)	580	58.84	7.76	Scores similar to population norms.
	*Critical* [203]	Personality Styles and Disorder Inventory (PSDI)	580	49.05	8.61	Scores similar to population norms.
	*Extraversion* [201, 202]	Eysenck Personality Questionnaire	77	12.22		*Information not available for interpretation*
		NEO Five-Factor Inventory (NEO FFI) 5 point scale	1298	40.20	7.30	Dentists score 40.20 out of 60, where higher score = higher association to personality trait.
	*Extrinsic importance* [197]	The Aspiration Index	594	3.27	1.14	Dentists scored 3.27 out of 7, where higher scores = higher association.
	*Extrinsic likelihood* [197]	The Aspiration Index	594	3.13	1.01	Dentists scored 3.13 out of 7, where higher scores = higher association.
	*Intrinsic importance* [197]	The Aspiration Index	594	5.74	0.78	Dentists scored5.74 out of 7, where higher scores = higher association.
	*Intrinsic likelihood* [197]	The Aspiration Index	594	4.90	0.96	Dentist scored 4.90 out of 7, where higher score = higher association.
	Intuition [203]	Personality Styles and Disorder Inventory (PSDI)	580	53.09	10.54	Scores differ to population norms.
	Loyal [203]	Personality Styles and Disorder Inventory (PSDI)	580	47.51	8.77	Scores differ to population norms.
	Neuroticism [201, 202]	Eysenck Personality Questionnaire	77	7.82		*Information not available for interpretation*
		NEO Five-Factor Inventory (NEO FFI) 5 point scale	1298	33.80	9.20	Dentists score 33.80 out of 60, where higher score = higher association to personality trait.
	Openness [201]	NEO Five-Factor Inventory (NEO FFI) 5 point scale	1298	40.10	7.30	Dentists score 42.10 out of 60, where higher score = higher association to personality trait.
	Optimism [203]	Personality Styles and Disorder Inventory (PSDI)	580	53.60	9.08	Scores differ to population norms.
	Passive [203]	Personality Styles and Disorder Inventory (PSDI)	580	48.85	8.25	Scores differ to population norms.
	Reserved [203]	Personality Styles and Disorder Inventory (PSDI)	580	45.47	10.50	Scores differ to population norms.
	Self-critical [203]	Personality Styles and Disorder Inventory (PSDI)	580	50.23	9.33	Scores similar to population norms.
	Spontaneous [203]	Personality Styles and Disorder Inventory (PSDI)	580	46.44	7.31	Scores differ to population norms.
	Unselfish [203]	Personality Styles and Disorder Inventory (PSDI)	580	52.39	10.23	Scores differ to population norms.
	Wilful [203]	Personality Styles and Disorder Inventory (PSDI)	580	44.72	9.79	Scores differ to population norms.
**Dietetics**	*Cooperativeness* [10, 28]	Temperament and Character Inventory	667	80.79	2.01	Dietitians exhibit high cooperativeness, when compared to population norms.
	*Harm avoidance* [10, 28]	Temperament and Character Inventory	667	56.85	9.78	Dietitians exhibit high harm avoidance, when compared to population norms.
	*Novelty Seeking* [10, 28]	Temperament and Character Inventory	667	54.11	2.47	Dietitians exhibit average novelty seeking, when compared to population norms.
	*Persistence* [10, 28]	Temperament and Character Inventory	667	73.53	5.03	Dietitians exhibit high persistence, when compared to population norms.
	*Reward Dependence* [10, 28]	Temperament and Character Inventory	667	70.61	2.62	Dietitians exhibit high reward dependence, when compared to population norms.
	*Self-directedness* [10, 28]	Temperament and Character Inventory	667	74.30	6.96	Dietitians exhibit high self-directedness, when compared to population norms.
	*Self-transcendence* [10, 28]	Temperament and Character Inventory	667	42.37	3.43	Dietitians exhibit low self-transcendence, when compared to population norms.
**Nursing Assistants**	*Adjustment* [209]	Hogan Personality Inventory	177	22.66	6.97	Nursing assistants scored, 22.66 out of 37, where higher score = higher association.
	*Ambition* [209]	Hogan Personality Inventory	177	20.58	5.28	Nursing assistants scored, 20.58 out of 29, where higher score = higher association.
	*Intellectance* [209]	Hogan Personality Inventory	177	11.96	4.52	Nursing assistants scored, 11.96 out of 25, where higher score = higher association.
	*Likeability* [209]	Hogan Personality Inventory	177	18.63	3.07	Nursing assistants scored, 18.63 out of 22, where higher score = higher association.
	*Prudence* [209]	Hogan Personality Inventory	177	19.94	4.83	Nursing assistants scored, 19.94 out of 31, where higher score = higher association.
	*School success* [209]	Hogan Personality Inventory	177	7.77	3.14	Nursing assistants scored, 7.77 out of 14, where higher score = higher association.
	*Sociability* [209]	Hogan Personality Inventory	177	11.33	4.76	Nursing assistants scored, 11.33 out of 24, where higher score = higher association.
**Paramedical**	*Agreeableness* [207, 208]	NEO-Five Factor Inventory-Short Form	252	26.60	4.70	*Information not available for interpretation*
		Big Five Inventory (BFI) *Subscale Sum Score*	395	33.47	4.40	Paramedics scored 33.47 out 45, where higher scores = higher association with personality trait.
	*Conscientiousness* [207, 208]	NEO-Five Factor Inventory-Short Form	252	29.10	5.20	*Information not available for interpretation*
		Big Five Inventory (BFI) *Subscale Sum Score*	395	34.77	4.67	Paramedics scored 34.77 out 45, where higher scores = higher association with personality trait.
	*Depressivity* [206]	Freiburg Personality Inventory	97	4.86	0.00	Paramedics exhibit intermediate depressivity.
	*Emotional liability* [206]	Freiburg Personality Inventory	97	5.43	0.00	Paramedics exhibit intermediate emotional liability.
	*Extroversion/Introversion* [206]	Freiburg Personality Inventory	97	6.44	0.70	Paramedics exhibit intermediate association to extroversion-introversion.
	*Extroversion* [207, 208]	NEO-Five Factor Inventory-Short Form (NEO-FFI-SF)	252	26.50	5.00	*Information not available for interpretation*
		Big Five Inventory (BFI) *Subscale Sum Score*	395	29.06	5.20	Paramedics scored 29.06 out 40, where higher scores = higher association with personality trait.
	*Irritability* [206]	Freiburg Personality Inventory	97	5.73	0.23	Paramedics exhibit intermediate irritability.
	*Masculinity/femininity* [206]	Freiburg Personality Inventory	97	6.60	0.56	Paramedics exhibit intermediate association to masculity-feminity.
	*Mental balance* [206]	Freiburg Personality Inventory	97	6.09	0.73	Paramedics exhibit intermediate mental balance.
	*Neuroticism* [206–208]	Freiburg Personality Inventory	97	5.59	0.00	Paramedics exhibit intermediate neuroticism.
		Big Five Inventory (BFI) *Subscale Sum Score*	395	21.87	3.11	Paramedics scored 21.87 out 45, where higher scores = higher association with personality trait.
		NEO-Five Factor Inventory-Short Form	252	20.60	5.10	*Information not available for interpretation*
	*Openness* [206–208]	Freiburg Personality Inventory	97	6.09	0.00	Paramedics exhibit intermediate openness.
		Big Five Inventory (BFI) *Subscale Sum Score*	395	34.65	5.51	Paramedics scored 34.65 out 50, where higher scores = higher association with personality trait.
		NEO-Five Factor Inventory-Short Form	252	22.70	3.70	*Information not available for interpretation*
	*Reactive aggressivity* [206]	Freiburg Personality Inventory	97	5.97	0.00	Paramedics exhibit intermediate reactive aggressivity.
	*Shyness* [206]	Freiburg Personality Inventory	97	6.37	0.00	Paramedics exhibit intermediate shyness.
	*Sociability* [206]	Freiburg Personality Inventory	97	4.11	0.00	Paramedics exhibit intermediate sociability.
	*Spontaneous aggressivity* [206]	Freiburg Personality Inventory	97	4.84	0.63	Paramedics exhibit intermediate spontaneous aggressivity.
**Pharmacy**	*Agreeableness* [192]	Big Five Inventory	23	4.21	0.46	Doctors scored 4.21 out of 5, where higher scores = higher association with personality trait.
	*Ascendency* [189]	Gordon Personal Profile Index	275	20.99	5.90	Pharmacists score 20.99 out of 36, where higher score = higher association.
	*Cautiousness* [189]	Gordon Personal Profile Index	275	26.46	5.40	Pharmacists scored 26.46 out of 40, where higher score = higher association.
	*Conscientiousness* [192]	Big Five Inventory	23	4.01	0.71	Pharmacists scored 4.01 out of 5, where higher score = higher association with personality trait.
	*Emotional stability* [189]	Gordon Personal Profile Index	275	22.42	6.30	Pharmacists scored 22.42 out of 36, where higher score = higher association.
	*Extroversion* [192]	Big Five Inventory	23	3.56	0.92	Pharmacists scored 3.56 out of 5, where higher score = higher association with personality trait.
	*Neuroticism* [192]	Big Five Inventory	23	2.51	0.76	Pharmacists scored 2.51 out of 5, where higher score = higher association with personality trait.
	*Openness* [192]	Big Five Inventory	23	3.67	0.65	Pharmacists scored 3.67 out of 5, where higher score = higher association with personality trait.
	*Original thinking* [189]	Gordon Personal Profile Index	275	24.87	5.40	Pharmacists scored 24.87 out of 40, where higher score = higher association.
	*Personal relations* [189]	Gordon Personal Profile Index	275	22.55	5.70	Pharmacists scored 22.55 out of 40, where higher score = higher association.
	*Responsibility* [189]	Gordon Personal Profile Index	275	27.43	4.50	Pharmacists scored 27.43 out of 36, where higher score = higher association.
	*Self-esteem* [189]	Gordon Personal Profile Index	275	91.94	15.20	Pharmacists scored 91.94 out of 180, where higher score = higher association.
	*Sociability* [189]	Gordon Personal Profile Index	275	21.09	6.30	Pharmacists scored 21.90 out of 36, where higher score = higher association.
	*Vigour* [189]	Gordon Personal Profile Index	275	26.90	5.80	Pharmacists scored 26.90 out of 40, where higher score = higher association.
**Physiotherapy**	*Agreeableness* [186, 193]	Big Five Inventory	85	3.75	0.03	Physiotherapists scored 3.75 out of 5, where higher score = higher association with personality trait.
	*Conscientiousness* [186, 193]	Big Five Inventory	85	3.69	0.00	Physiotherapists scored 3.69 out of 5, where higher score = higher association with personality trait.
	*Extroversion* [186, 193]	Big Five Inventory	85	3.49	0.00	Physiotherapists scored 3.49 out of 5, where higher score = higher association with personality trait.
	*Neuroticism* [186, 193]	Big Five Inventory	85	2.38	0.00	Physiotherapists scored 2.38 out of 5, where higher score = higher association with personality trait.
	*Openness* [186, 193]	Big Five Inventory	85	3.42	0.00	Physiotherapists scored 3.42 out of 5, where higher score = higher association with personality trait.
**Pathologist**	*Agreeableness* [210]	Big Five Inventory (BFI)	37	3.97	1.09	Pathologists scored 3.97 out of 5, where higher score = higher association with personality trait.
	*Conscientiousness* [210]	Big Five Inventory (BFI)	37	3.88	0.65	Pathologists scored 3.88 out of 5, where higher score = higher association with personality trait.
	*Extroversion* [210]	Big Five Inventory (BFI)	37	3.98	0.73	Pathologists scored 3.98 out of 5, where higher score = higher association with personality trait.
	*Neuroticism* [210]	Big Five Inventory (BFI)	37	2.38	0.76	Pathologists scored 2.38 out of 5, where higher score = higher association with personality trait.
	*Openness* [210]	Big Five Inventory (BFI)	37	3.77	0.59	Pathologists scored 3.77 out of 5, where higher score = higher association with personality trait.
**Radiologist**	*Global Score* [196]	The Personal Views Survey II	95	89.90	11.90	Radiologist scores 89.9 out of 150, where higher score = higher hardiness
**Social Workers**	*Neuroticism* [178]	Mini-International Personality Item Pool (Mini-IPIP)	8	11.55	2.16	Social workers demonstrated a lower level of neuroticism
**Unidentified Health Professionals**	*Extroversion* [204]	Big Five Inventory (BFI) Subscale Sum Score	65	31.12	5.07	Health Professionals scored 31.12 out 40, where higher scores = higher association with personality trait.
	*Agreeableness* [204]	Big Five Inventory (BFI) Subscale Sum Score	65	37.42	5.46	Health Professionals scored 37.42 out 45, where higher scores = higher association with personality trait.
	*Conscientiousness* [204]	Big Five Inventory (BFI) Subscale Sum Score	65	37.10	5.48	Health Professionals scored 37.10 out 45, where higher scores = higher association with personality trait.
	*Neuroticism* [204]	Big Five Inventory (BFI) Subscale Sum Score	65	19.30	6.35	Health Professionals scored 19.30 out 45, where higher scores = higher association with personality trait.

#### Nursing

Sixty-one tools were utilised to investigate nursing personality across the 98 included studies (*n* = 31,971). Synthesised results demonstrate that nurses exhibited high levels of agreeableness [51, 53, 55, 61, 64, 71, 75, 77, 78, 82, 100, 102, 105], assertiveness [50], dominance [52, 106], conscientiousness [53, 55, 61, 64, 75, 78, 82, 100, 105] and hardiness [53, 63, 83, 92, 97, 98, 101]; Nurses were shown to have lower levels of abstractedness [52, 106], apprehension [52, 106], boldness [52], imagination [50], independence [50, 52] and neuroticism [51, 62, 70, 71, 79, 89, 100]. The literature is less clear on traits of openness, with some studies reporting high levels [51–53, 55, 60, 61, 64, 77, 100, 216] whilst other studies reported lower levels [71]. The same was evident for extroversion-introversion: a greater number of studies identified high levels of extroversion in nurses [50–52, 71, 74, 77, 104, 137], however there were also multiple studies identifying nurses to be more introverted [50, 56, 69, 84, 93, 111, 124] in nature. Nurses were identified to have higher levels of sensing and judging personality traits [73, 87, 94, 111, 124, 137]. Finally, Huang, Cai [217] examined nurses’ personality relative to psychological distress and identified that nurses with mod-severe psychological distress demonstrate significantly more negative personality traits (53%), compared to those who have none-mild distress demonstrating 97% positive traits.

#### Nursing assistants

Nursing assistants were investigated in one study (*n* = 177) [209], with results indicating that nurse assistants’ exhibit personality traits with high levels of likeability (18.63 ± 3.07) and ambition (20.58 ± 5.28), and low levels of sociability (11.33 ± 4.76) and intellectance (openness to new experiences) (11.96 ± 4.52).

#### Medicine

The 52 studies investigating medical professionals (*n* = 21,125), used 33 different outcome tools (some with multiple versions). Results demonstrated doctors have high personality traits of dominance [154], instrumentality [211], perfectionism [154], reasoning [154], reward-dependence [1, 148, 149, 151], sensitivity [154], shrewdness [154], anxiety [179], agreeableness [181], openness [181] and tension [154]. Medical professionals were shown to have low levels of narcissism [58], abstractedness [154], neuroticism [90, 100, 147, 152, 153, 157, 181, 218], psychoticism [58, 90, 153] and social boldness [154]. The literature was unclear in terms of extroversion-introversion with some studies indicating higher levels of introversion [94, 145, 152, 153, 164], where others indicated higher levels of extroversion [150, 155, 218], and one had a balanced representation of both introversion and extroversion [183]. The same was evident for the personality trait of openness with Reeve [154] indicating lower levels, and van Mol, Nijkamp [100] finding higher levels, whilst the remaining studies investigating openness recorded average levels [150, 156, 157, 218].

#### Dentistry

Of the seven studies investigating dentists (*n* = 3664), three studies qualitatively reported on personality traits utilising the Myers-Briggs Type Indicator (MBTI) [198–200], and one study investigated aspiration utilising The Aspiration Index [197]. Dentists most commonly presented with ISTJ (i.e. introversion-sensing-thinking-judging; 16.0–54%) and ESTJ (extroversion-sensing-thinking-judging; 13.0–14.3%), with higher levels of judging and perceiving [198, 199]. Aspiration was primarily driven by intrinsic importance [197].

#### Allied health

Campbell, Eley [187] and Campbell, Eley [188] investigated allied health personality as a collective professional group utilising the Temperament and Character Inventory and found very high personality traits of cooperativeness (83.38 ± 0.00), self-directedness (77.39 ± 0.00), persistence (72.38 ± 0.00), reward dependence (71.87 ± 0.00), and low self-transcendence (44.98 ± 0.00). These results (*n* = 1123) indicated that allied health clinicians are highly self-motivated, work well in a team, but are less spiritual in nature.

#### Dietetics

Three studies investigated dietitians (*n* = 776) [10, 28, 190]. Two studies utilised the Temperament and Character Inventory [10, 28], with results identifying high cooperativeness (80.79 ± 2.01), persistence (73.53 ± 5.03), self-directedness (74.30 ± 6.96), harm avoidance (56.85 ± 9.78) and novelty seeking (54.11 ± 2.47); with low self-transcendence (42.37 ± 3.43) [10, 28]. Hagan and Taylor [190] utilised the Myers-Briggs Type Indicator, and demonstrated ESFJ (i.e., extroversion-sensing-feeling-judging; 16.7%) was the most common combination of personality traits, with sensing and judging being the most prevalent across all personality combinations for dietitians.

#### Occupational therapy

Two studies explored occupational therapists’ personality [191, 195]. Lysack, McNevin [191] (*n* = 128) utilised the Kiersey-Bates Personality Inventory, and found occupational therapists exhibited sensing-perceiving (SP) (49%) and intuitive-feeling (NF) (27%) traits, suggesting they are resourceful, and sensitive to the needs of people. Radonsky [195] assessed against the Myers-Briggs Type Indicator (MBTI) with results demonstrating ISF (i.e., introversion-sensing-feeling-judging, 69%) was the most common personality trait.

#### Physiotherapy

Four studies (*n* = 495) investigated physiotherapists’ personality traits [186, 191, 193, 194]. Buining, Kooijman [186] and Kooijman, Buining [193] utilised the Big Five Inventory identifying highest in rank association with agreeableness (3.75 ± 0.03), conscientiousness (3.69 ± 0.00), extroversion (3.49 ± 0.00), openness (3.42 ± 0.00) and neuroticism (2.38 ± 0.00). Lysack, McNevin [191] identified that physiotherapists exhibited a sensing-judging (SJ) (66%) temperament. These results suggest that physiotherapists are generally calm, relaxed, secure, stable and resilient clinicians. Finally, Martinussen, Borgen [194] examined Type A personality with the Revised Jenkins Activity survey and established higher levels of Achievement strivings (3.5 ± 0.44) compared to impatience-irritability (2.4 ± 0.57).

#### Pharmacy

Pharmacists were investigated in two studies (*n* = 298). One study utilised the Big Five Inventory [192] across traits of agreeableness, conscientiousness, extroversion, neuroticism, and openness. The other study investigated ascendence, cautiousness, emotional stability, original thinking, personal relations, responsibility, self-esteem, sociability, and vigour utilising the Gordon Personality Profile Index [189]. Results indicated that pharmacists exhibit average or above scores across all items. The highest scoring traits were agreeableness, extroversion, openness, and responsibility; suggesting pharmacists are cooperative, outgoing and responsible.

#### Paramedics

Three studies investigated paramedics’ personality traits [207, 208, 219]. Paramedics were found to have highest scores in conscientiousness and lowest scores in neuroticism across both the Big Five Inventory (*n* = 395) [207] and NEO-Five Factor Inventory-Short Form (*n* = 252) [208]. Bergmueller, Zavgorodnii [206] investigated paramedics’ personality traits (*n* = 97) utilising the Freiburg Personality Inventory. Results indicated that paramedics exhibit intermediate association across all 12 attributes of emotional liability, extroversion-introversion, irritability, masculinity-femininity, mental balance, neuroticism, openness, reactive aggressivity, spontaneous aggressivity, shyness, and sociability. Despite all attributes of personality being intermediate levels, for those aged < 35 years, paramedics demonstrated higher mental balance, extraversion-introversion, and masculinity-femininity than those aged ≥45 years. In addition, those aged ≥45 years demonstrated lower levels of spontaneous aggression. These results suggest that paramedics have a balanced personality, though have slight variation relative to their age.

#### Pathologists

Iorga, Soponaru [210] was the only study to investigate pathologist personality utilising the Big Five Inventory, with highest scores in extraversion (3.98 ± 0.73) and agreeableness (3.97 ± 1.09).

#### Radiologists

Sciacchitano, Goldstein [196] was the only study to investigate radiologist personality utilising The Personal Views Survey II. Results indicated that Radiologists have a high level of hardiness (89.9 ± 11.9), suggesting they have a higher level of resilience.

#### Social workers

Social worker personality trait of neuroticism was assessed in one study (*n* = 8) [178], identifying they exhibited lower levels of neuroticism (11.55 ± 2.16) compared to their medical colleagues.

Table 2 provides detailed information about the personality traits of health professionals.

### Behaviour styles

Of the 321 studies, ten studies investigated behaviour (*n* = 6709); five of these were with nurses [99, 220–223], three studies were in medicine [25, 175, 224], one study was with occupational therapists [225], and one study in psychologists [226].

#### Nursing

Four studies (*n* = 2068) were included in the meta-aggregation [99, 220–222] (Table 3), and one study reported narratively (*n* = 3396) [223]. Two studies [99, 220] demonstrated moderate association to Type B behaviours (relaxed, patient and friendly); whilst two other studies demonstrated high association with Type A behaviours (ambitious, organised, impatient, punctual and irritable). Keogh, Robinson [223] described nurses to have high dominance (39%) and conscientiousness (35%) behaviours. Table 3 provides detailed information about the behaviour styles of health professionals.

**Table 3:** Meta-aggregated results for behaviour styles of health professionals (*n* = 7 studies)

Profession	Behaviour Style	Tool	N	Mean	SD	Practical Interpretation
**Nurse**	*Type A / Type B Behaviour* [99, 220–222]	Bortner Measure of Type A behaviour	925	92.29	4.15	Nurses demonstrate moderate association with Type B behaviour.
		Type A Behaviour Scale	1043	44.18	6.09	Nurses demonstrate association with Type A behaviour.
		Type A Behavioural Pattern Scale	100	60.97	9.23	Nurses demonstrate association with Type A behaviour.
		Type B Behavioural Pattern Scale	100	37.6	6.58	Nurses demonstrate low association with Type B behaviour.
**Medicine**	*Dominance* [25]	DiSC	46	2.90	0.14	Doctors have a lower level of Dominance
	*Influence* [25]	DiSC	46	3.63	0.00	Doctors have a lower level of Influence
	*Steadiness* [25]	DiSC	46	4.93	0.00	Doctors have a higher prevalence of Steadiness
	*Conscientiousness* [25]	DiSC	46	5.14	0.16	Doctors have a higher prevalence of Conscientiousness
	*Type A Behaviour* [224]	Modified Bortner Measure of Type A behaviour	41	86.44	–	Doctors demonstrate slightly more association with Type A behaviours.
**Psychologists**	*Type A Behaviour* [226]	Jenkins Activity Survey	118	10.3	3.4	Psychologists demonstrate slightly higher Type A behaviours.

#### Medicine

Three studies (*n* = 742) in medicine investigated behaviour utilising different tools. Marcisz-Dyla, Dąbek [175] investigated Type A behaviour utilising the Framingham Type A Scale. Results concluded that doctors equal had Type A (33.1%), Type B (33.8%) and intermediate (33.1%) behaviours [175]. Ogunyemi, Mahller [25] demonstrated that doctors have a higher prevalence of Conscientiousness (5.14 ± 0.16) and Steadiness (4.93 ± 0.00), compared to Dominance (2.90 ± 0.14) and Influence (3.63 ± 0.00) utilising the DiSC assessment tool [25] (Table 3) [224]. study demonstrated doctors have slightly more association with Type A behaviours (mean score of 86.44, where 84 is neutral).

#### Occupational therapy

Bailey [225] investigated behaviour traits exhibited by occupational therapists using the Rokeach Values Survey. Results concluded that lovingness, mature love and inner harmony are associated with occupational therapy clinicians; whilst capability and sense of accomplishment behaviours are more likely associated with occupational therapy administrators [225].

#### Psychology

Matthews, Heimreich [226] studied Type A behaviours in psychologists, with results demonstrating slightly higher Type A behaviours (10.3 ± 3.4).

### Emotional intelligence

One hundred and forty five studies investigated emotional intelligence (*n* = 42,795) Nurses were investigated in 105 studies [20, 79, 80, 82, 91, 102, 213, 216, 227–314], 33 studies explored EI with medical doctors [239, 253, 257, 265, 315–342], three were in dentistry (*n* = 661) [343–345], three studied EI in occupational therapists (*n* = 1369) [346–348], two in physiotherapists (*n* = 189) [349, 350], one in radiologists (*n* = 22) [351] and six collectively explored health professionals [31, 342, 352–355]. Meta-aggregation of 142 of the studies is provided in Table 4; the remaining three studies [227, 236, 351] were synthesised narratively.

**Table 4:** Meta-aggregated results of emotional intelligence (*n* = 142 studies)

Profession	Emotional Intelligence Characteristic	Tool	N	Mean	SD	Practical Interpretation
**Health Professional**	*Emotional Intelligence (total)* [352, 353]	Bar-On’s Emotional Quotient Inventory - 133 item	27	99.00	9.60	Health professionals exhibit average levels of emotional intelligence
		Multidimensional Measure of Emotional Intelligence	600	255.48	2.69	*Information not available for interpretation*
	*Adaptability* [352, 354]	Bar-On’s Emotional Quotient Inventory - 133 item	27	97.10	9.10	Health professionals as a whole exhibit average adaptability abilities.
		Brief Emotional Intelligence Inventory for Senior Citizens (EQ-i-20 M)	386	11.20	2.05	*Information not available for interpretation*
	*General Mood* [352, 354]	Bar-On’s Emotional Quotient Inventory - 133 item	27	100.60	9.20	Health professionals as a whole exhibit average general mood abilities.
		Brief Emotional Intelligence Inventory for Senior Citizens (EQ-i-20 M)	386	12.35	2.27	*Information not available for interpretation*
	*Handling relationships* [31]	Multidimensional Measure of Emotional Intelligence	600	50.69	1.29	*Information not available for interpretation*
	*Interpersonal* [352, 354]	Bar-On’s Emotional Quotient Inventory - 133 item	27	102.60	9.20	Health professionals as a whole exhibit average interpersonal abilities.
		Brief Emotional Intelligence Inventory for Senior Citizens (EQ-i-20 M)	386	11.87	2.02	*Information not available for interpretation*
	*Intrapersonal* [352, 354]	Bar-On’s Emotional Quotient Inventory - 133 item	27	98.10	11.50	Health professionals as a whole exhibit average intrapersonal abilities.
		Brief Emotional Intelligence Inventory for Senior Citizens (EQ-i-20 M)	386	10.39	2.73	*Information not available for interpretation*
	*Managing emotions* [31]	Multidimensional Measure of Emotional Intelligence	600	51.69	0.97	*Information not available for interpretation*
	*Motivating oneself* [31]	Multidimensional Measure of Emotional Intelligence	600	51.97	0.87	*Information not available for interpretation*
	*Self-awareness* [31]	Multidimensional Measure of Emotional Intelligence	600	50.22	1.55	*Information not available for interpretation*
	*Stress Management* [352]	Bar-On’s Emotional Quotient Inventory - 133 item	27	100.40	12.00	Health professionals as a whole exhibit average stress management.
		Brief Emotional Intelligence Inventory for Senior Citizens (EQ-i-20 M)	386	6.75	1.77	*Information not available for interpretation*
	*self emotional appraisal* [355]	Wong and Law Emotional Intelligence Scale (WLEIS) – 5-point scale	260	15.97	2.55	*Information not available for interpretation*
	*others emotional appraisal* [355]	Wong and Law Emotional Intelligence Scale (WLEIS) – 5-point scale	260	13.91	2.65	*Information not available for interpretation*
	*use of emotion* [355]	Wong and Law Emotional Intelligence Scale (WLEIS) – 5-point scale	260	15.41	2.21	*Information not available for interpretation*
	regulation of emotion [355]	Wong and Law Emotional Intelligence Scale (WLEIS) - 5-point scale	260	15.31	2.36	Information not available for interpretation
**Medicine**	*Emotional Intelligence* [214, 253, 265, 316–322, 325–328, 330, 332, 334, 336, 337, 339, 341, 356]	Bar-On’s Emotional Quotient Inventory - 133 item sum	30	495.46	35.75	*Information not available for interpretation*
		Bar-On’s Emotional Quotient Inventory (EQ-I) - 133 item	54	102.06	2.63	*Information not available for interpretation*
		Bar-On’s Emotional Quotient Inventory (EQ-I) - 90 item	245	330.24	38.50	*Information not available for interpretation*
		Bar-On’s Emotional Quotient Inventory 2.0	66	106.75	3.41	Doctors exhibit medium emotional intelligence
		Mayer-Salovey-Caruso Emotional Intelligence Test	26	101.89	15.44	Doctors exhibit average emotional intelligence
		Schuttes Emotional Intelligence Test	50	145.44	13.29	Doctors scored 145.44 out of 205, where higher scores = higher EI.
		Self Emotional Intelligence Scale	658	3.53	0.33	Doctors exhibit a medium level of emotional intelligence
		Trait Emotional Intelligence Questionnaire-Short Form	2450	5.00	0.64	Doctors exhibit higher than normal emotional intelligence
		Trait Emotional Intelligence Questionnaire (TEIQue)	4024	5.25	0.06	*Information not available for interpretation*
		Trait Emotional Intelligence Questionnaire (TEIQue) - sum	139	101.00	8.10	*Information not available for interpretation*
		Wong and Law Emotional Intelligence Scale – 7-point scale	488	4.96	0.40	Doctors scored 4.96 out of 7, where higher scores = higher EI.
		Wong and Law Emotional Intelligence Scale – 7-point scale sum	740	81.29	14.76	Doctors scored 81.29 out of 112, where higher scores = higher EI.
		Hay 360 Emotional Competence Inventory (Hay 360 ECI)	33	64.40	11.60	*Information not available for interpretation*
	*Adaptability* [253, 331, 339]	Bar-On’s Emotional Quotient Inventory - 133 item	60	98.94	0.00	Doctors exhibit average levels of adaptability.
		Emotional and Social Competency Inventory (ESCI)	226	4.00	2.68	*Information not available for interpretation*
	Achievement orientation [331]	Emotional and Social Competency Inventory (ESCI)	226	4.50	2.30	*Information not available for interpretation*
	*Benefiting from emotions* [356]	Schuttes Emotional Intelligence Test	50	21.52	1.92	Doctors exhibit lower levels of benefiting from emotions abilities.
	*Cognitive thought* [239]	Emotional Competence Inventory	23	22.57	0.16	*Information not available for interpretation*
	*Conflict management* [331]	Emotional and Social Competency Inventory (ESCI)	226	3.90	0.60	*Information not available for interpretation*
	*Decision making* [320]	Bar-On’s Emotional Quotient Inventory 2.0	31	109.86	12.44	Doctors exhibit medium decision-making abilities of emotional intelligence.
	*Emotion Expression* [356]	Schuttes Emotional Intelligence Test	50	34.60	5.27	*Information not available for interpretation*
	*Emotional Labour Deep acting* [318]	Emotional Labour	740	12.72	3.12	Doctors exhibit moderate deep acting strategies of emotional labour.
	*Emotional Labour Natural acting* [318]	Emotional Labour	740	10.60	2.33	Doctors exhibit high natural acting strategies of emotional labour.
	*Emotional Labour Surface acting* [318]	Emotional Labour	740	19.40	5.91	Doctors exhibit moderate surface acting strategies of emotional labour.
	*Emotionality* [316, 319, 325, 327, 336]	Trait Emotional Intelligence Questionnaire-Short Form	4024	4.91	0.65	Doctors exhibit normal levels of emotionality than population norms.
		Trait Emotional Intelligence Questionnaire (TEIQue)	3993	5.38	0.18	*Information not available for interpretation*
	*General Mood* [253, 320]	Bar-On’s Emotional Quotient Inventory - 133 item	60	83.12	24.39	Doctors exhibit markedly low general mood traits.
	*Interpersonal* [253, 320]	Bar-On’s Emotional Quotient Inventory - 133 item	60	107.08	6.50	Doctors exhibit high interpersonal abilities.
		Bar-On’s Emotional Quotient Inventory 2.0	31	113.65	8.55	Doctors exhibit medium interpersonal abilities.
	*Intrapersonal* [253, 320]	Bar-On’s Emotional Quotient Inventory - 133 item	60	124.61	34.61	Doctors exhibit markedly high intrapersonal abilities.
	*Managing relations* [239]	Emotional Competence Inventory	23	96.52	1.06	*Information not available for interpretation*
	*Optimism/emotion regulation* [356]	Schuttes Emotional Intelligence Test	50	44.10	6.16	*Information not available for interpretation*
	*Others emotional appraisal* [321, 337, 340, 341, 357]	Self Emotional Intelligence Scale	658	3.77	0.49	Doctors scored 3.77 out of 5, where lower score = higher EI.
		Wong and Law Emotional Intelligence Scale – 7-point scale	380	4.90	0.33	Doctors scored 54.90 out of 7, where higher scores = higher EI.
	*Regulation of emotion* [321, 337, 340, 341, 357]	Self Emotional Intelligence Scale	658	3.64	0.48	Doctors scored 3.64 out of 5, where lower score = higher EI.
		Wong and Law Emotional Intelligence Scale – 7-point scale	380	5.27	0.44	Doctors scored 5.27 out of 7, where higher scores = higher EI.
	*Self emotional appraisal* [321, 337, 340, 341, 357]	Self Emotional Intelligence Scale	658	3.83	0.58	Doctors scored 3.83 out of 5, where lower score = higher EI.
		Wong and Law Emotional Intelligence Scale – 7-point scale	380	5.51	0.62	Doctors scored 5.51 out of 7, where higher scores = higher EI.
	*Self-control* [316, 319, 325, 327, 331, 336]	Trait Emotional Intelligence Questionnaire-Short Form	2317	4.77	0.35	Doctors exhibit higher than normal self-control.
		Trait Emotional Intelligence Questionnaire (TEIQue)	3993	5.08	0.06	*Information not available for interpretation*
		Emotional and Social Competency Inventory (ESCI)	226	4.20	3.45	*Information not available for interpretation*
	*Self-awareness* [239, 331]	Emotional Competence Inventory	23	35.32	0.12	*Information not available for interpretation*
		Emotional and Social Competency Inventory (ESCI)	226	4.00	3.07	*Information not available for interpretation*
	*Self-expression* [320]	Bar-On’s Emotional Quotient Inventory 2.0	31	104.22	14.77	Doctors exhibit medium self-expression abilities.
	*Self-perception* [320]	Bar-On’s Emotional Quotient Inventory 2.0	31	107.43	9.33	Doctors exhibit medium levels of self-perception.
	*Self-management* [239]	Emotional Competence Inventory	23	76.44	0.26	*Information not available for interpretation*
	*Sociability* [316, 319, 325, 327, 336]	Trait Emotional Intelligence Questionnaire-Short Form	2377	4.75	0.34	Doctors exhibit normal levels of sociability.
		Trait Emotional Intelligence Questionnaire (TEIQue)	3993	5.11	0.04	*Information not available for interpretation*
	*Social awareness* [239]	Emotional Competence Inventory	23	33.51	0.12	*Information not available for interpretation*
	*Stress Management* [253, 320]	Bar-On’s Emotional Quotient Inventory - 133 item	60	83.93	20.18	Doctors exhibit markedly low stress management.
		Bar-On’s Emotional Quotient Inventory 2.0	31	104.64	10.10	Doctors exhibit medium stress management.
	*Use of emotion* [321, 337, 340, 341, 357]	Self Emotional Intelligence Scale	658	3.40	0.51	Doctors scored 3.4 out of 5, where lower score = higher EI.
		Wong and Law Emotional Intelligence Scale – 7-point scale	380	5.36	0.48	Doctors scored 5.36 out of 7, where higher scores = higher EI.
	*Well-being* [316, 319, 320, 325, 327, 336]	Trait Emotional Intelligence Questionnaire-Short Form	2377	5.52	0.66	Doctors exhibit higher than normal well-being.
		Trait Emotional Intelligence Questionnaire (TEIQue)	3993	5.49	0.13	*Information not available for interpretation*
		Bar-On’s Emotional Quotient Inventory 2.0	31	103.64	14.77	Doctor exhibit medium well-being.
**Nurse**	*Emotional Intelligence (total)* [20, 79, 80, 82, 118, 213, 216, 228–231, 233, 237, 238, 240, 241, 243–256, 258–269, 271, 272, 274, 275, 277, 278, 280, 283–285, 288–293, 295–304, 306–313, 338, 356, 358–364]	Bar-On’s Emotional Quotient Inventory - 125 item	200	434.66	0.00	*Information not available for interpretation*
		Bar-On’s Emotional Quotient Inventory - 133 item	972	106.61	1.62	Nurses exhibit average emotional intelligence.
		Bar-On’s Emotional Quotient Inventory (EQ-I) - 133 item mean	910	5.22	1.28	*Information not available for interpretation*
		Bar-On’s Emotional Quotient Inventory - 133 item sum	172	326.52	99.86	*Information not available for interpretation*
		Bar-On’s Emotional Quotient Inventory - 87 items	277	2.75	0.19	Nurses exhibit medium emotional intelligence.
		Bar-On’s Emotional Quotient Inventory (EQ-I) - 87 items sum	312	332.30	31.30	Nurses score 332.30 out of 435, where higher score = higher emotional intelligence.
		Bar-On’s Emotional Quotient Inventory (EQ-I) - 90 item	513	302.70	42.37	Nurses have moderate levels of emotional intelligence.
		Bar-On’s Emotional Quotient Inventory 2.0	394	106.24	0.56	Nurses exhibit medium emotional intelligence.
		Bradberry & Greaves Emotional Intelligence Questionnaire	388	116.16	7.54	Nurses exhibit excellent emotional intelligence.
		Bradberry & Greaves Emotional Intelligence Questionnaire 100 scale	581	75.71	12.20	Nurses score 75.71 out of 100, where higher scores = higher EI.
		Emotional Intelligence Scale (EIS)	120	124.85	0.00	Nurses demonstrated positive emotional intelligence.
		Emotional Intelligence Scale (EQS)	1150	221.43	77.77	Nurses scored 221.43 out of 252, where higher scores = higher EI.
		GENOS Emotional Intelligence Self-Assessment	254	3.70	0.23	*Information not available for interpretation*
		GENOS EI 31 item	248	3.63	0.49	*Information not available for interpretation*
		Goleman’s Emotional Intelligence Scale	253	78.31	12.88	Nurses scored 78.31 out of 112, where higher scores = higher EI.
		Goleman’s Emotional Intelligence Scale 5-point scale	98	3.64	0.48	Nurses scored 3.64 out of 5, where higher score = higher EI
		Mayer-Salovey-Caruso Emotional Intelligence Test	385	99.65	1.67	Nurses exhibit average emotional intelligence.
		Nursing Emotional Intelligence Scale	41	100.00	15.00	Nurses demonstrate same level of emotional intelligence as general population.
		Schutte Self-Report Emotional Intelligence Test	1623	3.85	0.05	Nurse scored 3.85 out of 5, where higher score = higher EI.
		Schutte Self-Report Emotional Intelligence Test - sum score	2184	125.02	5.95	Nurse scored 125.02 out of 165, where higher score = higher EI.
		Schuttes Emotional Intelligence Test	50	156.56	18.69	Nurse scored 156.56 out of 205 where higher score = higher EI.
		Revised Schutte Emotional Entelligence Scale (RSEIS)	218	152.10	14.80	*Information not available for interpretation*
		Schutte Self-Report Emotional Intelligence Test (SSEIT-33)	173	98.18	9.28	*Information not available for interpretation*
		Self-Rated Emotional Intelligence Scale	101	3.52	0.47	Nurses exhibit slightly lower emotional intelligence.
		Self-Report Emotional Intelligence Test	312	3.73	0.36	*Information not available for interpretation*
		Swinburne University Emotional Intelligence Test	122	213.35	18.04	Nurses scored 213.35 out of 320, where higher scores = higher EI.
		Trait Emotional Intelligence Questionnaire-Short Form	668	5.33	0.19	Nurses exhibit higher than normal emotional intelligence.
		Trait Emotional Intelligence Questionnaire-Short Form - sum score	391	166.40	11.52	*Information not available for interpretation*
		Wong and Law Emotional Intelligence Scale – 5-point scale	1260	3.70	0.22	Nurses score 3.70out of 5, where higher scores = higher EI.
		Wong and Law Emotional Intelligence Scale – 5-point scale (sum)	423	56.82	2.87	Nurses score 56.82 out of 80, where higher scores = higher EI.
		Wong and Law Emotional Intelligence Scale (WLEIS) – 6-point scale	580	5.60	0.78	Nurses score 5.60 out of 6, where higher scores = higher EI.
		Wong and Law Emotional Intelligence Scale – 6-point scale (sum)	528	67.23	8.92	Nurses score 67.23 out of 96, where higher scores = higher EI.
		Wong and Law Emotional Intelligence Scale – 7-point scale	1850	4.97	0.29	Nurses score 4.97 out of 5, where higher scores = higher EI.
		Wong and Law Emotional Intelligence Scale – 7-point scale sum	138	78.64	11.87	Nurses score 78.64 out of 112, where higher scores = higher EI.
		Wong and Law Emotional Intelligence Scale (WLEIS-C) - Chinese version	202	69.81	12.65	*Information not available for interpretation*
		Brief Emotional Intelligence Scale (BEIS-10)	194	2.56	0.39	*Information not available for interpretation*
		Cyberia Shrink 33 items questionnaire	243	3.79	0.48	Nurses scored 3.79 out of 5, where higher score = higher EI.
		Emotional Competence Inventory (ECI) 2.0	283	3.43	0.70	Nurses scored 3.43 out of 5, where higher socre = higher EI.
		Emotional Intelligence Assessment Scale (EIA)	490	142.62	8.10	*Information not available for interpretation*
		Emotional Intelligence Index (EQI)	159	83.30	39.07	*Information not available for interpretation*
		Emotional Intelligence Questionnaire Mean	304	2.08	0.05	*Information not available for interpretation*
		Emotional Intelligence Questionnaire (EIQ)	18	43.95	3.96	*Information not available for interpretation*
		Emotional Intelligence Scale (EIS)	883	122.40	13.50	*Information not available for interpretation*
		Shrink’s Emotional Intelligence Questionnaire	256	91.32		*Information not available for interpretation*
		Siberia Schering’s Emotional Intelligence Standard Questionnaire	500	113.59	14.70	*Information not available for interpretation*
	*Adaptability* [233, 234, 241, 245, 253, 264, 289]	Bar-On’s Emotional Quotient Inventory - 133 item	640	98.55	6.42	Nurses exhibit average adaptability.
		Bar-On’s Emotional Quotient Inventory - 87 items	277	2.81	0.31	Nurses exhibit medium adaptability.
		Bar-On’s Emotional Quotient Inventory (EQ-I) - 87 item sum	312	55.28	6.33	*Information not available for interpretation*
		Reduced Emotional Intelligence Inventory for Adults	2126	2.91	0.53	Nurses score 3.91 out of 4, where higher scores = higher EI.
	*Appraisal of own emotions* [213, 314]	Brief Emotional Intelligence Scale (BEIS-10)	395	1.97	0.00	*Information not available for interpretation*
	*Appraisal of emotions* [292, 361]	Revised Schutte Emotional Intelligence Scale (RSEIS)	218	246.00	4.20	*Information not available for interpretation*
		Schutte Self-Report Emotional Intelligence Test (SSEIT)	156	41.84	1.50	*Information not available for interpretation*
	*Appraisal of other’s emotions* [213, 314]	Brief Emotional Intelligence Scale (BEIS-10)	395	2.73	0.71	*Information not available for interpretation*
	*Altruistic behaviour* [238]	Emotional Intelligence Scale	120	7.14	0.00	Nurses exhibit positive altruistic behaviour abilities.
	*Benefiting from emotions* [356]	Schuttes Emotional Intelligence Test	50	22.64	3.49	*Information not available for interpretation*
	*Cognitive thought* [239]	Emotional Competence Inventory	80	22.77	0.16	*Information not available for interpretation*
	*Commitment* [238]	Emotional Intelligence Scale	120	7.80	0.00	Nurses exhibit positive commitment.
	*Controlling emotions* [229]	GENOS Emotional Intelligence Self-Assessment	194	3.48	0.73	*Information not available for interpretation*
	*Decision making* [20, 229, 237]	Bar-On’s Emotional Quotient Inventory 2.0	166	107.08	0.67	Nurses exhibit medium level decision making
		GENOS Emotional Intelligence Self-Assessment	194	3.51	0.71	*Information not available for interpretation*
	*Emotional expression* [356]	Schuttes Emotional Intelligence Test	50	38.02	5.56	*Information not available for interpretation*
	*Emotional attention* [232, 242, 281, 282, 365, 366]	Trait Meta Mood Scale (TMMS-24)	919	27.58	1.50	Nurses scored 27.57 out of 40 for emotional attention.
		Trait Meta Mood Scale (TMMS-24) – 5-point scale	1100	3.57	0.02	Nurses scored 3.57 out of 5, where higher scores = higher EI.
	*Emotional clarity* [232, 242, 281, 282, 365, 366]	Trait Meta Mood Scale (TMMS-24)	919	30.66	1.62	Nurses scored 30.66 out of 40 for emotional attention.
		Trait Meta Mood Scale (TMMS-24) – 5-point scale	1100	3.82	0.06	Nurses scored 3.82 out of 5, where higher scores = higher EI.
	*Emotional repair* [232, 242, 281, 282, 365, 366]	Trait Meta Mood Scale (TMMS-24)	919	31.45	2.64	Nurses scored 31.45 out of 40 for emotional attention.
		Trait Meta Mood Scale (TMMS-24) – 5-point scale	1100	3.81	0.00	Nurses scored 3.81 out of 5, where higher scores = higher EI.
	*Emotional control* [243, 279]	Swinburne University Emotional Intelligence Test	122	27.01	4.88	*Information not available for interpretation*
		Nursing Manager’s Leadership Behavior Scale	960	43.90	14.30	*Information not available for interpretation*
	*Emotional management* [243]	Swinburne University Emotional Intelligence Test	122	36.85	5.31	*Information not available for interpretation*
	*Emotional recognition & expression* [243, 269]	Swinburne University Emotional Intelligence Test	122	31.76	5.95	*Information not available for interpretation*
		GENOS EI 31 item	248	3.75	0.64	*Information not available for interpretation*
	*Emotions direct cognition* [243]	Swinburne University Emotional Intelligence Test	122	39.43	6.55	*Information not available for interpretation*
	*Understanding emotions* [243]	Swinburne University Emotional Intelligence Test	122	62.79	8.82	*Information not available for interpretation*
	*Emotional stability* [238]	Emotional Intelligence Scale	120	15.29	0.00	Nurses exhibit positive emotional stability.
	*Emotional reasoning* [269]	GENOS EI 31 item	248	3.60	0.66	*Information not available for interpretation*
	*Emotionality* [228, 252, 278, 304]	Trait Emotional Intelligence Questionnaire-Short Form	627	5.32	0.30	Nurses exhibit higher than normal emotionality.
		Trait Emotional Intelligence Questionnaire-Short Form (TEIQue-SF) - sum score	100	45.43	5.40	*Information not available for interpretation*
	*General Mood* [233, 234, 241, 245, 253, 264, 289]	Bar-On’s Emotional Quotient Inventory - 133 item	640	74.15	28.19	Nurses exhibit very low general mood abilities.
		Bar-On’s Emotional Quotient Inventory - 87 items	277	2.64	0.29	Nurses exhibit medium general mood abilities.
		Bar-On’s Emotional Quotient Inventory (EQ-I) - 87 items sum	312	47.14	5.99	*Information not available for interpretation*
		Reduced Emotional Intelligence Inventory for Adults	2126	3.08	0.60	Nurses scored 308 out of 5, where higher score = higher EI.
	*Identifying emotions* [259, 268, 358]	Mayer-Salovey-Caruso Emotional Intelligence Test	260	100.01	0.00	Nurse exhibit average ability to identify emotions.
	*Integrity* [238]	Emotional Intelligence Scale	120	10.49	0.00	Nurses demonstrate positive integrity.
	*Interpersonal* [20, 233, 234, 237, 240, 241, 245, 253, 264, 285, 289]	Bar-On’s Emotional Quotient Inventory - 133 item	640	106.88	3.26	Nurse exhibit average interpersonal EI.
		Bar-On’s Emotional Quotient Inventory - 87 items	277	2.50	0.28	Nurse exhibit low interpersonal EI.
		Bar-On’s Emotional Quotient Inventory (EQ-I) - 87 item sum	312	75.02	6.49	*Information not available for interpretation*
		Bar-On’s Emotional Quotient Inventory 2.0	166	107.84	0.00	Nurse exhibit medium interpersonal EI.
		Emotional Intelligence Scale (EQS)	1150	41.10	2.01	*Information not available for interpretation*
		Reduced Emotional Intelligence Inventory for Adults	2126	3.06	0.50	Nurses score 3.06 out of 5, where higher score = higher EI.
		Bar-On’s Emotional Quotient Inventory - 87 items	277	2.82	0.25	Nurse exhibit medium interpersonal EI.
		Emotional Intelligence Scale	1045	37.50	10.60	*Information not available for interpretation*
		Reduced Emotional Intelligence Inventory for Adults	2126	2.62	0.70	Nurses scored 2.62 out of 4, where higher score = higher EI.
	*Interpersonal relationships* [234, 241, 245, 253, 264]	Bar-On’s Emotional Quotient Inventory - 133 item	640	127.87	24.29	Nurse exhibit very high interpersonal EI.
	*Intrapersonal* [234, 240, 241, 245, 253, 264, 285, 289, 367]	Bar-On’s Emotional Quotient Inventory (EQ-I) - 87 items	277	2.82	0.25	Nurse exhibit medium interpersonal EI.
		Bar-On’s Emotional Quotient Inventory (EQ-I) - 87 items sum	312	111.74	12.72	*Information not available for interpretation*
		Bar-On’s Emotional Quotient Inventory (EQ-I) - 133 item	598	128.63	24.00	Nurse exhibit high intrapersonal skills.
		Emotional Intelligence Scale (EQS)	1150	38.21	6.01	*Information not available for interpretation*
		Reduced Emotional Intelligence Inventory for Adults (EQ-i-20 M)	2126	2.62	0.70	*Information not available for interpretation*
	*Managing emotions* [229, 259, 268, 358]	Self-Rated Emotional Intelligence Scale	101	4.17	0.47	Nurses exhibit slightly lower ability to manage emotions.
		GENOS Emotional Intelligence Self-Assessment	194	3.50	0.71	*Information not available for interpretation*
		Mayer-Salovey-Caruso Emotional Intelligence Test	260	100.33	1.54	Nurse exhibit average managing emotions.
	*Managing others emotions* [249, 269, 276, 296, 312]	Schutte Self-Report Emotional Intelligence Test	448	3.73	0.39	Nurse scored 3.73 out of 5, where higher score = higher EI.
		Emotional Intelligence Scale (EIS)	883	22.90	3.10	*Information not available for interpretation*
		GENO EI 31 item	248	3.45	0.68	*Information not available for interpretation*
		Schutte Self-Report Emotional Intelligence Test (SSEIT) - sum score	1271	29.97	3.81	*Information not available for interpretation*
	*Managing own emotions* [249, 276, 296]	Schutte Self-Report Emotional Intelligence Test	448	4.09	0.37	Nurse scored 4.09 out of 5, where higher score = higher EI.
		Schutte Self-Report Emotional Intelligence Test (SSEIT) - sum score	1566	34.52	0.66	*Information not available for interpretation*
	*Managing relations* [238, 239]	Emotional Competence Inventory	80	97.92	1.06	*Information not available for interpretation*
		Emotional Intelligence Scale	120	14.18	0.00	Nurses exhibit positive managing relations.
	*Motivation* [238]	Emotional Intelligence Scale	120	21.28	0.00	Nurses exhibit positive motivation.
	*Optimism/emotion regulation* [292, 356, 361]	Schuttes Emotional Intelligence Test	50	46.68	6.11	*Information not available for interpretation*
		Revised Schutte Emotional Intelligence Scale (RSEIS)	218	78.90	12.10	*Information not available for interpretation*
		Schutte Self-Report Emotional Intelligence Test (SSEIT) - sum score	156	39.67	5.48	*Information not available for interpretation*
	*Others emotional appraisal* [235, 246, 247, 251, 263, 266, 270, 273, 286–288, 294, 297, 308]	Self Emotional Intelligence Scale	67	3.43	0.34	Nurses scored 3.43 out of 5, where lower score = more uncertain and lower EI.
		Wong and Law Emotional Intelligence Scale (WLEIS) - 4 point scale	280	3.00	0.50	Nurses scored 3.00 out of 4, where higher scores = higher EI.
		Wong and Law Emotional Intelligence Scale – 5-point scale	1577	3.57	0.13	Nurses scored 3.57 out of 5, where higher scores = higher EI.
		Wong and Law Emotional Intelligence Scale – 5-point scale (sum)	423	13.30	0.95	Nurses scored 13.30 out of 20, where higher scores = higher EI.
		Wong and Law Emotional Intelligence Scale – 6-point scale (sum)	228	18.10	3.93	Nurses scored 18.1 out of 24, where higher scores = higher EI.
		Wong and Law Emotional Intelligence Scale – 7-point scale	177	4.61	0.93	Nurses scored 4.61 out of 7, where higher scores = higher EI.
		Wong and Law Emotional Intelligence Scale (WLEIS-C) - Chinese version	202	17.11	3.82	*Information not available for interpretation*
		Korean Emotional Intelligence Scale	300	3.71	0.61	Nurses scored 3.89 out of 5, where higher score = higher EI.
	*Perception of emotion* [249, 259, 276, 296, 312]	Self-Rated Emotional Intelligence Scale	101	4.17	0.48	Nurses exhibit slightly lower ability to perceive emotions.
		Schutte Self-Report Emotional Intelligence Test	448	3.70	0.38	Nurse scored 3.7 out of 5, where higher score = higher EI.
		Emotional Intelligence Scale (EIS)	883	42.00	5.20	*Information not available for interpretation*
		Schutte Self-Report Emotional Intelligence Test (SSEIT) - sum score	1566	37.81	0.00	*Information not available for interpretation*
	*Recognising and expressing emotions* [229, 269]	GENOS Emotional Intelligence Self-Assessment	194	3.51	0.75	*Information not available for interpretation*
		GENO EI 31 item	248	0.60	0.66	*Information not available for interpretation*
	*Regulation of emotion* [213, 235, 246, 247, 251, 263, 266, 270, 271, 273, 286–288, 294, 297, 308, 314]	Self Emotional Intelligence Scale	67	2.63	0.25	Nurses scored 2.63 out of 5, where lower score = more uncertain and lower EI.
		Wong and Law Emotional Intelligence Scale (WLEIS) - 4 point scale	280	3.00	0.60	Nurses scored 3.00 out of 4, where higher scores = higher EI.
		Wong and Law Emotional Intelligence Scale – 5-point scale	1577	3.47	0.28	Nurses score 3.47 out of 5, where higher scores = higher EI.
		Wong and Law Emotional Intelligence Scale – 5-point scale (sum)	423	13.76	0.64	Nurses scored 13.76 out of 20, where higher scores = higher EI.
		Wong and Law Emotional Intelligence Scale (WLEIS) - 6 point scale	580	5.54	0.90	Nurses scored 5.54 out of 6, where higher score = higher EI.
		Wong and Law Emotional Intelligence Scale – 6-point scale (sum)	228	19.82	3.30	Nurses scored 19.82 out of 24, where higher scores = higher EI.
		Wong and Law Emotional Intelligence Scale – 7-point scale	66	3.53	0.63	Nurses scored 3.53 out of 7, where higher scores = higher EI.
		Wong and Law Emotional Intelligence Scale (WLEIS-C) - Chinese version	202	17.57	3.92	*Information not available for interpretation*
		Brief Emotional Intelligence Scale (BEIS-10)	395	2.33	0.00	*Information not available for interpretation*
		Korean Emotional Intelligence Scale	300	3.52	0.60	Nurses scored 3.52 out of 5, where higher score = higher EI.
	Regulation of others emotions [213]	Brief Emotional Intelligence Scale (BEIS-10)	395	2.39	0.24	*Information not available for interpretation*
	*Relationship management* [79, 262, 275, 300, 310]	Bradberry & Greaves Emotional Intelligence Questionnaire	388	25.84	0.43	Nurses scored 35.84 out of 48, where higher score = high EI
		Bradberry & Greaves Emotional Intelligence Questionnaire 100 scale	581	73.99	9.90	Nurses score 73.99 out of 100, where higher scores = higher EI.
	*Self emotional appraisal* [235, 246, 247, 251, 263, 266, 270, 271, 273, 286–288, 294, 297, 308]	Self Emotional Intelligence Scale	67	3.30	0.25	Nurses scored 3.30 out of 5, where lower score = more uncertain and lower EI.
		Wong and Law Emotional Intelligence Scale (WLEIS) - 4 point scale	280	3.10	0.50	Nurses scored 3.00 out of 4, where higher scores = higher EI.
		Wong and Law Emotional Intelligence Scale – 5-point scale	1577	3.76	0.25	Nurses scored 3.76 out of 5, where higher scores = higher EI.
		Wong and Law Emotional Intelligence Scale – 5-point scale (sum)	423	15.95	0.42	Nurses scored 15.96 out of 20, where higher scores = higher EI.
		Wong and Law Emotional Intelligence Scale (WLEIS) - 6 point scale	580	5.59	0.06	Nurses scored 5.59 out of 6, where higher score = higher EI.
		Wong and Law Emotional Intelligence Scale – 6-point scale (sum)	228	19.53	3.29	Nurses scored 19.53 out of 24, where higher scores = higher EI.
		Wong and Law Emotional Intelligence Scale – 7-point scale	177	4.60	0.98	Nurses scored 4.60out of 7, where higher scores = higher EI.
		Wong and Law Emotional Intelligence Scale (WLEIS-C) - Chinese version	202	17.51	3.28	*Information not available for interpretation*
		Korean Emotional Intelligence Scale	300	3.89	0.54	Nurses scored 3.89 out of 5, where higher score = higher EI.
	*Self-awareness* [79, 238, 239, 255, 262, 269, 274, 275, 300, 302, 303, 309, 310]	Bradberry & Greaves Emotional Intelligence Questionnaire	388	27.80	0.87	Nurses scored 27.80 out of 36, where higher score = high EI
		Bradberry & Greaves Emotional Intelligence Questionnaire 100 scale	581	75.29	7.66	Nurses score 75.29 out of 100, where higher scores = higher EI.
		Emotional Competence Inventory (ECI)	80	35.55	0.12	*Information not available for interpretation*
		Emotional Intelligence Scale (EIS)	120	16.13	0.00	Nurses exhibit positive self-awareness.
		Goleman’s Emotional Intelligence Scale	253	20.83	3.94	*Information not available for interpretation*
		Emotional Intelligence Assessment Scale (EIA)	490	27.97	0.11	*Information not available for interpretation*
		GENOS EI 31 item	248	3.61	0.65	*Information not available for interpretation*
		Shrink’s Emotional Intelligence Questionnaire	256	23.25		*Information not available for interpretation*
		Siberia Schering’s Emotional Intelligence Standard Questionnaire	500	29.01	4.70	*Information not available for interpretation*
	*Self-control* [228, 252, 269, 278, 303, 304, 309]	Trait Emotional Intelligence Questionnaire-Short Form	627	5.00	0.13	Nurses exhibit higher than normal self-control.
		Trait Emotional Intelligence Questionnaire-Short Form (TEIQue-SF) - sum score	100	32.42	4.51	*Information not available for interpretation*
		GENOS EI 31 item	248	3.55	0.62	*Information not available for interpretation*
		Shrink’s Emotional Intelligence Questionnaire	256	16.24		*Information not available for interpretation*
		Siberia Schering’s Emotional Intelligence Standard Questionnaire	500	22.98	4.16	*Information not available for interpretation*
	*Self-development* [238]	Emotional Intelligence Scale	120	7.22	0.00	Nurses exhibit positive self-development.
	*Self-expression* [20, 237]	Bar-On’s Emotional Quotient Inventory 2.0	166	105.63	0.33	Nurses exhibit medium self-expression.
	*Self-management* [79, 239, 255, 262, 269, 275, 300, 310]	Bradberry & Greaves Emotional Intelligence Questionnaire	388	32.31	3.38	Nurses scored 32.31 out of 54, where higher score = high EI
		Bradberry & Greaves Emotional Intelligence Questionnaire 100 scale	581	67.00	17.28	Nurses score 67.00 out of 100, where higher scores = higher EI.
		Emotional Intelligence Scale (EIS)	883	30.10	3.50	*Information not available for interpretation*
		GENOS EI 31 item	248	3.77	0.65	*Information not available for interpretation*
		Emotional Competence Inventory	80	76.35	0.26	*Information not available for interpretation*
		Goleman’s Emotional Intelligence Scale	253	18.19	4.05	*Information not available for interpretation*
	*Self-motivation* [274, 302, 303, 309]	Emotional Intelligence Assessment Scale (EIA)	490	28.84	1.24	*Information not available for interpretation*
		Shrink’s Emotional Intelligence Questionnaire	256	18.74		*Information not available for interpretation*
		Siberia Schering’s Emotional Intelligence Standard Questionnaire	500	23.08	3.25	*Information not available for interpretation*
	*Self-perception* [20, 237]	Bar-On’s Emotional Quotient Inventory 2.0	166	104.39	1.47	Nurses exhibit medium self-perception.
	*Self-regulation* [274, 302]	Emotional Intelligence Assessment Scale (EIA)	490	28.26	2.59	*Information not available for interpretation*
	*Situational* [240, 285]	Emotional Intelligence Scale	1150	38.25	5.53	*Information not available for interpretation*
	*Sociability* [228, 252, 278, 304]	Trait Emotional Intelligence Questionnaire-Short Form	627	4.90	0.35	Nurses exhibit higher than normal sociability.
		Trait Emotional Intelligence Questionnaire-Short Form (TEIQue-SF) - sum score	100	30.13	4.83	*Information not available for interpretation*
	*Social awareness* [79, 239, 255, 262, 275, 300, 310]	Bradberry & Greaves Emotional Intelligence Questionnaire	388	23.66	5.38	Nurses scored 23.66 out of 30, where higher score = high EI
		Bradberry & Greaves Emotional Intelligence Questionnaire 100 scale	581	73.14	12.31	Nurses score 73.14 out of 100, where higher scores = higher EI.
		Emotional Competence Inventory	80	33.74	0.12	*Information not available for interpretation*
		Goleman’s Emotional Intelligence Scale	253	19.40	3.25	*Information not available for interpretation*
	*Social skills* [255, 274, 302, 303, 309]	Goleman’s Emotional Intelligence Scale	253	19.89	4.03	*Information not available for interpretation*
		Emotional Intelligence Assessment Scale (EIA)	490	28.46	2.55	*Information not available for interpretation*
		Shrink’s Emotional Intelligence Questionnaire	256	14.46		*Information not available for interpretation*
		Siberia Schering’s Emotional Intelligence Standard Questionnaire	500	17.04	3.14	*Information not available for interpretation*
	*Stress Management* [20, 233, 234, 237, 241, 245, 253, 264, 289, 368]	Bar-On’s Emotional Quotient Inventory - 133 item	640	64.84	30.73	Nurses exhibit markedly low stress management.
		Bar-On’s Emotional Quotient Inventory - 87 items	277	2.99	0.38	Nurses exhibit medium stress management.
		Bar-On’s Emotional Quotient Inventory (EQ-I) - 87 items sum	312	43.14	7.13	*Information not available for interpretation*
		Bar-On’s Emotional Quotient Inventory 2.0	166	106.91	0.00	Nurses exhibit medium stress management.
		Reduced Emotional Intelligence Inventory for Adults	2126	3.25	0.57	Nurses scored 3.25 out of 4, where higher score = higher EI.
	*Understanding branch* [259, 268, 358]	Self-Rated Emotional Intelligence Scale	101	4.09	0.73	Nurses exhibit lower ability to understand emotions.
		Mayer-Salovey-Caruso Emotional Intelligence Test	260	100.443	2.27	Nurses exhibit average understanding of emotions.
	*Understanding others emotions* [229]	GENOS Emotional Intelligence Self-Assessment	194	3.42	0.76	*Information not available for interpretation*
	*Use of emotion* [235, 246, 247, 249, 251, 259, 263, 266, 268, 270, 271, 273, 276, 286–288, 292, 294, 296, 297, 308, 312, 358, 361]	Mayer-Salovey-Caruso Emotional Intelligence Test	260	98.89	1.28	Nurses exhibit average use of emotions.
		Self Emotional Intelligence Scale	67	3.17	0.00	Nurses score 3.17 out of 5, where lower score = more uncertain and lower EI.
		Wong and Law Emotional Intelligence Scale (WLEIS) - 4 point scale	280	3.10	0.60	Nurses scored 3.00 out of 4, where higher scores = higher EI.
		Wong and Law Emotional Intelligence Scale – 5-point scale	1577	3.63	0.37	Nurses scored 3.63 out of 5, where higher scores = higher EI.
		Wong and Law Emotional Intelligence Scale – 5-point scale (sum)	335	16.69	0.37	Nurses scored 16.69 out of 20, where higher scores = higher EI.
		Wong and Law Emotional Intelligence Scale (WLEIS) - 6 point scale	580	5.58	0.87	Nurses score 5.54 out of 6, where higher scores = higher EI.
		Wong and Law Emotional Intelligence Scale WLEIS) – 6-point scale (sum)	228	20.77	3.25	Nurses scored 20.77 out of 24, where higher scores = higher EI.
		Wong and Law Emotional Intelligence Scale – 7-point scale	177	4.08	0.47	Nurses scored 4.08 out of 5, where higher scores = higher EI.
		Wong and Law Emotional Intelligence Scale (WLEIS-C) - Chinese version	202	17.57	3.38	*Information not available for interpretation*
		Self-Rated Emotional Intelligence Scale	101	3.34	0.40	Nurses exhibit lower ability to use emotions.
		Schutte Self-Report Emotional Intelligence Test	448	3.88	0.43	Nurse scored 3.88 out of 5, where higher score = higher EI.
		Schutte Self-Report Emotional Intelligence Test (SSEIT) - sum score	1722	23.84	4.89	*Information not available for interpretation*
		Emotional Intelligence Scale (EIS)	883	27.40	3.70	*Information not available for interpretation*
		Korean Emotional Intelligence Scale	300	3.14	0.70	Nurses scored 3.14 out of 5, where higher score = higher EI
		Revised Schutte Emotional Intelligence Scale (RSEIS)	218	48.50	6.60	*Information not available for interpretation*
	*Value orientation* [238]	Emotional Intelligence Scale	120	7.11	0.00	Nurses exhibit positive value orientation.
	*Well-being* [228, 252, 278, 304]	Trait Emotional Intelligence Questionnaire-Short Form	627	5.88	0.23	Nurses exhibit higher than normal well-being.
		Trait Emotional Intelligence Questionnaire-Short Form (TEIQue-SF) - sum score	100	37.21	3.20	*Information not available for interpretation*
**Dentist**	*Emotional Intelligence* [343–345]	Emotional Intelligence Screening Test	133	172.31	16.40	Dentists exhibit high emotional intelligence (172.31/208).
		Schutte Self-Report Emotional Intelligence Test - sum score	528	128.52	16.25	Dentists exhibit high levels of emotional intelligence (128.52/165).
	*Empathy* [343]	Jefferson Scale Physician Empathy	186	92.04	9.43	Dentists exhibit high levels of empathy.
**Occupational Therapist**	*Emotional Intelligence (total)* [346, 347]	Swinburne University Emotional Intelligence Test	134	229.51	21.11	Occupational therapists scored 229.51 out of 320, where higher score = higher EI.
		Trait Emotional Intelligence Questionnaire-Short Form	1235	5.41	0.01	Occupational therapists exhibit higher than normal emotional intelligence.
	*Emotional control* [347]	Swinburne University Emotional Intelligence Test	134	31.67	4.40	*Information not available for interpretation*
	*Emotional management* [347]	Swinburne University Emotional Intelligence Test	134	41.41	5.31	*Information not available for interpretation*
	*Emotional recognition & expression* [347]	Swinburne University Emotional Intelligence Test	134	39.65	5.26	*Information not available for interpretation*
	*Emotions direct cognition* [347]	Swinburne University Emotional Intelligence Test	134	38.03	6.19	*Information not available for interpretation*
	*Understanding emotions* [347]	Swinburne University Emotional Intelligence Test	134	78.46	8.24	*Information not available for interpretation*
	*Well-being* [348]	Trait Emotional Intelligence Questionnaire-Short Form (TEIQue-SF)	808	5.79	0.82	Occupational therapists exhibit higher than normal emotional intelligence.
	*Self-control* [348]	Trait Emotional Intelligence Questionnaire-Short Form (TEIQue-SF)	808	4.93	0.88	Occupational therapists exhibit higher than normal emotional intelligence.
	*Emotionality* [348]	Trait Emotional Intelligence Questionnaire-Short Form (TEIQue-SF)	808	5.76	0.73	Occupational therapists exhibit higher than normal emotional intelligence.
	*Sociability* [348]	Trait Emotional Intelligence Questionnaire-Short Form (TEIQue-SF)	808	5.07	0.78	Occupational therapists exhibit higher than normal emotional intelligence.
**Physiotherapist**	*Emotional Intelligence (total)* [350]	Genos Emotional Intelligence Inventory – Concise Questionnaire (GEII)	171	129.36	18.31	*Information not available for interpretation*

#### Health professionals

Health professionals’ EI was investigated in six studies (*n* = 1973) utilising the Multidimensional Measure of Emotional Intelligence [31, 353], Bar-On’s Emotional Quotient Inventory [352], Brief Emotional Intelligence Inventory for Senior Citizens [354], Wong and Law Emotional Intelligence Scale [355] and Schutt Self-Reports Emotional Intelligence Test [342]. All six studies demonstrated that health professionals generally have average EI, and this trend is observed across each subscale of EI also as outlined in Table 4.

#### Nursing

Global emotional intelligence scores varied in nurses from low [259] (1 study; *n* = 131), average [20, 82, 233, 237, 241, 245, 254, 259, 267, 268, 277, 295, 298, 299, 301, 306, 307, 358] (18 studies, *n* = 2521), to above average and higher [79, 230, 231, 238, 249, 262, 290, 310] (8 studies, *n* = 2011). Nurses also demonstrated average adaptability [233, 241, 245, 253, 264] (5 studies, *n* = 827); positive altruistic behaviours, commitment and emotional stability [238] (1 study, *n* = 170); and high emotionality [228, 252] (2 studies, *n* = 582). Interpersonal abilities ranged from low [233] (1 study, *n* = 277) to average [20, 234, 237, 240, 241, 245, 264] (7 studies, *n* = 3857). However interpersonal relationships were reported to be very high in nurses [241, 245, 253, 264] (4 studies, *n* = 550). These results suggested that nurses have low to average EI overall, but positively exhibit self-less, committed and emotional stable relationships.

#### Medical

Global EI scores for medical practitioners ranged from average [253, 257, 317, 320, 321, 339] (6 studies, *n* = 915) to above average [265, 316, 318, 319, 322, 327] (6 studies, *n* = 3367), across seven different outcome tools. Subscale items of EI results demonstrated medical practitioners exhibit high natural acting emotional labour strategies [318] (1 study, *n* = 740), intra and interpersonal emotional intelligence [253] (1 study, *n* = 120), and self-control [316, 319, 327] (3 studies, *n* = 2377); whilst also displaying low benefiting from emotions [257] (1 study, *n* = 50), and markedly low general mood and stress management [253] (1 study, *n* = 120). These results suggest doctors have high ability to manage their own emotions, and require less effort to change their emotions, but potentially struggle with feeling satisfied. Nooryan, Gasparyan [253] established that training in EI within medical professionals also aids in reducing stress.

#### Dentistry

Dentists were found to exhibit high levels of emotional intelligence across three studies (*n* = 661), utilising the Schutte Self-Report Emotional Intelligence Test [343, 344] and Emotional Intelligence Screening Test [345]. Dentists also were reported to have light levels of empathy [343], which suggests that dentists have a greater than average ability to appreciate the emotions of others and can more easily understand their patients point-of-view.

#### Occupational therapy

Occupational therapists had higher than normal EI across three studies (*n* = 1369) [346–348]. One utilised the Trait Emotional Intelligence Questionnaire-Short Form [346] examining global EI. The second study utilised the Swinburne University Emotional Intelligence Test, which identified occupational therapists have a high ability to understand emotions (78.46 ± 8.24) [347]. Finally, McKenna, Webb [348] utilised the Trait Emotional Intelligence Questionnaire-Short Form (TEIQue-SF), which identified higher than normal scores across all sub scales of well-being (5.79 ± 0.82), self-control (4.93 ± 0.88), emotionality (5.76 ± 0.73) and sociability (5.07 ± 0.78).

#### Radiology

Abu Awwad, Lewis [351] investigated chief radiologist emotional intelligence relative to their years of experience. Results found high mean scores across global and subscale scores (5.15–6.25) with no significant differences found across global, subscale scores, or years of experience [351].

#### Physiotherapy

Nizar Abdul Majeed, Mohammed Abdulrazzaq [350] was the only study to assess physiotherapy emotional intelligence utilising the Genos Emotional Intelligence Inventory – Concise Questionnaire (GEII). Results indicated that physiotherapists have a moderate (129.36 ± 18.314) level of emotional intelligence, that is negatively correlated with occupational stress [350].

## Discussion

The primary purpose of this systematic review was to profile the personality traits, behaviour styles, and EI of qualified health practitioners. By meta-aggregating results from multiple studies, we aimed to explore the differences and similarities between the health professions, critically appraising and collating the empirical literature reporting on this topic. In total, 321 publications were included with 68% achieving high methodological quality score on the MMAT checklist. The distribution of health professionals across the systematic review demonstrates that research is limited and inconsistent within this field with at least one of the non-cognitive traits being investigated in each of the 13 health professional groups; nursing (personality, behaviour, emotional intelligence), medicine (personality, EI), nursing assistants (personality), dentistry (personality, EI), dietetics (personality), occupational therapy (personality, behaviour, EI), physiotherapy (personality, EI), pharmacy (personality), psychologists (behaviour), radiologists (personality, EI), social workers (personality), pathologists (personality) and paramedics (personality).

### Personality traits

The results from included studies suggest that all health professionals demonstrate agreeable, cooperative and self-directed traits, with low levels of neuroticism supporting the concept that health professionals are relaxed, calm, stable individuals who have the ability to work well in teams, which are all required within the complex social context of healthcare environments [369]. However, most other personality traits exhibit some variation across health professional groups. Recognising that non-cognitive traits can enhance individual understanding and possibly predict the conduct of health professionals Eley and Eley [1] it is important to explore these differences.

One of the most consistent results pertaining to health professional personalities is the sensing-judging trait. Most health professional groups exhibit high sensing-judging scores on personality measures, suggesting that they perceive information through direct, objective, factual senses and utilise mental functions through structured planning that is decisive, controlled and committed [370]. Occupational therapists are an exception to this, exhibiting higher sensing-perceiving and intuitive-feeling, suggesting they have more indecisive mental functions, subjective perceptions and make decisions based on experiences and compassion. One explanation for this variation within occupational therapy is that these health professionals tend to place more emphasis on psychological well-being, giving greater attention to occupational performance of the whole person, compared to a body structure impairment approach taken by many of the other health professions [191].

Extroversion-introversion personality trait varied across professions with nursing and medicine demonstrating variability of these traits across the continuum. Comparatively, physiotherapists and pharmacists all showed greater attenuation to extroversion. Previous research supports this with person-orientated health professionals such as physiotherapists working in more socially engaging roles over longer occasions of service and therefore attracting more extroverted individuals to the profession [188]. Interestingly, pharmacists possess both person-oriented and technique-orientated skills, with non-homogeneous personality traits dependant on the primary skills required within their role [189]. The results of this systematic review suggest that most pharmacists are in person-orientated positions that are conducive to a more extroverted nature, with higher social interactions, than other technique-oriented positions which are more physically or emotionally separate from patients [188] which attract more introverted individuals.

Nurses and medical practitioners were found to have higher levels of dominance and lower levels of abstractedness suggesting that they are inclined to be more assertive, forceful and stubborn, and are grounded, practical, solution-orientated individuals [106]. Dominance relates to the amount of control that an individual either submits to or exercises over others, ranging from dominance to submissive [371, 372]. The current review suggests that both medical practitioners and nurses tend to exhibit more control, are not concerned with conflict, and will exhibit traits of assertiveness when presenting their views. This is of particular interest given the proximity of the working relationship for these professions, where they frequently perceive differences in clinical assessment data or intervention techniques which could create disagreement [373] and impact the collaborative team approach to healthcare if each did not trust and respect the input of the other professional.

### Behaviour styles

Personality traits are believed to contribute to an individual’s behaviour style, which is an expression of internally coordinated responses to both internal and external stimuli [11]. Behaviour Style was only investigated in four professions. Nurses exhibited both Type A and B behaviour, with higher association to Type A. Type A behaviour is associated with competitiveness, time urgency and tendency towards anger and hostility with an external locus of control; whereas Type B represents easy-going, relaxed and unhurried behavioural tendencies, internal locus of control with less compulsive and perfectionistic behaviours than Type A [221, 374]. Great attenuation of Type A behaviours aligns with nurses’ personality traits of dominance which supports the inclination of more assertive behaviours, influenced by viewing time as an enemy. Given the clinical environment is considered to be unpredictable, challenging and stressful [375], this would predispose nurses to frustration, which has been previously documented as counterproductive to career success [374]. Similarly, male psychology scientists were also associated with having higher Type A behaviours, identifying that the more Type A behaviour is exhibited, the more likely an individual is to prefer challenging tasks and competitiveness that influences work satisfaction.

Occupational therapists demonstrated differences in behaviours dependant on their role as a clinician or administrator. It perhaps is not surprising that clinicians are demonstrating higher lovingness, inner harmony and mature love, as health professionals are expected to be compassionate and empathetic when working with patients, where administrators are sought for their more pragmatic and objective characteristics [225]. Administrators also often undertake the operational management of others, which may explain why they demonstrate a higher emphasis on capability than clinicians. The research demonstrates that there is a behavioural focus shift from clinician to administrator that could help individuals to identify areas for development if they wish to transition between these roles. Additionally, it may also be helpful for individuals making decisions about entry into higher education programs and career choices, identifying areas to focus development that might not be aligned with their preferred behavioural style.

Medical professionals were found to be more successful in executing tasks, high performance on examination and high level patient safety with high behaviour profiles of Dominance and Conscientiousness, acknowledging the competitive nature of health care and that these traits are associated with high level performance which are required in the medical field.

The results of the current review provide nurses, psychologists and doctors with information regarding the drivers of their behaviours, potential influences on how they interact with the team, and implications for career success. It is therefore important to consider these traits not only for career success, but in the education of health professional students. If educators and students are informed of the typical profiles of qualified health professionals, and the implications of these on their performance; this knowledge could be applied during student education and training within both the university and clinical learning environment settings, potentially leading to desirable behavioural change and improved performance [12].

### Emotional intelligence

EI is known to influence an individuals’ ability to perceive, understand and cope with the environmental demands and pressures [14]. Therefore awareness and ability to monitor one’s own emotional response and others’ feelings, whilst discriminating between them, provides useful information for clinicians to guide their thinking and actions [376]. The literature demonstrates high homogeneity of overall EI scores within professional groups, with most health professionals exhibiting average to above average EI. The exception was nurses who, on aggregated results, demonstrated low to average EI. Howie, Heaney [305] has suggested that higher levels of EI could explain why some practitioners are better at delivering patient-centred care than others. Despite demonstrating lower global EI, nurses demonstrated very high interpersonal relationships, with emotional stability and commitment to their patients than their medical practitioner colleagues. This supports literature outlining the importance of the nurse-patient relationship, which indicates that nurses who are unable to develop a relationship with patients are more likely to have patients demonstrating ‘difficult’ behaviours, impacting on patient care [377].

Despite medical professionals exhibiting average EI, they perceive that the benefits of emotions are low, suggesting that they do not perceive emotionality in their work context as important and are less likely to utilise it. Lucius-Hoene, Thiele [378], identified that medical practitioners tend to communicate in a neutral manner without emotional content. However, if medical practitioners were to display appreciation, sympathy and support, they may play a pivotal role in the patients coming to terms with their illness and feeling understood and respected [378]. There is very little literature identifying why there is a difference between professional groups in terms of EI subscales. However, it is possible that nurses have a higher interpersonal and emotional stability than medical practitioners because they historically would spend more direct clinical care time with a patient.

### Implications for health professions

The studies included in this review have identified both consistency and variation between health professionals with respect to personality traits, behavioural styles and EI, with various implications to professional practice and patient interactions. Considering the evidence, the characterisation of health professionals based on these traits will aid health professionals to understand their own non-cognitive features and how these might be useful in predicting performance within their chosen profession [1] with the potential to adapt these to enhance success in their professional roles. Utilisation of this information could be implemented at all stages of a clinician’s career development. This could start from pre-registration, providing students with an understanding and systematic training in humanistic qualities [379] within their programs, including decision making for entry into a health professional program. Further this could continue within the professions to target training to reduce stress and burnout [253] and enhance teamwork and communication between professions [380].

Despite highlighting the benefits of being able to understand health professional non-cognitive characteristics, it is evident that there are gaps in the literature in profiling these traits across all health professional groups. To date, most research has been in the medical and nursing professions, with the majority of literature focused on personality and EI. There is inconsistency in tools used to measure traits, making comparisons within and between professions difficult. There is limited research exploring non-cognitive characteristics of allied health practitioners and very little exploration of behaviour styles in health practitioners.

This review is the first of its kind and provides substantial aggregated information to inform readers about the personality traits, behaviour styles, and EI of health professionals. However, gaps in the literature are evident where several health professional groups are not represented across all measured factors, and there is a lack of literature on behaviour styles of health professionals when compared to other characteristics. This systematic review represents the most comprehensive review to date of the literature relating to health professional non-cognitive characteristics, capturing 321 studies across a range of health professions.

Understanding and knowledge of the non-cognitive profiles of health professionals would be valuable in supporting students before and during university training, as well as in their early career [1]. Understanding these non-cognitive traits provides academics, practitioners, clinical educators and students insight into how their own and other professionals’ traits might influence their engagement, success and challenges within academic training, clinical placement and the workplace. Furthermore, it provides students and health professionals greater knowledge to support decision making in selecting university programs, making career pathway choices and undertaking further professional development based on their own personality traits, behaviour styles and EI.

## Conclusion

Personality traits, behaviour styles and EI are non-cognitive characteristics of health professionals. All health professional groups demonstrate agreeable, cooperative, and self-directed personality traits with lower levels of neuroticism. However, physiotherapists and pharmacists have a higher level of extroversion which is likely to be related to the person-oriented aspects of their role compared to other health professional groups. Medicine and nursing are more dominant and less abstracted in their expression of personality and are inclined to be more assertive and forceful than other professions. Nurses and psychologists tend to exhibit Type A behaviour styles, including higher levels of competitiveness, time urgency, and with an external locus of control. Comparatively, occupational therapists appear to demonstrate behaviour dependent on their role, with clinician behaviour focused on patient interactions, whereas administrators are more pragmatic and objective with a focus on the operational management of others. Collectively, health professionals exhibit average to above average global EI, except for nurses who demonstrate average to low EI on standardised assessments.

## Supplementary Information

**Additional file 1.** Search term concepts.

**Additional file 2.** Search terms and filters used in the systematic search by database.

**Additional file 3.** Personality, Behaviour, and Emotional Intelligence subscale/category and tools used to measure each function.

**Additional file 4.** Data Extraction Table.

## Data Availability

The dataset supporting the conclusions of this article is included within the article (and its additional files).

## Abbreviations

EI: Emotional intelligence
ESCI: Exploratory Software for Confidence Intervals
ESFJ: Extroversion-Sensing-Feeling-Judging
ESTJ: Extroversion-Sensing-Thinking-Judging
ISTJ: Introversion-Sensing-Thinking-Judging
MBTI: Myers-Briggs Type Indicator
MMAT: Mixed Methods Appraisal Tool
MSCEIT: Mayer-Salovey-Caruso Emotional Intelligence Test
NF: Intuitive-Feeling
PRISMA-P: Preferred Reporting Items for Systematic and Meta-Analysis Protocols
PROSPERO: Prospective Register of Systematic Reviews
SJ: Sensing-Judging
SP: Sensing-Perceiving
SPSS: Statistical Package for the Social Sciences
