# Supplementary material for: Profiling health professionals’ personality traits, behaviour styles and emotional intelligence: a systematic review
Source: BMC Med Educ. 2023 Feb 18;23:120. doi: 10.1186/s12909-023-04003-y (PMC9938999; doi:10.1186/s12909-023-04003-y)
Supplement: Supplementary file 1 — Additional file 1. Search term concepts. [file 12909_2023_4003_MOESM1_ESM.pdf]

Additional file 1. Search term concepts

Synonyms	Concept 1	AND	Concept 2
	Personalit*		"allied health"
OR	"personalit* tests"		physiotherap*
OR	"personality inventory"		"physical therap*"
OR	"behavior* style*"		Dentist*
OR	"behaviour* style*"		Dietician*
OR	"behaviour* profile*"		Dietitian*
OR	"behavior* profile*"		Dietetic*
OR	"behaviour* pattern*"		Nutritionist*
OR	"behavior* pattern*"		"Nutrition science*"
OR	"preferred behaviour*"		Podiatr*
OR	"preferred behavior"		Orthotist*
OR	"human behaviour*"		"occupational therap*"
OR	"human behavior"		"Speech patholog*"
OR	"behaviour state*"		"Speech-language patholog*"
OR	"behavior state*"		psychologist*
OR	"behaviour trait*"		"Medical Practitioner"
OR	"behavior trait"		Doctor*
OR	"behaviour tendenc*"		"medical physician"
OR	"behavior tendenc*"		chiropract*
OR	"observed behaviour*"		"general practitioner*"
OR	"observed behavior*"		GP*

OR	"behaviour attribute*"	nurs*
OR	"behavior attribute*"	midwi*
OR	"Emotional Intelligence*"	
OR	EI	
OR	"emotional intellect*"	
OR	"emotional competence"	
OR	"DiSC AND Profile"	
OR	"DiSC AND Personalit*"	
OR	"DiSC AND Behavio*"	
OR	"DiSC AND Trait*"	
OR	"Five factor model*"	
OR	FFM*	
OR	"myers briggs*"	
OR	"myers-briggs*"	
OR	MBTI	
OR	"temperament and character inventory"	
OR	"Eysenck personality questionnaire"	
OR	EPQ	
OR	"NEO five factor inventory of personality"	
OR	"trait emotional intelligence"	
OR	TEI	
OR	"hamburg personality inventory"	

OR	HPI
OR	"emotional quotient inventory"
OR	EQI
OR	"emotional intelligence scale"
OR	EIS
OR	extroversion
OR	introversion
OR	agreeableness
OR	openness
OR	neuroticism
OR	conscientiousness

* Reflects truncation of each term.
