# Supplementary material for: Profiling health professionals’ personality traits, behaviour styles and emotional intelligence: a systematic review
Source: BMC Med Educ. 2023 Feb 18;23:120. doi: 10.1186/s12909-023-04003-y (PMC9938999; doi:10.1186/s12909-023-04003-y)
Supplement: Supplementary file 2 — Additional file 2. Search terms and filters used in the systematic search by database. [file 12909_2023_4003_MOESM2_ESM.pdf]

Additional file 2. Search terms and filters used in the systematic search by database.

Database	Date Searched	Search Terms	Filters[a]	Results
**PubMed**	09/09/2022	(("Personality"[majr:noexp] OR "Personalit*"[tiab] OR "Personality Tests"[majr] OR "Personality Test"[tiab] OR "personality tests"[tiab] OR "personality inventory"[tiab] OR "Personality Inventory"[majr] OR "Personality Inventories"[tiab] OR "behavior style*"[tiab] OR "behaviour style*"[tiab] OR "behaviour profile*"[tiab] OR "behavior profile*"[tiab] OR "behaviour pattern*"[tiab] OR "behavior pattern*"[tiab] OR "behavioral style*"[tiab] OR "behavioural style*"[tiab] OR "behavioural profile*"[tiab] OR "behavioral profile*"[tiab] OR "behavioural pattern*"[tiab] OR "behavioral pattern*"[tiab] OR "preferred behaviour*"[tiab] OR "preferred behavior"[tiab] OR "preferred behavior*"[tiab] OR "human behaviour*"[tiab] OR "human behavior"[tiab] OR "human behavior*"[tiab] OR "behaviour state*"[tiab] OR "behavior state*"[tiab] OR "behaviour trait*"[tiab] OR "behavior trait"[tiab] OR "behavior trait*"[tiab] OR "behaviour tendenc*"[tiab] OR "behavior tendenc*"[tiab] OR "behavioural tendenc*"[tiab] OR "behavioral tendenc*"[tiab] OR "observed behaviour*"[tiab]	Title and abstract 1980 -	8903

OR "observed behavior*"[tiab] OR "behaviour attribute*"[tiab] OR "behavior attribute*"[tiab] OR "Emotional Intelligence*"[tiab] OR "Emotional Intelligence"[majr] OR "emotional intellect*"[tiab] OR "emotional competence"[tiab] OR "emotional competen*"[tiab] OR "DiSC AND Profile"[tiab] OR "DiSC AND Personalit*"[tiab] OR "DiSC AND Behavio*"[tiab] OR "DiSC AND Trait*"[tiab] OR "Five factor model*"[tiab] OR "5 factor model*"[tiab] OR "myers briggs*"[tiab] OR "myers-briggs*"[tiab] OR "temperament and character inventory"[tiab] OR "Eysenck personality questionnaire"[tiab] OR "NEO five factor inventory of personality"[tiab] OR "trait emotional intelligence"[tiab] OR "hamburg personality inventory"[tiab] OR "emotional quotient inventory"[tiab] OR "emotional intelligence scale"[tiab] OR "extroversion"[tiab] OR "introversion"[tiab] OR "agreeableness"[tiab] OR "openness"[tiab] OR "neuroticism"[tiab] OR "conscientiousness"[tiab]) AND ("Allied Health Personnel/psychology"[majr] OR "allied health personnel"[tiab] OR "Allied Health Professional"[tiab] OR "Allied Health Professionals"[tiab] OR "Healthcare Assistant"[tiab] OR "Healthcare Assistants"[tiab] OR "Healthcare Support Worker"[tiab] OR "Healthcare Support Workers"[tiab] OR "Paramedic"[tiab] OR "Paramedical Personnel"[tiab] OR "Paramedics"[tiab] OR "Physical

		Therapists/psychology"[majr] OR "physiotherapist*"[tiab] OR "physical therapist*"[tiab] OR "Dentists/psychology"[majr] OR "Dentist"[tiab] OR "Dentists"[tiab] OR "Nutritionists/psychology"[majr] OR "Dietician*"[tiab] OR "Dietitian*"[tiab] OR "Nutritionist*"[tiab] OR "Podiatry"[majr] OR "Podiatrist*"[tiab] OR "Orthotist*"[tiab] OR "Occupational Therapists/psychology"[majr] OR "occupational therapist*"[tiab] OR "Speech pathologist*"[tiab] OR "Speech-Language Pathology"[majr] OR "Speech-language pathologist*"[tiab] OR "Psychology/psychology"[majr] OR "psychologist*"[tiab] OR "Physicians/psychology"[majr] OR "Medical Practitioner*"[tiab] OR "Doctor*"[tiab] OR "medical physician*"[tiab] OR "chiropractor*"[tiab] OR "general practitioner*"[tiab] OR "Nurses/psychology"[majr] OR "Nursing Staff/psychology"[majr] OR "nurse"[tiab] OR "nurse"[tiab] OR "Midwifery"[majr] OR "midwi*"[tiab]))		
**CINHAL (Ebsco)**	09/09/2022	((Personality/de/mj OR Personalit*:ti,ab OR 'Personality Tests'/exp/mj OR 'Personality Test':ti,ab OR 'personality tests':ti,ab OR 'personality inventory':ti,ab OR 'Personality Inventory'/exp/mj OR 'Personality Inventories':ti,ab OR 'behavior style*':ti,ab OR 'behaviour style*':ti,ab OR 'behaviour profile*':ti,ab OR 'behavior profile*':ti,ab OR 'behaviour pattern*':ti,ab OR 'behavior pattern*':ti,ab	Title and abstract 1980 - English	3870

OR 'behavioral style*':ti,ab OR 'behavioural style*':ti,ab OR 'behavioural profile*':ti,ab OR 'behavioral profile*':ti,ab OR 'behavioural pattern*':ti,ab OR 'behavioral pattern*':ti,ab OR 'preferred behaviour*':ti,ab OR 'preferred behavior':ti,ab OR 'preferred behavior*':ti,ab OR 'human behaviour*':ti,ab OR 'human behavior':ti,ab OR 'human behavior*':ti,ab OR 'behaviour state*':ti,ab OR 'behavior state*':ti,ab OR 'behaviour trait*':ti,ab OR 'behavior trait':ti,ab OR 'behavior trait*':ti,ab OR 'behaviour tendenc*':ti,ab OR 'behavior tendenc*':ti,ab OR 'behavioural tendenc*':ti,ab OR 'behavioral tendenc*':ti,ab OR 'observed behaviour*':ti,ab OR 'observed behavior*':ti,ab OR 'behaviour attribute*':ti,ab OR 'behavior attribute*':ti,ab OR 'Emotional Intelligence*':ti,ab OR 'Emotional Intelligence'/exp/mj OR 'emotional intellect*':ti,ab OR 'emotional competence':ti,ab OR 'emotional competen*':ti,ab OR 'DiSC AND Profile':ti,ab OR 'DiSC AND Personalit*':ti,ab OR 'DiSC AND Behavio*':ti,ab OR 'DiSC AND Trait*':ti,ab OR 'Five factor model*':ti,ab OR '5 factor model*':ti,ab OR 'myers briggs*':ti,ab OR myers-briggs*:ti,ab OR 'temperament and character inventory':ti,ab OR 'Eysenck personality questionnaire':ti,ab OR 'NEO five factor inventory of personality':ti,ab OR 'trait emotional intelligence':ti,ab OR 'hamburg personality inventory':ti,ab OR 'emotional quotient inventory':ti,ab OR 'emotional

intelligence scale':ti,ab OR extroversion:ti,ab OR introversion:ti,ab OR agreeableness:ti,ab OR openness:ti,ab OR neuroticism:ti,ab OR conscientiousness:ti,ab) AND ('Allied Health Personnel/psychology'/exp/mj OR 'allied health personnel':ti,ab OR 'Allied Health Professional':ti,ab OR 'Allied Health Professionals':ti,ab OR 'Healthcare Assistant':ti,ab OR 'Healthcare Assistants':ti,ab OR 'Healthcare Support Worker':ti,ab OR 'Healthcare Support Workers':ti,ab OR Paramedic:ti,ab OR 'Paramedical Personnel':ti,ab OR Paramedics:ti,ab OR 'Physical Therapists/psychology'/exp/mj OR physiotherapist*:ti,ab OR 'physical therapist*':ti,ab OR ''Dentists/psychology''/exp/mj OR Dentist:ti,ab OR Dentists:ti,ab OR ''Nutritionists/psychology''/exp/mj OR Dietician*:ti,ab OR Dietitian*:ti,ab OR Nutritionist*:ti,ab OR Podiatry/exp/mj OR Podiatrist*:ti,ab OR Orthotist*:ti,ab OR 'Occupational Therapists/psychology'/exp/mj OR 'occupational therapist*':ti,ab OR 'Speech pathologist*':ti,ab OR 'Speech-Language Pathology'/exp/mj OR 'Speech-language pathologist*':ti,ab OR ''Psychology/psychology''/exp/mj OR psychologist*:ti,ab OR ''Physicians/psychology''/exp/mj OR 'Medical Practitioner*':ti,ab OR Doctor*:ti,ab OR 'medical physician*':ti,ab OR chiropractor*:ti,ab OR 'general practitioner*':ti,ab OR

		"Nurses/psychology"/exp/mj OR 'Nursing Staff/psychology'/exp/mj OR nurse:ti,ab OR nurse:ti,ab OR Midwifery/exp/mj OR midwi*:ti,ab))		
**EMBASE (Elsevier)**	09/09/2022	((Personality/de/mj OR Personalit*:ti,ab OR 'Personality Tests'/exp/mj OR 'Personality Test':ti,ab OR 'personality tests':ti,ab OR 'personality inventory':ti,ab OR 'Personality Inventory'/exp/mj OR 'Personality Inventories':ti,ab OR 'behavior style*':ti,ab OR 'behaviour style*':ti,ab OR 'behaviour profile*':ti,ab OR 'behavior profile*':ti,ab OR 'behaviour pattern*':ti,ab OR 'behavior pattern*':ti,ab OR 'behavioral style*':ti,ab OR 'behavioural style*':ti,ab OR 'behavioural profile*':ti,ab OR 'behavioral profile*':ti,ab OR 'behavioural pattern*':ti,ab OR 'behavioral pattern*':ti,ab OR 'preferred behaviour*':ti,ab OR 'preferred behavior':ti,ab OR 'preferred behavior*':ti,ab OR 'human behaviour*':ti,ab OR 'human behavior':ti,ab OR 'human behavior*':ti,ab OR 'behaviour state*':ti,ab OR 'behavior state*':ti,ab OR 'behaviour trait*':ti,ab OR 'behavior trait':ti,ab OR 'behavior trait*':ti,ab OR 'behaviour tendenc*':ti,ab OR 'behavior tendenc*':ti,ab OR 'behavioural tendenc*':ti,ab OR 'behavioral tendenc*':ti,ab OR 'observed behaviour*':ti,ab OR 'observed behavior*':ti,ab OR 'behaviour attribute*':ti,ab OR 'behavior attribute*':ti,ab OR 'Emotional Intelligence*':ti,ab OR 'Emotional Intelligence'/exp/mj OR 'emotional intellect*':ti,ab OR 'emotional	1980 - 2022	6159

competence':ti,ab OR 'emotional competen*':ti,ab OR 'DiSC AND Profile':ti,ab OR 'DiSC AND Personalit*':ti,ab OR 'DiSC AND Behavio*':ti,ab OR 'DiSC AND Trait*':ti,ab OR 'Five factor model*':ti,ab OR '5 factor model*':ti,ab OR 'myers briggs*':ti,ab OR myers-briggs*:ti,ab OR 'temperament and character inventory':ti,ab OR 'Eysenck personality questionnaire':ti,ab OR 'NEO five factor inventory of personality':ti,ab OR 'trait emotional intelligence':ti,ab OR 'hamburg personality inventory':ti,ab OR 'emotional quotient inventory':ti,ab OR 'emotional intelligence scale':ti,ab OR extroversion:ti,ab OR introversion:ti,ab OR agreeableness:ti,ab OR openness:ti,ab OR neuroticism:ti,ab OR conscientiousness:ti,ab) AND ('Allied Health Personnel/psychology'/exp/mj OR 'allied health personnel':ti,ab OR 'Allied Health Professional':ti,ab OR 'Allied Health Professionals':ti,ab OR 'Healthcare Assistant':ti,ab OR 'Healthcare Assistants':ti,ab OR 'Healthcare Support Worker':ti,ab OR 'Healthcare Support Workers':ti,ab OR Paramedic:ti,ab OR 'Paramedical Personnel':ti,ab OR Paramedics:ti,ab OR 'Physical Therapists/psychology'/exp/mj OR physiotherapist*:ti,ab OR 'physical therapist*':ti,ab OR ''Dentists/psychology''/exp/mj OR Dentist:ti,ab OR Dentists:ti,ab OR ''Nutritionists/psychology''/exp/mj OR Dietician*:ti,ab OR Dietitian*:ti,ab OR

		Nutritionist*:ti,ab OR Podiatry/exp/mj OR Podiatrist*:ti,ab OR Orthotist*:ti,ab OR 'Occupational Therapists/psychology'/exp/mj OR 'occupational therapist*':ti,ab OR 'Speech pathologist*':ti,ab OR 'Speech-Language Pathology'/exp/mj OR 'Speech-language pathologist*':ti,ab OR "Psychology/psychology"/exp/mj OR psychologist*:ti,ab OR "Physicians/psychology"/exp/mj OR 'Medical Practitioner*':ti,ab OR Doctor*:ti,ab OR 'medical physician*':ti,ab OR chiropractor*:ti,ab OR 'general practitioner*':ti,ab OR "Nurses/psychology"/exp/mj OR 'Nursing Staff/psychology'/exp/mj OR nurse:ti,ab OR nurse:ti,ab OR Midwifery/exp/mj OR midwi*:ti,ab))		
**ProQuest Central**	09/09/2022	(MJMESH.EXACT(Personality) OR TI,AB(Personalit*) OR MJMESH.EXACT("Personality Tests") OR TI,AB("Personality Test") OR TI,AB("personality tests") OR TI,AB("personality inventory") OR MJMESH.EXACT("Personality Inventory") OR TI,AB("Personality Inventories") OR TI,AB("behavior style*") OR TI,AB("behaviour style*") OR TI,AB("behaviour profile*") OR TI,AB("behavior profile*") OR TI,AB("behaviour pattern*") OR TI,AB("behavior pattern*") OR TI,AB("behavioral style*") OR TI,AB("behavioural style*") OR TI,AB("behavioural profile*") OR TI,AB("behavioral profile*") OR TI,AB("behavioural pattern*") OR TI,AB("behavioral pattern*") OR	Title and abstract<br>1980 -<br>English<br>Full text<br>Peer reviewed<br>Scholarly Journals	1927

	TI,AB("preferred behaviour*") OR TI,AB("preferred behavior") OR TI,AB("preferred behavior*") OR TI,AB("human behaviour*") OR TI,AB("human behavior") OR TI,AB("human behavior*") OR TI,AB("behaviour state*") OR TI,AB("behavior state*") OR TI,AB("behaviour trait*") OR TI,AB("behavior trait") OR TI,AB("behavior trait*") OR TI,AB("behaviour tendenc*") OR TI,AB("behavior tendenc*") OR TI,AB("behavioural tendenc*") OR TI,AB("behavioral tendenc*") OR TI,AB("observed behaviour*") OR TI,AB("observed behavior*") OR TI,AB("behaviour attribute*") OR TI,AB("behavior attribute*") OR TI,AB("Emotional Intelligence*") OR MJMESH.EXACT("Emotional Intelligence") OR TI,AB("emotional intellect*") OR TI,AB("emotional competence") OR TI,AB("emotional competen*") OR (TI,AB("DiSC[tiab] AND Profile")) OR (TI,AB("DiSC[tiab] AND Personalit*")) OR (TI,AB("DiSC[tiab] AND Behavio*")) OR (TI,AB("DiSC[tiab] AND Trait*")) OR TI,AB("Five factor model*") OR TI,AB("5 factor model*") OR TI,AB("myers briggs*") OR TI,AB(myers-briggs*) OR TI,AB("temperament and character inventory") OR TI,AB("Eysenck personality questionnaire") OR TI,AB("NEO five factor inventory of personality") OR TI,AB("trait emotional intelligence") OR TI,AB("hamburg personality inventory") OR TI,AB("emotional quotient	Article

inventory") OR TI,AB("emotional intelligence scale") OR TI,AB(extroversion) OR TI,AB(introversion) OR TI,AB(agreeableness) OR TI,AB(openness) OR TI,AB(neuroticism) OR TI,AB(conscientiousness)) AND (MJMESH.EXACT("Allied Health Personnel/psychology") OR TI,AB("allied health personnel") OR TI,AB("Allied Health Professional") OR TI,AB("Allied Health Professionals") OR TI,AB("Healthcare Assistant") OR TI,AB("Healthcare Assistants") OR TI,AB("Healthcare Support Worker") OR TI,AB("Healthcare Support Workers") OR TI,AB(Paramedic) OR TI,AB("Paramedical Personnel") OR TI,AB(Paramedics) OR MJMESH.EXACT("Physical Therapists/psychology") OR TI,AB(physiotherapist*) OR TI,AB(("physical therapist" OR "physical therapists")) OR MJMESH.EXACT("Dentists/psychology") OR TI,AB(Dentist) OR TI,AB(Dentists) OR MJMESH.EXACT("Nutritionists/psychology") OR TI,AB(Dietician*) OR TI,AB(Dietitian*) OR TI,AB(Nutritionist*) OR MJMESH.EXACT(Podiatry) OR TI,AB(Podiatrist*) OR TI,AB(Orthotist*) OR MJMESH.EXACT("Occupational Therapists/psychology") OR TI,AB(("occupational therapist" OR "occupational therapists")) OR TI,AB(("speech pathologist" OR "speech pathologists")) OR MJMESH.EXACT("Speech-Language Pathology") OR TI,AB("Speech-language

	pathologist*") OR MJMESH.EXACT("Psychology/psychology") OR TI,AB(psychologist*) OR MJMESH.EXACT("Physicians/psychology") OR TI,AB(("medical practitioner" OR "medical practitioners")) OR TI,AB(Doctor*) OR TI,AB(("medical physician" OR "medical physicians")) OR TI,AB(chiropractor*) OR TI,AB(("general practitioner" OR "general practitioners")) OR MJMESH.EXACT("Nurses/psychology") OR MJMESH.EXACT("Nursing Staff/psychology") OR TI,AB(nurse) OR TI,AB(nurse) OR MJMESH.EXACT(Midwifery) OR TI,AB(midwi*))	
**Hand Added Articles**	Articles known to warrant full text review located through previous literature searches, that was not picked up in search strategies above.	215

[a] *Filters utilised were selected based on the available filters for each database. The authors attempted to maintain consistency across all filters selected where able.*
