# Supplementary material for: Profiling health professionals’ personality traits, behaviour styles and emotional intelligence: a systematic review
Source: BMC Med Educ. 2023 Feb 18;23:120. doi: 10.1186/s12909-023-04003-y (PMC9938999; doi:10.1186/s12909-023-04003-y)
Supplement: Supplementary file 3 — Additional file 3. Personality, Behaviour, and Emotional Intelligence subscale/category and tools used to measure each function. [file 12909_2023_4003_MOESM3_ESM.pdf]

Additional file 3. Personality, Behaviour, and Emotional Intelligence subscale/category and tools used to measure each function.

Subscale/ Category	Tool	Author (Year)
**Personality**		
Abasement	Edwards Personal Preference Schedule (EPPS)	Wright (1993a)
Ability to cope and tolerance of negative emotion	Resiliency Assessment Scale	Marcisz-Dyla (2022)
Abstractedness	16F Personality Indicator	Ayuso-Murillo (2017)
	Cattell's Sixteen Personality Factor (16PF - 4)	Zhang (2013)
	Cattell's Sixteen Personality Factor Form C (16PF - C)	Reeve (1980)
Achievement	Edwards Personal Preference Schedule (EPPS)	Wright (1993a)
Achievement via conformance	California Psychological Inventory	Bry (1980)<br>Coombs (1993)
Achievement via independence	California Psychological Inventory	Bry (1980)<br>Coombs (1993)
Actions	NEO Personality Inventory - 3 (NEO-PI-3)	Kennedy (2014)

Activity	NEO Personality Inventory - 3 (NEO-PI-3)	Kennedy (2014)
Adjustment	Hogan Personality Inventory (HPI)	Kovach (2010)
Affiliation	Edwards Personal Preference Schedule (EPPS)	Wright (1993a)
Aggression	Edwards Personal Preference Schedule (EPPS)	Wright (1993a)
	Karolinska Scales of Personality (KSP)	Pålsson (1996)
Agreeableness	10-Item Big Five Inventory (BFI-10)	Greinacher (2022)
	Big Five Inventory - Short Form (BFI-SF)	Takase (2018b)

	Big Five Inventory (BFI)	Bagley (2018)<br>Barr (2018a)<br>Barr (2018b)<br>Buining (2015)<br>Drach-Zahavy (2019)<br>Drosdeck (2015)<br>Iorga (2016)<br>Iorga (2017)<br>Iorga (2020)<br>Kisten (2018)<br>Kooijman (2019)<br>Martos Martinez (2021)<br>Rosenthal (2015)<br>Scheepers (2016)<br>Uguz (2022)<br>vanderWal (2016)<br>Yazdanian (2016)
	Big Five Inventory (BFI) - Sum score	Duschek (2020)<br>Hudek-Knezević (2011)<br>Khanjankhani (2017)<br>Kiani (2020)<br>Yeh (2016)
	Big Five Personality Scale (BFPS)	Altuntaş (2022)

	Chinese Big Five Personality Inventory Brief Version (CBF-PI-B)	Wan (2019)
	Five Factor Model of personality (FFM)	Mullola (2018)<br>Prins (2019)
	Five-Factor Inventory	Chen (2016)
	Five-Factor Personality Inventory (FFPI)	Erdenk (2017)
	HEXACO Personality Inventory-Revised (HEXACO-PI-R)	Winters (2019)
	International Personality Item Pool (IPIP)	Lalonde (2017)
	Mini-markers of the Big Five Factor Structure personality scale	Krasner (2009)

	NEO Five-Factor Inventory (NEO FFI) - 5 point scale	Ang (2016)<br>De la Fuente-Solana (2021a)<br>De la Fuente-Solana (2021b)<br>Fatima (2008)<br>Gallardo (2018)<br>Gómez-Polo (2021)<br>Jones (2012)<br>Kheirkhah (2018)<br>Lydon (2015)<br>Membrive-Jiménez (2022)<br>Molavynejad (2019)<br>Ortega-Campos (2019)
	NEO Five-Factor Inventory (NEO FFI) - original	Hansen (1995)
	NEO Personality Inventory - 3 (NEO-PI-3)	Kennedy (2014)
	NEO Personality Inventory Revised (NEO-PI-R)	Chen (2018)<br>Geuens (2017)<br>Hojat (1999)<br>Joffe (2022)<br>Kyaw (2022)<br>Magee (1998)

		Van Mol (2018)
	NEO-Five Factor Inventory-Short Form (NEO-FFI-SF)	Froutan (2018)
	Revised Short-Form Personality 5-Factor Model	Kim (2016)
	Ten-Item Personality Inventory - Japanese (TIPI-J)	Okumura (2022)
	Ten-Item Personality Inventory (TIPI)	Bogacheva (2019)<br>Mahoney (2020)<br>Surbeck (2020)<br>Williams (2009)
	NEO Five-Factor Inventory (NEO FFI) - sten score	Cybukska (2022)<br>Kotus (2021)<br>Kwarta (2016)
	NEO Personality Inventory - 240 items	Masmouei (2020)
Alienation self	Hardiness	Rich (1987)<br>Topf (1989)

Alienation work	Hardiness	Rich (1987)<br>Topf (1989)
Altruism	NEO Personality Inventory - 3 (NEO-PI-3)	Kennedy (2014)
Ambition	Hogan Personality Inventory (HPI)	Kovach (2010)
	Personality Styles and Disorder Inventory (PSDI)	Wolf (2022)
Anger Control	Trait Anger–Anger Expression Scales	Kaya (2018)
Anger-In	Trait Anger–Anger Expression Scales	Kaya (2018)
Anger-Out	Trait Anger–Anger Expression Scales	Kaya (2018)
Anxiety	16F Personality Indicator	Amenta (1984)<br>Ayuso-Murillo (2017)
	Hamburg Personality Inventroy (HPI-K84)	Pajonk (2012)
Apprehension	16F Personality Indicator	Ayuso-Murillo (2017)
	Cattell's Sixteen Personality Factor (16PF - 4)	Zhang (2013)
	Cattell's Sixteen Personality Factor Form C (16PF - C)	Reeve (1980)
Artistic	Big Five Inventory (BFI) - Openness subscales	Chang (2016)

	John Holland Personality Test	Ahmadpanah (2015)
Ascendency	Gordon Personal Profile Index (GPPI)	Cordina (2012)
Aesthetics	NEO Personality Inventory - 3 (NEO-PI-3)	Kennedy (2014)
Assertive	Personality Styles and Disorder Inventory (PSDI)	Wolf (2022)
Assertiveness	16F Personality Indicator	Amenta (1984)
	NEO Personality Inventory - 3 (NEO-PI-3)	Kennedy (2014)
Attachment/Concern About Separation	Sociotropy– autonomy Scale (SAS)	Kaya (2018)
Autonomy	Edwards Personal Preference Schedule (EPPS)	Wright (1993a)
	Sociotropy– autonomy Scale (SAS)	Engin (2009)<br>Kaya (2018)
Balance intro/extroversion	Myers-Briggs Type Indicator (MBTI)	Bean (1995)
	Personal Style Inventory (PSI)	Bean (1993)
Balanced perceiving/judging	Myers-Briggs Type Indicator (MBTI)	Bean (1995)
	Personal Style Inventory (PSI)	Bean (1993)
Balanced sensing/intuition	Myers-Briggs Type Indicator (MBTI)	Bean (1995)

	Personal Style Inventory (PSI)	Bean (1993)
Balanced thinking/feeling	Myers-Briggs Type Indicator (MBTI)	Bean (1995)
	Personal Style Inventory (PSI)	Bean (1993)
BAS Drive	Behavior Inhibitory System/Behavior Approach System (BIS/BAS)	Symer (2018)
BAS Fun Seeking	Behavior Inhibitory System/Behavior Approach System (BIS/BAS)	Symer (2018)
BAS Reward Response	Behavior Inhibitory System/Behavior Approach System (BIS/BAS)	Symer (2018)
BIS	Behavior Inhibitory System/Behavior Approach System (BIS/BAS)	Symer (2018)
Boldness	16F Personality Indicator	Ayuso-Murillo (2017)
Capacity for status	California Psychological Inventory	Bry (1980)<br>Coombs (1993)
Cautiousness	Gordon Personal Profile Index (GPPI)	Cordina (2012)
Challenge	Hardiness Scale (HS)	VanServellen (1994)
	Third generation personal views (Hardiness) questionnaire	Tierney (1997)

Change	Edwards Personal Preference Schedule (EPPS)	Wright (1993a)
Charming	Personality Styles and Disorder Inventory (PSDI)	Wolf (2022)
Cohereance	Sense of Cohereance	Buddeberg-Fischer (2008)<br>Lewis (1994)
Commitment	Hardiness Scale (HS)	VanServellen (1994)
	Third generation personal views (Hardiness) questionnaire	Tierney (1997)
Communality	California Psychological Inventory	Bry (1980)<br>Coombs (1993)
Compassionate	Myers-Briggs Type Indicator (MBTI)	Meesusen (2010)
Compliance	NEO Personality Inventory - 3 (NEO-PI-3)	Kennedy (2014)
Concern About Disapproval	Sociotropy– autonomy Scale (SAS)	Kaya (2018)
Conformity	PROSCAN	Eastburg (1991)
Conscientiousness	10-Item Big Five Inventory (BFI-10)	Greinacher (2022)
	16F Personality Indicator	Amenta (1984)
	Big Five Inventory - Short Form (BFI-SF)	Takase (2018a)<br>Takase (2018b)

	Big Five Inventory (BFI)	Bagley (2018)<br>Barr (2018a)<br>Barr (2018b)<br>Buining (2015)<br>Donato (2017)<br>Drach-Zahavy (2019)<br>Drosdeck (2015)<br>Iorga (2016)<br>Iorga (2017)<br>Iorga (2020)<br>Kisten (2018)<br>Kooijman (2019)<br>Martos Martinez (2021)<br>Rosenthal (2015)<br>Scheepers (2016)<br>Uguz (2022)<br>vanderWal (2016)<br>Yazdanian (2016)
	Big Five Inventory (BFI) - Sum score	Duschek (2020)<br>Hudek-Knezević (2011)<br>Khanjankhani (2017)<br>Kiani (2020)<br>Yeh (2016)

	Chinese Big Five Personality Inventory Brief Version (CBF-PI-B)	Wan (2019)
	Five Factor Model of personality (FFM)	Mullola (2018)<br>Prins (2019)
	Five-Factor Inventory	Chen (2016)
	Five-Factor Personality Inventory (FFPI)	Erdenk (2017)
	HEXACO Personality Inventory-Revised (HEXACO-PI-R)	Winters (2019)
	International Personality Item Pool (IPIP)	Lalonde (2017)
	Mini-markers of the Big Five Factor Structure personality scale	Krasner (2009)

	NEO Five-Factor Inventory (NEO FFI) - 5 point scale	Ang (2016)<br>De la Fuente-Solana (2021a)<br>De la Fuente-Solana (2021b)<br>Ellershaw (2016)<br>Fatima (2008)<br>Gallardo (2018)<br>Gómez-Polo (2021)<br>Jones (2012)<br>Kheirkhah (2018)<br>Lydon (2015)<br>Membrive-Jiménez (2022)<br>Molavynejad (2019)<br>Ortega-Campos (2019)
	NEO Five-Factor Inventory (NEO FFI) - original	Hansen (1995)
	NEO Personality Inventory Revised (NEO-PI-R)	Chen (2018)<br>Geuens (2017)<br>Hojat (1999)<br>Joffe (2022)<br>Kyaw (2022)<br>Magee (1998)<br>Van Mol (2018)

	NEO-Five Factor Inventory-Short Form (NEO-FFI-SF)	Froutan (2018)
	Personality Styles and Disorder Inventory (PSDI)	Wolf (2022)
	Revised Short-Form Personality 5-Factor Model	Kim (2016)
	Ten-Item Personality Inventory - Japanese (TIPI-J)	Okumura (2022)
	Ten-Item Personality Inventory (TIPI)	Mahoney (2020)<br>Surbeck (2020)<br>Williams (2009)
	NEO Five-Factor Inventory (NEO FFI) - sten score	Cybukska (2022)<br>Kotus (2021)<br>Kwarta (2016)
	NEO Personality Inventory - 240 items	Masmouei (2020)
Control	16F Personality Indicator	Amenta (1984)
	Basic Character Inventory (BCI)	Myhren (2013)
	Eysenck Personality Questionnaire - Revised (EPQ-R)	Al-Alawi (2017)
	Hardiness Scale (HS)	VanServellen (1994)
	Third generation personal views (Hardiness) questionnaire	Tierney (1997)
Conventional	John Holland Personality Test	Ahmadpanah (2015)

Cooperativeness	Temperament and Character Inventory (TCI-240)	Yildirim (2012)
	Temperament and Character Inventory (TCI-R 140)	Ball (2015)<br>Ball (2016)<br>Campbell (2013)<br>Campbell (2014)<br>Eley (2009)<br>Eley (2011a)<br>Eley (2011b)<br>Eley (2012)<br>Eley (2013)
	Temperament and Character Inventory (TCI-R 140) - mean score	Eley (2015)<br>Laurence (2016)
Creative	Big Five Inventory (BFI) - Openness subscales	Chang (2016)
Critical	Personality Styles and Disorder Inventory (PSDI)	Wolf (2022)
Cynicism	Minnesota Multiphasic Personality Inventory (MMPI)	McCranie (1989)
Deep	Big Five Inventory (BFI) - Openness subscales	Chang (2016)
Deference	Edwards Personal Preference Schedule (EPPS)	Wright (1993a)

Depressivity	Freiburg Personality Inventory (FPI)	Bergmueller (2018)
Detachment	Karolinska Scales of Personality (KSP)	Pålsson (1996)
Dogmatism	Rokeach Dogmatism Scale (D-20)	DiRenzo et al (1981)
Dominance	16F Personality Indicator	Ayuso-Murillo (2017)
	California Psychological Inventory	Bry (1980)<br>Coombs (1993)
	Cattell's Sixteen Personality Factor (16PF - 4)	Zhang (2013)
	Cattell's Sixteen Personality Factor Form C (16PF - C)	Reeve (1980)
	Edwards Personal Preference Schedule (EPPS)	Wright (1993a)
	PROSCAN	Eastburg (1991)
Easy going	Myers-Briggs Type Indicator (MBTI)	Meesusen (2010)
Ego strength	16F Personality Indicator	Amenta (1984)
Emotional awareness of others	Genos 31-item questionnaire	Al-Hamdan (2019)
Emotional disability	Five-Factor Personality Inventory (FFPI)	Erdenk (2017)
Emotional exhaustion	Big Five Inventory (BFI)	Scheepers (2016)
Emotional expression	Genos 31-item questionnaire	Al-Hamdan (2019)

Emotional lability	Freiburg Personality Inventory (FPI)	Bergmueller (2018)
Emotional management of others	Genos 31-item questionnaire	Al-Hamdan (2019)
Emotional reasoning	Genos 31-item questionnaire	Al-Hamdan (2019)
Emotional self-management	Genos 31-item questionnaire	Al-Hamdan (2019)
Emotional stability	Cattell's Sixteen Personality Factor (16PF - 4)	Zhang (2013)
	Cattell's Sixteen Personality Factor Form C (16PF - C)	Reeve (1980)
	Core Self-Evaluations Scale (CSES)	Farčić (2020)
	Five-Factor Inventory	Chen (2016)
	Gordon Personal Profile Index (GPPI)	Cordina (2012)
	International Personality Item Pool (IPIP)	Lalonde (2017)
	Mini-markers of the Big Five Factor Structure personality scale	Krasner (2009)
	NEO Personality Inventory Revised (NEO-PI-R)	Chen (2018)
	Ten-Item Personality Inventory (TIPI)	Mahoney (2020)<br>Williams (2009)

Emotionality	HEXACO Personality Inventory-Revised (HEXACO-PI-R)	Winters (2019)
Empathy	California Psychological Inventory	Dumitru (2012)
Endurance	Edwards Personal Preference Schedule (EPPS)	Wright (1993a)
ENFJ	Kiersey-Bates Personality Inventory	Lysack (2001)
	Myers-Briggs Type Indicator (MBTI)	Al-Dlaigan (2017)<br>Grandy (1996)<br>Hagan (1999)<br>Sladek (2010)<br>Talha (2022)
ENFJ	Myers-Briggs Type Indicator (MBTI)	Bean (1995)
ENFP	Kiersey-Bates Personality Inventory	Lysack (2001)
	Myers-Briggs Type Indicator (MBTI)	Al-Dlaigan (2017)<br>Grandy (1996)<br>Hagan (1999)<br>Needleman (2011)<br>Sladek (2010)<br>Talha (2022)
	Personal Style Inventory (PSI)	Bean (1993)

ENFP	Myers-Briggs Type Indicator (MBTI)	Bean (1995)
ENJF	Myers-Briggs Type Indicator (MBTI)	Needleman (2011)
Enterprising	John Holland Personality Test	Ahmadpanah (2015)
ENTJ	Kiersey-Bates Personality Inventory	Lysack (2001)
	Myers-Briggs Type Indicator (MBTI)	Al-Dlaigan (2017)<br>Grandy (1996)<br>Hagan (1999)<br>Radonsky (1980)<br>Sladek (2010)
ENTJ	Myers-Briggs Type Indicator (MBTI)	Bean (1995)
ENTP	Myers-Briggs Type Indicator (MBTI)	Al-Dlaigan (2017)<br>Grandy (1996)<br>Hagan (1999)<br>Sladek (2010)<br>Talha (2022)
ESFJ	Kiersey-Bates Personality Inventory	Lysack (2001)

	Myers-Briggs Type Indicator (MBTI)	Grandy (1996)<br>Hagan (1999)<br>Needleman (2011)<br>Sladek (2010)<br>Talha (2022)
	Personal Style Inventory (PSI)	Bean (1993)
ESFJ	Myers-Briggs Type Indicator (MBTI)	Bean (1995)
ESFP	Kiersey-Bates Personality Inventory	Lysack (2001)
	Myers-Briggs Type Indicator (MBTI)	Grandy (1996)<br>Hagan (1999)<br>Sladek (2010)<br>Talha (2022)
	Personal Style Inventory (PSI)	Bean (1993)
ESTJ	Kiersey-Bates Personality Inventory	Lysack (2001)
	Myers-Briggs Type Indicator (MBTI)	Grandy (1996)<br>Hagan (1999)<br>Needleman (2011)<br>Sladek (2010)<br>Sladek 2010
	Personal Style Inventory (PSI)	Bean (1993)

ESTJ	Myers-Briggs Type Indicator (MBTI)	Bean (1995)
ESTP	Kiersey-Bates Personality Inventory	Lysack (2001)
	Myers-Briggs Type Indicator (MBTI)	Grandy (1996)<br>Hagan (1999)<br>Sladek (2010)<br>Talha (2022)
ESTY	Myers-Briggs Type Indicator (MBTI)	Talha (2022)
Excitement-seeking	NEO Personality Inventory - 3 (NEO-PI-3)	Kennedy (2014)
Exhibition	Edwards Personal Preference Schedule (EPPS)	Wright (1993a)
Expressiveness	16F Personality Indicator	Amenta (1984)
	Personal Attribute Questionnaire (PAQ)	Buddeberg-Fischer (2008)
Extraversion	10-Item Big Five Inventory (BFI-10)	Greinacher (2022)
	16F Personality Indicator	Amenta (1984)
		Ayuso-Murillo (2017)
	Basic Character Inventory (BCI)	Myhren (2013)
	Big Five Inventory - Short Form (BFI-SF)	Takase (2018a)<br>Takase (2018b)

	Big Five Inventory (BFI)	Bagley (2018)<br>Barr (2018a)<br>Barr (2018b)<br>Buining (2015)<br>Drach-Zahavy (2019)<br>Drosdeck (2015)<br>Iorga (2016)<br>Iorga (2017)<br>Iorga (2020)<br>Kisten (2018)<br>Kooijman (2019)<br>Martos Martinez (2021)<br>Rosenthal (2015)<br>Scheepers (2016)<br>Uguz (2022)<br>vanderWal (2016)<br>Yazdanian (2016)
	Big Five Inventory (BFI) - Sum score	Duschek (2020)<br>Hudek-Knezević (2011)<br>Khanjankhani (2017)<br>Kiani (2020)<br>Yeh (2016)
	Big Five Personality Scale (BFPS)	Altuntaş (2022)

	Chinese Big Five Personality Inventory Brief Version (CBF-PI-B)	Wan (2019)
	Eysenck Personality Questionnaire	Farzianpour (2015)<br>Ntantana (2017)<br>Rolander (2008)
	Eysenck Personality Questionnaire - Revised (EPQ-R)	Al-Alawi (2017)
	Eysenck Personality Questionnaire - Revised Short Scale (EPQ-RS)	Li (2014)<br>Głębocka (2019)<br>McCulloch (2005)<br>Nash (2009)<br>Shimizutani (2008)
	Five Factor Model of personality (FFM)	Mullola (2018)<br>Prins (2019)
	Five-Factor Inventory	Chen (2016)
	Five-Factor Personality Inventory (FFPI)	Erdenk (2017)
	HEXACO Personality Inventory-Revised (HEXACO-PI-R)	Winters (2019)
	International Personality Item Pool (IPIP)	Anitha (2022)
	Mini-markers of the Big Five Factor Structure personality scale	Krasner (2009)

	Minnesota Multiphasic Personality Inventory (MMPI)	McCranie (1989)
	Myers-Briggs Type Indicator (MBTI)	Al-Dlaigan (2017)<br>Bean (1995)<br>Chang (2019)<br>Clack (2004)<br>Cross (1984)<br>Grandy (1996)<br>Harris (1985)<br>Lewis (1994)<br>Needleman (2011)<br>Whitworth (2005)

	NEO Five-Factor Inventory (NEO FFI) - 5 point scale	Albendín-García (2022)<br>Ang (2016)<br>De la Fuente-Solana (2019)<br>De la Fuente-Solana (2021a)<br>De la Fuente-Solana (2021b)<br>Ellershaw (2016)<br>Fatima (2008)<br>Gallardo (2018)<br>Gómez-Polo (2021)<br>Jones (2012)<br>Kheirkhah (2018)<br>Lydon (2015)<br>Membrive-Jiménez (2022)<br>Molavynejad (2019)<br>Ortega-Campos (2019)
	NEO Five-Factor Inventory (NEO FFI) - original	Hansen (1995)
	NEO Personality Inventory - 3 (NEO-PI-3)	Kennedy (2014)

	NEO Personality Inventory Revised (NEO-PI-R)	Chen (2018)<br>Geuens (2017)<br>Hojat (1999)<br>Joffe (2022)<br>Kyaw (2022)<br>Magee (1998)<br>Van Mol (2018)
	NEO-Five Factor Inventory-Short Form (NEO-FFI-SF)	Froutan (2018)
	Personal Style Inventory (PSI)	Bean (1993)
	Personality-trait questionnaire	Zaid (2022)
	PROSCAN	Eastburg (1991)
	Revised Short-Form Personality 5-Factor Model	Kim (2016)
	Ten-Item Personality Inventory - Japanese (TIPI-J)	Okumura (2022)
	Ten-Item Personality Inventory (TIPI)	Mahoney (2020)<br>Surbeck (2020)<br>Williams (2009)
	NEO Five-Factor Inventory (NEO FFI) - sten score	Cybukska (2022)<br>Kotus (2021)<br>Kwarta (2016)
	NEO Personality Inventory - 240 items	Masmouei (2020)

Extraversion/introversion	Freiburg Personality Inventory (FPI)	Bergmueller (2018)
	Myers-Briggs Type Indicator (MBTI)	Amenta (1984)
Extrinsic importance	The Aspiration Index	Montasem (2014)
Extrinsic likelihood	The Aspiration Index	Montasem (2014)
Family cohersion	Resilence Scale for Adults	Kutluturkan (2016)
Fantasy	NEO Personality Inventory - 3 (NEO-PI-3)	Kennedy (2014)
Feeling	Myers-Briggs Type Indicator (MBTI)	Al-Dlaigan (2017)
		Bean (1995)
		Chang (2019)
		Clack (2004)
		Cross (1984)
		Grandy (1996)
		Harris (1985)
		Lewis (1994)
		Needleman (2011)
		Whitworth (2005)
	Personal Style Inventory (PSI)	Bean (1993)
	NEO Personality Inventory - 3 (NEO-PI-3)	Kennedy (2014)

Femininity	California Psychological Inventory	Bry (1980)<br>Coombs (1993)<br>Dumitru (2012)
Flexibility	California Psychological Inventory	Bry (1980)<br>Coombs (1993)
Forthrightness	16F Personality Indicator	Amenta (1984)
Friendliness	NEO Five-Factor Inventory (NEO FFI) - 5 point scale	Albendín-García (2022)<br>De la Fuente-Solana (2019)
Good impression	California Psychological Inventory	Bry (1980)<br>Coombs (1993)<br>Dumitru (2012)
Gregariousness	NEO Personality Inventory - 3 (NEO-PI-3)	Kennedy (2014)
Hardiness	Abridged Hardiness Scale	McCranie (1987)
	Dispositional Resilience (hardiness) Scale-Revised (DRS15R)	Saksvik-Lehouillier (2012)<br>Saksvik-Lehouillier (2016)
	Hardiness of Personality Inventory (HPI)	Langemo (1990)
	Hardiness Scale (HS)	Judkins (2006)
		VanServellen (1994)
	Hardiness Test (HT)	DePew (1999)<br>Wright (1993b)
	The Short Hardiness Inventory	Bagley (2018)

	Third generation personal views (Hardiness) questionnaire	Tierney (1997)
Harm avoidance	Temperament and Character Inventory (TCI-240)	Yildirim (2012)
	Temperament and Character Inventory (TCI-R 140)	Ball (2015)<br>Ball (2016)<br>Campbell (2013)<br>Campbell (2014)<br>Eley (2009)<br>Eley (2011a)<br>Eley (2011b)<br>Eley (2012)<br>Eley (2013)
	Temperament and Character Inventory (TCI-R 140) - mean score	Eley (2015)<br>Laurence (2016)
Heterosexuality	Edwards Personal Preference Schedule (EPPS)	Wright (1993a)
Honesty/humility	HEXACO Personality Inventory-Revised (HEXACO-PI-R)	Winters (2019)
Hostility	Karolinska Scales of Personality (KSP)	Pålsson (1996)
Ideas	NEO Personality Inventory - 3 (NEO-PI-3)	Kennedy (2014)
Imagination	16F Personality Indicator	Amenta (1984)

Impulsiveness	Karolinska Scales of Personality (KSP)	Pålsson (1996)
Inadequacy	Minnesota Multiphasic Personality Inventory (MMPI)	McCranie (1989)
Independence	16F Personality Indicator	Amenta (1984)<br>Ayuso-Murillo (2017)
	California Psychological Inventory	Dumitru (2012)
Individualistic or Autonomous Achievement	Sociotropy– autonomy Scale (SAS)	Kaya (2018)
INFJ	Kiersey-Bates Personality Inventory	Lysack (2001)
	Myers-Briggs Type Indicator (MBTI)	Al-Dlaigan (2017)<br>Grandy (1996)<br>Sladek (2010)<br>Talha (2022)
	Personal Style Inventory (PSI)	Bean (1993)
INFJ	Myers-Briggs Type Indicator (MBTI)	Bean (1995)<br>Hagan (1999)
INFP	Kiersey-Bates Personality Inventory	Lysack (2001)

	Myers-Briggs Type Indicator (MBTI)	Al-Dlaigan (2017)<br>Grandy (1996)<br>Sladek (2010)<br>Talha (2022)
	Personal Style Inventory (PSI)	Bean (1993)
INFP	Myers-Briggs Type Indicator (MBTI)	Hagan (1999)
Inhibition of aggression	Karolinska Scales of Personality (KSP)	Pålsson (1996)
Instrumentality	Personal Attribute Questionnaire (PAQ)	Buddeberg-Fischer (2008)
Intelligence	Big Five Inventory (BFI) - Openness subscales	Chang (2016)
	Hogan Personality Inventory (HPI)	Kovach (2010)
	Big Five Inventory (BFI) - Openness subscales	Chang (2016)
	16F Personality Indicator	Amenta (1984)
Intellectual efficiency	California Psychological Inventory	Bry (1980)<br>Coombs (1993)<br>Dumitru (2012)
Intellectual interest	Minnesota Multiphasic Personality Inventory (MMPI)	McCranie (1989)
INTJ	Kiersey-Bates Personality Inventory	Lysack (2001)

	Myers-Briggs Type Indicator (MBTI)	Grandy (1996)<br>Sladek (2010)<br>Talha (2022)
INTJ	Myers-Briggs Type Indicator (MBTI)	Hagan (1999)
Intolerance for ambiguity	Ten-Item Personality Inventory (TIPI)	Bogacheva (2019)
INTP	Kiersey-Bates Personality Inventory	Lysack (2001)
	Myers-Briggs Type Indicator (MBTI)	Al-Dlaigan (2017)<br>Grandy (1996)<br>Hagan (1999)<br>Talha (2022)
Intraception	Edwards Personal Preference Schedule (EPPS)	Wright (1993a)
Intrinsic importance	The Aspiration Index	Montasem (2014)
Intrinsic likelihood	The Aspiration Index	Montasem (2014)
Introversion	Eysenck Personality Questionnaire	Farzianpour (2015)

	Myers-Briggs Type Indicator (MBTI)	Al-Dlaigan (2017)<br>Bean (1995)<br>Chang (2019)<br>Clack (2004)<br>Cross (1984)<br>Grandy (1996)<br>Harris (1985)<br>Lewis (1994)<br>Needleman (2011)<br>Whitworth (2005)
	Personal Style Inventory (PSI)	Bean (1993)
Intuition	Myers-Briggs Type Indicator (MBTI)	Al-Dlaigan (2017)<br>Bean (1995)<br>Chang (2019)<br>Clack (2004)<br>Cross (1984)<br>Grandy (1996)<br>Harris (1985)<br>Lewis (1994)<br>Needleman (2011)<br>Whitworth (2005)
	Personal Style Inventory (PSI)	Bean (1993)
	Personality Styles and Disorder Inventory (PSDI)	Wolf (2022)

Intuition/feeling	Kiersey Temperament Sorter	McPhail (2002)
	Kiersey-Bates Personality Inventory	Lysack (2001)
	Myers-Briggs Type Indicator (MBTI)	Clack (2004)<br>Grandy (1996)
Intuition/thinking	Kiersey Temperament Sorter	McPhail (2002)
	Kiersey-Bates Personality Inventory	Lysack (2001)
	Myers-Briggs Type Indicator (MBTI)	Clack (2004)<br>Grandy (1996)
Investigative	John Holland Personality Test	Ahmadpanah (2015)
Irritability	Freiburg Personality Inventory (FPI)	Bergmueller (2018)
ISFJ	Kiersey-Bates Personality Inventory	Lysack (2001)
	Myers-Briggs Type Indicator (MBTI)	Al-Dlaigan (2017)<br>Grandy (1996)<br>Needleman (2011)<br>Radonsky (1980)<br>Sladek (2010)<br>Talha (2022)
	Personal Style Inventory (PSI)	Bean (1993)
ISFJ	Myers-Briggs Type Indicator (MBTI)	Bean (1995)<br>Hagan (1999)

ISFP	Kiersey-Bates Personality Inventory	Lysack (2001)
	Myers-Briggs Type Indicator (MBTI)	Grandy (1996)<br>Sladek (2010)<br>Talha (2022)
	Personal Style Inventory (PSI)	Bean (1993)
ISFP	Myers-Briggs Type Indicator (MBTI)	Hagan (1999)
ISTJ	Kiersey-Bates Personality Inventory	Lysack (2001)
	Myers-Briggs Type Indicator (MBTI)	Al-Dlaigan (2017)<br>Bean (1995)<br>Grandy (1996)<br>Hagan (1999)<br>Needleman (2011)<br>Sladek (2010)<br>Talha (2022)
	Personal Style Inventory (PSI)	Bean (1993)
ISTP	Kiersey-Bates Personality Inventory	Lysack (2001)
	Myers-Briggs Type Indicator (MBTI)	Al-Dlaigan (2017)<br>Grandy (1996)<br>Sladek (2010)<br>Talha (2022)

Judgement	Myers-Briggs Type Indicator (MBTI)	Al-Dlaigan (2017)<br>Chang (2019)<br>Cross (1984)<br>Harris (1985)<br>Lewis (1994)<br>Whitworth (2005)<br>Bean (1995)<br>Clack (2004)<br>Grandy (1996)<br>Needleman (2011)
	Personal Style Inventory (PSI)	Bean (1993)
Judgement/perception	Myers-Briggs Type Indicator (MBTI)	Amenta (1984)
Lie	Personality-trait questionnaire	Zaid (2022)
lie scale	Eysenck Personality Questionnaire - Revised Short Scale (EPQ-RS)	Głębocka (2019)<br>Li (2014)
Likeability	Hogan Personality Inventory (HPI)	Kovach (2010)
Liveliness	16F Personality Indicator	Ayuso-Murillo (2017)
	Cattell's Sixteen Personality Factor (16PF - 4)	Zhang (2013)
	Cattell's Sixteen Personality Factor Form C (16PF - C)	Reeve (1980)
Locus of control	Core Self-Evaluations Scale (CSES)	Farčić (2020)

	Hardiness	Rich (1987)
		Topf (1989)
Logic	PROSCAN	Eastburg (1991)
Loyal	Personality Styles and Disorder Inventory (PSDI)	Wolf (2022)
Machiavellianism	MACH-IV Test of Machiavellianism	Bucknall (2015)
Masculinity/femininity	Freiburg Personality Inventory (FPI)	Bergmueller (2018)
	Minnesota Multiphasic Personality Inventory (MMPI)	McCranie (1989)
Mental balance	Freiburg Personality Inventory (FPI)	Bergmueller (2018)
Mobility/Freedom from Control by Others	Sociotropy– autonomy Scale (SAS)	Kaya (2018)
Modesty	NEO Personality Inventory - 3 (NEO-PI-3)	Kennedy (2014)
Monotony avoidance	Karolinska Scales of Personality (KSP)	Pålsson (1996)
Muscular tension	Karolinska Scales of Personality (KSP)	Pålsson (1996)
Narcissism	Dark Triad Personality (DTP)	Ying (2018)
	Narcissistic Personality Inventory (NPI)	Bucknall (2015)

Negative	Ten-Item Personality Inventory - Chinese (TIPI-C)	Huang (2021)
Neuroticism	10-Item Big Five Inventory (BFI-10)	Greinacher (2022)
	Basic Character Inventory (BCI)	Myhren (2013)
	Big Five Inventory - Short Form (BFI-SF)	Takase (2018b)
	Big Five Inventory (BFI)	Bagley (2018)<br>Barr (2018a)<br>Barr (2018b)<br>Buining (2015)<br>Donato (2017)<br>Drach-Zahavy (2019)<br>Drosdeck (2015)<br>Iorga (2016)<br>Iorga (2017)<br>Iorga (2020)<br>Kisten (2018)<br>Kooijman (2019)<br>Lu (2022)<br>Martos Martinez (2021)<br>Rosenthal (2015)<br>Uguz (2022)<br>vanderWal (2016)<br>Yazdanian (2016)

	Big Five Inventory (BFI) - Sum score	Duschek (2020)<br>Hudek-Knezević (2011)<br>Khanjankhani (2017)<br>Kiani (2020)<br>Yeh (2016)
	Big Five Personality Scale (BFPS)	Altuntaş (2022)
	Chinese Big Five Personality Inventory Brief Version (CBF-PI-B)	Wan (2019)
	Eysenck Personality Questionnaire	IsakssonRo (2010)<br>Ntantana (2017)<br>Rolander (2008)
	Eysenck Personality Questionnaire - Revised (EPQ-R)	Al-Alawi (2017)<br>Głębocka (2019)
	Eysenck Personality Questionnaire - Revised Short Scale (EPQ-RS)	Li (2014)<br>McCulloch (2005)<br>Nash (2009)<br>Shimizutani (2008)
	Five Factor Model of personality (FFM)	Mullola (2018)<br>Prins (2019)
	Freiburg Personality Inventory (FPI)	Bergmueller (2018)
	Mini-International Personality Item Pool (Mini-IPIP)	O'Mahony (2018)

	Minnesota Multiphasic Personality Inventory (MMPI)	McCranie (1989)
	NEO Five-Factor Inventory (NEO FFI) - 5 point scale	Albendín-García (2022)<br>Ang (2016)<br>De la Fuente-Solana (2019)<br>De la Fuente-Solana (2021a)<br>De la Fuente-Solana (2021b)<br>Fatima (2008)<br>Gallardo (2018)<br>Gómez-Polo (2021)<br>Jones (2012)<br>Kheirkhah (2018)<br>Lydon (2015)<br>Membrive-Jiménez (2022)<br>Molavynejad (2019)<br>Narumoto (2008)<br>Ortega-Campos (2019)
	NEO Five-Factor Inventory (NEO FFI) - original	Hansen (1995)

	NEO Personality Inventory Revised (NEO-PI-R)	Geuens (2017)<br>Hojat (1999)<br>Joffe (2022)<br>Kyaw (2022)<br>Magee (1998)<br>Van Mol (2018)
	NEO-Five Factor Inventory-Short Form (NEO-FFI-SF)	Froutan (2018)
	Neuroticism Scale	Stovall (2021)
	Personality-trait questionnaire	Zaid (2022)
	Revised Short-Form Personality 5-Factor Model	Kim (2016)
	Ten-Item Personality Inventory - Japanese (TIPI-J)	Okumura (2022)
	Ten-Item Personality Inventory (TIPI)	Surbeck (2020)
	NEO Five-Factor Inventory (NEO FFI) - sten score	Allen (2002)<br>Cybukska (2022)<br>Kotus (2021)<br>Kwarta (2016)
	NEO Personality Inventory - 240 items	Masmouei (2020)
Normative	Ten-Item Personality Inventory - Chinese (TIPI-C)	Huang (2021)

Novelty seeking	Temperament and Character Inventory (TCI-240)	Yildirim (2012)
	Temperament and Character Inventory (TCI-R 140)	Ball (2015)<br>Ball (2016)<br>Campbell (2013)<br>Campbell (2014)<br>Eley (2009)<br>Eley (2011a)<br>Eley (2011b)<br>Eley (2012)<br>Eley (2013)
	Temperament and Character Inventory (TCI-R 140) - mean score	Eley (2015)<br>Laurence (2016)
Nurturance	Edwards Personal Preference Schedule (EPPS)	Wright (1993a)
Openness	10-Item Big Five Inventory (BFI-10)	Greinacher (2022)
	Big Five Inventory - Short Form (BFI-SF)	Takase (2018a)<br>Takase (2018b)

	Big Five Inventory (BFI)	Bagley (2018)<br>Barr (2018a)<br>Barr (2018b)<br>Buining (2015)<br>Drach-Zahavy (2019)<br>Drosdeck (2015)<br>Iorga (2016)<br>Iorga (2017)<br>Kisten (2018)<br>Kooijman (2019)<br>Martos Martinez (2021)<br>Rosenthal (2015)<br>Scheepers (2016)<br>Uguz (2022)<br>vanderWal (2016)<br>Yazdanian (2016)
	Big Five Inventory (BFI) - Sum score	Duschek (2020)<br>Hudek-Knezević (2011)<br>Khanjankhani (2017)<br>Kiani (2020)<br>Yeh (2016)
	Big Five Personality Scale (BFPS)	Altuntaş (2022)
	Cattell's Sixteen Personality Factor (16PF - 4)	Zhang (2013)

	Cattell's Sixteen Personality Factor Form C (16PF - C)	Reeve (1980)
	Chinese Big Five Personality Inventory Brief Version (CBF-PI-B)	Wan (2019)
	Five Factor Model of personality (FFM)	Mullola (2018)<br>Prins (2019)
	Five-Factor Inventory	Chen (2016)
	Five-Factor Personality Inventory (FFPI)	Erdenk (2017)
	Freiburg Personality Inventory (FPI)	Bergmueller (2018)
	HEXACO Personality Inventory-Revised (HEXACO-PI-R)	Winters (2019)
	International Personality Item Pool (IPIP)	Lalonde (2017)
	Mini-markers of the Big Five Factor Structure personality scale	Krasner (2009)

	NEO Five-Factor Inventory (NEO FFI) - 5 point scale	Albendín-García (2022)<br>Ang (2016)<br>De la Fuente-Solana (2019)<br>De la Fuente-Solana (2021a)<br>De la Fuente-Solana (2021b)<br>Ellershaw (2016)<br>Fatima (2008)<br>Gallardo (2018)<br>Gómez-Polo (2021)<br>Jones (2012)<br>Kheirkhah (2018)<br>Lydon (2015)<br>Membrive-Jiménez (2022)<br>Molavynejad (2019)<br>Ortega-Campos (2019)
	NEO Five-Factor Inventory (NEO FFI) - original	Hansen (1995)
	NEO Personality Inventory - 3 (NEO-PI-3)	Kennedy (2014)

	NEO Personality Inventory Revised (NEO-PI-R)	Chen (2018)<br>Geuens (2017)<br>Hojat (1999)<br>Joffe (2022)<br>Kyaw (2022)<br>Magee (1998)<br>Van Mol (2018)
	NEO-Five Factor Inventory-Short Form (NEO-FFI-SF)	Froutan (2018)
	Revised Short-Form Personality 5-Factor Model	Kim (2016)
	Ten-Item Personality Inventory - Japanese (TIPI-J)	Okumura (2022)
	Ten-Item Personality Inventory (TIPI)	Mahoney (2020)<br>Surbeck (2020)<br>Williams (2009)
	NEO Five-Factor Inventory (NEO FFI) - sten score	Cybukska (2022)<br>Kotus (2021)<br>Kwarta (2016)
	NEO Personality Inventory - 240 items	Masmouei (2020)
Openness and sense of humour	Resiliency Assessment Scale	Marcisz-Dyla (2022)
Openness to change	16F Personality Indicator	Ayuso-Murillo (2017)

Optimistic	Personality Styles and Disorder Inventory (PSDI)	Wolf (2022)
Optimistic and ability to mobilise one's self	Resiliency Assessment Scale	Marcisz-Dyla (2022)
Order	Edwards Personal Preference Schedule (EPPS)	Wright (1993a)
Orderly	Myers-Briggs Type Indicator (MBTI)	Meesusen (2010)
Original thinking	Gordon Personal Profile Index (GPPI)	Cordina (2012)
Pace	PROSCAN	Eastburg (1991)
Passive	Personality Styles and Disorder Inventory (PSDI)	Wolf (2022)
Perceiving	Myers-Briggs Type Indicator (MBTI)	Bean (1995)<br>Clack (2004)<br>Grandy (1996)<br>Needleman (2011)
	Personal Style Inventory (PSI)	Bean (1993)
Perception	Myers-Briggs Type Indicator (MBTI)	Al-Dlaigan (2017)<br>Chang (2019)<br>Cross (1984)<br>Harris (1985)<br>Lewis (1994)<br>Whitworth (2005)

Perception of future	Resilience Scale for Adults	Kutluturkan (2016)
Perfectionism	16F Personality Indicator	Ayuso-Murillo (2017)
	Cattell's Sixteen Personality Factor (16PF - 4)	Zhang (2013)
	Cattell's Sixteen Personality Factor Form C (16PF - C)	Reeve (1980)
Persistence	Temperament and Character Inventory (TCI-240)	Yildirim (2012)
	Temperament and Character Inventory (TCI-R 140)	Ball (2015)<br>Ball (2016)<br>Campbell (2013)<br>Campbell (2014)<br>Eley (2009)<br>Eley (2011a)<br>Eley (2011b)<br>Eley (2012)<br>Eley (2013)
	Temperament and Character Inventory (TCI-R 140) - mean score	Eley (2015)<br>Laurence (2016)
Persistence and determination	Resiliency Assessment Scale	Marcisz-Dyla (2022)
Personal relations	Gordon Personal Profile Index (GPPI)	Cordina (2012)

Philosophical	Big Five Inventory (BFI) - Openness subscales	Chang (2016)
Pleasing Others	Sociotropy– autonomy Scale (SAS)	Kaya (2018)
Poise	16F Personality Indicator	Amenta (1984)
Positive	Ten-Item Personality Inventory - Chinese (TIPI-C)	Huang (2021)
Positive emotions	NEO Personality Inventory - 3 (NEO-PI-3)	Kennedy (2014)
Powerlessness	Hardiness	Rich (1987)
Preference for Solitude	Sociotropy– autonomy Scale (SAS)	Kaya (2018)
Privateness	16F Personality Indicator	Ayuso-Murillo (2017)
Proactive personality	Proactive Coping Scale	Chang (2010)
	Proactive Personality Scale (PPS)	Erkutlu (2012)
		Farooq (2020)
		Hu (2021)
		Lv (2018)
Process	16F Personality Indicator	Amenta (1984)
Prudence	Hogan Personality Inventory (HPI)	Kovach (2010)
Psychasthenia	Karolinska Scales of Personality (KSP)	Pålsson (1996)
Psychic anxiety	Karolinska Scales of Personality (KSP)	Pålsson (1996)
Psychological-mindedness	California Psychological Inventory	Bry (1980)<br>Coombs (1993)

Psychoticism / infrequency	Minnesota Multiphasic Personality Inventory (MMPI)	McCranie (1989)
	Personality-trait questionnaire	Zaid (2022)
Psychotism	Eysenck Personality Questionnaire	Ntantana (2017)
	Eysenck Personality Questionnaire - Revised (EPQ-R)	Al-Alawi (2017)<br>Głębocka (2019)
	Eysenck Personality Questionnaire - Revised Short Scale (EPQ-RS)	Li (2014)<br>Nash (2009)
Radicalism	16F Personality Indicator	Amenta (1984)
Rationality	Ten-Item Personality Inventory (TIPI)	Bogacheva (2019)
Reactive aggressivity	Freiburg Personality Inventory (FPI)	Bergmueller (2018)
Realistic	John Holland Personality Test	Ahmadpanah (2015)
Reasoning	16F Personality Indicator	Ayuso-Murillo (2017)
	Cattell's Sixteen Personality Factor (16PF - 4)	Zhang (2013)
	Cattell's Sixteen Personality Factor Form C (16PF - C)	Reeve (1980)
Receptive	Myers-Briggs Type Indicator (MBTI)	Meesusen (2010)
Religious orthodoxy	Minnesota Multiphasic Personality Inventory (MMPI)	McCranie (1989)

Reserved	Personality Styles and Disorder Inventory (PSDI)	Wolf (2022)
Resilience	Resilence Scale for Adults	Kutluturkan (2016)
	Resiliencey Assessment Scale	Marcisz-Dyla (2022)
Responsibility	Big Five Personality Scale (BFPS)	Altuntaş (2022)
	California Psychological Inventory	Bry (1980)<br>Coombs (1993)
	Gordon Personal Profile Index (GPPI)	Cordina (2012)
	NEO Five-Factor Inventory (NEO FFI) - 5 point scale	Albendín-García (2022)<br>De la Fuente-Solana (2019)
Reward dependence	Temperament and Character Inventory (TCI-240)	Yildirim (2012)
	Temperament and Character Inventory (TCI-R 140)	Ball (2015)<br>Ball (2016)<br>Campbell (2013)<br>Campbell (2014)<br>Eley (2009)<br>Eley (2011a)<br>Eley (2011b)<br>Eley (2012)<br>Eley (2013)
	Temperament and Character Inventory (TCI-R 140) - mean score	Eley (2015)<br>Laurence (2016)

Risk-prone index	Jackson Personality Inventory-revised	Abelsen (2015)
Risk-readiness	Ten-Item Personality Inventory (TIPI)	Bogacheva (2019)
Rule-consciousness	16F Personality Indicator	Ayuso-Murillo (2017)
	Cattell's Sixteen Personality Factor (16PF - 4)	Zhang (2013)
	Cattell's Sixteen Personality Factor Form C (16PF - C)	Reeve (1980)
School success	Hogan Personality Inventory (HPI)	Kovach (2010)
Security	Hardiness	Rich (1987)
		Topf (1989)
Self-efficacy	Core Self-Evaluations Scale (CSES)	Farčić (2020)
Self-acceptance	California Psychological Inventory	Bry (1980)<br>Coombs (1993)
Self-awareness	Genos 31-item questionnaire	Al-Hamdan (2019)
Self-control	16F Personality Indicator	Ayuso-Murillo (2017)
	California Psychological Inventory	Bry (1980)
		Coombs (1993)
	Genos 31-item questionnaire	Al-Hamdan (2019)
Self-critical	Personality Styles and Disorder Inventory (PSDI)	Wolf (2022)

Self-directedness	Temperament and Character Inventory (TCI-240)	Yildirim (2012)
	Temperament and Character Inventory (TCI-R 140)	Ball (2015)<br>Ball (2016)<br>Campbell (2013)<br>Campbell (2014)<br>Eley (2009)<br>Eley (2011a)<br>Eley (2011b)<br>Eley (2012)<br>Eley (2013)
	Temperament and Character Inventory (TCI-R 140) - mean score	Eley (2015)<br>Laurence (2016)
Self-esteem	Core Self-Evaluations Scale (CSES)	Farčić (2020)
	Gordon Personal Profile Index (GPPI)	Cordina (2012)
Self-perception	Resilience Scale for Adults	Kutluturkan (2016)
Self-reliance	16F Personality Indicator	Ayuso-Murillo (2017)
	Cattell's Sixteen Personality Factor (16PF - 4)	Zhang (2013)
	Cattell's Sixteen Personality Factor Form C (16PF - C)	Reeve (1980)
Self-sufficiency	16F Personality Indicator	Amenta (1984)

Self-transcendence	Temperament and Character Inventory (TCI-240)	Yildirim (2012)
	Temperament and Character Inventory (TCI-R 140)	Ball (2015)<br>Ball (2016)<br>Campbell (2013)<br>Campbell (2014)<br>Eley (2009)<br>Eley (2011a)<br>Eley (2011b)<br>Eley (2012)<br>Eley (2013)
	Temperament and Character Inventory (TCI-R 140) - mean score	Eley (2015)
		Laurence (2016)
Sense of well-being	California Psychological Inventory	Bry (1980)<br>Coombs (1993)

Sensing	Myers-Briggs Type Indicator (MBTI)	Al-Dlaigan (2017)<br>Bean (1995)<br>Chang (2019)<br>Clack (2004)<br>Cross (1984)<br>Grandy (1996)<br>Harris (1985)<br>Lewis (1994)<br>Needleman (2011)<br>Whitworth (2005)
	Personal Style Inventory (PSI)	Bean (1993)
Sensing/feeling	Myers-Briggs Type Indicator (MBTI)	Clack (2004)
Sensing/intuition	Myers-Briggs Type Indicator (MBTI)	Amenta (1984)
Sensing/judging	Kiersey Temperament Sorter	McPhail (2002)
	Kiersey-Bates Personality Inventory	Lysack (2001)
	Myers-Briggs Type Indicator (MBTI)	Grandy (1996)
Sensing/perceiving	Kiersey Temperament Sorter	McPhail (2002)
	Kiersey-Bates Personality Inventory	Lysack (2001)

	Myers-Briggs Type Indicator (MBTI)	Grandy (1996)
Sensing/thinking	Myers-Briggs Type Indicator (MBTI)	Clack (2004)
Sensitivity	16F Personality Indicator	Amenta (1984)<br>Ayuso-Murillo (2017)
	Cattell's Sixteen Personality Factor (16PF - 4)	Zhang (2013)
	Cattell's Sixteen Personality Factor Form C (16PF - C)	Reeve (1980)
Shrewdness	Cattell's Sixteen Personality Factor (16PF - 4)	Zhang (2013)
	Cattell's Sixteen Personality Factor Form C (16PF - C)	Reeve (1980)
Shyness	Freiburg Personality Inventory (FPI)	Bergmueller (2018)
Sociability	California Psychological Inventory	Bry (1980)<br>Coombs (1993)
	Freiburg Personality Inventory (FPI)	Bergmueller (2018)
	Gordon Personal Profile Index (GPPI)	Cordina (2012)
	Hogan Personality Inventory (HPI)	Kovach (2010)
Social	John Holland Personality Test	Ahmadpanah (2015)

Social boldness	Cattell's Sixteen Personality Factor (16PF - 4)	Zhang (2013)
	Cattell's Sixteen Personality Factor Form C (16PF - C)	Reeve (1980)
Social competence	Resilence Scale for Adults	Kutluturkan (2016)
Social pressure	California Psychological Inventory	Bry (1980)
		Coombs (1993)
		Dumitru (2012)
Social resources	Resilence Scale for Adults	Kutluturkan (2016)
Socialisation	California Psychological Inventory	Bry (1980)<br>Coombs (1993)
	Karolinska Scales of Personality (KSP)	Pålsson (1996)
Sociotropy	Sociotropy– autonomy Scale (SAS)	Engin (2009)<br>Kaya (2018)
Somatic anxiety	Karolinska Scales of Personality (KSP)	Pålsson (1996)
Somatic complaints	Minnesota Multiphasic Personality Inventory (MMPI)	McCranie (1989)
Spontaneous	Personality Styles and Disorder Inventory (PSDI)	Wolf (2022)
Spontaneous aggressivity	Freiburg Personality Inventory (FPI)	Bergmueller (2018)
Stability	16F Personality Indicator	Ayuso-Murillo (2017)

Straightforwardness	NEO Personality Inventory - 3 (NEO-PI-3)	Kennedy (2014)
Structural style	Resilence Scale for Adults	Kutluturkan (2016)
Succourance	Edwards Personal Preference Schedule (EPPS)	Wright (1993a)
Suspiciousness	16F Personality Indicator	Amenta (1984)
Tender-mindedness	NEO Personality Inventory - 3 (NEO-PI-3)	Kennedy (2014)
Tension	16F Personality Indicator	Amenta (1984)<br>Ayuso-Murillo (2017)
	Cattell's Sixteen Personality Factor (16PF - 4)	Zhang (2013)
	Cattell's Sixteen Personality Factor Form C (16PF - C)	Reeve (1980)
The cool type	Hamburg Personality Inventroy (HPI-K84)	Pajonk (2012)
The crisis-manger type	Hamburg Personality Inventroy (HPI-K84)	Pajonk (2012)
The unconventional type	Hamburg Personality Inventroy (HPI-K84)	Pajonk (2012)

Thinking	Myers-Briggs Type Indicator (MBTI)	Al-Dlaigan (2017)<br>Bean (1995)<br>Chang (2019)<br>Clack (2004)<br>Cross (1984)<br>Grandy (1996)<br>Harris (1985)<br>Lewis (1994)<br>Needleman (2011)<br>Whitworth (2005)
	Personal Style Inventory (PSI)	Bean (1993)
Thinking/feeling	Myers-Briggs Type Indicator (MBTI)	Amenta (1984)
Tolerance	California Psychological Inventory	Bry (1980)<br>Coombs (1993)<br>Dumitru (2012)
Tolerance of failure and viewing life as a challenge	Resiliencey Assessment Scale	Marcisz-Dyla (2022)
Tough-mindedness	16F Personality Indicator	Ayuso-Murillo (2017)
Trait Anger	Trait Anger–Anger Expression Scales	Kaya (2018)
Trust	NEO Personality Inventory - 3 (NEO-PI-3)	Kennedy (2014)

Type A behaviour - achievement striving	Revised Jenkins Activity Survey	Martinussen (2011)
Type A behaviour - impatience-irritability	Revised Jenkins Activity Survey	Martinussen (2011)
Type D	Type D Personality Scale (DS-14)	Tuman (2022)<br>Vallone (2020)
Type D - Sum	Type D Personality Scale (DS-14)	Marcisz-Dyla (2022)
Unselfish	Personality Styles and Disorder Inventory (PSDI)	Wolf (2022)
Values	NEO Personality Inventory - 3 (NEO-PI-3)	Kennedy (2014)
Venturesomeness	16F Personality Indicator	Amenta (1984)
Vigilance	16F Personality Indicator	Ayuso-Murillo (2017)
	Cattell's Sixteen Personality Factor (16PF - 4)	Zhang (2013)
	Cattell's Sixteen Personality Factor Form C (16PF - C)	Reeve (1980)
	Ten-Item Personality Inventory (TIPI)	Bogacheva (2019)
Vigour	Gordon Personal Profile Index (GPPI)	Cordina (2012)
Warmth	16F Personality Indicator	Amenta (1984)
		Ayuso-Murillo (2017)

	Cattell's Sixteen Personality Factor (16PF - 4)	Zhang (2013)
	Cattell's Sixteen Personality Factor Form C (16PF - C)	Reeve (1980)
	NEO Personality Inventory - 3 (NEO-PI-3)	Kennedy (2014)
Well-being	California Psychological Inventory	Dumitru (2012)
Wilful	Personality Styles and Disorder Inventory (PSDI)	Wolf (2022)
Work orientation	California Psychological Inventory	Dumitru (2012)

**Behaviour**		
Behaviour (total)	Rokeach Values Survey (RVS)	Bailey 1988
Conscientiousness	DiSC	Keogh (2019)<br>Ogunyemi (2011)
Dominance	DiSC	Keogh (2019)<br>Ogunyemi (2011)
Influence	DiSC	Keogh (2019)<br>Ogunyemi (2011)
Intermediate type	Framingham Type A Scale	Marcisz-Dyla (2022)
Steadiness	DiSC	Keogh (2019)<br>Ogunyemi (2011)
Type A	Bortner Measure of Type A behaivour	Baglioni (1990)<br>Vallone (2020)
	Framingham Type A Scale	Marcisz-Dyla (2022)
	Jenkins Activity Survey	Matthews (1980)
	Modified Bortner Measure of Type A behaivour	Pascual (2021)
	Type A Behaviour Scale (TABS)	Boey (1999)
	Type A/B Behavioural Pattern Scale (ABBPS)	Ghasemian (2017)
Type B	Framingham Type A Scale	Marcisz-Dyla (2022)
	Type A/B Behavioural Pattern Scale (ABBPS)	Ghasemian (2017)

**Emotional Intelligence**		
Emotional Intelligence (total)	Bar-On's Emotional Quotient Inventory (EQ-I) - 125 item	Ranjha (2010)
	Bar-On's Emotional Quotient Inventory (EQ-I) - 133 item	Bamberger (2017)<br>Crowne (2017)<br>Dugan (2014)<br>Erkutlu (2012)<br>Gertis (2004)<br>Harper (2012)<br>Looff (2019)<br>Van Dusseldorp (2011)<br>Wagner (2002)
	Bar-On's Emotional Quotient Inventory (EQ-I) - 133 item sum	Nooryan (2012)<br>Sharif (2013)
	Bar-On's Emotional Quotient Inventory (EQ-I) - 87 items	Başoğul (2016)<br>Kahraman (2016)
	Bar-On's Emotional Quotient Inventory (EQ-I) - 90 item	Foji (2020)<br>Kousha (2018)<br>Sabzevar (2016)<br>Shabany (2018)<br>Yarbeigi (2021)

	Bar-On's Emotional Quotient Inventory 2.0 (EQ-I 2.0)	Coladonato (2017)<br>MazzellaEbstein (2019)<br>Mazzella-Ebstein (2021)<br>Papanagnou (2017)<br>Ramzan Shahid (2018)<br>Tyczkowski (2015)
	Bradberry & Greaves Emotional Intelligence Questionnaire	Bikmoradi (2018)<br>Kheirkhah (2018)<br>Moradian (2022)<br>Tofighi (2015)<br>Tofighi (2022)
	Brief Emotional Intelligence Scale (BEIS-10)	Abdulah (2021)
	Cyberia Shrink 33 items questionnaire	Ezzatabadi (2012)
	Emotional Competence Inventory (ECI) 2.0	Young-Ritchie (2019)
	Emotional Intelligence Assessment Scale (EIA)	AsİKarakaŞ (2020)<br>Ordu (2022)
	Emotional Intelligence Index (EQI)	vanZyl (2017)
	Emotional Intelligence Questionnaire	Alshammari (2020)
	emotional intelligence questionnaire (EIQ)	Khatri (2012)

	Emotional Intelligence Scale (EIS)	Edbor (2016)<br>Xie (2021)
	Emotional Intelligence Scale (EQS)	Fujino (2015)<br>Hirai (2020)
	Emotional Intelligence Screening Test	Por Pan (2020)
	Genos Emotional Intelligence Inventory – Concise Questionnaire (GEII)	NizarAbdulMajeed (2019)
	GENOS Emotional Intelligence Self-Assessment (GENOS EI SA)	Al-Hamdan (2017)<br>Kozlowski (2018)
	Goleman's Emotional Intelligence Scale	Raeissi (2019)
	Goleman's Emotional Intelligence Scale – 5-point scale average	Chao (2016)
	Hay 360 Emotional Competence Inventory (Hay 360 ECI)	Gorgas (2015)
	Mayer-Salovey-Caruso Emotional Intelligence Test (MSCEIT)	Cherry (2018)<br>Codier (2013)<br>Ohlson (2015)<br>Prufeta (2017)<br>Reemts (2015)<br>Sims (2017)

	Multidimensional Measure of Emotional Intelligence (MMEI)	Bidlan (2014b)
	Nursing Emotional Intelligence Scale (NEIS)	Lalonde (2017)
	Occupational Stress Index	Bidlan (2014b)
	Revised Schutte Emotional Intelligence Scale (RSEIS)	Kılıç (2022)
	Scale of Emotional Functioning: Health Service Provider (SEF:HSP)	Beierle (2019)
	Schutte Self-Report Emotional Intelligence Test (SSEIT)	Ali (2017)<br>Kaur (2013)<br>Popa-Velea (2019)<br>Taylan (2021)<br>Zeidner (2013)
	Schutte Self-Report Emotional Intelligence Test (SSEIT) - sum score	Bittinger (2020)<br>Głębocka (2019)<br>Lartey (2021)<br>Maillet (2021)<br>Pau (2015)<br>Tagoe (2017)
	Schutte Self-Report Emotional Intelligence Test (SSEIT-33)	Heydari (2016)
	Schutte's Emotional Intelligence Test (SEIT)	Sabanciogullari (2020)

	Self-Emotional Intelligence Scale	Sharmila (2014)
	Self-Rated Emotional Intelligence Scale (SREIS)	Al-Hamdan (2021)<br>Sims (2017)
	Self-Report Emotional Intelligence Test (SREIT)	Karimi (2014)
	Shrink's Emotional Intelligence Questionnaire	Tajigharajeh (2021)
	Siberia Schering's Emotional Intelligence Standard Questionnaire	Rakhshani (2018)
	Swinburne University Emotional Intelligence Test (SUEIT)	Chaffey (2012)<br>Görgens-Ekermans (2012)
	Trait Emotional Intelligence Questionnaire (TEIQue)	Coskun (2018)<br>Hollis (2017)<br>McKinley (2015)<br>Placek (2019)

	Trait Emotional Intelligence Questionnaire-Short Form (TEIQue-SF)	Abu Awwad (2020)<br>Al Hosani (2020)<br>Al Huseini (2019)<br>Frias (2021)<br>Gelkop (2022)<br>Gleason (2020)<br>Holliday (2017)<br>Jacobs (2016)<br>Khrais (2021)<br>Lin (2016)<br>Mackay (2012)<br>McKenna (2020)<br>Ramsey-Haynes (2021)
	Trait Emotional Intelligence Questionnaire-Short Form (TEIQue-SF) - sum score	Nagel (2016)<br>Spano-Szekely (2016)<br>Swami (2013)
	Wong and Law Emotional Intelligence Scale (WLEIS) - 5-point scale	Cheng (2012)<br>Hong (2016)<br>Jummi (2019)<br>Mao (2021)<br>Srivastava (2021)<br>Zhu (2015)

	Wong and Law Emotional Intelligence Scale (WLEIS) - 5-point scale (sum)	Uzonwanne (2016)
	Wong and Law Emotional Intelligence Scale (WLEIS) - 6-point scale	Al-Ruzzieh (2021)<br>Habib (2012)<br>Lawal (2017)
	Wong and Law Emotional Intelligence Scale (WLEIS) - 7-point scale	Gou (2021)<br>Hwang (2022)<br>Jones (2010)<br>Ju-Young (2019)<br>Shah (2022)<br>Weng (2008)<br>Weng (2011a)<br>Zeb (2021)
	Wong and Law Emotional Intelligence Scale (WLEIS) - 7-point scale sum	Kim (2016)<br>Liu (2018)
	Wong and Law Emotional Intelligence Scale (WLEIS-C) - chinese version	Sun (2021)
Achievement orientation	Emotional and Social Competency Inventory (ESCI)	Jacoby (2022)

Adaptability	Bar-On's Emotional Quotient Inventory (EQ-I) - 133 item	Bamberger (2017)<br>Gertis (2004)<br>Harper (2012)<br>Nooryan (2012)<br>Van Dusseldorp (2011)<br>Wagner (2002)
	Bar-On's Emotional Quotient Inventory (EQ-I) - 87 items	Başoğul (2016)<br>Kahraman (2016)
	Brief Emotional Intelligence Inventory for Senior Citizens (EQ-i-20M)	Pérez-Fuentes (2019)
	Emotional and Social Competency Inventory (ESCI)	Jacoby (2022)
	Reduced Emotional Intelligence Inventory for Adults (EQ-i-20M)	Pérez-Fuentes (2018)
Altruistic behaviour	Emotional Intelligence Scale (EIS)	Edbor (2016)
Appraisal of Emotions	Revised Schutte Emotional Intelligence Scale (RSEIS)	Kılıç (2022)
	Schutte Self-Report Emotional Intelligence Test (SSEIT)	Taylan (2021)
Appraisal of other's emotions	Brief Emotional Intelligence Scale (BEIS-10)	Abdulah (2021)<br>YousifAli (2020)
Appraisal of own emotions	Brief Emotional Intelligence Scale (BEIS-10)	Abdulah (2021)<br>YousifAli (2020)

Assertiveness	Bar-On's Emotional Quotient Inventory (EQ-I) - 133 item	Gertis (2004)<br>Harper (2012)<br>Nooryan (2012)<br>Van Dusseldorp (2011)
	Bar-On's Emotional Quotient Inventory (EQ-I) - 87 items	Başoğul (2016)
	Bar-On's Emotional Quotient Inventory 2.0 (EQ-I 2.0)	Coladonato (2017)<br>Ramzan Shahid (2018)
Benefiting from emotions	Schuttes Emotional Intelligence Test (SEIT)	Sabanciogullari (2020)
Care for patients	Nursing Manager's Leadership Behavior Scale	Furukawa (2021)
Coach and mentor	Emotional and Social Competency Inventory (ESCI)	Jacoby (2022)
Cognitive thought	Emotional Competence Inventory (ECI)	Efkarpidis (2012)
Commitment	Emotional Intelligence Scale (EIS)	Edbor (2016)
Conflict management	Emotional and Social Competency Inventory (ESCI)	Jacoby (2022)
Controlling emotions	GENOS Emotional Intelligence Self-Assessment (GENOS EI SA)	Al-Hamdan (2017)
Decision making	Bar-On's Emotional Quotient Inventory 2.0 (EQ-I 2.0)	Coladonato (2017)<br>Ramzan Shahid (2018)<br>Tyczkowski (2015)

	GENOS Emotional Intelligence Self-Assessment (GENOS EI SA)	Al-Hamdan (2017)
Deep acting	Emotional Labour	Liu (2018)
Emotional attention	Trait Meta Mood Scale (TMMS-24)	Aradilla-Herrero (2014)<br>AugustoLanda (2008)<br>Giménez-Espert (2017)<br>Giménez-Espert (2018)<br>Giménez-Espert (2019)<br>Giménez-Espert (2020)<br>Prado-Gascó (2019)
Emotional clarity	Trait Meta Mood Scale (TMMS-24)	Aradilla-Herrero (2014)<br>AugustoLanda (2008)<br>Giménez-Espert (2017)<br>Giménez-Espert (2018)<br>Giménez-Espert (2019)<br>Giménez-Espert (2020)<br>Prado-Gascó (2019)
Emotional control	Nursing Manager's Leadership Behavior Scale	Furukawa (2021)
	Swinburne University Emotional Intelligence Test (SUEIT)	Chaffey (2012)<br>Görgens-Ekermans (2012)
Emotional expression	Bar-On's Emotional Quotient Inventory 2.0 (EQ-I 2.0)	Coladonato (2017)<br>Ramzan Shahid (2018)

	Schuttes Emotional Intelligence Test (SEIT)	Sabanciogullari (2020)
Emotional management	Swinburne University Emotional Intelligence Test (SUEIT)	Chaffey (2012)<br>Görgens-Ekermans (2012)
Emotional perception	Emotional Intelligence Scale (EIS)	Xie (2021)
Emotional recognition & expression	Swinburne University Emotional Intelligence Test (SUEIT)	Chaffey (2012)<br>Görgens-Ekermans (2012)
Emotional repair	Trait Meta Mood Scale (TMMS-24)	Aradilla-Herrero (2014)<br>AugustoLanda (2008)<br>Giménez-Espert (2017)<br>Giménez-Espert (2018)<br>Giménez-Espert (2019)<br>Giménez-Espert (2020)<br>Prado-Gascó (2019)
Emotional self-awareness	Bar-On's Emotional Quotient Inventory (EQ-I) - 133 item	Gertis (2004)<br>Harper (2012)
	Bar-On's Emotional Quotient Inventory (EQ-I) - 87 items	Başoğul (2016)
	Bar-On's Emotional Quotient Inventory 2.0 (EQ-I 2.0)	Coladonato (2017)<br>Ramzan Shahid (2018)
	Emotional and Social Competency Inventory (ESCI)	Jacoby (2022)
Emotional self-control	Emotional and Social Competency Inventory (ESCI)	Jacoby (2022)

Emotional self-management	Emotional Intelligence Scale (EIS)	Xie (2021)
Emotional stability	Emotional Intelligence Scale (EIS)	Edbor (2016)
Emotionality	Trait Emotional Intelligence Questionnaire (TEIQue)	Coskun (2018)<br>Placek (2019)
	Trait Emotional Intelligence Questionnaire-Short Form (TEIQue-SF)	Abu Awwad (2020)<br>Al Hosani (2020)<br>Al Huseini (2019)<br>Frias (2021)<br>Gleason (2020)<br>Holliday (2017)<br>Mackay (2012)<br>McKenna (2020)<br>Nagel (2016)<br>Ramsey-Haynes (2021)
Emotions direct cognition	Swinburne University Emotional Intelligence Test (SUEIT)	Chaffey (2012)<br>Görgens-Ekermans (2012)
Empathy	Bar-On's Emotional Quotient Inventory (EQ-I) - 133 item	Gertis (2004)<br>Harper (2012)<br>Nooryan (2012)<br>Van Dusseldorp (2011)
	Bar-On's Emotional Quotient Inventory (EQ-I) - 87 items	Başoğul (2016)

	Bar-On's Emotional Quotient Inventory 2.0 (EQ-I 2.0)	Coladonato (2017)<br>Ramzan Shahid (2018)
	Emotional and Social Competency Inventory (ESCI)	Jacoby (2022)
	Emotional Intelligence Assessment Scale (EIA)	AsİKarakaŞ (2020)<br>Ordu (2022)
	Emotional Intelligence Scale (EIS)	Edbor (2016)
	Multidimensional Measure of Emotional Intelligence (MMEI)	Bidlan (2014a)
	Shrink's Emotional Intelligence Questionnaire	Tajigharajeh (2021)
Experiential EI	Mayer-Salovey-Caruso Emotional Intelligence Test (MSCEIT)	Codier (2013)
Flexibility	Bar-On's Emotional Quotient Inventory (EQ-I) - 133 item	Gertis (2004)<br>Harper (2012)<br>Nooryan (2012)<br>Van Dusseldorp (2011)
	Bar-On's Emotional Quotient Inventory (EQ-I) - 87 items	Başoğul (2016)
	Bar-On's Emotional Quotient Inventory 2.0 (EQ-I 2.0)	Coladonato (2017)<br>Ramzan Shahid (2018)

General Mood	Bar-On's Emotional Quotient Inventory (EQ-I) - 133 item	Bamberger (2017)<br>Gertis (2004)<br>Harper (2012)<br>Nooryan (2012)<br>Van Dusseldorp (2011)<br>Wagner (2002)
	Bar-On's Emotional Quotient Inventory (EQ-I) - 87 items	Başoğul (2016)<br>Kahraman (2016)
	Brief Emotional Intelligence Inventory for Senior Citizens (EQ-i-20M)	Pérez-Fuentes (2019)
	Reduced Emotional Intelligence Inventory for Adults (EQ-i-20M)	Pérez-Fuentes (2018)
Handling relationships	Multidimensional Measure of Emotional Intelligence (MMEI)	Bidlan (2014a)
Happiness	Bar-On's Emotional Quotient Inventory (EQ-I) - 133 item	Gertis (2004)<br>Harper (2012)<br>Nooryan (2012)<br>Van Dusseldorp (2011)
	Bar-On's Emotional Quotient Inventory (EQ-I) - 87 items	Başoğul (2016)
	Bar-On's Emotional Quotient Inventory 2.0 (EQ-I 2.0)	Tyczkowski (2015)

Identifying emotions	Mayer-Salovey-Caruso Emotional Intelligence Test (MSCEIT)	Codier (2013)<br>Reemts (2015)<br>Sims (2017)
Impulse control	Bar-On's Emotional Quotient Inventory (EQ-I) - 133 item	Gertis (2004)<br>Harper (2012)<br>Nooryan (2012)<br>Van Dusseldorp (2011)
	Bar-On's Emotional Quotient Inventory (EQ-I) - 87 items	Başoğul (2016)
	Bar-On's Emotional Quotient Inventory 2.0 (EQ-I 2.0)	Coladonato (2017)<br>Ramzan Shahid (2018)
Independence	Bar-On's Emotional Quotient Inventory (EQ-I) - 133 item	Gertis (2004)<br>Harper (2012)<br>Nooryan (2012)<br>Van Dusseldorp (2011)
	Bar-On's Emotional Quotient Inventory (EQ-I) - 87 items	Başoğul (2016)
	Bar-On's Emotional Quotient Inventory 2.0 (EQ-I 2.0)	Coladonato (2017)<br>Ramzan Shahid (2018)
Influence	Emotional and Social Competency Inventory (ESCI)	Jacoby (2022)
Inspirational leadership	Emotional and Social Competency Inventory (ESCI)	Jacoby (2022)
Integrity	Emotional Intelligence Scale (EIS)	Edbor (2016)

Interpersonal	Bar-On's Emotional Quotient Inventory (EQ-I) - 133 item	Bamberger (2017)<br>Gertis (2004)<br>Harper (2012)<br>Nooryan (2012)<br>Van Dusseldorp (2011)<br>Wagner (2002)
	Bar-On's Emotional Quotient Inventory (EQ-I) - 87 items	Başoğul (2016)<br>Kahraman (2016)
	Bar-On's Emotional Quotient Inventory 2.0 (EQ-I 2.0)	Coladonato (2017)<br>Ramzan Shahid (2018)<br>Tyczkowski (2015)
	Brief Emotional Intelligence Inventory for Senior Citizens (EQ-i-20M)	Pérez-Fuentes (2019)
	Emotional Intelligence Scale (EQS)	Fujino (2015)<br>Hirai (2020)
	Reduced Emotional Intelligence Inventory for Adults (EQ-i-20M)	Pérez-Fuentes (2018)
Interpersonal relationships	Bar-On's Emotional Quotient Inventory (EQ-I) - 133 item	Gertis (2004)<br>Harper (2012)<br>Nooryan (2012)<br>Van Dusseldorp (2011)
	Bar-On's Emotional Quotient Inventory (EQ-I) - 87 items	Başoğul (2016)

	Bar-On's Emotional Quotient Inventory 2.0 (EQ-I 2.0)	Coladonato (2017)<br>Ramzan Shahid (2018)
Intrapersonal	Bar-On's Emotional Quotient Inventory (EQ-I) - 133 item	Bamberger (2017)<br>Gertis (2004)<br>Harper (2012)<br>Nooryan (2012)<br>Van Dusseldorp (2011)<br>Wagner (2002)
	Bar-On's Emotional Quotient Inventory (EQ-I) - 87 items	Başoğul (2016)<br>Kahraman (2016)
	Brief Emotional Intelligence Inventory for Senior Citizens (EQ-i-20M)	Pérez-Fuentes (2019)
	Emotional Intelligence Scale (EQS)	Fujino (2015)<br>Hirai (2020)
	Reduced Emotional Intelligence Inventory for Adults (EQ-i-20M)	Pérez-Fuentes (2018)
Management of others' emotions	Emotional Intelligence Scale (EIS)	Xie (2021)
Managing branch	Self-Rated Emotional Intelligence Scale (SREIS)	Sims (2017)
Managing emotions	GENOS Emotional Intelligence Self-Assessment (GENOS EI SA)	Al-Hamdan (2017)

	Mayer-Salovey-Caruso Emotional Intelligence Test (MSCEIT)	Codier (2008)<br>Codier (2013)<br>Reemts (2015)<br>Sims (2017)
	Multidimensional Measure of Emotional Intelligence (MMEI)	Bidlan (2014a)
Managing others’ emotions	Schutte Self-Report Emotional Intelligence Test (SSEIT)	Bittinger (2020)<br>Kaur (2015)<br>Maillet (2021)
Managing own emotions	Schutte Self-Report Emotional Intelligence Test (SSEIT)	Bittinger (2020)<br>Kaur (2015)<br>Maillet (2021)
Managing relations	Emotional Competence Inventory (ECI)<br>Emotional Intelligence Scale (EIS)	Efkarpidis (2012)<br>Edbor (2016)
Motivating oneself	Multidimensional Measure of Emotional Intelligence (MMEI)	Bidlan (2014a)
Motivation	Emotional Intelligence Scale (EIS)	Edbor (2016)
Natural acting	Emotional Labour	Liu (2018)
Optimism	Bar-On's Emotional Quotient Inventory (EQ-I) - 133 item	Gertis (2004)<br>Harper (2012)<br>Nooryan (2012)<br>Van Dusseldorp (2011)

	Bar-On's Emotional Quotient Inventory (EQ-I) - 87 items	Başoğul (2016)
	Bar-On's Emotional Quotient Inventory 2.0 (EQ-I 2.0)	Coladonato (2017)<br>Ramzan Shahid (2018)
Optimism/emotion regulation	Revised Schutte Emotional Intelligence Scale (RSEIS)	Kılıç (2022)
	Schutte Self-Report Emotional Intelligence Test (SSEIT)	Taylan (2021)
	Schuttes Emotional Intelligence Test (SEIT)	Sabanciogullari (2020)
Organisational awareness	Emotional and Social Competency Inventory (ESCI)	Jacoby (2022)
Others emotional appraisal	Korean Emotional Intelligence Scale	Lee (2021)
	Self-Emotional Intelligence Scale	Choudary (2011)<br>Sharmila (2014)
	Wong and Law Emotional Intelligence Scale (WLEIS) - 4-point scale	Al-Hamdan (2020)

	Wong and Law Emotional Intelligence Scale (WLEIS) - 5-point scale	Apore (2019)<br>Hong (2016)<br>Issa (2022)<br>Jummi (2019)<br>Mao (2021)<br>Srivastava (2021)<br>Zhu (2015)
	Wong and Law Emotional Intelligence Scale (WLEIS) - 5-point scale (sum)	Uzonwanne (2016)
	Wong and Law Emotional Intelligence Scale (WLEIS) - 6-point scale	Al-Ruzzieh (2021)<br>Lawal (2017)
	Wong and Law Emotional Intelligence Scale (WLEIS) - 7-point scale	Jones (2010)<br>Ju-Young (2019)<br>Shah (2022)<br>Weng (2011b)<br>Weng (2011c)<br>Zeb (2021)
	Wong and Law Emotional Intelligence Scale (WLEIS-C) - Chinese version	Sun (2021)
Perceiving branch	Self-Rated Emotional Intelligence Scale (SREIS)	Sims (2017)

Perceiving emotions	Mayer-Salovey-Caruso Emotional Intelligence Test (MSCEIT)	Codier (2008)
Perception of emotion	Schutte Self-Report Emotional Intelligence Test (SSEIT)	Bittinger (2020)<br>Kaur (2015)<br>Maillet (2021)
Positive outlook	Emotional and Social Competency Inventory (ESCI)	Jacoby (2022)
Problem solving	Bar-On's Emotional Quotient Inventory (EQ-I) - 133 item	Gertis (2004)<br>Harper (2012)<br>Nooryan (2012)<br>Van Dusseldorp (2011)
	Bar-On's Emotional Quotient Inventory (EQ-I) - 87 items	Başoğul (2016)
	Bar-On's Emotional Quotient Inventory 2.0 (EQ-I 2.0)	Coladonato (2017)<br>Ramzan Shahid (2018)
Reality testing	Bar-On's Emotional Quotient Inventory (EQ-I) - 133 item	Gertis (2004)<br>Harper (2012)<br>Nooryan (2012)<br>Van Dusseldorp (2011)
	Bar-On's Emotional Quotient Inventory (EQ-I) - 87 items	Başoğul (2016)
	Bar-On's Emotional Quotient Inventory 2.0 (EQ-I 2.0)	Coladonato (2017)<br>Ramzan Shahid (2018)

Recognising and expressing emotions	GENOS Emotional Intelligence Self-Assessment (GENOS EI SA)	Al-Hamdan (2017)
Regulation of emotion	Korean Emotional Intelligence Scale	Lee (2021)
	Self-Emotional Intelligence Scale	Choudary (2011)<br>Sharmila (2014)
	Wong and Law Emotional Intelligence Scale (WLEIS) - 4-point scale	Al-Hamdan (2020)
	Wong and Law Emotional Intelligence Scale (WLEIS) - 5-point scale	Apore (2019)<br>Hong (2016)<br>Issa (2022)<br>Jummi (2019)<br>Mao (2021)<br>Srivastava (2021)<br>Zhu (2015)
	Wong and Law Emotional Intelligence Scale (WLEIS) - 5-point scale (sum)	Uzonwanne (2016)
	Wong and Law Emotional Intelligence Scale (WLEIS) - 6-point scale	Al-Ruzzieh (2021)<br>Lawal (2017)

	Wong and Law Emotional Intelligence Scale (WLEIS) - 7-point scale	Jones (2010)<br>Ju-Young (2019)<br>Shah (2022)<br>Weng (2011b)<br>Weng (2011c)<br>Zeb (2021)
	Wong and Law Emotional Intelligence Scale (WLEIS-C) - Chinese version	Sun (2021)
Regulation of others' emotions	Brief Emotional Intelligence Scale (BEIS-10)	Abdulah (2021)<br>YousifAli (2020)
Regulation of own emotions	Brief Emotional Intelligence Scale (BEIS-10)	Abdulah (2021)<br>YousifAli (2020)
Relationship management	Bradberry & Greaves Emotional Intelligence Questionnaire	Bikmoradi (2018)<br>Kheirkhah (2018)<br>Moradian (2022)<br>Tofighi (2015)<br>Tofighi (2022)
Self-emotional appraisal	Korean Emotional Intelligence Scale	Lee (2021)
	Self-Emotional Intelligence Scale	Choudary (2011)<br>Sharmila (2014)

	Wong and Law Emotional Intelligence Scale (WLEIS) - 4-point scale	Al-Hamdan (2020)
	Wong and Law Emotional Intelligence Scale (WLEIS) - 5-point scale	Apore (2019)<br>Hong (2016)<br>Issa (2022)<br>Jummi (2019)<br>Mao (2021)<br>Srivastava (2021)<br>Zhu (2015)
	Wong and Law Emotional Intelligence Scale (WLEIS) - 5-point scale (sum)	Uzonwanne (2016)
	Wong and Law Emotional Intelligence Scale (WLEIS) - 6-point scale	Al-Ruzzieh (2021)<br>Lawal (2017)
	Wong and Law Emotional Intelligence Scale (WLEIS) - 7-point scale	Jones (2010)<br>Ju-Young (2019)<br>Shah (2022)<br>Weng (2011b)<br>Weng (2011c)<br>Zeb (2021)

	Wong and Law Emotional Intelligence Scale (WLEIS-C) - chinese version	Sun (2021)
Self-actualisation	Bar-On's Emotional Quotient Inventory (EQ-I) - 133 item	Gertis (2004)<br>Harper (2012)<br>Nooryan (2012)<br>Van Dusseldorp (2011)
	Bar-On's Emotional Quotient Inventory (EQ-I) - 87 items	Başoğul (2016)
	Bar-On's Emotional Quotient Inventory 2.0 (EQ-I 2.0)	Coladonato (2017)
		Ramzan Shahid (2018)
Self-awareness	Bar-On's Emotional Quotient Inventory (EQ-I) - 133 item	Nooryan (2012)<br>Van Dusseldorp (2011)
	Bradberry & Greaves Emotional Intelligence Questionnaire	Bikmoradi (2018)<br>Kheirkhah (2018)<br>Moradian (2022)<br>Tofighi (2015)<br>Tofighi (2022)
	Emotional Competence Inventory (ECI)	Efkarpidis (2012)
	Emotional Intelligence Assessment Scale (EIA)	AsİKarakaŞ (2020)<br>Ordu (2022)
	Emotional Intelligence Scale (EIS)	Edbor (2016)

	Goleman's Emotional Intelligence Scale	Raeissi (2019)
	Multidimensional Measure of Emotional Intelligence (MMEI)	Bidlan (2014a)
	Shrink's Emotional Intelligence Questionnaire	Tajigharajeh (2021)
	Siberia Schering's Emotional Intelligence Standard Questionnaire	Rakhshani (2018)
Self-control	Shrink's Emotional Intelligence Questionnaire	Tajigharajeh (2021)
	Siberia Schering's Emotional Intelligence Standard Questionnaire	Rakhshani (2018)
	Trait Emotional Intelligence Questionnaire (TEIQue)	Coskun (2018)<br>Placek (2019)

	Trait Emotional Intelligence Questionnaire-Short Form (TEIQue-SF)	Abu Awwad (2020)<br>Al Hosani (2020)<br>Al Huseini (2019)<br>Frias (2021)<br>Gleason (2020)<br>Holliday (2017)<br>Mackay (2012)<br>McKenna (2020)<br>Nagel (2016)<br>Ramsey-Haynes (2021)
Self-development	Emotional Intelligence Scale (EIS)	Edbor (2016)
Self-expression	Bar-On's Emotional Quotient Inventory 2.0 (EQ-I 2.0)	Coladonato (2017)<br>Ramzan Shahid (2018)<br>Tyczkowski (2015)
Self-management	Bradberry & Greaves Emotional Intelligence Questionnaire	Bikmoradi (2018)<br>Kheirkhah (2018)<br>Moradian (2022)<br>Tofighi (2015)<br>Tofighi (2022)
	Emotional Competence Inventory (ECI)	Efkarpidis (2012)
	Goleman's Emotional Intelligence Scale	Raeissi (2019)

Self-motivation	Emotional Intelligence Assessment Scale (EIA)	AsİKarakaŞ (2020)<br>Ordu (2022)
	Shrink's Emotional Intelligence Questionnaire	Tajigharajeh (2021)
	Siberia Schering's Emotional Intelligence Standard Questionnaire	Rakhshani (2018)
Self-perception	Bar-On's Emotional Quotient Inventory 2.0 (EQ-I 2.0)	Coladonato (2017)<br>Ramzan Shahid (2018)<br>Tyczkowski (2015)
Self-regard	Bar-On's Emotional Quotient Inventory (EQ-I) - 133 item	Gertis (2004)<br>Harper (2012)<br>Nooryan (2012)<br>Van Dusseldorp (2011)
	Bar-On's Emotional Quotient Inventory (EQ-I) - 87 items	Başoğul (2016)
	Bar-On's Emotional Quotient Inventory 2.0 (EQ-I 2.0)	Coladonato (2017)<br>Ramzan Shahid (2018)
Self-regulation	Emotional Intelligence Assessment Scale (EIA)	AsİKarakaŞ (2020)<br>Ordu (2022)
Situational	Emotional Intelligence Scale (EQS)	Fujino (2015)<br>Hirai (2020)
Sociability	Trait Emotional Intelligence Questionnaire (TEIQue)	Coskun (2018)<br>Placek (2019)

	Trait Emotional Intelligence Questionnaire-Short Form (TEIQue-SF)	Abu Awwad (2020)<br>Al Hosani (2020)<br>Al Huseini (2019)<br>Frias (2021)<br>Gleason (2020)<br>Holliday (2017)<br>Mackay (2012)<br>McKenna (2020)<br>Nagel (2016)<br>Ramsey-Haynes (2021)
Social awareness	Bradberry & Greaves Emotional Intelligence Questionnaire	Bikmoradi (2018)<br>Kheirkhah (2018)<br>Moradian (2022)<br>Tofighi (2015)<br>Tofighi (2022)
	Emotional Competence Inventory (ECI)	Efkarpidis (2012)
	Goleman's Emotional Intelligence Scale	Raeissi (2019)
Social consciousness	Siberia Schering's Emotional Intelligence Standard Questionnaire	Rakhshani (2018)

Social responsibility	Bar-On's Emotional Quotient Inventory (EQ-I) - 133 item	Gertis (2004)<br>Harper (2012)<br>Nooryan (2012)<br>Van Dusseldorp (2011)
	Bar-On's Emotional Quotient Inventory (EQ-I) - 87 items	Başoğul (2016)
	Bar-On's Emotional Quotient Inventory 2.0 (EQ-I 2.0)	Coladonato (2017)<br>Ramzan Shahid (2018)
Social skills	Emotional Intelligence Assessment Scale (EIA)	AsİKarakaŞ (2020)<br>Ordu (2022)
	Goleman's Emotional Intelligence Scale	Raeissi (2019)
	Shrink's Emotional Intelligence Questionnaire	Tajigharajeh (2021)
	Siberia Schering's Emotional Intelligence Standard Questionnaire	Rakhshani (2018)
Staff nurturing and support	Nursing Manager's Leadership Behavior Scale	Furukawa (2021)
Strategic EI	Mayer-Salovey-Caruso Emotional Intelligence Test (MSCEIT)	Codier (2013)

Stress Management	Bar-On's Emotional Quotient Inventory (EQ-I) - 133 item	Bamberger (2017)<br>Gertis (2004)<br>Harper (2012)<br>Nooryan (2012)<br>Van Dusseldorp (2011)<br>Wagner (2002)
	Bar-On's Emotional Quotient Inventory (EQ-I) - 87 items	Başoğul (2016)<br>Kahraman (2016)
	Bar-On's Emotional Quotient Inventory 2.0 (EQ-I 2.0)	Coladonato (2017)<br>Ramzan Shahid (2018)<br>Tyczkowski (2015)
	Brief Emotional Intelligence Inventory for Senior Citizens (EQ-i-20M)	Pérez-Fuentes (2019)
	Reduced Emotional Intelligence Inventory for Adults (EQ-i-20M)	Pérez-Fuentes (2018)
Stress tolerance	Bar-On's Emotional Quotient Inventory (EQ-I) - 133 item	Gertis (2004)<br>Harper (2012)<br>Nooryan (2012)<br>Van Dusseldorp (2011)
	Bar-On's Emotional Quotient Inventory (EQ-I) - 87 items	Başoğul (2016)
	Bar-On's Emotional Quotient Inventory 2.0 (EQ-I 2.0)	Coladonato (2017)<br>Ramzan Shahid (2018)

Surface acting	Emotional Labour	Liu (2018)
Teamwork	Emotional and Social Competency Inventory (ESCI)	Jacoby (2022)
Understanding branch	Self-Rated Emotional Intelligence Scale (SREIS)	Sims (2017)
Understanding emotions	Mayer-Salovey-Caruso Emotional Intelligence Test (MSCEIT)	Codier (2008)<br>Codier (2013)<br>Reemts (2015)<br>Sims (2017)
	Swinburne University Emotional Intelligence Test (SUEIT)	Chaffey (2012)
Understanding others' emotions	GENOS Emotional Intelligence Self-Assessment (GENOS EI SA)	Al-Hamdan (2017)
	Swinburne University Emotional Intelligence Test (SUEIT)	Görgens-Ekermans (2012)
Use of emotion	Emotional Intelligence Scale (EIS)	Xie (2021)
	Korean Emotional Intelligence Scale	Lee (2021)
	Self-Emotional Intelligence Scale	Choudary (2011)<br>Sharmila (2014)
	Wong and Law Emotional Intelligence Scale (WLEIS) - 4-point scale	Al-Hamdan (2020)

	Wong and Law Emotional Intelligence Scale (WLEIS) - 5-point scale	Apore (2019)<br>Hong (2016)<br>Issa (2022)<br>Jummi (2019)<br>Mao (2021)<br>Srivastava (2021)<br>Zhu (2015)
	Wong and Law Emotional Intelligence Scale (WLEIS) - 5-point scale (sum)	Uzonwanne (2016)
	Wong and Law Emotional Intelligence Scale (WLEIS) - 6-point scale	Al-Ruzzieh (2021)<br>Lawal (2017)
	Wong and Law Emotional Intelligence Scale (WLEIS) - 7-point scale	Jones (2010)<br>Ju-Young (2019)<br>Shah (2022)<br>Weng (2011b)<br>Weng (2011c)<br>Zeb (2021)
	Wong and Law Emotional Intelligence Scale (WLEIS-C) - Chinese version	Sun (2021)
Using branch	Self-Rated Emotional Intelligence Scale (SREIS)	Sims (2017)

Using emotions	Mayer-Salovey-Caruso Emotional Intelligence Test (MSCEIT)	Codier (2008)<br>Codier (2013)<br>Reemts (2015)<br>Sims (2017)
Utilising emotions	Revised Schutte Emotional Intelligence Scale (RSEIS)	Kılıç (2022)
	Schutte Self-Report Emotional Intelligence Test (SSEIT)	Bittinger (2020)<br>Kaur (2015)<br>Maillet (2021)<br>Taylan (2021)
	Brief Emotional Intelligence Scale (BEIS-10)	Abdulah (2021)<br>YousifAli (2020)
Value orientation	Emotional Intelligence Scale (EIS)	Edbor (2016)
Well-being	Trait Emotional Intelligence Questionnaire (TEIQue)	Coskun (2018)<br>Placek (2019)

	Trait Emotional Intelligence Questionnaire-Short Form (TEIQue-SF)	Abu Awwad (2020)<br>Al Hosani (2020)<br>Al Huseini (2019)<br>Frias (2021)<br>Gleason (2020)<br>Holliday (2017)<br>Mackay (2012)<br>McKenna (2020)<br>Nagel (2016)<br>Ramsey-Haynes (2021)
Wellness	Bar-On's Emotional Quotient Inventory 2.0 (EQ-I 2.0)	Ramzan Shahid (2018)
