# Supplementary material for: Profiling health professionals’ personality traits, behaviour styles and emotional intelligence: a systematic review
Source: BMC Med Educ. 2023 Feb 18;23:120. doi: 10.1186/s12909-023-04003-y (PMC9938999; doi:10.1186/s12909-023-04003-y)
Supplement: Supplementary file 4 — Additional file 4. Data Extraction Table. [file 12909_2023_4003_MOESM4_ESM.pdf]

Additional file 4. Data Extraction Table

Author (Year) Title	Participants	Measured Factors	Tool	Outcomes and Context	Main Findings	Critical Appraisal %
**Abdulah (2021) Emotional intelligence and its impacts on the clinical performance of nurses in general public hospitals**	Nurses (n=194)<br>Males (n=110)<br>Females (n=84)	Emotional Intelligence	Brief Emotional Intelligence Scale (BEIS-10)	Country:<br>Iraqi<br>Method of Collection:<br>Nil specifics provided.<br>Analysis:<br>mean and standard deviations	Nursing EI mean (SD) total of 2.56 (0.39) and score by subscale:<br>- appraisal of own emotions 1.95 (0.71)<br>- Appraisal of other's emotions 3.20 (0.74)<br>- Regulation of own emotions 2.33 (0.75)<br>- Regulation of others 'emotions 2.59 (0.82)<br>- Utilization of emotions 2.72 (0.94)	100.00%
**Abelsen (2015) Young doctors' preferences for payment systems: the influence of gender and personality traits.**	Final year medical students and interns (n=829)<br>Males (n=338)<br>Females (n=490)	Personality	Jackson Personality Inventory-revised	Country:<br>Norway<br>Method of Collection:<br>Online survey distributed through internship organisers, with two reminder emails for completion.<br>Analysis:<br>six risk attitude questions within tool were averaged to create a risk-prone index.	Risk-prone index mean 2.9 (1-5.3), with higher scores indicating that the doctors are more risk prone.	100.00%

**AbuAwwad (2020) Examining the Relationship between Emotional Intelligence, Leadership Attributes and Workplace Experience of Australian Chief Radiographers**	Chief Radiographers (n= 22)	Emotional Intelligence	TEIQUe-SF	Country:<br>Australia<br>Method of Collection:<br>Survey was emailed to convenient sample of chief radiographers on database.<br>Analysis:<br>Descriptive statistics, Kruskal-Wallis, Mann-Whitney U-test, Spearman's correlations.	Chief Radiologists < 10 years as chief EI mean 5.59 and by subscale:<br>- Well-being 6.25<br>- Self-control 5.48<br>- Emotionality 5.36<br>- Sociability 5.15<br><br>Chief Radiologists 10-20 years as chief EI mean 5.76 and by subscale:<br>- Well-being 5.95<br>- Self-control 5.52<br>- Emotionality 5.61<br>- Sociability 5.64<br><br>Chief Radiologists > 20 years as chief EI mean 5.46 and by subscale:<br>- Well-being 5.91<br>- Self-control 5.19<br>- Emotionality 5.36<br>- Sociability 5.29<br><br>No significant difference found across global and subscale scores and years of experience as chief radiographer.	85.71%

**Ahmadpanah (2015) Association of occupational burnout and type of personality in Iranian general practitioners.**	General practitioners working in emergency wards (n=100)<br>Males (n=71)<br>Females (n=29)	Personality	John Holland Personality Test	<u>Country:</u><br>Iran<br><u>Method of Collection:</u><br>Two tools were collected via face-to-face interviews, over 12 month period across 8 hospitals.<br><u>Analysis:</u><br>Individual Analysis was completed with respect to each tool and relationship between them.	Percentage of general practitioner population personality type:<br>- Realistic 2%<br>- Social 41%<br>- Investigative 35%<br>- Enterprising 6%<br>- Artistic 13%<br>- Conventional 3%<br><br>General practitioners exhibit largely social and investigative personality traits.	71.43%
**Al Hosani (2020) Reliability and Validity Testing of the Trait Emotional Intelligence Questionnaire-Short Form to Predict Nurses' Job Satisfaction**	Nurses (n=552)<br>Males (n=98)<br>Females (n=454)	Emotional Intelligence	Trait Emotional Intelligence Questionnaire-Short Form (TEIQue-SF)	<u>Country:</u><br>Dubai<br><u>Method of Collection:</u><br>Nursing staff in Abu Dhabi governmental hospitals, survey collected online between May - June 2018 via distribution through HR.<br><u>Analysis:</u><br>Descriptive statistics, Kolmogorov-Smirnov statistics, Pearson product-moment correlation coefficient and multiple regression analysis.	EI for female nurses mean (SD) of 5.3 (0.57)<br>- Emotionality 5.3 (0.73)<br>- Sociability 4.9 (0.71)<br>- Self-control 5.0 (0.72)<br>- well-being 5.9 (0.64)<br><br>EI for male nurses mean (SD) of 5.2 (0.59)<br>- Emotionality 5.0 (0.78)<br>- Sociability 4.8 (0.71)<br>- Self-control 4.9 (0.71)<br>- well-being 5.8 (0.62)<br><br>Both female and male nurses exhibit above average global EI scores. Females score above average all EI subscales. Males	85.71%

	score above average on all subscale scores, with the exception of sociability with they are below average.

**Al Huseini (2019) Trait Emotional Intelligence and Its Correlates in Oman Medical Specialty Board Residents**	Medical Residents (n=320)<br>Males (n=101)<br>Females (n=219)	Emotional Intelligence	TEIQue-SF	<u>Country:</u><br>Oman<br><u>Method of Collection:</u><br>Random selection (via randomisation software) of residents within the Oman Medical Speciality Board (OMSB) training program. Data collection took places during a mandatory OMSB workshop.<br><u>Analysis:</u><br>Comparisons completed by t-tests and ANOVA. Correlations calculated via Person product-moment coefficient. Predictors calculated via multiple linear regressions.	Global medical resident EI mean (SD) scores of 4.20 (0.42)<br>Medical resident EI Subscale mean (SD) scores:<br>- Well-being 4.57 (0.67)<br>- Self-control 4.29 (0.72)<br>- Emotionality 3.75 (0.70)<br>- Sociability 4.31 (0.61)<br><br>Medical residents exhibit high EI subscale scores of well-being, sociability, self-control and emotionality.	85.71%

**Al-Alawi (2017) Influence of Eysenckian Personality Traits in Choice of Specialization by Young Omani Doctors**	Doctors (n=255)<br>Males (n=103)<br>Females (n=152)<br><br>Surgical (n=95)<br>Medical (n=134)<br>Diagnostic (n=26)	Personality	Eysenck Personality Questionnaire–Revised (EPQ-R)	Country:<br>Oman<br>Method of Collection:<br>Voluntary survey completed while attending mandatory workshop organised by Oman Medical Specialty Board.<br>Analysis:<br>descriptive statistics, ANOVA	Resident personality traits mean (SD) by speciality and subscale:<br><br>Surgical Residents<br>- Control 10.8 (3.6)<br>- Extroversion 12.7 (4.3)<br>- Psychotism 6.5 (2.5)<br>- Neuroticism 9.3 (5.4)<br><br>Medical Residents<br>- Control 11.6 (3.3)<br>- Extroversion 11.8 (4.1)<br>- Psychotism 5.7 (1.9)<br>- Neuroticism 8.5 (5.4)<br><br>Diagnostic Residents<br>- Control 10.7 (3.1)<br>- Extroversion 12.6 (3.9)<br>- Psychotism 5.4 (1.7)<br>- Neuroticism 8.8 (5.4)<br><br>Mean psychoticism trait score of 6.5±2.5 for surgical specialties was significantly higher than that of medical and diagnostic specialties	100.00%

**Albendín-García (2022) Explanatory Models of Burnout Diagnosis Based on Personality Factors in Primary Care Nurses**	Nurses (n=242)<br>Males (n=109)<br>Females (n=133)	Personality	NEO Five-Factor Inventory (NEO FFI)<br>Maslach Burnout Inventory - 22 item	<u>Country:</u><br>Spain<br><u>Method of Collection:</u><br>Nurses working in Andalusian Health Service.<br><u>Analysis:</u><br>Bivariate and tri-variate exploratory analysis, categorical-response ordinal logistic regression.	Nursing personality mean (SD) by subscale:<br>- neuroticism 28.26 (8.20)<br>- friendliness 44.72 (7.65)<br>- responsibility 46.60 (7.22)<br>- extraversion 42.01 (7.88)<br>- openness 39.08 (6.72)<br><br>Nurses have high levels of neuroticism and responsibility, with love levels of extraversion and friendliness.	57.14%

**Al-Dlaigan (2017) Study on Personality Types of Dentists in different Disciplines of Dentistry**	Dentists (n=243)<br>Male (n=152)<br>Female (n=91)<br><br>Paediatric dentistry (n=37)<br>Periodontics (n=36)<br>Endodontics (n=32)<br>Prosthodontics (n=36)<br>Orthodontist (n=30)<br>Restorative dentistry (n=41)<br>Oromaxillofacial surgery (n=31)	Personality	Myers–Briggs Type Indicator (MBTI)	Country:<br>Saudi Arabia<br>Method of Collection:<br>Face-to-face distribution and collection of survey to specialist dentistry staff working in Riyadh Sity, Saudi Arabia.<br>Analysis:<br>Descriptive statistics	Specialist Dentist percentage personality type by subscale:<br>- ISTJ 54%<br>- ISFJ 2%<br>- INFJ 1%<br>- ISTP 0.4%<br>- INFP 4%<br>- INTP 3%<br>- ENFP 17%<br>- ENTP 8%<br>- ENFJ 8%<br>- ENTJ 2%<br><br>Specialist Dentist percentage personality characteristic by subscale:<br>- extroversion 35%<br>- introversion 65%<br>- sensing 57%<br>- intuition 43%<br>- thinking 67%<br>- feeling 33%<br>- judgement 68%<br>- perception 32%	85.71%

**Al-Hamdan (2017) Correlating Emotional Intelligence and Job Performance Among Jordanian Hospitals' Registered Nurses.**	Registered Nurses (n=194)<br>Males (n=88)<br>Females (n=109)	Emotional Intelligence	Genos EI Assessment Scale	<u>Country:</u><br>Jordan<br><u>Method of Collection:</u><br>Self-questionnaires to RN's of six selected hospitals (2 public, 2 private, 2 teaching hospitals).<br><u>Analysis:</u><br>Analysis was completed on both measures and relationship between them utilising ANOVA, independent t-tests and multiple regressions.	Global registered nurses mean (SD) of EI is 3.49 (0.61).<br>Registered nurses subscale scores of EI mean (SD):<br>- Recognising and expressing emotions 3.51 (0.745)<br>- Understanding others emotions 3.42 (0.756)<br>- Decision making 3.51 (0.710)<br>- Managing emotions 3.50 (0.715)<br>- Controlling emotions 3.48 (0.726)<br><br>21% of nurses demonstrated low EI, 59% mid-range and 21% high EI scores.<br>Small positive correlation between job performance and 4 subscales (recognizing and expressing emotions, understanding others emotions, decision making and managing emotions).	100.00%

**Al-Hamdan (2019) The impact of emotional intelligence on conflict management styles used by Jordanian nurse managers**	Nurse managers (n=248)<br>Males (n=106)<br>Females (n=142)	Emotional Intelligence	Genos 31-item questionnaire	<u>Country:</u><br>Jordan<br><u>Method of Collection:</u><br>Invitation letter sent to eligible nurse managers over a 3 month period.<br><u>Analysis:</u><br>Descriptive statistics, persons correlations, independent t-tests and one-way ANOVA.	Global nurse mangers mean (SD) of EI 3.63 (0.49).<br><br>Nurse mangers subscale scores of EI mean (SD):<br>- self-awareness 3.61 (0.65)<br>- emotional expression 3.55 (0.62)<br>- self-control 3.71 (0.60)<br>- emotional self-management 3.77 (0.65)<br>- emotional awareness of others 3.75 (0.64)<br>- emotional management of others 3.45 (0.68)<br>- emotional reasoning 3.60 (0.66)<br><br>EI has a positive influence on nurse managers conflict management style.	100.00%

**Al-Hamdan (2020) Emotional intelligence and intent to stay among nurses employed in Jordanian hospitals**	Nurses (n=280)<br>Males (n=139)<br>Females (n=141)	Emotional Intelligence	Wong and Law Emotional Intelligence Scale (WLEIS) - 4 point scale	<u>Country:</u><br>Jordan<br><u>Method of Collection:</u><br>Data collected from university, government and private tertiary hospitals in Jordan. Hospitals were randomly selected that had a minimum of 300 registered nurses and bd platform of 200.<br><u>Analysis:</u><br>Descriptive statistics, Pearson's, r tests for examine relationships.	Nurses EI mean (SD) by subscale:<br>- self-emotion appraisal 3.1 (0.5)<br>- others emotion appraisal 3.0 (0.5)<br>- use of emotion 3.1 (0.6)<br>- regulation of emotions 3.0 (0.6)<br><br>EI was found to have a positive relationship with nurses intent to stay.	100.00%
**Al-Hamdan (2021) The Relationship Between Emotional Intelligence and Nurse–Nurse Collaboration**	Nurses (n=311)<br>Males (n=116)<br>Females (n=15)	Emotional Intelligence	Self-Report Emotional Intelligence Scale (SREIS)	<u>Country:</u><br>Jordan<br><u>Method of Collection:</u><br>Eligible nurses from two hospitals were individually asked to participate.<br><u>Analysis:</u><br>Descriptive statistics, parsons correlations	Global EI of nurses was 3.36, which is considered a medium level.<br><br>EI was significantly correlated:<br>- positively and weakly with nursing collaboration for conflict management<br>- positively and moderately correlated with shared processing<br>- positively and moderately correlated with professionalism.	100.00%

**Ali (2017) High Level of Emotional Intelligence Is Related to High Level of Online Teaching Self-Efficacy among Academic Nurse Educators**	Academic nursing faculty (n=115)<br>Males (n=7)<br>Females (n=108)	Emotional Intelligence	Schutte's Emotional Intelligence Test (SSEIT)	<u>Country:</u><br>USA<br><u>Method of Collection:</u><br>Electronic survey sent to 300 administrators of accredited nursing schools, with instruction to forward to all faculty who teach online.<br><u>Analysis:</u><br>Descriptive statistics; relationships calculated using Pearson's correlations; difference between groups measured by one-way ANOVA.	Academic online teaching nursing faculty have a high total EI mean (SD) scores of 4.16 (0.36).<br><br>Moderate relationship between EI and online teaching self-efficacy. As EI increases so too does online teaching self-efficacy. Age is not an impactor, nor was level of degree attained.	85.71%

**Allen (2002) Work context, personal control, and burnout amongst nurses.**	Major public health services nurses (n=104)<br>Males (n=5)<br>Females (n=99)	Personality	NEO Five-Factor Inventory, 12 neuroticism items (NEO-FFI)	Country:<br>Australia<br>Method of Collection:<br>Questionnaires distributed by nursing administration of public health service in Victoria, Australia.<br>Analysis:<br>Nurses categorised as chronic or acute care group dependant on work context.<br>Group comparisons calculated via MANOVA. Relationships explored via correlations and hierarchal regressions.	Major public health nurses neuroticism mean (SD) scores:<br>- Chronic 4.00 (1.32)<br>- Acute 4.05 (1.28)<br>- Total 4.04 (1.29)<br><br>Nil difference in burnout and neuroticism between groups. Neuroticism significant correlation with exhaustion, cynicism and professional efficacy.	71.43%
**Al-Ruzzieh (2021) Impact of nurses' emotional intelligence on the implementation of a professional practice model in cancer care**	Nurses (n=580)<br>Males (n=241)<br>Females (n=339)	Emotional Intelligence	Wong and Law Emotional Intelligence Scale (WLEIS) - 6 point scale	Country:<br>Jordan<br>Method of Collection:<br>Eligible nurses at King Hussein Cancer Centre identified through staff database. Questionnaires delivered and distributed via web platform distributed via email.<br>Analysis:<br>Descriptive statistics, Pearson correlation coefficient test.	Nursing global EI mean (SD) of 5.60 (0.78) and subscale:<br>- self-emotion appraisal 5.59 (0.06)<br>- others emotion appraisal 5.38 (1.02)<br>- use of emotion 5.88 (0.87)<br>- regulation of emotions 5.54 (0.90)<br><br>EI is positive correlated with effective implementation of professional practice model.	85.71%

**Alshammari (2020) Emotional intelligence and authentic leadership among Saudi nursing leaders in the Kingdom of Saudi Arabia**	Nurse Leaders (n=152)<br>Males (n=53)<br>Females (n=99)	Emotional Intelligence	Emotional Intelligence Questionnaire	<u>Country:</u><br>Saudi Arabia<br><u>Method of Collection:</u><br>2 stage cluster sample, surveys emailed to participants.<br><u>Analysis:</u><br>Descriptive statistics, ANOVA, t-tests, Pearson’s correlations.	Nurses EI mean (SD) by gender:<br>- Male 2.108 (0.218)<br>- Female 2.027 (0.275)	85.71%
**Altuntaş (2022) The relationship between nurses' personality traits and their perceptions of management by values, organizational justice, and turnover intention**	Nurses (n=176)<br>Males (n=15)<br>Females (n=161)	Personality	Big Five Personality Scale (BFPS)	<u>Country:</u><br>Istanbul<br><u>Method of Collection:</u><br>Nurses at public university hospital between Jun - Sept 2016.<br><u>Analysis:</u><br>descriptive statistics, Pearson’s product-moment correlations	Nurses personality mean (SD) by trait:<br>- Extraversion 28.64 (5.02) - lower than average<br>- responsibility 35.25 (5.39) - higher than average<br>- Openness 36.20 (5.47) - higher than average<br>- Agreeableness 35.88 (4.81) - higher than average<br>- Neuroticism 25.57 (4.16) - lower than average.	85.71%

**Amenta (1984) Traits of hospice nurses compared with those who work in traditional settings.**	Hospice nurses (n=36)<br>Male (n=0)<br>Female (n=36)<br><br>Traditional setting nurses (n=35)<br>Male (n=0)<br>Female (n=35)	Personality	Myers-Briggs Type Indicator<br>16F Personality Indicator	<u>Country:</u><br>USA<br><u>Method of Collection:</u><br>Tools collected as part of orientation to service process between Feb 1979 and April 1981.<br><u>Analysis:</u><br>t-tests	Nurses <u>MBTI</u> mean (SD) scores of <u>extraversion/introversion</u>:<br>- Hospice 104.18 (21.87)<br>- Traditional 104.94 (24.82)<br>Nurses <u>MBTI</u> mean (SD) scores of <u>sensing/intuition</u>:<br>- Hospice 100.56 (25.64)<br>- Traditional 82.77 (26.51)<br>Nurses <u>MBTI</u> mean (SD) scores of <u>thinking/feeling</u>:<br>- Hospice 116.94 (17.76)<br>- Traditional 118.14 (20.25)<br>Nurses <u>MBTI</u> mean (SD) scores of <u>judgement/perception</u>:<br>- Hospice 100.65 (25.79)<br>- Traditional 89.77 (25.72)<br>Both slightly more introverted and higher feelings. Traditional more sensing. Nil significant differentiation for judgement/perception, traditional more inclined to judgement.<br><br>Nurses <u>16PF Traits</u> mean (SD) scores of <u>warmth</u>:<br>- Hospice 4.64 (2.03)<br>- Traditional 5.00 (1.71)<br>Nurses <u>16PF Traits</u> mean (SD) scores of <u>intelligence</u>:<br>- Hospice 7.11 (1.73)	71.43%

		- Traditional 6.57 (1.59)<br>Nurses 16PF Traits mean (SD) scores of ego strength:<br>- Hospice 5.69 (1.80)<br>- Traditional 5.17 (2.19)<br>Nurses 16PF Traits mean (SD) scores of assertiveness:<br>- Hospice 6.61 (1.57)<br>- Traditional 4.97 (2.43)<br>Nurses 16PF Traits mean (SD) scores of expressive:<br>- Hospice 5.36 (2.05)<br>- Traditional 5.85 (2.10)<br>Nurses 16PF Traits mean (SD) scores of conscientiousness:<br>- Hospice 5.27 (1.78)<br>- Traditional 6.02 (2.39)<br>Nurses 16PF Traits mean (SD) scores of venturesomeness:<br>- Hospice 5.91 (2.22)<br>- Traditional 5.31 (2.23)<br>Nurses 16PF Traits mean (SD) scores of sensitivity:<br>- Hospice 5.88 (2.13)<br>- Traditional 5.68 (1.51)<br>Nurses 16PF Traits mean (SD) scores of suspiciousness:<br>- Hospice 5.94 (1.99)<br>- Traditional 5.57 (2.51)<br>Nurses 16PF Traits mean (SD) scores of imagination:

- Hospice 5.63 (1.67)
- Traditional 4.62 (2.03)

Nurses 16PF Traits mean (SD) scores of forthrightness:

- Hospice 3.53 (2.29)
- Traditional 4.85 (2.29)

Nurses 16PF Traits mean (SD) scores of guilt prowess:

- Hospice 5.50 (1.92)
- Traditional 5.82 (1.74)

Nurses 16PF Traits mean (SD) scores of radicalism:

- Hospice 6.16 (1.65)
- Traditional 5.00 (2.06)

Nurses 16PF Traits mean (SD) scores of self-sufficiency:

- Hospice 5.77 (1.89)
- Traditional 5.68 (2.29)

Nurses 16PF Traits mean (SD) scores of control:

- Hospice 5.69 (1.56)
- Traditional 6.00 (1.92)

Nurses 16PF Traits mean (SD) scores of tension:

- Hospice 6.14 (1.90)
- Traditional 6.40 (1.67)

Nurses 16PF Traits mean (SD) scores of extraversion:

- Hospice 6.77 (1.70)
- Traditional 6.60 (1.92)

Nurses 16PF Traits mean (SD)

			scores of anxiety:<br>- Hospice 5.68 (1.72)<br>- Traditional 6.19 (1.57)<br>Nurses 16PF Traits mean (SD) scores of poise:<br>- Hospice 6.27 (1.84)<br>- Traditional 5.71 (2.03)<br>Nurses 16PF Traits mean (SD) scores of independence:<br>- Hospice 5.74 (1.55)<br>- Traditional 4.13 (1.74)<br>Hospice nurses more assertive, imaginative, radical, forthright, free-thinking and independent than traditional.

**Ang (2016) Demographics and Personality Factors Associated with Burnout among Nurses in a Singapore Tertiary Hospital.**	Nurses (n=1830)<br>Males (n=72)<br>Females (n=1002)	Personality	NEO Five-Factor Inventory (NEO-FFI)	<u>Country:</u><br>Singapore<br><u>Method of Collection:</u><br>All nurses at 1600 bed tertiary hospital eligible for study, surveys circulated at staff meeting, with reminder email for completion at 2 weeks.<br><u>Analysis:</u><br>Analysed personality and influence on burnout using univariable and multivariable logistic regressions.	% of nurse population <u>personality (NEO-FFI) traits</u>:<br>- low or average in Neuroticism 72%<br>- average or high in Extraversion 82%<br>- average or high in Openness 79%<br>- average or high in Agreeableness 77%<br>- average or high in Conscientiousness 81%<br><br>High neuroticism = increased EE and DP, and low PA<br>Low extraversion, agreeableness and conscientiousness (compared to avg) = high EE, high DP and low PA	85.71%

**Anitha (2022) How organizational citizenship behaviour is promoted among nurses: A moderated mediation model**	Nurses (n=311)<br>Males (n=unknown)<br>Females (n=unknown)	Personality	International Personality Item Pool (IPIP)	<u>Country:</u><br>India<br><u>Method of Collection:</u><br>Nurses at 5 hospitals in Chennai, Southern India, who had been working in the last 3 months. Surveys delivered at end of shift between Jun-Sep 2019.<br><u>Analysis:</u><br>descriptive statistics, correlations.	Nurses extraversion mean (SD) 3.54 (0.76)	100.00%
**Apore (2019) Emotional intelligence, gender and transformational leadership among nurses in emerging economies**	Nurse (n=310)<br>Males (n=104)<br>Females (n=206)	Emotional Intelligence	Wong and Law Emotional Intelligence Scale (WLEIS)	<u>Country:</u><br>Ghana<br><u>Method of Collection:</u><br>Nurses at 5 hospitals in greater Accra region of Ghana,<br><u>Analysis:</u><br>descriptive statistics, multiple regression	Nurse EI mean (SD) by subscale:<br>- self-emotional appraisal 3.5 (1.68)<br>- others emotional appraisal 3.32 (1.31)<br>- use of emotion 3.17 (1.43)<br>- regulation of emotion 2.86 (1.30)<br><br>Where mean score from 1 to 2.33 indicates a low level, 2.34 to 3.67 indicates moderate and then 3.68 to 5 is high level.	100.00%

**Aradilla-Herrero (2014) Perceived emotional intelligence in nursing: psychometric properties of the Trait Meta-Mood Scale**	Nurses (n=209)<br>Males (n=unknown)<br>Females (n=unknown)	Emotional Intelligence	Trait Meta Mood Scale (TMMS-24)	<u>Country:</u><br>Spain<br><u>Method of Collection:</u><br>Written paper form.<br><u>Analysis:</u><br>Descriptive statistics, factor analysis	Nursing EI mean (SD) by subscale:<br>- emotional attention 27.82 (5.11)<br>- emotional clarity 30.34 (4.61)<br>- emotional repair 30.58 (4.81)	85.71%
**Asiamah (2017) Enhancing nurses' emotional intelligence: Are tenure prolongation, education and in-service training applicable methods even when not specialized?**	Healthcare institution nurses (clinics, polyclinics, and hospitals) (n=510)<br>Males (n=236)<br>Females (n=274)	Emotional intelligence	Schutte's Emotional Intelligence Test (SSEIT)	<u>Country:</u><br>Ghana<br><u>Method of Collection:</u><br>Randomised sample of nurses working in registered healthcare institutions controlled by Ghana Health Service (GHS).<br>Surveys were hand delivered and collected over a 28 working day period.<br><u>Analysis:</u><br>Data validated and reliability and outliers via confirmatory factor analysis. Explored the relationship between education, tenure and IST method of Inservice training with emotional intelligence with Pearson's correlations	Global nurses mean (SD) of EI is 3.816 (1.001).<br><br>Education, IST and tenure do not significantly predict the development of EI in nurses. Argued that EI only develops over time if exposed to an environment that supports the improvement of EI.	85.71%

**AsİKarakaŞ (2020) A study to determine the relationship between the emotional intelligence levels and perceptions of spiritual support of nurses working in a hospital for psychiatric and neurological diseases in Turkey**	Nurses (n=159)<br>Males (n=59)<br>Females (n=98)	Emotional Intelligence	Emotional Intelligence Assessment Scale (EIA)	Country:<br>Turkey<br>Method of Collection:<br>Nurses working in hospital between Dec 2016 - April 2017<br>Analysis:<br>descriptive statistics, Pearson's correlations.	Nurses global EI mean (SD) 133.40 (32.28) (score range 53-172) and by subscale:<br>- awareness of emotions 27.38 (7.99) (score range 8-36)<br>- managing one's emotions 25.49 (6.57) (score range 8-36)<br>- motivating oneself 27.35 (7.34) (score range 10-36)<br>- empathy 27.39 (7.13) (score range 9-36)<br>- social skills 25.77 (6.40) (score range 10-36)	100.00%
**AugustoLanda (2008) The relationship between emotional intelligence, occupational stress and health in nurses: a questionnaire survey.**	Nurses (n=180)<br>Males (n=46)<br>Females (n=134)	Emotional intelligence	Trait Meta Mood Scale (TMMS)	Country:<br>Spain<br>Method of Collection:<br>Questionnaires distributed and collected by 3 data collectors of nurses working at the public hospital on the day. Questionnaires were completed in their workplace and returned at the end of the day.<br>Analysis:<br>Relationship between stress and EI; and health and EI were	Nursing EI subscale interpersonal factors mean (SD):<br>- Emotional attention 24.30 (5.50)<br>- Emotional clarity 26.68 (6.03)<br>- Emotional repair 27.69 (6.06)<br><br>Nurses with higher emotional clarity and repair report less stress.<br>Nurses with high scores in emotional attention experience greater stress.	100.00%

analysed with t-tests, post hoc
and regression analysis

**Ayuso-Murillo (2017) Communication skills in ICU and adult hospitalisation unit nursing staff.**	ICU and adult medical admission nurses (n=69)<br><br>ICU (n=29)<br>Males (n=3)<br>Female (n=26)<br><br>Adult medical admission (n=40)<br>Males (n=6)<br>Females (n=34)	Personality	16F Personality Indicator - 5	Country:<br>Spain<br>Method of Collection:<br>Questionnaires distributed via nursing management team in 2 hospitals over 4 month period.<br>Analysis:<br>Comparison between groups personality and communication via t-test and/or Mann-Whitney U-test.	Nurses personality primary factor mean (SD) scores of warmth:<br>- ICU 5.6 (1.9)<br>- adult medical admissions 6 (1.4)<br>Nurses personality primary factor mean (SD) scores of reasoning:<br>- ICU 6.1 (1.5)<br>- adult medical admissions 6.1 (1.2)<br>Nurses personality primary factor mean (SD) scores of stability:<br>- ICU 5.2 (1.3)<br>- adult medical admissions 4.8 (1.1)<br>Nurses personality primary factor mean (SD) scores of dominance:<br>- ICU 4.6 (1.8)<br>- adult medical admissions 6.4 (1.6)<br>Nurses personality primary factor mean (SD) scores of liveliness:<br>- ICU 5.3 (1.6)<br>- adult medical admissions 4.9 (1.9)<br>Nurses personality primary	71.43%

factor mean (SD) scores of rule-consciousness:
- ICU 5.6 (1.5)
- adult medical admissions 5.4 (1.4)

Nurses personality primary factor mean (SD) scores of boldness:
- ICU 5.1 (2.1)
- adult medical admissions 5.4 (1.6)

Nurses personality primary factor mean (SD) scores of sensitivity:
- ICU 6.6 (1.6)
- adult medical admissions 6.7 (1.7)

Nurses personality primary factor mean (SD) scores of vigilance:
- ICU 5.5 (1.5)
- adult medical admissions 5.6 (1.6)

Nurses personality primary factor mean (SD) scores of abstraction:
- ICU 5.2 (1.4)
- adult medical admissions 5.6 (1.6)

Nurses personality primary factor mean (SD) scores of

privateness:
- ICU 5.9 (2.2)
- adult medical admissions 5.9 (1.7)

Nurses personality primary factor mean (SD) scores of apprehension:
- ICU 6.2 (1.6)
- adult medical admissions 6.3 (1.8)

Nurses personality primary factor mean (SD) scores of openness to change:
- ICU 5.3 (1.7)
- adult medical admissions 5.8 (1.5)

Nurses personality primary factor mean (SD) scores of self-reliance:
- ICU 6.2 (1.5)
- adult medical admissions 6.3 (1.7)

Nurses personality primary factor mean (SD) scores of perfectionism:
- ICU 4.9 (1.2)
- adult medical admissions 5.5 (1.6)

Nurses personality primary factor mean (SD) scores of tension:

- ICU 5.7 (1.6)
- adult medical admissions 6.4 (1.5)

Nurses personality global factor mean (SD) scores of extroversion:
- ICU 5.4 (2.2)
- adult medical admissions 5.6 (2)

Nurses personality global factor mean (SD) scores of tough-mindedness:
- ICU 6.1 (1.7)
- adult medical admissions 6.6 (1.9)

Nurses personality global factor mean (SD) scores of anxiety:
- ICU 5.5 (2)
- adult medical admissions 4.8 (1.7)

Nurses personality global factor mean (SD) scores of independence:
- ICU 4.6 (1.9)
- adult medical admissions 5.9 (1.9)

Nurses personality global factor mean (SD) scores of self-control:
- ICU 5.6 (1.6)
- adult medical admissions 6.3

	(1.8)<br>Personality factors associated with communication skills in ICU nurses are below those of adult medical admission nurses.

**Bagley (2018) Personality, Work-Life Balance, Hardiness, and Vocation: A Typology of Nurses and Nursing Values in a Special Sample of English Hospital Nurses**	Nurses (n=192)<br>Males (n=unknown)<br>Females (n=unknown)	Personality	Maslach's Burnout Inventory<br>The Short Hardiness Inventory<br>Big 5 Personality Inventory	Country:<br>United Kingdom<br>Method of Collection:<br>Longitudinal study of English nurses through snowball sampling method. Surveys were completed on laptop provided by researcher<br>Analysis:<br>Correlations of variables were used to establish predictability of nursing values and intention to leave; and therefore level of support required within the profession.	English nurses personality mean (SD) of:<br>- neuroticism 2.49 (0.76)<br>- extraversion 3.49 (0.74)<br>- openness 3.74 (0.59)<br>- agreeableness 3.87<br>- conscientiousness 3.76 (0.54)<br><br>English nurses total hardiness mean (SD) of 33.85 (8.5)<br><br>English nurses burnout mean (SD) by subscale:<br>- emotional exhaustion 2.02 (0.58)<br>- depersonalisation 1.78 (0.50)<br>- lack of achievement 28.89 (5.50)<br><br>Posited four types of nurses based on the outcome measure scores and correlations:<br>- **"the soldiers":** medium scores on most measures; some burnout; fewer intentions to leave nursing, but more work-life stress; somewhat lower scores on agreeable personality, and lower scores on nursing values scale.	85.71%

					- **"cheerful professionals":** higher job rank; more extraverted; more agreeable; better self-esteem; not depressed; few plans to leave nursing; medium-to-low scores on neuroticism and depression; good self-esteem; middle range hardy personality scale score; somewhat higher attachment to core nursing values.<br>- **"high stressed, potential leavers"**: high levels of depression and neuroticism; poor self-esteem; less extraverted; low "hardy personality" profiles; experiencing work-life stress; somewhat lesser attachment to core nursing values.<br>- **"high achievers, strong and stable"**: higher job rank; more extraverted; lower scores on neuroticism and depression measures; higher scores on hardiness and self-esteem; less work-life stress; less burnout; higher nursing values profile.

**Baglioni (1990) Job stress, mental health and job satisfaction among UK senior nurses.**	Senior Nurses (n=475)<br>Males (n=0)<br>Females (n=475)	Behaviour	Bortner Measure of Type A behaviour	<u>Country:</u><br>United Kingdom<br><u>Method of Collection:</u><br>Surveys were sent to all nurse managers in one national health service authority in southwest of England.<br><u>Analysis:</u><br>Descriptive statistics, Cronbach's alphas	Mean (SD) type A behaviour of senior nurses was 94.2 (17.2), slightly more Type A traits than the normal population.	71.43%
**Bailey (1988) Occupational Therapy Administrators and Clinicians: Differences in Demographics and Values: Occupation, Participation and Health**	Occupational Therapists (n=385)<br>Males (n=unknown)<br>Females (n=unknown)<br><br>Administrators (n=201)<br>Clinicians (n=184)	Behaviour	Rokeach Values Survey	<u>Country:</u><br>USA<br><u>Method of Collection:</u><br>Administrators and clinicians with > 5 years' experience were eligible. Randomly selected form American Occupational Therapy Association registration list.<br><u>Analysis:</u><br>Descriptive statistics, chi-squares, multivariate analysis to determine differences between groups. Stepwise multiple regression analysis determine variance factors.	Administrators demonstrated higher values in capability and sense of accomplishment than clinicians.<br>Clinicians demonstrated higher values of lovingness, mature love and inner harmony.	85.71%

**Ball (2015) A cross-sectional exploration of the personality traits of dietitians.**	Dietitians (n=346)<br>Males (n=18)<br>Females (n=328)	Personality	Temperament and Character Inventory (TCI-R 140)	Country:<br>Australia<br>Method of Collection:<br>Online survey distributed to Dietician Connection members via their newsletter and social media pages for 2 month period.<br>Analysis:<br>Descriptive statistics comparison to population norms, relationships between characteristic and personality traits using multivariate analyses.	Dietician temperament and character trait mean (SD) / rank against pop norms scores:<br>- Novelty seeking 54.14 (9.68) / average<br>- Harm avoidance 57.05 (13.65) / high<br>- Reward Dependence 70.55 (9.81) / high<br>- Persistence 73.48 (10.79) / high<br>- Self-directedness 74.30 (11.71) / high<br>- Cooperativeness 80.70 (9.68) / high<br>- Self-transcendence 42.34 (10.86) / low<br><br>Compared to pop norms, dieticians display average novelty-seeking, high harm avoidance, reward dependence, persistence, self-directedness and cooperativeness and low-levels of self-transcendence.	85.71%

**Ball (2016) Association between dietitians' personality profiles and practice areas.**	Dieticians (n=346)<br>Males (n=18)<br>Females (n=328)<br><br>Profile A (n=122)<br>Profile B (n=96)<br>Profile C (n=103)	Personality	Temperament and Character Inventory (TCI-R 140)	Country:<br>Australia<br>Method of Collection:<br>Online survey<br>Analysis:<br>Descriptive statistics, where means were clustered into 3 group profiles (A,B & C). Pearson's chi-squares. Univariate and multivariate analysis to determine relationships.	All dieticians profiles demonstrated complimentary traits of high reward dependence and persistence. However differences between profiles:<br>- Profile A: highest reward dependence and self-transcendence<br>- Profile B: lowest in harm avoidance and highest in persistence and self-directness<br>- Profile C: highest in harm avoidance and lowest in self-directness	71.43%
**Bamberger (2017) A pilot study of an emotional intelligence training intervention for a paediatric team.**	Paediatric Doctors and Nurses (n=38)<br>Males (n=unknown)<br>Females (n=unknown)<br><br>Doctors (n=28)<br>Intervention (n=17)<br>Control (n=11)<br><br>Nurses (n=10)<br>Intervention	Emotional Intelligence	Bar-On's Emotional Quotient Inventory (EQ-I)	Country:<br>Northern Israel<br>Method of Collection:<br>Employees of paediatric unit of large metropolitan teaching hospital were invited to participate in the study.<br>Analysis:<br>Pre-post intervention comparisons with Kruskal-Wallis tests. Differences between staff types with Mann-Whitney U test.	Total intervention group baseline EI mean (SD) of 99.0 (9.6) (slightly below mean standard measure) and composite subscale scores of:<br>- Intrapersonal 98.1 (11.5) - slightly below standard mean measure<br>- adaptability 97.1 (9.1) - slightly below standard mean measure<br>- stress management 100.4 (12.0) - slight above standard mean measure<br>- general mood 100.6 (9.2) - slightly below standard mean	85.71%

	(n=10)<br>Control (n=0)				measure<br>- interpersonal 102.6 (9.2) - slightly below standard mean measure	
**Barr (2018a) Personality Traits, State Positive and Negative Affect, and Professional Quality of Life in Neonatal Nurses.**	Neonatal Nurses (n=142)<br>Males (n=3)<br>Females (n=139)	Personality	Core Self-evaluations Scale (CSES)<br>Big Five Inventory (BFI)	<u>Country:</u><br>Australia<br><u>Method of Collection:</u><br>SurveyMonkey distributed to four Level 3-4 NICU nursing staff via email, posters and in-service meetings in New South Wales, Australia. Survey open for 4 months between Feb-May 2016.<br><u>Analysis:</u><br>Hierarchical multiple regression analysis.	Neonatal Nurses core self-evaluation mean (SD) of 3.5 (0.52).<br><br>Neonatal Nursed personality BFI subscale mean (SD):<br>- Neuroticism 2.7 (0.70)<br>- Agreeableness 4.0 (0.47)<br>- Extraversion 3.2 (0.1)<br>- Conscientiousness 4.0 (0.48)<br>- Openness 3.4 (0.49)<br><br>Nurses with higher CSES = increased confidence in abilities and security in themselves to be successful at work = less burnout.	85.71%

					Agreeableness moderately strong correlation with burnout, but weak correlation with CSES.	
**Barr (2018b) The five-factor model of personality, work stress and professional quality of life in neonatal intensive care unit nurses.**	Neonatal ICU nurses (n=140)<br>Males (3%)<br>Females (97%)	Personality	Big Five Inventory	<u>Country:</u><br>Australia<br><u>Method of Collection:</u><br>Registered nurses in 4 of the 8 level 3-4 NICU's in NSW received email, posters and in services with link to online survey.<br><u>Analysis:</u><br>Descriptive statistics, bivariate correlations, hierarchical multiple linear regressions and moderation and deviation analyses.	Nurses personality traits mean (SD) by subscale:<br>- Conscientiousness 4.1 (0.46)<br>- Agreeableness 4.0 (0.47)<br>- Extraversion 3.3 (0.71)<br>- Neuroticism 2.7 (0.69)<br>- Openness 3.4 (0.49)<br><br>Neuroticism and agreeableness were related to burnout<br>Neuroticism was related to secondary traumatic stress<br>Extraversion was related to compassion satisfaction	85.71%

**Başoğul (2016) Role of Emotional Intelligence in Conflict Management Strategies of Nurses.**	Nurses (n=277)<br>Males (n=unknown)<br>Females (n=unknown)	Emotional Intelligence	Bar-On's Emotional Quotient Inventory (EQ-I)	<u>Country:</u><br>Turkey<br><u>Method of Collection:</u><br>Stratified random sample from two units (internal and surgical) in a university hospital. Researchers hand-delivered questionnaires and collected 1 week later over a 3 month period.<br><u>Analysis:</u><br>Frequency distribution, percentage distribution, means with t-tests significant between variables and variance analysis.	Total emotional intelligence of nurses mean (SD) 2.75 (0.19).<br><br>EQI total meta factor of <u>Intrapersonal skills</u> mean (SD) of 2.82 (0.25) and subscales scores of:<br>- emotional self-awareness 2.44 (0.4)<br>- self-regard 2.82 (0.52)<br>- assertiveness 2.41 (0.42)<br>- self-actualization 2.8 (0.4)<br>- independence 3.62 (0.65)<br>EQI total meta factor of <u>Interpersonal skills</u> mean (SD) of 2.5 (0.28) and subscales scores of:<br>- empathy 2.49 (0.42)<br>- interpersonal relationships 2.54 (0.38)<br>- social responsibility 2.46 (0.38)<br>EQI total meta factor of <u>Adaptability</u> mean (SD) of 2.81 (0.31) and subscales scores of:<br>- problem solving 2.36 (0.41)<br>- reality testing 3.26 (0.5)<br>- flexibility 2.8 (0.45)<br>EQI total meta factor of <u>Stress Management</u> mean (SD) of 2.99 (0.38) and subscales	100.00%

	scores of:<br>- stress tolerance 2.63 (0.42)<br>- impulse control 3.35 (0.64)<br>EQI total meta factor of General Mood mean (SD) of 2.64 (0.29) and subscales scores of:<br>- happiness 2.88 (0.43)<br>- optimism 2.39 (0.42)<br><br>Significant, but weak correlation in same direction between integrating, obliging, donating and compromising conflict management strategies and emotional intelligence scores.<br>Significant weak negative correlation between avoiding strategy and emotional intelligence scores.

**Bean (1993) Personality types of oncology nurses.**	Oncology Nurses (n=40)<br>Males (n=1)<br>Females (n=39)	Personality	Personal Style Inventory (PSI)	<u>Country:</u><br>USA<br><u>Method of Collection:</u><br>Convenience sample attained from Alabama Division of the American Cancer Society mailed to 100 oncology nurses. Coded surveys and responses returned were include in the study.<br><u>Analysis:</u><br>Descriptive statistics	Oncology nurses percentage personality dimension of Introvert/Extravert:<br>- Introvert 65.0%<br>- Extravert 25.0%<br>- Balanced 10.0%<br>Oncology nurses percentage personality dimension of sensing/intuition:<br>- Sensing 57.5%<br>- Intuition 25.0%<br>- Balanced 17.5%<br>Oncology nurses percentage personality dimension of Feeling/Thinking:<br>- Feeling 55.0%<br>- Thinking 20.0%<br>- Balanced 25.0%<br>Oncology nurses percentage personality dimension of Perceiving/Judging:<br>- Perceiving 20.0%<br>- Judging 50.0%<br>- Balanced 30.0%<br>Oncology nurses percentage personality types:<br>- ISFJ 15.0%<br>- ESFJ 7.5%<br>- ENFP 7.5%<br>- ISTJ 5.0%<br>- INFJ 2.5%	71.43%

- ISFP 2.5%
- INFP 2.5%
- ESFP 2.5%
- ESTJ 2.5%

Nil Oncology personality types in INTJ, ISTP, INTP, ESP, ENTP, ENFJ and ENTJ.

**Bean (1995)**<br>**Personality types of adult nurse practitioners.**	Nurse Practitioner (n=57)<br>Males (n=0)<br>Females (n=57)	Personality	Myers–Briggs Type Indicator (MBTI)	<u>Country:</u><br>USA<br><u>Method of Collection:</u><br>Subjects' names and addresses were obtained from a mailing list of graduates of a master's ANP program and completed surveys sent back.<br><u>Analysis:</u><br>Profile of personality through frequency of personality dimension and type.	Adult nurse practitioners percentage <u>personality dimension</u> of <u>Introvert/Extravert</u>:<br>- Introvert 42.1%<br>- Extravert 40.4%<br>- Balanced 17.5%<br>Adult nurse practitioners percentage <u>personality dimension</u> of <u>sensing/intuition</u>:<br>- Sensing 56.1%<br>- Intuition 22.8%<br>- Balanced 21.1%<br>Adult nurse practitioners percentage <u>personality dimension</u> of <u>Feeling/Thinking</u>:<br>- Feeling 36.8%<br>- Thinking 31.6%<br>- Balanced 31.6%<br>Adult nurse practitioners percentage <u>personality dimension</u> of <u>Perceiving/Judging</u>:<br>- Perceiving 7.0%<br>- Judging 86.0%<br>- Balanced 7.0%<br>Adult nurse practitioners percentage <u>personality types</u>:<br>- ISTJ 28.6%<br>- INFJ 23.8%<br>- ISFJ 9.5%	85.71%

- ESFJ 9.5%
- ENFJ 9.5%
- ESTJ 9.5%
- ENTJ 4.8%
- ENFP 4.8%

**Beierle (2019) Evaluating and Exploring Variations in Surgical Resident Emotional Intelligence and Burnout**	Surgical Residents (n=86)<br>Males (n=63)<br>Females (n=23)	Emotional Intelligence	Scale of Emotional Functioning: Health Service Provider (SEF:HSP)	<u>Country:</u><br>USA<br><u>Method of Collection:</u><br>Survey completed during resident orientation.<br><u>Analysis:</u><br>Pearson correlation, descriptive statistics	Resident EI mean (CI) by three intake groups:<br>- Administration 1: 3.69 (3.54-3.83)<br>- Administration 2: 3.91 (3.75-4.06)<br>- Administration 3: 3.96 (3.80-4.12)	85.71%
**Bergmueller (2018) Relationship between Burnout Syndrome and Personality Characteristics in Emergency Ambulance Crew**	Paramedics (n=97)<br>Males (n=40)<br>Females (n=57)<br><br><u><35 years</u> (n=47)<br>Males (n=22)<br>Females (n=25)<br><br><u>35-45 years</u> (n=23)<br>Males (n=8)<br>Females (n=15)<br><br><u>>45 years</u> (n=27)<br>Males (n=10)<br>Females (n=17)	Personality	Freiburg Personality Inventory (FPI)	<u>Country:</u><br>Germany<br><u>Method of Collection:</u><br><br><u>Analysis:</u><br>Relationship between variables were identified by correlations and regression analysis.	Personality findings for age <35 years:<br>- mental balance, extraversion-introversion and masculinity-femininity significantly higher for this age group.<br>Personality findings for age 35-45 years:<br>- nil significant<br>Personality findings for age >45 years:<br>- decreased level of spontaneous aggression compared to 35-45.<br><br>Positive correlation of neuroticism, depressively, emotional liability and EE and D.<br>Positive correlation Increased spontaneous aggression associated with increased D.	57.14%

		Negative correlation between sociability and mental balance and EE and D.

**Bidlan (2014a) Occupational stress, burnout, coping and emotional intelligence: Exploring gender differences among different occupational groups of healthcare professionals**	Nursing, Doctors and Support staff (n=600)<br>Males (n=unknown)<br>Females (n=unknown)	Emotional Intelligence	Multimeasure of Emotional intelligence (MMEI)	Country:<br>India<br>Method of Collection:<br>Questionnaires were hand delivered to selected private hospitals personnel, who distributed to health professionals.<br>Analysis:<br>Comparison between groups and measures via ANOVA.	Healthcare Professionals EI mean (SD) for Self-awareness:<br>- Doctors 50.62 (3.52)<br>- Nursing 48.71 (2.95)<br>- Support Staff 51.63 (3.17)<br>Healthcare Professionals EI mean (SD) for Managing Emotions:<br>- Doctors 52.94 (3.64)<br>- Nursing 51.05 (2.82)<br>- Support Staff 51.52 (3.60)<br>Healthcare Professionals EI mean (SD) for Motivating Oneself:<br>- Doctors 52.97 (3.25)<br>- Nursing 51.31 (3.07)<br>- Support Staff 51.68 (3.38)<br>Healthcare Professionals EI mean (SD) for Empathy:<br>- Doctors 51.36 (3.51)<br>- Nursing 51.77 (3.44)<br>- Support Staff 52.01 (3.96)<br>Healthcare Professionals EI mean (SD) for Handling Relationships:<br>- Doctors 52.23 (3.19)<br>- Nursing 49.74 (2.93)<br>- Support Staff 50.30 (3.12)	57.14%

**Bidlan (2014b) Emotional intelligence among healthcare professionals: Exploring its moderating effect in occupational stress and burnout relationship**	Healthcare Professionals (n=600)<br>Males (n=300)<br>Females (n=300)	Emotional intelligence	Multidimensional Measure of Emotional Intelligence (MMEI)	<u>Country:</u><br>India<br><u>Method of Collection:</u><br>Hand delivery of surveys to private hospitals in Delhi. Any healthcare professional with minimum 4-5 years in current position eligible for study.<br><u>Analysis:</u><br>Descriptive statistics, moderate regressions exploring effect of emotional intelligence on stress and burnout.	Total Health profession EI scores ranged from 217 - 282 with mean of 255.48<br><br>Significant moderating effect in relationship between occupational stress and emotional exhaustion, indicating health professionals with higher EI are able to accommodate higher levels of occupation stress without developing burnout.	57.14%
**Bikmoradi (2018) Nurse managers' emotional intelligence in educational hospitals: A cross-sectional study from the west of Iran**	Nurse Managers (n=370)<br>Males (n=79)<br>Females (n=291)	Emotional Intelligence	Bradberry & Greaves Emotional Intelligence Questionnaire	<u>Country:</u><br>Iran<br><u>Method of Collection:</u><br>Nurse managers from 5 educational hospitals.<br><u>Analysis:</u><br>Descriptive and inferential statistics, Pearson's correlation coefficient, t-test and variance analysis.	Nurse managers total EI mean (Sd) 80.46 (6.7) considered good and subscale:<br>- self-awareness 79.71 (12.55) - moderate<br>- self-management 80.87 (15.14) - good<br>- social awareness 80.67 (12) - good<br>- social skills 80.59 (14.3) - good	100.00%

**Bittinger (2020) Relationship Between Emotional Intelligence and Occupational Stress Levels Among Certified Registered Nurse Anaesthetists**	Anaesthetic Nurses (n=295)<br>Males (n=87)<br>Females (n=186)<br>Unknown (n=22)	Emotional Intelligence	Schutte Self-Report Emotional Intelligence Test (SSEIT)	Country:<br>USA<br>Method of Collection:<br>Anaesthetic nurses with active email addresses and currently employed recruited through Association of Nurse Anaesthetist.<br>Analysis:<br>Descriptive statistics,	Nurses EI mean (SD) of 125.79 (11.55) and by subscale:<br>- perception of emotion 37.87 (4.84)<br>- managing own emotions 35.23 (3.78)<br>- managing other's emotions 30.34 (3.46)<br>- Utilisation of emotion 22.46 (2.70)<br><br>Respondents with higher levels of EI cope better with workplace stressors.	85.71%
**Boey (1999) Distressed and stress resistant nurses.**	Nurses (n=1043)<br>Males (n=unknown)<br>Females (n=unknown)<br><br>Assistant nurses (n=371)<br>Staff nurses (n=532)<br>Nursing officers (n=121)<br>Missing data (n=19)	Behaviour	Type A Behaviour Scale (TABS)	Country:<br>Singapore<br>Method of Collection:<br>3 main public hospital nurses invited to participate, surveys distributed through nursing officers and return to a drop box.<br>Analysis:	Mean (SD) type A behaviour of nurses was 44.18 (6.09), responses ranging from 25-60.<br><br>No difference in behaviour between distressed and stress-resistant nurses.	85.71%

**Bogacheva (2019) Relationships Between Medical Doctors' Personality Traits and Their Professional Risk Perception**	Doctors (n=64)<br>Males (n=27)<br>Females (n=37)	Personality	Ten-Item Personality Inventory (TIPI)	<u>Country:</u><br>Russia<br><u>Method of Collection:</u><br>Completed questionnaires on personality traits in pen-and-paper<br><u>Analysis:</u><br>descriptive statistics, Mann-Whitney U Test, Kruskal-Wallis H test, post hoc Dunn test	Doctors personality mean (SD) by trait:<br>- risk-readiness -0.48 (3.16)<br>- rationality 4.94 (3.06)<br>- vigilance 16.31 (2.05)<br>- agreeableness 8.74 (1.82)<br>- intolerance for ambiguity 28.90 (7.02)	85.71%

**Bry (1980) Personality characteristics associated with success in learning and practicing nurse-midwifery.**	Midwifery Nursing Students (registered nursed)(n=36)<br>Males (n=0)<br>Females (n=36)	Personality	California Psychological Inventory	Country:<br>USA<br>Method of Collection:<br>Registered nursed currently enrolled in either of two midwifery programs in northeast in 1975/76. Survey was administered on registration and returned prior to completion of first year.<br>Analysis:<br>stepwise discriminate analysis.	Midwifery nursing student Mean (SD) by subscale:<br>- Dominance 31.52 (4.58)<br>- capacity for status 22.36 (2.72)<br>- sociability 27.58 (3.97)<br>- social pressure 38.47 (5.31)<br>- self-acceptance 23.14 (3.34)<br>- sense of well-being 38.36 (3.22)<br>- responsibility 31.92 (4.28)<br>- socialisation 37.75 (4.86)<br>- Self-control 33.06 (6.03)<br>- Tolerance 25.53 (3.19)<br>- good impression 21.06 (5.32)<br>- communality 25.69 (2.21)<br>- achievement via conformance 31.00 (3.66)<br>- achievement via independence 22.67 (3.24)<br>- intellectual efficiency 44.14 (3.39)<br>- psychological-mindedness 16.56 (2.24)<br>- flexibility 11.14 (3.57)<br>- femininity 23.44 (2.80)<br><br>The Socialization, Dominance, Tolerance, and Self-Control scales, taken together, accounted for individuals who	71.43%

		completed the course without difficult and was practicing for all except two of the subjects.

**Bucknall (2015) Mirror on the ward, who's the most narcissistic of them all? Pathologic personality traits in health care**	Health Professionals (n=248)<br>Males (n=126)<br>Females (n=122)<br><br>Medical (n=199)<br>Nursing (n=49)	Personality	Narcissistic Personality Inventory (NPI)<br>MACH-IV test of Machiavellianism<br>Levenson Self-Report Psychopathy Scale (LSRP)	<u>Country:</u><br>United Kingdom<br><u>Method of Collection:</u><br>Questionnaire packs were distributed to a UK secondary care teaching hospital and university affiliated hospital that provide tertiary care. Any healthcare professional involved with direct patient care was eligible. Control general population sample recruited through snowball sampling via social media with links to survey completion.<br><u>Analysis:</u><br>Parametric data analysis. ANOVA with Bonferroni pos hoc for multiple comparisons between cohorts and subgroups.	Healthcare professionals total mean (95% CI) for the dark triad personality factors:<br>- Narcissism 12.0 (11.3 - 12.7)<br>- Machiavellianism 53.0 (51.9 - 54.1)<br>- Psychopathy 44.7 (43.8 - 45.6)<br>- Primary 26.4 (25.7 - 27.1)<br>- Secondary 18.4 (18.0 - 18.8)<br><br>Health care professionals scored significantly lower on narcissism, Machiavellianism and psychopathy than the general population. Health care professionals expressed low levels of dark triad personality traits.<br><br>See categorised quantitative data spreadsheet for detailed breakdown of dark triad personality factors by health care professional subgroup mean (95% CI) and speciality mean.	85.71%

**Buddeberg-Fischer (2008) The new generation of family physicians--career motivation, life goals and work-life balance.**	Medical Physicians (n=504)<br>Males (n=232)<br>Females (n=272)<br><br>Family Physician (n=84)<br>Other Specialists (n=420)	Personality	Personal Attribute Questionnaire (PAQ)<br>Sense of Coherence Scale (SOC-13)	Country:<br>Switzerland<br>Method of Collection:<br>longitudinal prospective career development data collected at 4 time frames from 2001 - 2007 of three medical schools from graduation through to 5-6 years of residency to determine family physician or other specialisation.<br>Analysis:<br>Comparison between groups and relationship between tools using multivariate analysis.	Physicians personality factors mean (SD) of sense of coherence:<br>- Family physicians 4.92 (0.83)<br>- Other specialists 5.06 (0.86)<br>Physicians personality factors mean (SD) of PAQ - Instrumentality:<br>- Family physicians 4.06 (0.65)<br>- Other specialists 4.17 (0.67)<br>Physicians personality factors mean (SD) of PAQ - Expressiveness:<br>- Family physicians 4.95 (0.53)<br>- Other specialists 4.85 (0.58)<br><br>Nil difference in personality factors between groups.	100.00%

**Buining (2015) Exploring physiotherapists' personality traits that may influence treatment outcome in patients with chronic diseases: a cohort study.**	Physiotherapists (n=39)<br>Males (n=29)<br>Females (n=10)	Personality	Big Five Inventory (Dutch version)	Country:<br>Netherlands<br>Method of Collection:<br>Cohort study of data from the NIVEL Primary Care Database (NPCD) in 2011 - 2012, and questionnaire via annual NPCD physical therapy questionnaire in 2012, of clinicians who treated eligible patients and consented to study.<br>Analysis:<br>Descriptive statistics, two-level linear regressions, independent t-tests or Mann-Whitney U tests exploring personality impact on treatment outcomes of chronic disease patients.	Physiotherapist personality mean (SD) (Range) by subcategories:<br>- Neuroticism 2.38 (0.09) (1.25-3.88)<br>- extraversion 3.49 (0.07) (2.63-4.63)<br>- Agreeableness 3.75 (0.06) (3.00-4.78)<br>- Conscientiousness 3.69 (0.08) (2.89-4.89)<br>- Openness 3.42 (0.09) (2.70-4.80)<br><br>Higher BFI scores on openness, extraversion, conscientiousness and agreeableness and lower on neuroticism.<br>Neuroticism only significant variable to have an influence on treatment outcome, with therapists that are calmer, more relaxed and hardy produce better patient outcomes.	100.00%

**Campbell (2013) What does personality tell us about working in the bush? Temperament and character traits of Australian remote allied health professionals.**	Allied Health Professionals (n=561)<br>Males (n=59)<br>Females (n=502)<br><br>Not Remote group (n=295)<br>Remote group (n=266)	Personality	Temperament and Character Inventory (TCI-R 140)	Country:<br>Australia<br>Method of Collection:<br>Snowball sampling through Australian Rural and Remote Allied Health (SARRAH), all AH professionals eligible and survey open for 3 months in 2010/2011.<br>Analysis:<br>Descriptive statistics, and comparisons between groups using t-tests, ANOVA and two-way ANOVA.	Total Allied Health professionals TCI traits mean (SD) and rank to pop norm by subscale:<br>- Novelty Seeking 55.5 (8.39) - High<br>- Harm Avoidance 54.19 (11.95) - Average<br>- Reward Dependence 71.84 (9.58) - Very high<br>- Persistence 72.36 (9.50) - Very high<br>- Self-directedness 77.38 (9.91) - Very High<br>- Cooperativeness 83.35 (7.31) - Very High<br>- Self-transcendence 44.96 (11.08) - Low<br><br>Remote AHPs were higher in novelty seeking and self-transcendence. Older remote AHPS were lower in reward dependence; younger remote AHPs were lower in self directedness and higher in harm avoidance .<br>Remote women were lower in harm avoidance. Women were more reward dependent and cooperative than men.	71.43%

**Campbell (2014) Investigating personality and conceptualising allied health as person or technique oriented**	Allied Health Professionals (n=562)<br>Males (n=59)<br>Females (n=503)<br><br>Person-Oriented Group (n=492):<br>Aboriginal health worker (n=3)<br>Dietitian (n=85)<br>Exercise physiologist (n=4)<br>Occupational therapist (n=94)<br>Physiotherapist (n=105)<br>Psychologist (n=48)<br>Social worker (n=53)<br>Speech pathologist (n=98)<br>Other (n=2)<br><br>Technique-Oriented Group (n=70)<br>Audiologist (n=4)<br>Health promotion	Personality	Temperament and Character Inventory (TCI-R 140)	Country:<br>Australia<br>Method of Collection:<br>Snowball sampling through Australian Rural and Remote Allied Health (SARRAH), all AH professionals eligible.<br>Analysis:<br>Descriptive statistics, and comparisons between groups using t-tests and ANOVA.	Total Allied Health professionals TCI traits mean (SD) by subscale:<br>- Novelty Seeking 55.5 (8.4)<br>- Harm Avoidance 54.2 (12.0)<br>- Reward Dependence 71.9 (9.6)<br>- Persistence 72.4 (9.5)<br>- Self-directedness 77.4 (10.0)<br>- Cooperativeness 83.4 (7.3)<br>- Self-transcendence 45.0 (11.0)<br><br>Person-Oriented Allied Health professionals TCI traits mean (SD) by subscale:<br>- Novelty Seeking 55.6 (8.5)<br>- Harm Avoidance 53.9 (12.0)<br>- Reward Dependence 72.3 (9.4)<br>- Persistence 72.2 (9.4)<br>- Self-directedness 77.8 (9.7)<br>- Cooperativeness 84.0 (7.0)<br>- Self-transcendence 45.5 (11.0)<br><br>Technique-oriented Allied Health professionals TCI traits mean (SD) by subscale:<br>- Novelty Seeking 55.0 (7.7)<br>- Harm Avoidance 55.7 11.8)	57.14%

	(n=8)<br>Imaging (n=14)<br>Medical laboratory science (n=1)<br>Optometry (n=5)<br>Oral health (n=3)<br>Orthotics (n=3)<br>Orthoptics (n=1)<br>Pharmacists (n=8)<br>Podiatrist (n=23)		- Reward Dependence 68.7 (10.6)<br>- Persistence 73.6 (9.7)<br>- Self-directedness 74.8 (11.0)<br>- Cooperativeness 79.5 (7.8)<br>- Self-transcendence 41.1 (10.9)<br><br>Professionals in the PO group showed significantly higher levels of traits that emphasise person orientation attributes, such as being sociable, empathic and cooperative, compared with AH professionals in professions with an emphasis on TO.

**Chaffey (2012) Relationship Between Intuition and Emotional Intelligence in Occupational Therapists in Mental Health Practice**	Mental Health Occupational Therapists (n=134)<br>Males (n=6)<br>Females (n=124)<br>Missing (n=4)	Emotional Intelligence	Swinburne University Emotional Intelligence Test (SUEIT)	<u>Country:</u><br>Australia<br><u>Method of Collection:</u><br>Surveys distributed to all OT;'s working in mental health via national association database and returned the surveys within 3 weeks.<br><u>Analysis:</u><br>Descriptive statistics, Mann-Whitney U tests compared variable scores, two-tailed Spearman ranks for associations.	Occupational Therapist mean (SD) EI total score of 229.51 (21.11) and subscale scores:<br>- Emotional recognition and expression: 39.65 (5.26) (higher than norm)<br>- Understanding Emotions, external: 78.46 (8.24) (higher than norm)<br>- emotions direct cognition: 38.03 (6.19) (lower than norm)<br>- emotional management 41.41 (5.31) (higher than norm)<br>- emotional control 31.67 (4.40) (higher than norm)<br><br>Occupational Therapist mean (SD) EI total scores by gender:<br>- Male: 213.00 (18.01)<br>- Female: 229.65 (30.93)<br>Positive difference in EI between genders (? as a result of small sample of males)<br><br>Occupational Therapist mean (SD) EI scores by level of experience:<br>- Novice (< 5 years): 219.85 (16.01)<br>- Intermediate (5-10 years): 231.51 (22.73)	71.43%

	- Experienced (>10 years): 231.98 (21.51)<br>Only significant difference between novice and experienced.

**Chang (2010) The influence of personality traits on nurses' job satisfaction in Taiwan**	Nurses (n=314)<br>Males (n=3)<br>Females (n=311)	Personality	Proactive Coping Scale	Country:<br>Taiwan<br>Method of Collection:<br><br>Analysis:<br>descriptive statistics, Pearson product-moment correlation and multiple regression.	Nurse proactive personality mean (SD) of 56.76 (7.74)<br><br>Personality variables should be considered during the process of evaluating job satisfaction.	100.00%
**Chang (2016) Openness to experience, work experience and patient safety.**	Nurses (n=421)<br>Males (n=4)<br>Females (n=410)<br>Missing (n=7)	Personality	Big Five Inventory (BFI) - Openness only.	Country:<br>Taiwan<br>Method of Collection:<br>Nurses working at major medical centre in north Taiwan. Paper surveys were distributed and collected via research assistants.<br>Analysis:<br>Hierarchical regression analysis	Nurses openness scores by subscale:<br>- Creative 5.39 (1.41)<br>- Intellectual 5.71 (1.28)<br>- Artistic 5.61 (1.26)<br>- Intelligent 2.67 (1.26)<br>- Philosophical 5.07 (1.36)<br>- Deep 5.36 (1.36)<br><br>Nurse openness is positively related to the patient safety construct; work experience reduces the relation between openness and patient safety.	85.71%

**Chang (2019) Examining the association of career stage and medical specialty with personality preferences - a cross-sectional survey of junior doctors and attending physicians from various specialties**	Doctors (n=98)<br>Males (n=70)<br>Females (n=28)<br><br>Attendings (n=59)<br>Juniors (n=39)	Personality	Myers-Briggs Type Indicator (MBTI)	<u>Country:</u><br>Taiwan<br><u>Method of Collection:</u><br>Convivence sample between Aug 2015 - July 2016 at Chang Gun Memorial Hospital.<br><u>Analysis:</u><br>descriptive statistics	Doctors personality mean (SD) by position and trait.<br><br><u>Junior</u> doctors:<br>- extraversion 9.90 (6.91)<br>- introversion 11.0 (6.94)<br>- sensing 14.5 (4.96)<br>- intuition 11.5 (4.96)<br>- thinking 12.2 (5.41)<br>- feeling 11.8 (5.41)<br>- judgement 12.3 (6.94)<br>- perception 9.67 (6.94)<br><br><u>Attending</u> doctors:<br>- extraversion 9.31 (5.95)<br>- introversion 11.7 (5.95)<br>- sensing 16.7 (4.99)<br>- intuition 9.24 (4.95)<br>- thinking 14.8 (5.47)<br>- feeling 9.15 (5.47)<br>- judgement 15.5 (6.27)<br>- perception 6.46 (6.46)	85.71%

**Chao (2016) Nurse occupational burnout and patient-rated quality of care: The boundary conditions of emotional intelligence and demographic profiles**	Nurses (n=98)<br>Males (n=0)<br>Females (n=98)	Emotional Intelligence	Goleman's Emotional Intelligence Scale	<u>Country:</u><br>Taiwan<br><u>Method of Collection:</u><br>Surveys distributed by trained research assistants and returned by nurses in envelope.<br><u>Analysis:</u><br>Descriptive statistics, Pearson's correlations, Cronbach's alpha	Nurses EI mean (SD) of 3.64 (0.48)	71.43%
**Chen (2016) Influence of work values and personality traits on intent to stay among nurses at various types of hospital in Taiwan.**	Nurses (n=1246)<br>Males (n=14)<br>Females (n=1232)	Personality	Five-Factor Inventory	<u>Country:</u><br>Taiwan<br><u>Method of Collection:</u><br>Nurses recruited from 3 different types (private, government & religious) hospitals in north-eastern Taiwan, working full time minimum 3 months.<br><u>Analysis:</u><br>Multiple regression analysis establish predictive factors.	Nursing EI mean (SD) by subscale:<br>- Emotional stability 18.6 (4.53)<br>- Extraversion 18.95 (2.44)<br>- Openness to experience 18.71 (2.35)<br>- Agreeableness 20.32 (2.92)<br>- Conscientiousness 20.25 (2.40)<br><br>Age, the role of work values, personality traits and type of hospital were crucial in enhancing the level of intent to stay at work.	85.71%

**Chen (2018) The influence of personality traits and socio-demographic characteristics on paediatric nurses' compassion satisfaction and fatigue**	Paediatric nurses (n=173)<br>Males (n=0)<br>Females (n=173)	Personality	Revised Neo-Personality Inventory (NEO-PI-R)	Country:<br>Taiwan<br>Method of Collection:<br>Nurses within minimum 3 months experience in teaching children's hospital distributed surveys between April - July 2014.<br>Analysis:<br>Descriptive statistics, multilinear regressions to determine relationships between variables.	Nurses personality traits mean (SD), in sequence from top to bottom by subscale:<br>- Agreeableness 3.57 (0.43)<br>- Conscientiousness 3.51 (0.43)<br>- Emotional stability 3.37 (0.67)<br>- Openness 3.08 (0.48)<br>- Extraversion 3.08 (0.38)<br><br>Agreeableness, extraversion, conscientiousness were predictive of compassion satisfaction. Emotional stability is a risk factors of compassion fatigue.	71.43%
**Cheng (2012) Longitudinal effects of job insecurity on employee outcomes: The moderating role of emotional intelligence and the leader-member exchange: APJM**	Nurses (n=323)<br>Males (n=0)<br>Females (n=323)	Emotional Intelligence	Wong and Law Emotional Intelligence (WLEIS)	Country:<br>China<br>Method of Collection:<br>Female nurses from 3 hospitals in central China, were hand delivered surveys and collected by research assistant.<br>Analysis:<br>Descriptive statistics, moderated hierarchical regression and regression analysis to determine relationship between variables	Nurses total EI mean (SD) of 3.63 (0.60)<br><br>Findings reveal that the ability of employees to deal with their emotions and their relationships with their supervisors is an important resource that serves to protect employee outcomes when job security is uncertain.	85.71%

**Cherry (2018) Do doctors' attachment styles and emotional intelligence influence patients' emotional expressions in primary care consultations? An exploratory study using multilevel analysis.**	Doctors (n=26)<br>Males (n=5)<br>Females (n=21)	Emotional Intelligence	Mayer-Salovey-Caruso Emotional Intelligence Test (MSCEIT)	<u>Country:</u><br>United Kingdom<br><u>Method of Collection:</u><br>Junior doctors and their patients from 20 GP practices in Northwest England.<br><u>Analysis:</u><br>Pearson's product-moment correlations, independent sample t-tests, Chi-squared tests and one-way ANOVAs for preliminary data exploration. Multi-level models investigated predictive relationships.	Total Doctor EI score mean (SD) of 101.89 (15.44) with a range of 79.77 - 129.20.<br><br>EI found to be significantly associated with patients' emotional expressions, patient and doctor level explanatory variables.	85.71%

**Choudary (2011) Emotional Intelligence as A Factor in Work Related Outcomes - A Study Among the Paramedical Staff at SRM Medical College, Kattankulathur, Chennai**	Nurses (n=67)<br>Males (n=unknown)<br>Females (n=unknown)	Emotional Intelligence	Self-Emotional Intelligence Scale	Country:<br>India<br>Method of Collection:<br>Random sample of nurses at group of hospitals within Kanchipuram.<br>Analysis:<br>Descriptive statistics,	Mean (SD) for each of the EI for self-emotional appraisal:<br>- Understand own emotions 3.6418 (1.12414) / moderate<br>- Understand the reason for emotions 3.0597 (1.15313) / not able to comment on their reason of emotions.<br>- Speak about personal problems to others 3.2239 (1.13912) / unaware about the time to speak to others.<br><br>Mean (SD) for each of the EI for others emotional appraisal:<br>- Know patients emotions from their behaviour 3.6866 (0.97248) / moderate understanding<br>- Frustration when patient's attendant is restless 2.9851 (1.27318) / uncertain<br>- Sensitivity to feelings and emotions of others 3.4179 (1.39424) / moderated sensitivity<br><br>Mean (SD) for each of the EI for use of emotions:<br>- personal goal setting and try best to achieve them 3.0448	71.43%

	(1.30770) / uncertain<br>- gives best to serve patient 3.1940 (1.15783) / unaware<br>- good mood to overcome obstacles 3.2388 (1.06015) / unaware of how to use good emotions and not able to control emotions<br><br>Mean (SD) for each of the EI for <u>regulation of emotions</u>:<br>- control temper and handle difficulties rationally (1) 2.4478 (1.10486) / moderately disagree<br>- control temper and handle difficulties rationally (2) 2.9552 (1.17335) / moderately disagree<br>- think clearly and stay focused under pressure 2.4925 (1.29537) / moderately disagree<br><br>Mean (SD) for each of the EI for <u>working environment</u>:<br>- think clearly and stay focused under pressure (1) 1.6716 (1.10629)<br>- think clearly and stay focused under pressure (2) 2.5672

	(1.36208)<br>- help others who have heavy workloads 3.5522 (1.24661) / moderate agree

**Clack (2004) Personality differences between doctors and their patients: implications for the teaching of communication skills.**	Doctors (n=313)<br>Males (n=151)<br>Females (n=162)	Personality	Myers–Briggs Type Indicator (MBTI)	Country:<br>United Kingdom<br>Method of Collection:<br>Survey distributed to medical graduates from the King's College School of Medicine and Dentistry between 1985 ⁄ 86 and 1989 ⁄ 90.<br>Analysis:<br>Chi square and Fisher Exact Test uses to compare doctors vs population norms personality.	Medical graduates percentage personality preferences for extraversion:<br>- All medical graduates 47.0%<br>- Males 39.1%<br>- Females 54.3%<br>Medical graduates percentage personality preferences for Introversion:<br>- All medical graduates 53.0%<br>- Male 60.9%<br>- Female 45.7%<br>Medical graduates percentage personality preferences for Sensing:<br>- All medical graduates 49.5%<br>- Male 45.0%<br>- Female 53.7%<br>Medical graduates percentage personality preferences for Intuition:<br>- All medical graduates 50.5%<br>- Male 55.0%<br>- Female 46.3%<br>Medical graduates percentage personality preferences for Thinking:<br>- All medical graduates 63.6%<br>- Male 80.8%<br>- Female 47.5%<br>Medical graduates percentage	85.71%

personality preferences for
Feeling:
- All medical graduates 36.4%
- Male 19.2%
- Female 52.5%

Medical graduates percentage personality preferences for Judging:
- All medical graduates 68.1%
- Male 64.2%
- Female 71.6%

Medical graduates percentage personality preferences for Perceiving:
- All medical graduates 31.9%
- Male 35.8%
- Female 28.4%

Medical graduates percentage personality preferences for Sensing with Thinking:
- All medical graduates 32.3%
- Male 39.7%
- Female 25.3%

Medical graduates percentage personality preferences for Sensing with Feeling:
- All medical graduates 17.2%
- Male 5.3%
- Female 28.4%

Medical graduates percentage personality preferences for

	<u>Intuition with Feeling</u>:<br>- All medical graduates 19.2%<br>- Male 13.9%<br>- Female 24.1%<br>Medical graduates percentage <u>personality preferences</u> for <u>Intuition with Thinking</u>:<br>- All medical graduates 31.3%<br>- Male 41.1%<br>- Female 22.2%

**Codier (2008) Measuring the emotional intelligence of clinical staff nurses: an approach for improving the clinical care environment.**	Nurses (n=37)<br>Males (n=3)<br>Females (n=24)	Emotional Intelligence	MSCEIT	<u>Country:</u><br>USA<br><u>Method of Collection:</u><br>Three hospitals in Honolulu, Hawaii nurses were sent survey to complete online.<br><u>Analysis:</u><br>Descriptive statistics, correlational analysis, and analysis of variance.	EI Frequency (%) by subscale <u>perceiving emotions</u>:<br>- below average 41%<br>- average 44%<br>- above average 15%<br>EI Frequency % by subscale <u>Using emotions</u>:<br>- below average 26%<br>- average 63%<br>- above average 12%<br>EI Frequency % by subscale <u>understanding emotions</u>:<br>- below average 11%<br>- average 78%<br>- above average 11%<br>EI Frequency % by subscale <u>managing emotions</u>:<br>- below average 22%<br>- average 44%<br>- above average 33%<br><br>High percentage of nurses below average EI.	57.14%

**Codier (2013) Developing Emotional Intelligence Ability in Oncology Nurses: A Clinical Rounds Approach**	Oncology Nurses (n=10)<br>Males (n=unknown)<br>Females (n=unknown)	Emotional intelligence	Mayer-Salovey-Caruso Emotional Intelligence Test (MSCEIT)	Country:<br>USA<br>Method of Collection:<br>RN's in a 24 bed oncology unit of a tertiary care hospital in urban Honolulu, HI. Electronically collected survey responses.<br>Analysis:	Total EI mean (range) scores of 99 (79-107), with subscale scores of:<br>- Experiential EI 100 (58-121)<br>- Strategic EI 100 (79-106)<br>- Identifying emotions 101 (85-129)<br>- using emotions to reason 100 (86-116)<br>- Understanding emotions 100 (70-113)<br>- Managing emotions 99 (91-106)<br><br>Ability to identify emotions in self and others was demonstrated less frequently than expected in this population.	57.14%

**Coladonato (2017) Nurse leader emotional intelligence: How does it affect clinical nurse job satisfaction?**	Nurse leaders (n=20)<br>Males (n=0)<br>Females (n=20)	Emotional Intelligence	Bar-On's Emotional Quotient Inventory (EQ-I 2.0)	Country:<br>USA<br>Method of Collection:<br>All nurse leadership positions are NFP community hospital in south-eastern Pennsylvania invited to participate via email of EI survey.<br>Analysis:<br>Descriptive stats and multiple linear regression relationship analysis.	Total EI mean (SD) (range) scores of 104.76 (10.549) (81-129)<br><br>EI composite scores mean (SD):<br>- self-perception 101.41 (11.335)<br>- self-expression 102.97 (12.511)<br>- interpersonal 106.6 (9.7450)<br>- decision making 104.41 (11.980)<br>- stress management 105.15 (10.944)<br>All scores fell in mid-range (90-110).	71.43%
**Coombs (1993) Surgeons' personalities: the influence of medical school**	Doctors (n=39)<br>Males (n=unknown)<br>Females (n=unknown)<br><br>Surgeons (n=12)<br>non-surgeons (n=27)	Personality	California Psychological Inventory	Country:<br>USA<br>Method of Collection:<br>Surveys completed as first year medical student and then followed up at graduation<br>Analysis:<br>Descriptive statistics, two-tailed tests of significance	Developing surgeons more flexible, adaptive to change than non-surgical classmates.	57.14%

**Cordina (2012) Career paths and personality in pharmacy**	Pharmacists (n=275)<br>Males (n=90)<br>Females (n=185)	Personality	Gordon Personal Profile Index (GPPI)	Country:<br>Malta<br>Method of Collection:<br>Surveys were mailed to pharmacists registered and residing in Malta and returned via mail.<br>Analysis:<br>Descriptive statistics, MANOVA and multiple correspondence analysis.	Pharmacists mean (SD) by personality traits:<br>- Ascendency: 20.99 (5.9)<br>- Responsibility; 27.43 (4.5)<br>- Emotional stability: 22.42 (6.3)<br>- Sociability: 21.09 (6.3)<br>- Self-esteem: 91.94 (15.2)<br>- Cautiousness: 26.46 (5.4)<br>- Original Thinking: 24.87 (5.4)<br>- Personal Relations: 22.55 (5.7)<br>- Vigour: 26.90 (5.8)<br>Break down by gender in categorised spreadsheet.<br><br>Most of the types of pharmacists lie somewhat close to the average categories of the GPPI attributes.<br>Importation and wholesale high on self-esteem, ascendency, original thinking, vigour, sociability.<br>Industry and Hospitals score low on the GPPI traits in general<br>Community scored high in personal relations and are a little more	85.71%

	likely to score higher in cautiousness and responsibility.

**Coskun (2018) Emotional intelligence and leadership traits among family physicians**	Doctors (n=3947)<br>Males (n=2975)<br>Females (n=972)	Emotional Intelligence	Trait Emotional Intelligence Questionnaire (TEIQue)	Country:<br>Turkey<br>Method of Collection:<br>Family doctors in Turkey between Sept 2013 - Dec 2014, questionnaire emailed to consenting participants.<br>Analysis:<br>Descriptive statistics,	Doctors EI mean (SD) by gender and subscale<br><br>Female global EI 5.32 (0.023)<br>- well-being 5.66 (0.028)<br>- self-control 5.00 (0.025)<br>- emotionality 5.6 (0.025)<br>- sociability 5.07 (0.027)<br><br>Male global EI 5.24 (0.014)<br>- well-being 5.47 (0.017)<br>- self-control 5.09 (0.015)<br>- emotionality 5.35 (0.015)<br>- sociability 5.12 (0.016)<br><br>Doctors TEI differs based on sex, age, health-care experience, and the geographical region where they work. Women had higher mean values than men for well-being, emotionality, and global TEI.	85.71%

**Cross (1984) Personality typing and anxiety for two groups of registered nurses**	Nurses (n=96)<br>Males (n=12)<br>Females (n=84)	Personality	Myers–Briggs Type Indicator (MBTI)	<u>Country:</u><br>Australia<br><u>Method of Collection:</u><br>Nurses at two large teaching hospitals in Brisbane, were approached during education session/ team meetings to participate.<br><u>Analysis:</u><br>Not specified.	Nurses personality type percentages:<br>- extroversion 28.1%<br>- introversion 71.9%<br>- Sensing 63.5%<br>- Intuition 36.5%<br>- Thinking 52.1%<br>- Feeling 47.9%<br>- Judging 54.2%<br>- Perception 45.8%<br><br>Nurse in this study cluster in the IS quadrant, where introversion and sensing are preferred most often.<br>Nurses more introverted than extroverted.	85.71%
**Crowne (2017) Leading nurses: emotional intelligence and leadership development effectiveness**	Nursing Home Nurse Leaders (n=20)<br>Males (n=1)<br>Females (n=19)	Emotional Intelligence	Bar-On's Emotional Quotient Inventory (EQ-I)	<u>Country:</u><br>USA<br><u>Method of Collection:</u><br>RN leaders in 30 nursing homes in five county areas Philadelphia, South Jersey and Delaware<br><u>Analysis:</u><br>Correlation analysis and t-tests	Total EI Mean (SD) scores of 97.25 (13.384).<br><br>Positive significant relationship between EI and transformational leadership.	85.71%

**Cybukska (2022) Analysis of Factors Related to Mental Health, Suppression of Emotions, and Personality Influencing Coping with Stress among Nurses**	Nurses (n=811)	Personality	NEO-FIVE Factor Inventory (NEO-FFI)	Country:<br>Poland<br>Method of Collection:<br>Nurses employed in West Pomeranian Voivodeship with at least 1 years work experience were randomly selected through dissemination of paper version of survey.<br>Analysis:<br>r-persons correlation coefficient, descriptive statistics, statistical inference.	Nurse personality mean (SD) by trait:<br>- Extraversion 5.8 (1.8)<br>- Openness 5.5 (1.8)<br>- Conscientiousness 6.4 (2.1)<br>- agreeableness 5.8 (2.2)<br>- Neuroticism 4.7 (2.2)	85.71%
**De la Fuente-Solana (2019) An Explanatory Model of Potential Changes in Burnout Diagnosis According to Personality Factors in Oncology Nurses.**	Oncology Nurses (n=96)<br>Males (n=unknown)<br>Females (n=unknown)	Personality	NEO Five-Factor Inventory (NEO-FFI)<br>Maslach Burnout Inventory (MBI)	Country:<br>Spain<br>Method of Collection:<br>Oncology nurses from Andalusian Health Service, contacted via Spanish nursing union with questionnaires coordinated collection.<br>Analysis:<br>Three categorical-response logit ordinal models for each dimension of burnout.	Oncology nurse personality mean (SD) by subscale:<br>- neuroticism 25.85 (6.79)<br>- friendliness 45.81 (5.90)<br>- responsibility 47.76 (6.08)<br>- extraversion 43.39 (6.67)<br>- openness 39.3 (6.68)<br><br>Nurses are more likely to develop high levels of burnout if they present high levels of neuroticism and low levels of friendliness and responsibility.	71.43%

**De la Fuente-Solana (2021a) Burnout Syndrome in Paediatric Nurses: A Multi-Centre Study**	Paediatric Nurses (n=95)<br>Males (n=22)<br>Females (n=73)	Personality	NEO Five-Factor Inventory (NEO FFI)	<u>Country:</u><br>Spain<br><u>Method of Collection:</u><br>Information provided to eligible nurses, with voluntary participation. Data collected between Oct 2019 and Feb 2020<br><u>Analysis:</u><br>Descriptive statistics, Pearson's correlation coefficient, multiple linear regressions.	Nurses personality mean (SD) by trait:<br>- Neuroticism 27.98 (6.849)<br>- Extraversion 42.51 (6.240)<br>- Openness 38.07 (6.539)<br>- Agreeableness 46.53 (5.045)<br>- Conscientiousness 47.36 (6.007)<br><br>Personality was found to play an important role in development of burnout syndrome.	85.71%
**De la Fuente-Solana (2021b) Predictors of burnout of health professionals in the departments of maternity and gynaecology, and its association with personality factors: A multicentre study**	Nurse midwives (n=150)<br>Males (n=32)<br>Females (n=118)	Personality	NEO Five-Factor Inventory (NEO FFI)	<u>Country:</u><br>Spain<br><u>Method of Collection:</u><br>Nurses from 18 hospitals, were approached during clinical meetings, survey distributed and returned in sealed envelopes.<br><u>Analysis:</u><br>Descriptive statistics, t-tests, Pearson's correlation coefficients	Nurses personality mean (SD) by trait:<br>- neuroticism 27.03 (6.705)<br>- extraversion 43.79 (6.629)<br>- conscientiousness 48.47 (6.006)<br>- agreeableness 47.72 (5.747)<br>- openness 10.43 (6.045)	100.00%

**DePew (1999) The relationship of burnout, stress, and hardiness in nurses in a military medical centre: a replicated descriptive study**	Nurses (n=49)<br>Males (n=18)<br>Females (n=29)<br>Missing (n=2)<br><br>Unable to determine n for each of the speciality areas.	Personality	Hardiness Test	Country:<br>USA<br>Method of Collection:<br>Nurse in military hospital received surveys in mail slots located at work unit, with envelope to return completed within 2 weeks (reminders at 7,0 and 14 days).<br>Analysis:<br>Descriptive statistics, Pearson's correlations	Nurses total hardiness mean (SD) of 72 (10) and by speciality area:<br>- Burns ICU: 64 (12)<br>- Medical ICU: 69 (14)<br>- Surgical ICU: 72 (7)<br>- Trauma ICU: 75 (11)<br>- Bone Marrow Transplant: 72 (8)<br>- Dialysis: 75 (6)<br>- ER: 74 (8)<br><br>Burns nurses greatest burnout and lowest hardiness.	71.43%
**DiRenzo (1981) Personality and values in family medicine residents**	Residents (n=22)<br>Males (n=17)<br>Females (n=5)	Personality	Dogmatism Scales	Country:<br>USA<br>Method of Collection:<br>residents in family practice program in large medical centre in eastern metropolitan area<br>Analysis:<br>Descriptive statistics	Resident dogmatism mean (SD) of 12.50 (16.98) (possible score range of 60).	14.29%

**Donato (2017) Personality Traits and Performance Contracts: Evidence from a Field Experiment among Maternity Care Providers in India**	Obstetric providers (n=97)<br>Males (n=unknown)<br>Females (n=unknown)	Personality	Big Five Inventory	<u>Country:</u><br>India<br><u>Method of Collection:</u><br>Solo practicing obstetric providers within Karnataka India invited to study.<br><u>Analysis:</u><br>?	means scores by subscale:<br>- Conscientiousness 4.64<br>- Neuroticism 1.81<br><br>Most providers exhibit high levels of conscientiousness and low levels of neuroticism. Conscientiousness providers perform better. Higher neuroticism do not improve their performance with incentives.	42.86%
**Drach-Zahavy (2019) The personality profile of the accountable nurse and missed nursing care.**	Nurses (n=290)<br>Males (28.7%)<br>Females (71.3%)	Personality	Big Five Inventory	<u>Country:</u><br>Israel<br><u>Method of Collection:</u><br>Snowball sampling where students distributed surveys to RN's across multiple hospitals and wards during 2017. surveys were completed at end of a shift.<br><u>Analysis:</u><br>Descriptive statistics and correlations and comparisons through chi-squares and degrees of freedom difference tests.	Personality mean (SD) by subscales:<br>- extraversion 3.46 (0.58)<br>- openness to experience 3.47 (0.54)<br>- agreeableness 3.81 (0.59)<br>- Conscientiousness 4.01 (0.55)<br>- neuroticism 2.46 (0.60)<br><br>Conscientiousness, Agreeableness, Openness and Neuroticism, but not Extraversion, were significantly related to personal accountability.	85.71%

**Drosdeck (2015) Surgeon and non-surgeon personalities at different career points**	Surgeons (n=68)<br>Males (n=44)<br>Females (n=24)	Personality	Big Five Inventory (BFI)	<u>Country:</u><br>USA<br><u>Method of Collection:</u><br>Survey completed online via SurveyMonkey.<br><u>Analysis:</u><br>descriptive statistics, nonpaired two-tailed t-tests	Surgeons personality mean (SD) by trait:<br>- openness 3.75 (0.48) - lower than pop norm<br>- conscientiousness 4.30 (0.41) - higher than pop norm<br>- extraversion 3.74 (0.68) - higher than pop norm<br>- agreeableness 3.76 (0.48) - lower than pop norm<br>- neuroticism 2.46 (0.75) - lower than pop norm<br><br>Inherent personality differences between surgical and nonsurgical specialties. Surgeons scored significantly higher on conscientiousness and extraversion but lower on agreeableness compared to non-surgeons. Surgery faculty scored lower in agreeableness compared to surgery staff. Non-surgeon faculty scored higher on extraversion compared non-surgeon staff.	85.71%

**Dugan (2014) A longitudinal study of emotional intelligence training for otolaryngology residents and faculty**	Doctors (n=106)<br>Males (n=<br>Females (n=	Emotional Intelligence	Bar-On's Emotional Quotient Inventory (EQ-I)	<u>Country:</u><br>USA<br><u>Method of Collection:</u><br>Single department survey completion pre- and post- EI training program.<br><u>Analysis:</u><br>descriptive statistics	Doctors global EI mean (SD) 104.29 (5.91).<br><br>EI training shown to increased EI.	85.71%

**Dumitru (2012) The relationship between stress and personality factors**	Psychiatric Nurses (n=34)<br>Males (n=32%)<br>Females (n=68%)	Personality	California Psychological Inventory	<u>Country:</u><br>Romania<br><u>Method of Collection:</u><br>Qualified nurses with min 1 years' experience<br><u>Analysis:</u>	Personality factors that were different mean (SD)/median between females and Males by subscales:<br>- Social presence<br>- Female 21.70 (4.466)<br>- Male 25.00 (4.000)<br>- Independence<br>- Female 17.17 (4.509)<br>- Male 18.82 (3.157)<br>- Good impression<br>- Female 18.39 (7.353)<br>- Male 17.55 (5.373)<br>- Well-being<br>- Female 28.48 (4.926)<br>- Male 31.64 (3.529)<br>- Tolerance<br>- Female 17.48 (3.918)<br>- Male 19.27 (3.495)<br>- Femineity<br>- Female 19.61 (3.858)<br>- Male 13.18 (2.442)<br>- Work orientation<br>- Female 26.57 (4.698)<br>- Male 28.27 (2.901)<br>- Empathy<br>- Female 18<br>- Male 18<br>- Intellectual efficiency<br>- Female 28<br>- Male 32	85.71%

Several personality factors, such as social presence, empathy, independence, good impression, intellectual efficiency, psychological intuition, work orientation, femininity render individuals more vulnerable to stress. There are significant differences between females and males in what concerns stress adaptation.

**Duschek (2020) Stress in paramedics: relationships with coping strategies and personality traits**	Paramedics (n=395)	Personality	Big Five Inventory (BFI)	<u>Country:</u><br>Germany<br><u>Method of Collection:</u><br>Paramedics who had been working for 5 years and on minimum duty for 520 hours per year in institutions across Germany and Austria were eligible. Surveys collected via online platform.<br><u>Analysis:</u>	Paramedic personality mean (SD) by trait:<br>- extraversion 29.06 (5.20), higher than control<br>- agreeableness 33.47 (4.40), higher than control<br>- conscientiousness 34.77 (4.67), higher than control<br>- neuroticism 21.87 (3.11), lower than control<br>- openness 34.65 (5.51), lower than control	71.43%
**Eastburg (1991) Social support, personality, and burnout in nurses**	Nurse (n=76)<br>Males (n=7)<br>Females (n=71)	Personality	PROSCAN	<u>Country:</u><br>USA<br><u>Method of Collection:</u><br>Nurses at private hospital in California,<br><u>Analysis:</u><br>descriptive statistics	Nurses personality mean (SD) by subscale, all were found to be in average range.<br>- Dominance 3.51 (1.4)<br>- extraversion 3.95 (1.44)<br>- Pace 4.86 (1.15)<br>- Conformity 4.67 (1.13)<br>- Logic 3.66 (1.14)	85.71%

**Edbor (2016) The role of emotional intelligence on personality**	Nurses (n=170)<br>Males (n=55)<br>Females (n=65)	Emotional Intelligence	Emotional Intelligence Scale (EIS)	Country:<br>India<br>Method of Collection:<br>Nurses aged between 22-30 years, working at medical institutions within central India Nagpur. Questionnaires were hand distributed and collected between June 2014 - July 2015.<br>Analysis:<br>Means sample comparisons.	Nursing EI total mean (SD) scores by gender:<br>- Males 125.23 (393.14)<br>- Females 124.72 (245.97)<br>Both demonstrate positive EI.	85.71%

**Efkarpidis (2012) A study of the emotional intelligence of employees at a District Hospital of Greece**	Doctors, Nurses and Administrative Staff (n=132)<br>Males (n=29)<br>Females (n=103)<br><br>Doctors (n=23)<br>Nursing (n=80)<br>Adm. Staff (n=29)	Emotional Intelligence	Emotional Competence Inventory (ECI)	<u>Country:</u><br>Greece<br><u>Method of Collection:</u><br>Employees at district general hospital. Data collected over 4 months.<br><u>Analysis:</u><br>Takey test (ANOVA/Post-HOC) investigate differences in participants.<br>Sum and conversion to mean, Z scores for survey results.	EI Mean scores for each subscale <u>self-awareness</u>:<br>- Doctors 35.32<br>- Nurses 35.55<br>EI Mean scores for each subscale <u>self-management</u>:<br>- Doctors 76.44<br>- Nurses 76.35<br>EI Mean scores for each subscale <u>social awareness</u>:<br>- Doctors 33.51<br>- Nurses 33.74<br>EI Mean scores for each subscale <u>managing interpersonal relationships</u>:<br>- Doctors 96.52<br>- Nurses 97.92<br>EI Mean scores for each subscale <u>cognitive thought</u>:<br>- Doctors 22.57<br>- Nurses 22.77<br><br>Nurses coming first in the factors of self-awareness, social awareness and cognitive thought.	71.43%

**Eley (2009) Exploring the temperament and character traits of rural and urban doctors.**	Rural and Urban Doctors (n=214)<br>Males (n=132)<br>Females (n=82)<br><br>Rural (n=120)<br>Males (n=84)<br>Females (n=36)<br><br>Urban (n=94)<br>Males (n=48)<br>Females (n=46)	Personality	TCI-R 140	Country:<br>Australia<br>Method of Collection:<br>Rural and urban GPs were recruited through the Australian College of Rural and Remote Medicine (ACRRM) and the Royal Australian College of General Practitioners (RACGP) from November 2006 to April 2007. Questionnaires mailed and returned with blind coded to active members in specified Rural, Remote and Metropolitan (RRMA) locations.<br>Analysis:<br>Comparison between groups utilising t-tests; ANOVA and two-way ANOVA comparing GP's and other demographic variables.	General Practitioners personality mean (SD) of Novelty seeking:<br>- Total GP sample 51.51 (7.38)<br>- Males 51.69 (7.15)<br>- Females 51.22 (7.76)<br>- Rural GPs 52.63 (7.39)<br>- Urban GPs 50.09 (7.15)<br>General Practitioners personality mean (SD) of Harm Avoidance:<br>- Total GP sample 53.93 (11.82)<br>- Males 52.85 (11.40)<br>- Females 55.66 (12.34)<br>- Rural GPs 52.52 (11.27)<br>- Urban GPs 55.72 (12.31)<br>General Practitioners personality mean (SD) of Reward Dependence:<br>- Total GP sample 65.18 (9.73)<br>- Males 63.09 (8.79)<br>- Females 68.54 (10.27)<br>- Rural GPs 64.87 (9.82)<br>- Urban GPs 65.57 (9.66)<br>General Practitioners personality mean (SD) of Persistence:<br>- Total GP sample 71.00 (8.51)<br>- Males 71.36 (8.33)<br>- Females 70.41 (8.82)	85.71%

			- Rural GPs 71.18 (8.32)<br>- Urban GPs 70.77 (8.79)<br>General Practitioners <u>personality</u> mean (SD) of <u>Self-Directedness</u>:<br>- Total GP sample 78.61 (9.23)<br>- Males 78.69 (9.72)<br>- Females 78.49 (8.43)<br>- Rural GPs 78.53 (9.73)<br>- Urban GPs 78.71 (8.60)<br>General Practitioners <u>personality</u> mean (SD) of <u>Cooperativeness</u>:<br>- Total GP sample 80.89 (7.33)<br>- Males 79.89 (7.50)<br>- Females 82.49 (6.78)<br>- Rural GPs 80.78 (6.65)<br>- Urban GPs 81.02 (8.149)<br>General Practitioners <u>personality</u> mean (SD) of <u>Self-Transcendence</u>:<br>- Total GP sample 40.12 (10.82)<br>- Males 40.15 (10.86)<br>- Females 40.07 (10.83)<br>- Rural GPs 39.88 (10.80)<br>- Urban GPs 40.44 (10.89)<br><br>Rural GPs were higher in the temperament traits of NS and lower in HA compared with the

urban GPs. All female GPs were higher in RD and CO compared with all
males, and all older GPs (over 55 years) were lower in RD compared with all younger GPs.

**Eley (2011a) Personality traits of Australian nurses and doctors: challenging stereotypes?**	Nurses and Doctors (n=426)<br><br>Doctors (n=214)<br>Males (n=132)<br>Females (n=82)<br><br>Nurses (n=212)<br>Males (n=25)<br>Females (n=187)	Personality	Temperament and Character Inventory (TCI-R 140)	Country:<br>Australia<br>Method of Collection:<br>RN's with health service district and GP's in Australian College of Rural and Remote Medicine who have been working for minimum 5 years distributed surveys via mail or online.<br>Analysis:<br>Chi-square categorical analysis, t-tests and two-way analysis of variance differences between groups.	Personality mean (SD) / population norms of Harm Avoidance:<br>- GP's 53.93 (11.522)<br>- Nurses 54.01 (10.086)<br>- Total 54.01 (10.980) / Average<br>Personality mean (SD) / population norms of Novelty seeking:<br>- GP's 51.51 (7.381)<br>- Nurses 54.34 (7.231)<br>- Total 52.92 (7.435) / Average<br>Personality mean (SD) / population norms of Reward Dependence:<br>- GP's 65.18 (9.737)<br>- Nurses 69.50 (8.725)<br>- Total 67.33 (7.435) / Very High<br>Personality mean (SD) / population norms of Persistence:<br>- GP's 71.00 (8.518)<br>- Nurses 70.87 (8.186<br>- Total 70.94 (8.345) / Very High<br>Personality mean (SD) / population norms of Self-Directedness:<br>- GP's 78.61 (9.232)	100.00%

- Nurses 74.73 (9.887)
- Total 76.68 (9.748) / Very High

Personality mean (SD) / population norms of Cooperativeness:
- GP's 80.89 (7.332)
- Nurses 79.75 (7.241)
- Total 80.32 (7.300) / Very High

Personality mean (SD) / population norms of Self-Transcendence:
- GP's 40.12 (10.826)
- Nurses 47.05 (10.052)
- Total 43.57 (10.996) / Low

Nurses were higher in the temperament traits of Novelty Seeking and Reward Dependence, lower in the character traits of Self-Directedness and Cooperativeness but higher in Self-Transcendence compared with the sample of doctors.

**Eley (2011b) Exploring temperament and character traits in nurses and nursing students in a large regional area of Australia**	Nurses (n=187)<br>Males (n=unknown)<br>Females (n=unknown)	Personality	Temperament and Character Inventory (TCI-R 140)	Country:<br>Australia<br>Method of Collection:<br>Online survey of nurses employed in public and private health services in Southeast Queensland.<br>Analysis:<br>descriptive statistics, univariate analysis.	Nurses personality mean (SD) by traits:<br>- novelty seeking 54.38 (7.2)<br>- harm avoidance 54.29 (10.24)<br>- reward dependence 69.86 (8.83)<br>- persistence 71.03 (8.28)<br>- self-directedness 75.28 (9.95)<br>- cooperativeness 80.41 (6.99)<br>- self-transcendence 46.98 (10.2)<br><br>Temperament and character trait levels congruent with a profession requiring high levels of persistence, self-directedness, cooperativeness and reward dependence.	85.71%

**Eley (2012) Why did I become a nurse? Personality traits and reasons for entering nursing.**	Registered Nurses (n=12)<br>Males (n=unknown)<br>Females (n=unknown)	Personality	Temperament and Character Inventory (TCI-R 140)	<u>Country:</u><br>Australia<br><u>Method of Collection:</u><br>Nurses working in regional are of Queensland Australia completed online survey in 2009 qualitative interviews in 2010.<br><u>Analysis:</u><br>Thematic analysis qualitative and	Nurses temperament and character trait mean (SD) / rank against pop norms scores:<br>- Novelty seeking 57 (5.7) / High<br>- Harm avoidance 56 (9.6) / High<br>- Reward Dependence 71 (11.5) / Very High<br>- Persistence 72 (7.8) / Very High<br>- Self-directedness 74 (9.6) / Very High<br>- Cooperativeness 82 (6.8) / Very High<br>- Self-transcendence 47 (12.0) / Average<br><br>No difference between RN's and nursing students.<br>'very high' in the temperament traits of Reward Dependence, Persistence and Cooperativeness and the character trait of Self Directedness; and 'high' in the temperament traits of Novelty Seeking and Harm Avoidance, and average in the character trait Self Transcendence when compared to pop norms.	100.00%

**Eley (2013) The relationship between resilience and personality traits in doctors: implications for enhancing well being**	Family Practitioners (n=479)<br>Males (n=192)<br>Females (n=287)	Personality	Temperament and Character Inventory (TCI-R 140)<br>Resilience Scale	Country:<br>Australia<br>Method of Collection:<br>Family practitioners from all states in Australia via Regional family practitioner training programs and rural and remote medicine collage offers one time invitation to complete questionnaire either paper, classroom or online.<br>Analysis:<br>Relationship between measures explored through Pearson product-moment correlations coefficients and multiple regression.	Family Practitioners temperament and character trait mean (SD) / rank against pop norms scores:<br>- Novelty seeking 53.25 (7.79) / Average<br>- Harm avoidance 54.65 (11.97) / Average<br>- Reward Dependence 69.80 (9.89) / Very High<br>- Persistence 71.34 (9.45) / Very High<br>- Self-directedness 77.08 (9.34) / Very High<br>- Cooperativeness 80.71 (7.95) / Very High<br>- Self-transcendence 42.34 (10.92) / Low<br><br>Strong to medium positive correlations between Resilience and Self-directedness, Persistence, and Cooperativeness and negative with Harm Avoidance.<br>Resilience was associated with a personality trait pattern that is mature, responsible, optimistic, persevering, and cooperative.<br>Individual differences in	100.00%

personality explained 39% of the variance in resilience. The three traits which contributed significantly to this variance were Self-directedness, Persistence and Harm Avoidance.

**Eley (2015) Who attracts whom to rural general practice? Variation in temperament and character profiles of GP registrars across different vocational training pathways**	Registrars (n=451)<br>Males (n=<br>Females (n=	Personality	Temperament and Character Inventory (TCI-R 140)	<u>Country:</u><br>Australia<br><u>Method of Collection:</u><br>Cross sample of GP registrars between 2011 - 2013 across Australia on rural training pathways. Survey distributed by either hard copy, classroom setting or online (SurveyMonkey).<br><u>Analysis:</u><br>Descriptive statistics, chi-squared tests, MANOVAs, ANOVA, post-hoc analyses	Registrars personality mean (SD) by traits:<br>- novelty seeking 2.67 (0.40)<br>- harm avoidance 2.71 (0.62)<br>- reward dependence 3.47 (0.49)<br>- persistence 3.56 (0.48)<br>- self-directedness 3.83 (0.49)<br>- cooperativeness 4.02 (0.41)<br>- self-transcendence 2.64 (0.67)<br><br>Registrars are a relatively homogenous in their overall trait levels as compared to population norms.	85.71%
**Ellershaw (2016) Conscientiousnes s, openness to experience and extraversion as predictors of nursing work performance: a facet-level analysis.**	Nurses (n=393)<br>Males (15%)<br>Females (85%)	Personality	NEO Five-Factor Inventory (NEO-FFI)	<u>Country:</u><br>Australia<br><u>Method of Collection:</u><br>Nurses from health care organisations across Australia recruited via online web panel during February 2013.<br><u>Analysis:</u><br>Descriptive statistics, path analyses determine relationships between variables.	Nursing personality mean (SD) by subscales:<br>- extraversion 40.71 (6.12)<br>- openness to experience 38.82 (5.42)<br>- conscientiousness 46.99 (6.71)<br><br>Conscientiousness was the strongest driver of work role performance across all the indicators, with extraversion also strongly associated with work role performance. Openness to experience	100.00%

					related to all of the work role performance indicators.	
**Engin (2009) Effect of self-awareness education on the self-efficacy and sociotropy-autonomy characteristics of nurses in a psychiatry clinic**	Nurses (n=22)<br>Males (n=unknown)<br>Females (n=unknown)	Personality	Sociotropy–autonomy Scale (SAS)	<u>Country:</u><br>Turkey<br><u>Method of Collection:</u><br>Survey provided pre and post education program.<br><u>Analysis:</u><br>descriptive statistics, Wilcoxon test.	Nurses mean (SD) by personality scale:<br>- sociotropy 56.181 (16.126)<br>- autonomy 70.954 (15.441)	71.43%

**Erdenk (2017) Do personality traits of nurses have an effect on conflict management strategies?**	Nurses (n=237)<br>Males (10%)<br>Females (90%)	Personality	Five-Factor Personality Inventory (FFPI)	<u>Country:</u><br>Turkey<br><u>Method of Collection:</u><br>Nurses working in three Ministry of Health hospitals between December 2013 - February 2014.<br><u>Analysis:</u><br>Cronbach's alpha coefficient, frequency and percentage distributions, descriptive statistics, Pearson product-moment correlation analysis, simple linear regression analysis, t-test were used in the evaluation of the data.	Personality traits of nurses mean (SD) by subscale:<br>- extraversion 3.18 (0.46)<br>- conscientiousness 3.31 (0.45)<br>- openness 3.31 (0.52)<br>- agreeableness 3.15 (0.41)<br>- emotional disability 2.96 (0.51)<br><br>personality traits of nurses were mostly 'conscientiousness' and 'openness'.	85.71%

**Erkutlu (2012) The impact of team empowerment on proactivity: the moderating roles of leader's emotional intelligence and proactive personality**	Nurses (n=910)	Emotional Intelligence Personality	Proactive Personality Scale Bar-On's Emotional Quotient Inventory (EQ-I) - 133 item	Country: Turkey Method of Collection: Nurses at 12 university hospitals in Turkey who had been in team with clear identify for minimum 6 months were eligible. Recruitment occurred via physical visits to hospitals and delivery and collected physical copies of survey. Analysis: descriptive statistics and correlations.	Nurses EI mean (SD) of 5.22 (1.28) and proactive personality mean (SD) of 4.02 (0.78).	100.00%
**Ezzatabadi (2012) Nurses' emotional intelligence impact on the quality of hospital services**	Nurses (n=243) Males (n=35) Females (n=208)	Emotional Intelligence	Cyberia Shrink 33 items questionnaire	Country: Iran Method of Collection: Nurses from 3 academic hospitals Analysis: Descriptive statistics, correlations	Nursing EI mean (SD) by hospital group: - group 1 3.88 (0.58) - group 2 3.41 (0.41) - group 3 4.30 (0.50) Nurses' emotional intelligence had a direct effect on the hospital services quality.	71.43%

**Farčić (2020) Personality traits of core self-evaluation as predictors on clinical decision-making in nursing profession**	Nurses (n=584)<br>Males (n=52)<br>Females (n=532)	Personality	Core Self-evaluations Scale (CSES)	<u>Country:</u><br>Croatia<br><u>Method of Collection:</u><br>Nurses at university hospital between April 2016 - January 2017. Questionnaires distributed and collected by researchers during their shift breaks.<br><u>Analysis:</u><br>Descriptive statistics, Pearson's correlations coefficients.	Nurses personality total mean (SD) (range) of 39.2 (9.5) (23-64) and subscale scores of:<br>- emotional stability 6.9 (2.0) (3-15)<br>- locus of control 10.1 (2.1) (2-15)<br>- self-efficacy 11.5 (1.5) (6-15)<br>- self-esteem 10.8 (1.6) (6-15)<br><br>The average values of the subscales of core self-evaluation lean slightly in the positive direction, except for the area of emotional stability.	71.43%
**Farooq (2020) Proactive Personality, Motivation and Employee Creativity in the Public Sector Hospitals of Peshawar City**	Doctors (n=220)<br>Males (n=<br>Females (n=	Personality	Proactive Personality Scale	<u>Country:</u><br>Pakistan<br><u>Method of Collection:</u><br>Doctors in 6 public sector hospitals in Peshawar City in BPS 17 and above were sent surveys.<br><u>Analysis:</u><br>descriptive statistics, regression and mediation analysis	Doctors personality mean (SD) 4.83 (0.76).<br><br>Positive significant relationship between proactive personality and creativity and motivation.	71.43%

**Farzianpour (2015) Relationship Between Shift Work and Personality Traits of Nurses and Their Coping Strategies.**	Nurses (n=305)<br>Males (n=78)<br>Females (n=227)	Personality	Eysenck Personality Questionnaire (EPQ)	<u>Country:</u><br>Iran<br><u>Method of Collection:</u><br>Nurses in 6 non-governmental hospital in Tehran in 2014, selected through two-stage cluster random sampling.<br><u>Analysis:</u><br>Descriptive and inferential statistics including paired t-tests, person's correlations, ANOVA, Mann-Whitney and Kruskal-Wallis test.	43.6% of nurses were introverts and 56.4% extroverted.	71.43%

**Fatima (2008) Explaining Doctors' Work Values from Personality Traits and Hospital Characteristics**	Doctors (n=200)<br>Males (64%)<br>Females (36%)<br><br>Public (n=100)<br>non-government (n=100)	Personality	NEO Five-Factor Inventory (NEO-FFI)	Country:<br>Pakistan<br>Method of Collection:<br>100 doctors randomly from public and non-government hospital, every third (public) and two after ever third (non-government) doctors on HR list of employed doctors were selected to participate.<br>Analysis:<br>Independent t-tests.	Doctors personality mean (SD) for neuroticism:<br>- Public 31.12 (4.10)<br>- Non-government 33.14 (4.30)<br>Doctors personality mean (SD) for extraversion:<br>- Public 40.22 (7.02)<br>- Non-government 35.92 (4.41)<br>Doctors personality mean (SD) for openness:<br>- Public 39.85 (5.70)<br>- Non-government 35.77 (4.99)<br>Doctors personality mean (SD) for agreeableness:<br>- Public 39.28 (5.22)<br>- Non-government 36.21 (5.13)<br>Doctors personality mean (SD) for conscientiousness:<br>- Public 39.73 (5.25)<br>- Non-government 35.37 (5.34)<br><br>Agreeableness and Conscientiousness emerged as the major personality traits of all doctors. Personality explained cognitive, affective and instrumental work values in nongovernment more than in public hospitals.	71.43%

**Foji (2020) The effect of emotional intelligence training on general health promotion among nurse**	Nurses (n=135)<br>Males (n=<br>Females (n=	Emotional Intelligence	Bar-On's Emotional Quotient Inventory (EQ-I) - 90 item	<u>Country:</u><br>Iran<br><u>Method of Collection:</u><br>Nurse in hospitals of Sabzevar, survey completed pre and post training session.<br><u>Analysis:</u><br>descriptive statistics, t-tests, person's correlations	Nurse baseline global EI mean (SD) of 327.02 (33.44)	57.14%
**Frias (2021) The impact of an emotional intelligence training program on transformational leadership**	Nurse Managers (n=45)<br>Males (n=3)<br>Females (n=42)	Emotional Intelligence	Trait Emotional Intelligence Questionnaire-Short Form (TEIQue-SF)	<u>Country:</u><br>Unknown<br><u>Method of Collection:</u><br>Quasi-experimental pre/post survey.<br><u>Analysis:</u><br>Nil specific details.	Nursing global EI mean (SD) of 5.77 (0.55) and by subscale:<br>- emotionality 5.84(0.57)<br>- self-control 5.33 (0.71)<br>- sociability 5.45 (0.62)<br>- well-being 6.19 (0.62)<br><br>EI intervention resulted in improvements across all EI scales.	71.43%
**Froutan (2018) Relationship between resilience and personality traits in paramedics**	Paramedics (n=252)<br>Males (n=unknown)<br>Females (n=unknown)	Personality	NEO-Five Factor Inventory-Short Form (NEO-FFI-SF)	<u>Country:</u><br>Iran<br><u>Method of Collection:</u><br>Survey distributed to paramedics in Eastern Iran between May-Oct 2016.<br><u>Analysis:</u><br>descriptive statistics, spearman correlation coefficient	Paramedics personality mean (SD) by trait:<br>- neuroticism 20.60 (5.10)<br>- extraversion 26.50 (5.00)<br>- openness 22.70 (3.70)<br>- agreeableness 26.60 (4.70)<br>- conscientiousness 29.10 (5.20)<br><br>The highest scores was associated with	85.71%

					conscientiousness, lowest scores associated with neuroticism.	
**Fujino (2015) The relationship between characteristics of nursing performance and years of experience in nurses with high emotional intelligence.**	Nursing (n=1045)<br>Males (n=59)<br>Females (n=986)	Emotional Intelligence	Emotional Intelligence Scale (EQS)	<u>Country:</u><br>Japan<br><u>Method of Collection:</u><br>Nurses working at general hospital approached via nursing directors/leaders distributed survey and collected 2 weeks post distribution between November 2012 to March 2011.<br><u>Analysis:</u><br>Descriptive statistics, Pearson's product-moment correlations coefficients relationships.	Total nursing EI mean (SD) (range) of 113.3 (31.3) (27-218), subscale scores:<br>- intrapersonal 37.5 (10.6) (7-73)<br>- interpersonal 41.1 (11.8 (6-78)<br>- situational 34.8 (34.8) (3-78)<br><br>Significant positive correlation between EI and nursing performance. Those who had high EI also had high nursing performance.	85.71%

**Furukawa (2021) The relationship between leadership behaviours of ward nurse managers and teamwork competency of nursing staff: A cross-sectional study in Japanese hospitals**	Nurses (n=960)<br>Males (n=92)<br>Females (n=857)	Emotional Intelligence	Nursing Manager's Leadership Behaviour Scale	<u>Country:</u><br>Japan<br><u>Method of Collection:</u><br>Questionnaire distributed to 13 hospitals with >200 beds, between May and August 2019.<br><u>Analysis:</u><br>Multiple regressions	Nursing leadership emotional intelligence behaviour by subscale:<br>- Staff nurturing and support 37.3 (12.0) (score range from 13-65)<br>- Emotional control 43.9 (14.3) (score range from 14-70)<br>- Care for patients 23.7 (6.3) (score range from 7-35)<br><br>Nurses' teamwork competencies were primarily related to emotional intelligence leadership	100.00%

**Gallardo (2018) Personality Predictors of Compassion Fatigue in Long-term Care Environments**	Registered nurses, licensed vocational nurses, certified medication aides, and certified nurse aides (n=234)<br>Males (n=unknown)<br>Females (n=unknown)<br><br>RN's (n=42)<br>Licensed vocational nurses (n=53)<br>certified medication aides (n=29)<br>Certified nursing aides (N=78)<br>Missing (n=32)	Personality	NEO Five-Factor Inventory (NEO-FFI)	Country:<br>USA<br>Method of Collection:<br>Direct patient care workers across Texas caring for long-term patients, recruited via team leaders and social media over 3 month period. Survey completed electronically.<br>Analysis:<br>Descriptive statistics and multiple regression analysis relationships.	Total Personality mean (SD) by subscales:<br>- Openness 30.0 (5.6)<br>- Conscientiousness 36.2 (7.6)<br>- Extraversion 30.2 (6.6)<br>- Agreeableness 34.3 (7.1)<br>- Neuroticism 19.1 (8.0)<br><br>Personality was a significant predictor of compassion fatigue. Big Five personality traits were significantly related to a risk for compassion fatigue, accounting for 37% of the variance.	71.43%

**Gelkop (2022) Are emotional intelligence and compassion associated with nursing safety and quality care? A cross-sectional investigation in paediatric settings**	Nurses (n= 71)	Emotional Intelligence	Trait Emotional Intelligence Questionnaire-Short Form (TEIQue-SF)	Country:<br>Israel<br>Method of Collection:<br>cross-sectional study, registered nurses of children hospitalised in paediatric ward. Data collected between April and June 2018.<br>Analysis:<br>Descriptive statistics, t-tests, hierarchical multiple regression.	Nurses global EI mean (SD) of 5.33 (0.61).	100.00%
**Gertis (2004) Emotional intelligence and adaptive success of nurses caring for people with mental retardation and severe behaviour problems.**	Nurses (n=380)<br>Males (n=146)<br>Females (n=234)	Emotional Intelligence	Bar-On Emotional Quotient Inventory	Country:<br>Netherlands<br>Method of Collection:<br>Nurses from 56 Dutch facilities for people with mental retardation, recruited from board of 96 residents. Researcher coordinated the collection and completion of questionnaires.<br>Analysis:<br>Bivariate correlations and binary logistic regression between variables.	Total EI mean (SD) by gender:<br>- Male 107.24 (10.66)<br>- Female 107.15 (11.54)<br>Detailed subcategory mean (SD) see quantitative analysis spreadsheet.<br><br>Male nurses scored significantly higher on Intrapersonal composite scale, Assertiveness, Self-Regard, Stress Management and Stress Tolerance.<br>Female nurses scored significantly higher on Interpersonal, Empathy, Interpersonal Relationship, and Social Responsibility.<br>No significant difference was	71.43%

					found between the male and female for total EI score.<br>Negative correlation was found between emotional intelligence and both burnout and psychopathology.	
**Geuens (2017) Vulnerability to burnout within the nursing workforce-The role of personality and interpersonal behaviour.**	Nurses (n=587)<br>Males (n=unknown)<br>Females (n=unknown)	Personality	NEO Five-Factor Inventory (NEO-FFI)	Country:<br>Belgium<br>Method of Collection:<br>Nurses from 16 General Hospitals via stratified sampling between November 2012 - July 2013.<br>Analysis:<br>Multiple linear regressions	Nurses personality big five factors % low to very low scores:<br>- Neuroticism 20.7%<br>- Extraversion 9.3%<br>- Openness 13.3%<br>- Agreeableness 10.9%<br>- Conscientiousness 12.3%<br><br>Nurses personality big five factors % high to very high scores:<br>- Neuroticism 19.8%<br>- Extraversion 40.1%<br>- Openness 25.5%	85.71%

					- Agreeableness 37.3%<br>- Conscientiousness 31.1%	
**Geyer (2018) Relationship of nurses' intrapersonal characteristics with work performance and caring behaviours: A cross-sectional study**	Nurses (n=218)<br>Males (n=13)<br>Females (n=205)	Personality	Core Self Evaluations Scale (CSES)	Country:<br>Unknown<br>Method of Collection:<br>Researchers distributed to RN's and collected 3 days later between Sept 2013 - March 2014.<br>Analysis:<br>Descriptive statistics,	Nurses personality mean (SD) of 61.68 (10.54)<br><br>Nurses had moderately high scores for personality.	85.71%

**Ghasemian (2017) Relationship between Personality Types and Stress: A Comparative Study among Male and Female Nurses in Health Care Setting**	Nurses (n=100)<br>Males (n=18)<br>Females (n=82)	Personality	Type A/B Behavioural Patten Scale (ABBPS)	<u>Country:</u><br>India<br><u>Method of Collection:</u><br>Nurses working in different hospital settings in Mysore and Bangalore region via simple random sampling.<br><u>Analysis:</u><br>Pearson's correlations and independent t-tests	Nurses Type A personality mean (SD) of 60.97 (9.23)<br>Nurses Type B personality mean (SD) of 37.6 (9.58)<br><br>Type A positively correlated with stress.<br>Type B is negatively correlated with stress.<br><br>No significant difference between genders for type A or Type B.	71.43%
**Giménez-Espert (2017) Emotional intelligence in nurses: the Trait Meta-Mood Scale**	Nurses (n=530)<br>Males (n=unknown)<br>Females (n=unknown)	Emotional Intelligence	Trait Meta Mood Scale (TMMS-24)	<u>Country:</u><br>Spain<br><u>Method of Collection:</u><br>Nurses from 11 Spanish hospital in Valencian community with permanent positions were recruited.<br><u>Analysis:</u><br>Internal validity, convergent validity, discriminate validity and variance to establish if measure validated in Spanish nurse population.	Nurses EI scores in order of highest to lowest by subscale mean (SD):<br>- Emotional clarity 3.84 (0.69)<br>- emotional repair 3.82 (0.77)<br>- Emotional attention 3.58 (0.77).<br><br>Supports the use of TMMS-24 in the Spanish nursing context	71.43%

**Giménez-Espert (2018) The role of empathy and emotional intelligence in nurses' communication attitudes using regression models and fuzzy-set qualitative comparative analysis models.**	Nurses (n=460)<br>Males (n=112)<br>Females (n=348)	Emotional Intelligence	Trait Meta Mood Scale (TMMS-24)	Country:<br>Spain<br>Method of Collection:<br>Surveys were distributed to nurses in six Spanish hospitals from the Valencian Community between September 2015–February 2016.<br>Analysis:<br>Relationship between emotional intelligence and empathy analysed by traditional regression models and fuzzy-set qualitative comparative analysis models.	Nursing EI mean (SD):<br>- Attention 757.86 (783.82) (score min-max: 1-3125)<br>- Clarity 84935.15 (111753.89) (score min-max 8-390625)<br>- Reparation 4604.05 (4679.15) (score min-max 2-15625)<br>Nursing Empathy mean (SD):<br>- Perspective-taking 1061467.10 (705515.65) (score min-max 2-1953125)<br>- Compassionate care 27.46 (67.23) (score min-max 1-625)<br>- Thinking as the patient 4.96 (5.28) (score min-max 1-25)<br><br>Empathy and emotional intelligence are predictors of nurses' attitudes towards communication.	85.71%

**Giménez-Espert (2019) Impact of work aspects on communication, emotional intelligence and empathy in nursing**	Nurses (n=450)<br>Males (n=unknown)<br>Females (n=unknown)	Emotional Intelligence	Trait Meta Mood Scale (TMMS-24)	Country:<br>Spain<br>Method of Collection:<br>Nurses at 7 public hospitals in Valencia, filled in paper forms and then submitted at designed polling places within their workplace.<br>Analysis:<br>descriptive statistics, one-factor ANOVA, hierarchical multiple linear regression	Nurses EI mean (SD) by subscale and contract type:<br><br>Temporary Contract nurses<br>- emotional attention 3.64 (0.77)<br>- emotional clarity 3.86 (0.55)<br>- emotional repair 3.80 (0.70)<br><br>Interim Contract nurses<br>- emotional attention 3.46 (0.82)<br>- emotional clarity 3.81 (0.71)<br>- emotional repair 3.83 (0.84)<br><br>Permanent contract nurses<br>- emotional attention 3.62 (0.74)<br>- emotional clarity 3.83 (0.72)<br>- emotional repair 3.80 (0.74)	85.71%

**Giménez-Espert (2020) Empathy, emotional intelligence, and communication in Nursing: The moderating effect of the organizational factors**	Nurses (n=286)<br>Males (n=unknown)<br>Females (n=unknown)	Emotional Intelligence	Trait Meta Mood Scale (TMMS-24)	<u>Country:</u><br>Spain<br><u>Method of Collection:</u><br>Nurses at 3 hospitals in Valencia, Spain, who were working at the time of data collection.<br><u>Analysis:</u><br>descriptive statistics, t-tests, multiple linear regressions.	Nurses EI mean (SD) by subscale and contract type:<br><br><u>Temporary</u> Contract nurses<br>- emotional attention 3.46 (0.74)<br>- emotional clarity 3.76 (0.61)<br>- emotional repair 3.77 (0.74)<br><br><u>Permanent</u> contract nurses<br>- emotional attention 3.57 (0.71)<br>- emotional clarity 3.74 (0.73)<br>- emotional repair 3.77 (0.76)<br><br>General Hospital service<br>- emotional attention 3.46 (0.74)<br>- emotional clarity 3.76 (0.61)<br>- emotional repair 3.77 (0.74)<br><br>type of contract and service exert an influence on the attitudes towards communication, empathy, and EI, with the consequent possibility of arising as risk or protection factors.	71.43%

**Gleason (2020) The Job Demands-Resources Model as a Framework to Identify Factors Associated with Burnout in Surgical Residents**	Surgical Residents (n=60)<br>Males (n=29)<br>Females (n=31)	Emotional Intelligence	Trait Emotional Intelligence Questionnaire-Short Form (TEIQue-SF)	Country:<br>USA<br>Method of Collection:<br>Electronic survey between May-June 2018.<br>Analysis:<br>Descriptive statistics, bivariate analysis, correlations	Surgical Resident EI (mean) SD by prevalence of burnout and subscale:<br><br>Burnout present (yes) global EI 5.18 (0.52)<br>- well-being 5.58 (0.67)<br>- self-control 4.89 (0.7)<br>- emotionality 5.14 (0.84)<br>- sociability 4.93 (0.71)<br><br>Burnout present (No) global EI 5.64 (0.59)<br>- well-being 6.1 (0.63)<br>- self-control 5.21 (0.86)<br>- emotionality 5.65 (0.68)<br>- sociability 5.2.8 (0.82)	57.14%

**Głębocka (2019) Stress and Dehumanizing Behaviours of Medical Staff Toward Patients.**	Nurses (n=96)<br>Males (n=unknown)<br>Females (n=unknown)<br><br>Experimental (n=49)<br>Control (n=47)	Personality	Eysenck Personality Questionnaire - Revised (EPQ-R)	Country:<br>Poland<br>Method of Collection:<br>Nurses participating in PD session with end exam completed survey prior to exam (experimental) stress provoking environment.<br>Control group had not exam and therefore emotion-neutral environment.<br>Analysis:<br>Multivariate analysis (MANOVA) for repeated measures and post hoc Schiffer for comparisons between groups.	Personality mean (SD) by subscale for experimental group:<br>- Lying scale 12.2 (3.1)<br>- Neuroticism 14.9 (5.0)<br>- Extraversion 14.2 (3.5)<br>- Psychoticism 10.0 (3.4)<br><br>Personality mean (SD) by subscale for control group:<br>- Lying scale 12.2 (3.7)<br>- Neuroticism 15.7 (4.7)<br>- Extraversion 14.5 (4.2)<br>- Psychoticism 10.4 (3.5)<br><br>No significant intergroup differences for personality traits evaluated with the EPQ-R questionnaire such as neuroticism, extraversion, and psychoticism.<br>Anxiety-prone condition enhanced the prospective nurses' attitude toward patent dehumanizing behaviours.	71.43%

**Gokhale (2019) Are empathy and emotional intelligence missing in dental practitioner's toolkit in Saudi Arabia? A cross-sectional study.**	Dentists (n=186)<br>Males (n=154)<br>Females (n=32)	Emotional Intelligence	Schutte's Emotional Intelligence Scale	Country:<br>Saudi Arabia<br>Method of Collection:<br>Dentists in Abha and Kahamis Mushayat cities in September 2018, as per registration board.<br>Analysis:<br>Mann-Whitney test and Kruskal-Wallis group comparisons, Correlations between measures using Spearman's rank order correlation tests.	Dentist EI mean (SD) total score of 151.17 (15.846) with breakdown by gender:<br>- Male 151.03 (15.869)<br>- Female 151.84 (15.836)<br><br>No statistically significant differences in EI scores between gender.<br>Emotional intelligence was found to have a strong positive correlation with empathy scores. Dentists with high empathy scores seem to show high EI levels.	71.43%
**Gómez-Polo (2021) Epidemiological study on burnout in Spanish dentists: Underlying psychological factors**	Dentists (n=1298)<br>Males (n=unknown)<br>Females (n=unknown)	Personality	NEO Five-Factor Inventory (NEO FFI)	Country:<br>Spain<br>Method of Collection:<br>Nil specific collection strategy provided.<br>Analysis:<br>descriptive statistics, multivariate analysis, binary logistic regression	Dentist personality mean (SD) by trait:<br>- neuroticism 33.8 (9.2)<br>- extraversion 40.2 (7.3)<br>- openness 40.1 (6.8)<br>- agreeableness 42.1 (5.9)<br>- conscientiousness 45.9 (6.0)	85.71%

**Gorgas (2015) Teaching Emotional Intelligence: A Control Group Study of a Brief Educational Intervention for Emergency Medicine Residents**	Emergency Medicine Residents (n=33)<br>Males (n=17<br>Females (n=16)	Emotional Intelligence	Hay 360 Emotional Competence Inventory (Hay 360 ECI)	Country:<br>USA<br>Method of Collection:<br>Residents at three levels of program, completed pre-testing prior to an EI interventions.<br>Analysis:<br>descriptive statistics, ANOVA	Emergency medicine residents global EI mean (SD) of 64.4 (11.6)	85.71%
**Görgens-Ekermans (2012) Emotional intelligence as a moderator in the stress-burnout relationship: a questionnaire study on nurses.**	Nurses (n=122)<br>Males (10.7%)<br>Females (89.3%)	Emotional Intelligence	Swinburne University Emotional Intelligence Test (SUEIT)	Country:<br>South Africa<br>Method of Collection:<br>Random sample drawn from private healthcare employment agency, were phoned to participate. Research packs delivered to 4 hospitals, were participants worked. Completed questionnaires returned to collection boxes.<br>Analysis:<br>Pearson's product-moment correlations and multiple regression analysis to explore relationships between variables.	Nurses EI mean (SD) total score of 213.35 (18.04) and by subscales:<br>- emotional recognition and expression 31.76 (5.95)<br>- understanding others emotions 62.79 (8.82)<br>- emotions direct cognition 39.43 (6.55)<br>- emotional management 36.85 (5.31)<br>- emotional control 27.01 (4.88)<br><br>Higher EI is significantly related with lower stress and burnout. The moderator effect of EI in the stress–burnout relationship suggests that enhanced EI may help diminish burnout	71.43%

					development when chronic stress is experienced.	
**Gou (2021) A multilevel analysis of the impact of group organisational citizenship behaviour on nurse-patient relationship: The mediating effect of work engagement and**	Nurses (n=1246)<br>Males (n=unknown)<br>Females (n=unknown)	Emotional Intelligence	Wong and Law Emotional Intelligence Scale (WLEIS) - 7 point scale	<u>Country:</u><br>China<br><u>Method of Collection:</u><br>Nurses from 55 nursing groups in a China hospital.<br><u>Analysis:</u><br>Descriptive statistics, multilevel analysis	Nurses global EI mean (SD) of 5.06 (0.69).	100.00%

**the moderating effect of emotional intelligence**

**Grandy (1996) Predicting dentists' career choices using the Myers-Briggs type indicator.**	Dentists (n=472)<br>Males (n=446)<br>Females (n=26)<br><br>General Practitioner (n=381)<br>Specialist (n=91)	Personality	Myers–Briggs Type Indicator (MBTI)	Country:<br>USA and Canada<br>Method of Collection:<br>Dentists from Creighton University dental school over a 20 year period, across 45/50 USA states, Canada and military stations overseas with minimum of 4 years' experience received survey in the amil.<br>Analysis:	Dentist percentage personality type for ISTJ:<br>- general practitioner 23.4%<br>- specialist 20.9%<br>Dentist percentage personality type for ISFJ:<br>- general practitioner 9.4%<br>- specialist 13.2%<br>Dentist percentage personality type for INFJ:<br>- general practitioner 3.9%<br>- specialist 6.6%<br>Dentist percentage personality type for INTJ:<br>- general practitioner 6.6%<br>- specialist 4.4%<br>Dentist percentage personality type for ISTP:<br>- general practitioner 5.5%<br>- specialist 4.4%<br>Dentist percentage personality type for ISFP:<br>- general practitioner 3.1%<br>- specialist 1.1%<br>Dentist percentage personality type for INFP:<br>- general practitioner 3.4%<br>- specialist 4.4%<br>Dentist percentage personality type for INTP:<br>- general practitioner 3.4%	71.43%

- specialist 4.4%

Dentist percentage personality type for ESTP:
- general practitioner 3.1%
- specialist 2.2%

Dentist percentage personality type for ESFP:
- general practitioner 1.1%
- specialist 0.0%

Dentist percentage personality type for ENFP:
- general practitioner 2.6%
- specialist 2.2%

Dentist percentage personality type for ENTP:
- general practitioner 3.1%
- specialist 3.3%

Dentist percentage personality type for ESTJ:
- general practitioner 13.9%
- specialist 14.3%

Dentist percentage personality type for ESFJ:
- general practitioner 6.6%
- specialist 3.3%

Dentist percentage personality type for ENFJ:
- general practitioner 2.9%
- specialist 6.6%

Dentist percentage personality type for ENTJ:

- general practitioner 7.6%
- specialist 8.8%

Dentist percentage personality characteristic for Extroversion:
- general practitioner 41.2%
- specialist 40.7%

Dentist percentage personality characteristic for Introversion:
- general practitioner 58.8%
- specialist 59.3

Dentist percentage personality characteristic for Sensing:
- general practitioner 66.4%
- specialist 59.3%

Dentist percentage personality characteristic for Intuitive:
- general practitioner 33.6%
- specialist 40.7%

Dentist percentage personality characteristic for Thinkers:
- general practitioner 66.7%
- specialist 62.6%

Dentist percentage personality characteristic for Feelers:
- general practitioner 33.3%
- specialist 37.4%

Dentist percentage personality characteristic for Judgers:
- general practitioner 74.3%
- specialist 78%

Dentist percentage personality

characteristic for Perceptive:
- general practitioner 25.7%
- specialist 22.0%

Dentist percentage personality temperaments for Intuitive-Feelers:
- general practitioner 12.9%
- specialist 19.8%

Dentist percentage personality characteristic for Intuitive-Thinkers:
- general practitioner 20.7%
- specialist 20.9%

Dentist percentage personality characteristic for Sensing-Judging:
- general practitioner 53.3%
- specialist 51.6%

Dentist percentage personality characteristic for Sensing-Perceptive:
- general practitioner 13.1%
- specialist 7.7%

No significant difference between general practitioners and specialists.

**Greinacher (2022) The impact of personality on intention to leave the nursing profession: A structural equation model**	Nurses (n=518)<br>Males (n=95)<br>Females (n=423)	Personality	10-Item Big Five Inventory (BFI-10)	<u>Country:</u><br>Germany<br><u>Method of Collection:</u><br>Nil specific collection strategy provided.<br><u>Analysis:</u><br>descriptive statistics, ANOVA, Welch test, chi-squared tests, RMSEA, SRMR	Nursing personality mean (SD) by trait:<br>- openness 3.53 (0.98)<br>- extraversion 3.49 (1.00)<br>- neuroticism 2.73 (0.94)<br>- conscientiousness 4.08 (0.77)<br>- agreeableness 3.41 (0.81)<br><br>Openness and neuroticism had positive effect on nurses intent to leave.	85.71%
**Habib (2012) Emotional Intelligence as Predictor of Life Satisfaction among Nurses: Mediating Role of Spiritual Wellness**	Nurses (n=300)<br>Males (n=unknown)<br>Females (n=unknown)	Emotional Intelligence	Wong and Law Emotional Intelligence Scale (WLEIS)	<u>Country:</u><br>Pakistan<br><u>Method of Collection:</u><br>Purposive convenient sampling from different hospitals of Islamabad.<br><u>Analysis:</u><br>Descriptive statistics, Alpha reliability coefficients, zero order correlations, multiple Regression and Hierarchal Multiple Regression analysis	Nursing EI mean (SD) of 61.77 (8.86).<br><br>EI had a significant positive effect on life satisfaction.	85.71%

**Hagan (1999) The personality of dietetics**	Dieticians (n=84)<br>Males (n=unknown)<br>Females (n=unknown)	Personality	Myers-Briggs Type Indicator	<u>Country:</u><br>USA<br><u>Method of Collection:</u><br>Surveys collected between September 1991 and September 1997.<br><u>Analysis:</u><br>Frequencies	Dieticians percentage and number (n) by MBTI types:<br>- ISTJ 11.9% (10)<br>- ISFJ 11.9% (10)<br>- INFJ 8.3% (7)<br>- INTJ 3.6% (3)<br>- ISTP 0% (0)<br>- ISFP 2.4% (2)<br>- INFP 2.4% (2)<br>- INTP 7.1% (6)<br>- ESTP 2.4% (2)<br>- ESFP 2.4% (2)<br>- ENFP 3.6% (3)<br>- ENTP 3.5% (3)<br>- ESTJ 13.1% (11)<br>- ESFJ 16.7% (14)<br>- ENFJ 3.6% (3)<br>- ENTJ 7.1% (6)<br><br>Largest percentage is ESFJ. All 16 MBTI types except (ISTP) were present in the dietetic internship program for the 7 year period.	85.71%

**Hansen (1995) Nurse manager personal traits and leadership characteristics.**	Nurse Unit Managers (n=99)<br>Males (4.1%)<br>Females (95.9%)	Personality	NEO Personality Inventory	<u>Country:</u><br>USA<br><u>Method of Collection:</u><br>Data pulled from database of four large (>300) beds) general acute care hospitals of nursing manager leadership retention study.<br><u>Analysis:</u><br>Relationship between personality, motivation to manage and leadership style.	Nursing managers personality characteristics mean (SD):<br>- Neuroticism 77.61 (20.62)<br>- Extroversion 115.97 (17.20)<br>- Openness 115.70 (16.25)<br>- Agreeableness 51.06 (6.46)<br>- Conscientiousness 49.22 (8.60)<br><br>Personality was linked modestly to motivation to manage and selected aspects of leadership style.	85.71%

**Harper (2012) The emotional intelligence profile of successful staff nurses.**	Nurses (n=42)<br>Males (n=1)<br>Females (n=41)	Emotional Intelligence	Bar-on Emotional Quotient Inventory (EQ-i)	<u>Country:</u><br>USA<br><u>Method of Collection:</u><br>Registered nurses who work with student nurses in clinical setting in six community hospitals in California and Texas. Selected by nurses leaders based on expert patient care, with minimum 2 years' experience. Questionnaire completed online.<br><u>Analysis:</u><br>Descriptive statistics, Pearson's correlations and variance analysis between groups.	Total EI Mean (range) of 102.45 (90-109).<br>- 19% below average<br>- 31% higher than average<br><br>EI subscale mean scores in order highest to lowest:<br>- stress tolerance 106.43<br>- problem solving 104.17<br>- self-regard 103.93<br>- self-actualisation 103.64<br>- social responsibility 102.93<br>- reality testing 102.10<br>- empathy 101.81<br>- happiness 101.69<br>- assertiveness 101.60<br>- optimism 101.48<br>- emotional self-awareness 101.24<br>- impulse control 99.88<br>- interpersonal relations 99.62<br>- independence 99.17<br>- flexibility 99.02<br><br>Successful staff nurses have average or higher levels of EI. Empathy among these nurses declines with age.	71.43%

**Harris (1985) Personality types of family practice residents as measured by the Myers-Briggs type indicator**	Doctors (n=75)<br>Males (n=unknown)<br>Females (n=unknown)<br><br>Rural Doctors (n=20)<br>Family Practice Residents (n=55)	Personality	Myers–Briggs Type Indicator (MBTI)	<u>Country:</u><br>USA<br><u>Method of Collection:</u><br>All incoming residents for the years 1977-1980 were given MBTI survey to completed.<br><u>Analysis:</u><br>Descriptive statistics	<u>Rural practitioners</u> personality traits:<br>- Extrovert 50%<br>- Introvert 50%<br>- sensing 70%<br>- Intuitive 30%<br>- Thinking 60%<br>- Feeling 40%<br>- Judging 65%<br>- Perceptive 35%<br><br><u>Family Practice Residents</u> personality traits:<br>- Extrovert 45%<br>- Introvert 55%<br>- sensing 31%<br>- Intuitive 69%<br>- Thinking 20%<br>- Feeling 80%<br>- Judging 69%<br>- Perceptive 31%	85.71%

**Heydari (2016) Is Nurses' Professional Competence Related to Their Personality and Emotional Intelligence? A Cross-Sectional Study**	Nurses (n=173)<br>Males (n=47)<br>Females (n=157)	Emotional Intelligence	Schutte Self-Report Emotional Intelligence Test (SSEIT-33)	Country:<br>Iran<br>Method of Collection:<br>Nurses at 11 teaching hospitals, paper form.<br>Analysis:<br>Descriptive statistics, Spearman's rank correlation coefficient, multiple linear regression	Nursed global EI mean (SD) 98.18 (9.277).	100.00%
**Hirai (2020) Emotional intelligence and work perceptions among nurse managers**	Nurse Managers (n=105)<br>Males (n=6)<br>Females (n=99)	Emotional Intelligence	Emotional Intelligence Scale (EQS)	Country:<br>Japan<br>Method of Collection:<br>Participating nurse managers at 30 hospitals between Dec 2018 - Jan 2019 in Chugoku-Shikoku region, receive a packet with questionnaire inside.<br>Analysis:<br>descriptive statistics, ANOVA, Kolmogorov-Smirnov test, t-tests, Pearson's correlation coefficients	Nurses global EI mean (SD) for 223.29 (1.3) (higher than adult average), and by subdomain:<br>- intrapersonal 46.08 (11.16) - higher than adult average<br>- interpersonal 44.17 (11.45) - higher than adult average<br>- situational 42.79 (12.64) - higher than adult average.	85.71%

**Hojat (1999) A comparison of the personality profiles of internal medicine residents, physician role models, and the general population**	Residents (n=292)<br>Males (n=234)<br>Females (n=58)<br><br>Residents (n=104)<br>Doctors (n=188)	Personality	NEO Personality Inventory Revised (NEO-PI-R)	Country:<br>USA<br>Method of Collection:<br>Survey distributed by hand to participants.<br>Analysis:<br>descriptive statistics, t-tests, Duncan post-hoc comparison tests	Resident personality traits mean (SD) by factor:<br>- extraversion 116.3 (20.8)<br>- openness 120.2 (17.6)<br>- agreeableness 121.4(16.9)<br>- conscientiousness 127.8 (18.8)<br>- neuroticism 83.8 (24.9)<br><br>Physician personality traits mean (SD) by factor:<br>- extraversion 121.0 (19.5)<br>- openness 120.3 (19.8)<br>- agreeableness 132.2 (16.6)<br>- conscientiousness 135.0 (17.5)<br>- neuroticism 66.2 (19.4)	85.71%
**Holliday (2017) Emotional Intelligence and Burnout in Academic Radiation Oncology Chairs**	Radiation Oncology Doctors (n=60)<br>Males (n=51)<br>Females (n=9)	Emotional Intelligence	Trait Emotional Intelligence Questionnaire-Short Form (TEIQue-SF)	Country:<br>USA<br>Method of Collection:<br>Doctors of academic radiation oncology department chairs, email sent to identified participants, survey completed through REDCap.<br>Analysis:<br>descriptive statistics, Pearson's correlation coefficient, Wilcoxon Rank Sum Test	Doctors global EI median (IQR) of 172 (155-182) of possible score of 210; subscale scores:<br>- well-being 37 (35-39) possible score of 42<br>- self-control 31.5 (17-39) possible score of 42<br>- emotionality 45 (40-49) possible score of 56<br>- sociability 32 (29-35) possible score of 42	85.71%

**Hollis (2017) Emotional intelligence in surgery is associated with resident job satisfaction**	General Surgery Residents (n=31)<br>Males (n=20)<br>Females (n=11)	Emotional Intelligence	Trait Emotional Intelligence Questionnaire (TEIQue)	<u>Country:</u><br>USA<br><u>Method of Collection:</u><br>General surgical residents at single institution completed online via Qualtrics in June 2015.<br><u>Analysis:</u><br>Descriptive statistics, t-tests, analysis of variance.	Resident global EI mean (SD) by gender:<br>- Female 5.4 (0.5)<br>- Male 5.1 (0.5)	100.00%
**Hong (2016) The mediating effect of emotional intelligence between emotional labour, job stress, burnout and nurses' turnover intention.**	Nurses (n=211)<br>Males (n=unknown)<br>Females (n=unknown)	Emotional Intelligence	Wong and Law Emotional Intelligence (WLEIS)	<u>Country:</u><br>South Korea<br><u>Method of Collection:</u><br>Nurses working in university hospital in G province for greater than 1 year. Questionnaires were collected in August 2014.<br><u>Analysis:</u><br>Descriptive statistics and correlations between measured variables.	Nursing EI mean (SD) total of 3.4 (0.47) and score by subscale:<br>- self emotion appraisal 3.5 (0.53)<br>- other's emotion appraisal 3.5 (0.52)<br>- regulation of emotion 3.4 (0.55)<br>- use of emotion 3.3 (0.58)<br><br>Emotional intelligence is negatively correlated with emotional labour and reduces job stress, burnout and turnover intention.<br>Emotional intelligence has decreasing effect on turnover intention through burnout, although its direct effect on	71.43%

					turnover intention is not significant.<br>Emotional intelligence has mediation effect between emotional labour and burnout.	
**Hosseini (2022) Personality and occupational correlates of anxiety and depression in nurses: the contribution of role conflict, core self-evaluations, negative affect and bullying**	Nurses (n=329)<br>Males (n=41)<br>Females (n=288)	Personality	Core Self-Evaluations Scale (CSES)	Country:<br>Iran<br>Method of Collection:<br>Nurses in hospitals of Bandar Abbas, Iran between Aug-Nov 2019<br>Analysis:<br>descriptive statistics, t-tests, ANOVA, Pearson's correlation coefficient, multiple regression analysis	Nurses personality mean (SD) score of 42.12 (5.42).	71.43%

**Hu (2021) Relationship Between Proactive Personality and Job Performance of Chinese Nurses: The Mediating Role of Competency and Work Engagement**	Nurses (n=246)<br>Males (n=0)<br>Females (n=246)	Personality	Proactive Personality Scale (PPS)	Country:<br>China<br>Method of Collection:<br>Nurses in large third-degree hospital in Xián, PR, China in Oct 2019, survey was distributed by surveyors.<br>Analysis:<br>Descriptive statistics, Pearson's correlations.	Nurses proactive personality mean (SD) of 5.461 (0.873).<br><br>Proactive personality significantly and positively associated with competency, work engagement, and job performance.	71.43%
**Huang (2021) Personality Profiles and Personal Factors Associated with Psychological Distress in Chinese Nurses**	Nurses (n=953)<br>Males (n=19)<br>Females (n=934)	Personality	Ten-Item Personality Inventory - Chinese (TIPI-C)	Country:<br>China<br>Method of Collection:<br>Nurses in Guangzhou, Guangdong Province in Nov 2020. Snowballing effect with survey completed through Questionnaire Star Platform.<br>Analysis:<br>descriptive statistics, z-scores, AIC, BIC, adjusted-LRT, BLRT.	Nurses prevalence (N) of personability trait by psychological distress and category:<br><br>No-mild psychological distress<br>- Negative 49.7% (143)<br>- Normative 79.9% (230)<br>- Positive 94.7% (54)<br><br>Mod-Severe psychological distress<br>- Negative 50.3% (306)<br>- Normative 20.1% (58)<br>- Positive 5.3% (3)	100.00%

**Hudek-Knezević (2011) Personality, organizational stress, and attitudes toward work as prospective predictors of professional burnout in hospital nurses.**	Nurses (n=118)<br>Males (n=0)<br>Females (n=118)	Personality	Big Five Inventory (BFI)	Country:<br>Croatia<br>Method of Collection:<br>Female nurses from 19 wards of Clinical Hospital in Reijeka in fall of 2014, recruited by ward managers.<br>Analysis:<br>Hierarchical regression analysis.	Nursing personality mean (SD) by subscales:<br>- extraversion 28.29 (4.35)<br>- agreeableness 35.36 (4.54)<br>- conscientiousness 37.30 (4.02)<br>- neuroticism 20.29 (5.19)<br>- openness 35.43 (5.03)<br><br>Personality traits are significant but weak prospective predictors of burnout and as a group predicted only reduced professional efficacy. Agreeableness single negative predictor.	85.71%
**Hwang (2022) Developing a structural equation model from Grandey's emotional regulation model to measure nurses' emotional labour, job satisfaction, and job performance**	Clinical Nurses (n=424)<br>Males (n=27)<br>Females (n=397)	Emotional Intelligence	Wong and Law Emotional Intelligence Scale (WLEIS) - 7 point scale	Country:<br>South Korea<br>Method of Collection:<br>Survey distributed to seven general hospitals in Gwangju metropolitan city and Jeollanam-do, participants randomly selected between Feb-March 2018<br>Analysis:<br>descriptive statistics, Cronbach's, Pearson's correlation coefficients	Nurses global EI mean (SD) 4.84 (0.720).	85.71%

**Iorga (2016) The burnout syndrome of forensic pathologists. The influences of personality traits, job satisfaction and environmental factors**	Forensic Pathologist (n=37)<br>Males (n=17)<br>Females (n=20)	Personality	Big Five Inventory (BFI)	<u>Country:</u><br>Romania<br><u>Method of Collection:</u><br>Surveys self-administered and then asked to return within the week.<br><u>Analysis:</u><br>descriptive statistics, t-tests, spearman's correlations, multiple linear regressions	Pathologist personality mean (SD) by trait:<br>- extraversion 3.98 (0.73)<br>- agreeableness 3.97 (1.09)<br>- conscientiousness 3.88 (0.65)<br>- neuroticism 2.68 (0.76)<br>- openness 3.77 (0.59)	85.71%
**Iorga (2017) Factors Influencing Burnout Syndrome in Obstetrics and Gynaecology Physicians**	Gynaecology Doctors (n=116)<br>Males (n=35)<br>Females (n=81)	Personality	Big Five Inventory (BFI)	<u>Country:</u><br>Romania<br><u>Method of Collection:</u><br>Self-administered questionnaires, distributed and returned to investigators.<br><u>Analysis:</u><br>Descriptive statistics, t-tests, Pearson correlations	Doctors personality mean (SD) by trait:<br>- extraversion 3.71 (0.71)<br>- neuroticism 2.79 (0.710<br>- openness 3.60 (0.570<br>- Conscientiousness 3.99 (0.59)<br>- agreeableness 3.85 (0.83)<br><br>Higher total scores for a neuroticism, and lower scores for extraversion, conscientiousness, and openness.	85.71%

**Iorga (2020) Study on professionals working in palliative care and oncology departments: The relationship between personality factors, professional events and burnout syndrome**	Oncology-Palliative Healthcare Professionals (n=65)<br>Males (n=18)<br>Females (n=47)<br><br>Doctors (n=17)<br>Nurses (n=34)<br>Other healthcare staff (n=14)	Personality	Big Five Inventory (BFI)	<u>Country:</u><br>Romania<br><u>Method of Collection:</u><br>Printed questionnaire distributed to doctors, nurses and healthcare staff.<br><u>Analysis:</u><br>Descriptive statistics, ANOVA's, Pearson correlations	Oncology-palliative healthcare professionals personality mean (SD) by trait:<br>- extraversion 31.12 (5.07)<br>- agreeableness 37.42 (5.46)<br>- consciousness 37.10 (5.48)<br>- neuroticism 19.30 (6.35)	85.71%
**IsakssonRo (2010) A three-year cohort study of the relationships between coping, job stress and burnout after a counselling intervention for help-seeking physicians**	Doctors (n=184)<br>Males (n=83)<br>Females (n=101)	Personality	Eysenck Personality Questionnaire	<u>Country:</u><br>Norway<br><u>Method of Collection:</u><br>Baseline survey completed prior to intervention. Survey was mailed to participants.<br><u>Analysis:</u><br>descriptive statistics, ANOVA	Doctors baseline neuroticism mean (SD) 2.6 (1.8) (score range from 1-6, where higher score = increase neuroticism).	100.00%

**Issa (2022) The Relationship between Emotional Intelligence and Pain Management Awareness among Nurses**	Nurses (n=330)<br>Males (n=99)<br>Females (n=231)	Emotional Intelligence	Wong and Law Emotional Intelligence Scale (WLEIS) - 5 point scale	<u>Country:</u><br>Saudi Arabia<br><u>Method of Collection:</u><br>Unknown.<br><u>Analysis:</u><br>Descriptive statistics, person's correlations	Nurse EI mean (SD) by subscale:<br>- self-emotional appraisal 3.651 (0.763)<br>- others emotional appraisal 3.63 (0.777)<br>- use of emotion 3.701 (0.87)<br>- regulation of emotion 3.585 (0.826)	85.71%
**Jacobs (2016) Mindfulness facets, trait emotional intelligence, emotional distress, and multiple health behaviours: A serial two-mediator model**	Occupational Therapists (n=427)<br>Males (n=22)<br>Females (n=405)	Emotional Intelligence	Trait Emotional Intelligence Questionnaire-Short Form (TEIQue-SF)	<u>Country:</u><br>Germany<br><u>Method of Collection:</u><br>Online survey on three online forums for German Occupational therapists.<br><u>Analysis:</u><br>Ordinary lease squares path analytical framework.	Occupational Therapist mean (SD) EI of 5.30 (0.67).	85.71%

**Jacoby (2022) Do incoming residents vary in measures of emotional status even prior to residency training?**	Residents (n=229)<br>Males (n=108)<br>Females (n=121)	Emotional Intelligence	Emotional and Social Competency Inventory (ESCI)	Country:<br>USA<br>Method of Collection:<br>Survey distributed via online Qualtrics platform as they entered their resident program, between 2015 - 2017.<br>Analysis:<br>Descriptive statistics, Kruskal-Wallis test, Chi-square or Fisher's Exact test, t-tests, Mann-Whitney U test	Resident EI Mean (SD/IQR) by subscale:<br>- achievement orientation 4.5 (4.2-4.8)<br>- adaptability 4.0 (3.8-4.5)<br>- conflict management 3.9 (0.6)<br>- coach and mentor 4.0 (3.7-4.3)<br>- empathy 4.2 (3.8-4.5)<br>- emotional self-awareness 4.0 (3.7-4.5)<br>- emotional self-control 4.2 (3.8-4.7)<br>- inspirational leadership 3.7 (0.5)<br>- influence 3.8 (3.5-4.3)<br>- organisational awareness 4.2 (3.8-4.5)<br>- positive outlook 4.0 (3.8-4.8)<br>- teamwork 3.0 (2.8-3.2)	100.00%

**Joffe (2022) Doctors in distress: The personality profile of derailing doctors**	Doctors (n=434)<br>Males (n=unknown)<br>Females (n=unknown)<br><br>Derailing (n=77)<br>Control (n=357)	Personality	NEO Personality Inventory Revised (NEO-PI-R)	<u>Country:</u><br>UK<br><u>Method of Collection:</u><br>Survey completed online over multiple years.<br><u>Analysis:</u><br>Descriptive statistics, A MANCOVA,	Doctors personality mean (SD) by derailing / non-derailing and trait:<br><br><u>Derailing</u> Doctors<br>- neuroticism 79.36 (20.60)<br>- extraversion 113.82 (17.82)<br>- openness 114.53 (20.09)<br>- agreeableness 125.55 (17.25)<br>- conscientiousness 129.90 (19.36)<br><br><u>Non-Derailing</u> Doctors<br>- neuroticism 59.37 (18.50)<br>- extraversion 126.62 (17.00)<br>- openness 125.02 (15.11)<br>- agreeableness 138.31 (14.60)<br>- conscientiousness 144.80 (16.51)<br><br>Doctors are more Neurotic (less resilient), and less Agreeable, Conscientious, Extraverted and Openness.	85.71%

**Jones (2010) Nurse-to-nurse hostility, confrontational anxiety, and emotional intelligence: an integral, descriptive pilot study.**	Acute Care Nurses (n=33)<br>Males (n=unknown)<br>Females (n=unknown)	Emotional Intelligence	Wong and Law Emotional Intelligence Scale (WLEIS)	<u>Country:</u><br>USA<br><u>Method of Collection:</u><br>Surveys collected between October 2007 - June 2008.<br><u>Analysis:</u><br>t-tests	Nurses with <u>confrontational anxiety scores (CAS) low ability</u> mean EI score of 3.63 and sore by subscale:<br>- self emotions appraisal 3.13<br>- others emotions appraisal 3.81<br>- use of emotion 3.56<br>- regulation of emotion 4<br><br>Nurses with <u>confrontational anxiety scores (CAS) high ability</u> mean EI score of 3.41 and sore by subscale:<br>- self emotions appraisal 3.88<br>- others emotions appraisal 3.24<br>- use of emotion 3.47<br>- regulation of emotion 3.06<br><br>Competent EI improves control over negative emotions and interactions with others.	42.86%

**Jones (2012) Is personality the missing link in understanding recruitment and retention of rural general practitioners?**	General Practitioners (n=472)<br><br>Rural practitioners (case) (n=372)<br>Urban practitioners (control) (n=100)	Personality	NEO Five-Factor Inventory	<u>Country:</u><br>Australia<br><u>Method of Collection:</u><br>Case-control sample for rural and urban doctors recruited by Rural doctors network and Sydney division of general practice.<br><u>Analysis:</u><br>Unconditional logistic regression comparing urban and rural.	<u>GP Total</u> Personality mean (SD) by subscale:<br>- openness 25.2 (6.1)<br>- conscientiousness 33.3 (6.6)<br>- extraversion 28.1 (6.2)<br>- agreeableness 33.4 (5.6)<br>- neuroticism 16.6 (7.6)<br><br><u>Rural GP</u> Total Personality mean (SD) by subscale:<br>- openness 24.2 (5.5)<br>- conscientiousness 33.8 (6.3)<br>- extraversion 28.3 (6.1)<br>- agreeableness 33.5 (5.5)<br>- neuroticism 16.7 (7.7)<br><br><u>Urban GP</u> Total Personality mean (SD) by subscale:<br>- openness 28.2 (6.9)<br>- conscientiousness 31.7 (7.4)<br>- extraversion 27.3 (6.6)<br>- agreeableness 33.0 (6.0)<br>- neuroticism 16.4 (7.2)<br><br>Rural GPs scored, on average, more highly than urban GPs with respect to conscientiousness and agreeableness but lower on openness.	71.43%

**Judkins (2006) Hardiness, stress, and use of ill-time among nurse managers: Is there a connection?**	Nurse Managers (n=15)<br>Males (n=4)<br>Females (n=11)	Personality	Hardiness Scale (HS)	<u>Country:</u><br>USA<br><u>Method of Collection:</u><br>Convenient sample from Texas Hospital, nil details of survey distribution method.<br><u>Analysis:</u><br>Descriptive statistics	Nurse managers hardiness mean (SD) of 96.1 (8.3), where max score is 135.	85.71%
**Jummi (2019) Emotional Intelligence, Emotional Labour and Exhaustion of the Clinical Nurses**	Clinical Nurses (n=215)<br>Males (n=<br>Females (n=	Emotional Intelligence	Wong and Law Emotional Intelligence Scale (WLEIS) - 5 point scale	<u>Country:</u><br>Korea<br><u>Method of Collection:</u><br>Hospital nurses working between May-Dec 2013,<br><u>Analysis:</u><br>Descriptive statistics, t-tests, one way ANVOA, Pearson's correlations	Nurses global EI mean (SD) of 3.66 (0.63) and by subscale:<br>- self-emotional appraisal 3.55 (0.67)<br>- others emotional appraisal 3.40 (0.64)<br>- Emotional control 3.15 (0.68)<br>- emotional application 3.30 (0.51)<br><br>Emotional intelligence of clinical nurses reduce emotional labour and emotional labour increased the exhaustion.	71.43%

**Ju-Young (2019) Influence of Perceptions of Death, End-of-Life Care Stress, and Emotional Intelligence on Attitudes towards End-of-Life Care among Nurses in the Neonatal Intensive Care Unit**	Neonatal Intensive Care Nurses (n=111)<br>Males (n=<br>Females (n=	Emotional Intelligence	Wong and Law Emotional Intelligence Scale (WLEIS) - 7 point scale	<u>Country:</u><br>Korea<br><u>Method of Collection:</u><br>Nurses from 3 university hospitals, 2 general hospitals, with at least 1 month of work. Survey distributed via email.<br><u>Analysis:</u><br>Descriptive statistics, t-tests, one way ANOVA, Scheffe test, Pearson's correlation coefficient	Nurses EI mean (SD) of 4.66 (0.70) out of 7 (where higher scores = higher EI) and subscale:<br>- self-emotional appraisal 4.89 (0.81)<br>- others emotional appraisal 4.86 (0.77)<br>- Regulation of emotion 4.63 (0.78)<br>- use of emotion 4.24 (0.87)	100.00%
**Kahraman (2016) Identifying emotional intelligence skills of Turkish clinical nurses according to sociodemographic and professional variables.**	Clinical Nurses (n=312)<br>Males (n=25)<br>Females (n=287)	Emotional Intelligence	Bar-On's Emotional Quotient Inventory (EQ-I) - 87 items	<u>Country:</u><br>Turkey<br><u>Method of Collection:</u><br>Nurses from 36 hospitals in metropolitan Ankara, data collected between Jun - Aug 2012.<br><u>Analysis:</u><br>Descriptive statistics, t-tests, Mann-Whitney U test, Kruskal-Wallis H test, ANOVA.	Nurse leaders EI mean (SD) 332.3 (31.3) and by subscale:<br>- intrapersonal 111.74 (12.72)<br>- interpersonal 75.02 (6.49)<br>- adaptability 55.28 (6.33)<br>- stress management 43.14 (7.13)<br>- general mood 47.14 (5.99)<br><br>No significant differences between emotional intelligence scores of the nurses according to demographic variables. Higher EI scores observed in staff who had >10 years' experience, successful in professional life, previously	100.00%

					received self-improvement training.	
**Karimi (2014) Emotional rescue: the role of emotional intelligence and emotional labour on well-being and job-stress among community nurses.**	Community Nurses (n=312)<br>Males (n=31)<br>Females (n=290)	Emotional Intelligence	Self-Report Emotional Intelligence Test (SREIT)	Country:<br>Australia<br>Method of Collection:<br>Self-reported questionnaire was mailed to all eligible Victorian community nurses during 2010.<br>Analysis:<br>SEM analysis to look at EI and emotional labour and the effects on job stress and well-being.	Community nurses EI mean (SD) of 3.73 (0.36)<br><br>Higher level of EI associated with higher level of well-being.	100.00%

**Kaur (2013) Effect of spiritual intelligence, emotional intelligence, psychological ownership and burnout on caring behaviour of nurses: a cross-sectional study**	Nurses (n=550)<br>Males (n=10)<br>Females (n=438)	Emotional Intelligence	Schutte's Emotional Intelligence Test (SSEIT)	<u>Country:</u><br>Malaysia<br><u>Method of Collection:</u><br>Random selection of nurses from 7 public hospitals.<br><u>Analysis:</u><br>Descriptive statistics, correlation between dimensions.	Nurse EI mean (SD) of 3.85 (0.06)	85.71%
**Kaur (2015) Impact of emotional intelligence and spiritual intelligence on the caring behaviour of nurses: a dimension-level exploratory study among public hospitals in Malaysia.**	Nurses (n=448)<br>Males (2%)<br>Females (98%)	Emotional intelligence	Schutte Self-Report Emotional Intelligence Test (SSEIT)	<u>Country:</u><br>Malaysia<br><u>Method of Collection:</u><br>Random sample of nurses at 7 major public hospitals in Kuala Lumpur. Questionnaires distributed through head nurses.<br><u>Analysis:</u><br>Descriptive statistics, correlation between dimensions.	Nurses EI mean (SD) by subscale:<br>- perception of emotion 3.70 (0.38)<br>- managing own emotions 4.09 (0.37)<br>- managing others 'emotion 3.73 (0.39)<br>- utilising emotions 3.88 (0.43)<br><br>Each dimension of EI has a cascading effect on other dimensions of EI with perception of emotion being the main driver.	71.43%

**Kaya (2018) The Relationship Between Nurses' Sociotropy-Autonomy Personality Characteristics and Trait Anger: Anger Expression Styles**	Nurses (n=370)<br>Males (n=6)<br>Females (n=364)	Personality	Trait Anger–Anger Expression Scales<br><br>Sociotropy–autonomy Scale (SAS)	<u>Country:</u><br>Turkey<br><u>Method of Collection:</u><br>Random stratified sampling of nurses at university hospital.<br><u>Analysis:</u><br>Cronbach's alpha analysis, Descriptive statistics, Pearson's correlations.	Nurses Sociotrophy-Autonomy scale mean (SD) by subscale:<br>Sociotropy 66.71(16.15)<br>- Concern About Disapproval 17.76 (6.92)<br>- Attachment/Concern About Separation 33.24 (7.73)<br>- Pleasing Others 15.70 (4.19)<br>Autonomy 75.41 (15.33)<br>- Individualistic or Autonomous Achievement 32.38 (6.78)<br>- Mobility/Freedom From Control by Others 29.58 (6.74)<br>- Preference for Solitude 13.45 (4.77)<br><br>Nurses Trait Anger-Anger Expression scale mean (SD) by subscale:<br>- Trait Anger 20.58 (4.46)<br>- Anger-In 16.24 (3.48)<br>- Anger-Out 15.38 (3.13)<br>- Anger Control 22.86 (4.27)<br><br>The higher the sociotropy–autonomy personality characteristics of the nurses are in an investigation, the higher the Trait Anger, Anger-In, and Anger-Out scores are.	85.71%

**Kennedy (2014) The personality of emergency nurses: is it unique?**	Emergency Nurses (n=72)<br>Males (19.4%)<br>Females (80.6%)	Personality	NEO-PI-3	Country:<br>Australia<br>Method of Collection:<br>Permanent emergency nurses in 1 Sydney ED between July - October 2012 surveyed via nurse educators.<br>Analysis:<br>Descriptive statistics, t-tests.	ED Nurses mean (SD) personality by subcales:<br>- Extraversion 121.8 (8)<br>- Openness to experience 119.9 (19.2)<br>- Agreeableness 125.9 (17.5)<br><br>Scored higher than population norms:<br>- 4/6 within the Extraversion domain: warmth; activity; excitement seeking and; positive emotions.<br>- 3/6 within openness to experience domain: feelings; actions and; values.<br>- 2/6 within agreeableness domain: altruism and modesty.<br>Nil difference in domains of neuroticism and conscientiousness.<br><br>Personality profile of this sample of emergency nurses is different to the population norm.	100.00%

**Keogh (2019) Assessing Behavioural Styles Among Nurse Managers: Implications for Leading Effective Teams.**	Nurse Managers (n=3396) Demographic data not collected.	Behaviour	DiSC	Country:<br>USA<br>Method of Collection:<br>Nurse leaders participating in 73 continuing education workshops throughout USA, collected nurses behaviour styles.<br>Analysis:	Nurse leaders behaviour style frequency, N (%) by DiSC profile:<br>- Dominance 1293 (38%)<br>- Influence 575 (17%)<br>- Steadiness 354 (10%)<br>- Conscientiousness 1174 (35%)<br><br>73% scored highest in Dominance and Conscientiousness.<br>27% scored highest in Influence and Steadiness.	85.71%
**Khanjankhani (2017) Applying artificial neural network approach to predict nurses' job performance based on personality traits and organizational factors**	Nurses (n=89)<br>Males (36%)<br>Females (64%)	Personality	Big Five Personality Traits	Country:<br>Iran<br>Method of Collection:<br>Nurse from inpatient medical departments in attaining hospital (Yazd Providence) with 1 year experience in 2016.<br>Analysis:<br>Descriptive statistics, artificial neural network to extract significance coefficients.	Nursed total personality score mean (SD) of 194.7 (8.6), and subscale score:<br>- Neuroticism 31.8 (4.33)<br>- Extraversion 40.3 (3.09)<br>- Openness to experience 36.5 (3.8)<br>- Agreeableness 41.5 (3.5)<br>- Conscientiousness 44.5 (4.3)<br><br>The most and the least scores were respectively conscientiousness and neuroticism.	71.43%

**Khatri (2012) Physiotherapist's Emotional Quotient and Patient Satisfaction**	Physiotherapists (n=18)<br>Males (n=6)<br>Females (n=12)	Emotional Intelligence	emotional intelligence questionnaire (EIQ)	Country:<br>India<br>Method of Collection:<br>Participants contacted through phone between June-October 2010.<br>Analysis:<br>Descriptive statistics	Physiotherapist mean (SD) of EI of 43.95 (3.961)<br><br>No significant correlation between physiotherapists' emotional intelligence, job satisfaction and patient satisfaction.	42.86%
**Kheirkhah (2018) The Relationship Between Job Stress, Personality Traits and the Emotional Intelligence of Midwives Working in Health Centres of Lorestan University of Medical Sciences in 2017.**	Midwives (n=200)<br>Males (n=unknown)<br>Females (n=unknown)	Emotional Intelligence<br>Personality	Brad berry & Greaves Emotional Intelligence Questionnaire<br>NEO Five-Factor Inventory	Country:<br>Iran<br>Method of Collection:<br>Random selection of midwives working in all cities of the Lorestan province with minimum 6 months working hand delivery questionnaires.<br>Analysis:<br>Descriptive statistics, correlations coefficient test included Pearson's, independent t-test and ANOVA.	Midwives total EI mean (SD) of 111.87 (14.30) and score by subscale:<br>- self-awareness 28.25<br>- self-control / self-management 33.95<br>- social awareness 20.88<br>- Relation management / social skill 35.68<br><br>Midwives total Personality mean (SD) of 135.51 (15.73) and score by subscale:<br>- neuroticism 21.13 (5.45)<br>- extraversion 30.10 (4.29)<br>- openness 27.95 (4.12)<br>- agreeableness 26.64 (4.85)<br>- conscientiousness 29.83 (3.58)<br><br>Negative relationship between intelligence and job stress.	71.43%

					No significant relationship between personality traits and job stress.	
**Khrais (2021) Traits of transformational leaders in nursing: emotional intelligence counts?**	Nurse Managers (n=57)<br>Males (n=22)<br>Females (n=35)	Emotional Intelligence	Trait Emotional Intelligence Questionnaire-Short Form (TEIQue-SF)	<u>Country:</u><br>Jordan<br><u>Method of Collection:</u><br>Nil specific details.<br><u>Analysis:</u><br>Descriptive statistics,	Nurses global EI mean (SD) by gender<br>- Female 162.7 (16.1)<br>- Male 179.4 (18.6)	85.71%

**Kiani (2020) Relationship between personality factors and marital satisfaction among working married doctors: Moderating role of gender**	Doctors (n=100)<br>Males (n=47)<br>Females (n=53)	Personality	Big Five Inventory (BFI) - Sum score	<u>Country:</u><br>Pakistan<br><u>Method of Collection:</u><br>Armed Forces Post Graduate Medical Institute convenient sample between sept -Nov 2018.<br><u>Analysis:</u><br>descriptive statistics, t-tests	Doctors global personality mean (SD) score of 150.52, and by subscale trait and gender:<br>- extroversion: Male 26.94 (4.48) Female 25.81 (4.66)<br>- agreeableness: Male 35.61 (5.51) Female 36.19 (6.29)<br>- Conscientiousness: Male 33.68 (4.89) Female 33.62 (6.33)<br>- Neuroticism: Male 19.13 (4.89) Female 21.13 (4.85)<br>- Openness: Male 35.15 (3.57) Female 35.55 (4.81)<br><br>Gender had significant difference on the subscale of neuroticism.	85.71%
**Kılıç (2022) Relationship Between Nurses' Compassion Level and Emotional Intelligence During the COVID-19 Pandemic: Case of City Hospitals**	Nurses (n=218)<br>Males (n=164)<br>Females (n=54)	Emotional Intelligence	Revised Schutte Emotional Intelligence Scale (RSEIS)	<u>Country:</u><br>China<br><u>Method of Collection:</u><br>Survey collected through google forms from nurses working in city hospitals in the Eastern Anatolia region.<br><u>Analysis:</u><br>Descriptive statistics, Shapiro-Wilk test, Q-Q graphs.	Nursing global EI mean (SD) of 152.1 (14.8) and by subscale:<br>- optimism/mood regulation 78.9 (12.1)<br>- utilisation of emotions 78.9 (12.1)<br>- appraisal of emotions 24.6 (4.2)<br><br>Nurses had a medium–high level of EI during the COVID-19 pandemic	85.71%

**Kim (2016) Relationships between Personal Traits, Emotional Intelligence, Internal Marketing, Service Management, and Customer Orientation in Korean Outpatient Department Nurses.**	OPD Nurses (n=138)<br>Males (n=unknown)<br>Females (n=unknown)	Emotional Intelligence<br>Personality	Big Five Inventory<br>Wong and Law Emotional Intelligence Scale (WLEIS)	Country:<br>Korea<br>Method of Collection:<br>OPD nurses in 4 general hospitals in Seoul. Survey packs delivered by hand and returned by mail.<br>Analysis:<br>Descriptive statistics, relationship between variable using Pearson's correlations coefficient and hierarchical multiple regression.	OPD nurses personality trait mean (SD):<br>- agreeableness 14.44 (2.62)<br>- conscientiousness 20.92 (3.29)<br>- openness 17.46 (3.44)<br>- extraversion 14.54 (2.86)<br>- neuroticism 17.25 (2.82)<br><br>OPD nurses EI mean (SD) of 78.64 (11.87)<br><br>Conscientiousness and emotional intelligence were associated with customer orientation	85.71%
**Kisten (2018) An evaluation of personality traits associated with job satisfaction among South African anaesthetists using the Big Five Inventory**	Anaesthetic Doctors (n=463)<br>Males (n=267)<br>Females (n=196)	Personality	Big Five Inventory (BFI)	Country:<br>South Africa<br>Method of Collection:<br>Electronic questionnaire distributed to all members of African Society of Anaesthesiologists in 2016 via REDCap.<br>Analysis:<br>Nil specific details.	Anaesthetic doctors mean (median, CI) by personality trait:<br>- extraversion 25.78 (26, 25.13-26.42)<br>- agreeableness 36.36 (36,35.85-36.86)<br>- Conscientiousness 39.4 (40,38.98-39.83)<br>- neuroticism 19.38 (19,18.82-19.94)<br>- openness 35.05 (35,34.48)<br><br>Neuroticism is the strongest and most consistent negative	100.00%

					correlate of job satisfaction.<br>Agreeableness was positively associated with job satisfaction.	
**Kooijman (2020) Do therapist effects determine outcome in patients with shoulder pain in a primary care physiotherapy setting?**	Physiotherapists (n=46)<br>Males (n=30)<br>Females (n= 16)	Personality	Big Five Inventory (BFI)	<u>Country:</u><br>Netherlands<br><u>Method of Collection:</u><br>Random sample of physiotherapists registered on national NIVEL primary care database. To be eligible need to be a private practice as a general physiotherapist at least half of their time.<br><u>Analysis:</u><br>Multilevel linear analyses	Physiotherapist personality mean (SD) by subcategories:<br>- Neuroticism 2.40 (0.53)<br>- extraversion 3.52 (0.48)<br>- Agreeableness 3.82 (0.37)<br>- Conscientiousness 3.74 (0.48)<br>- Openness 3.38 (0.47)	85.71%

**Kotus (2021) Personality Traits and the Sense of Self-Efficacy among Nurse Anaesthetists. Multi-Centre Questionnaire Survey**	Anaesthetic Nurses (n=143)<br>Males (n=16)<br>Females (n=127)	Personality	NEO Five-Factor Inventory (NEO FFI)	Country:<br>Poland<br>Method of Collection:<br>Anaesthetic nurses from 5 Poland hospitals,<br>Analysis:<br>descriptive statistics, regression analysis	Nurses personality mean (SD) by trait:<br>- Neuroticism 4.39 (2.0) - low<br>- Extraversion 6.04 (1.76) - high<br>- Openness 5.62 (1.88)<br>- Agreeableness 5.58 (2.1)<br>- Conscientiousness 6.4 (1.93) - high	85.71%
**Kousha (2018) Emotional intelligence and anxiety, stress, and depression in Iranian resident physicians**	Resident Doctors (n=245)<br>Males (n=26)<br>Females (n=74)	Emotional Intelligence	Bar-On's Emotional Quotient Inventory (EQ-I) - 90 item	Country:<br>Iran<br>Method of Collection:<br>Residents if medical sciences between 2014 and 2015.<br>Analysis:<br>descriptive statistics, Kolmogorov-Smirnov test, one-way variance.	Resident global EI mean (SD) of 330.24 (38.5).<br><br>Higher EI is a good predictor of lower stress, anxiety and depression in residents.	85.71%

**Kovach (2010) Do personality traits predict work outcomes of certified nursing assistants?**	Nursing Home Nurse Assistants (n=177)<br>Males (n=13)<br>Females (n=164)	Personality	Hogan Personality Inventory (HPI)	<u>Country:</u><br>USA<br><u>Method of Collection:</u><br>Clinical nurse assistants at 3 nursing homes in Midwest received questionnaires with pay checks.<br><u>Analysis:</u><br>Descriptive statistics, correlational analysis and multiple linear regression.	Nursing assistants personality mean (SD) % high / average / low by trait:<br>- adjustment 22.66 (6.97) 20% high, 22% average, 58% low<br>- ambition 20.58 (5.28) 8% high, 34% average, 58% low<br>- intellectance 11.96 (4.52) 16% high, 29% average, 55% low<br>- likeability 18.63 (3.07) 26% high, 43% average, 31% low<br>- prudence 19.94 (4.83) 43% high, 17% average, 40% low<br>- school success 7.77 (3.14) 32% high, 34% average, 34% low<br>- sociability 11.33 (4.76) 24% high, 34% average, 42% low	71.43%
**Kozlowski (2018) Increasing nurses' emotional intelligence with a brief intervention.**	Nurses (n=39)<br>Males (n=unknown)<br>Females (n=unknown)<br><br>Intervention (n=unknown)<br>Control (n=unknown)	Emotional Intelligence	GENOS Emotional Intelligence Self-Assessment (GENOS EI SA)	<u>Country:</u><br>Australia<br><u>Method of Collection:</u><br>Registered nurses from 8 units, across 2 sites in NSW.<br><u>Analysis:</u>	Nurses total EI score mean (SD) by group:<br>- Intervention 3.77 (0.18)<br>- Control 4.16 (nil SD)<br><br>Control group had higher EI scores, intervention group able to develop EI scores with training, still lower than control. Found that higher EI predicts higher retention.	71.43%

**Krasner (2009) Association of an educational program in mindful communication with burnout, empathy, and attitudes among primary care physicians**	Doctors (n=70)<br>Males (n=38)<br>Females (n=32)	Personality	Mini markers of the Big Five Factor Structure personality scale	<u>Country:</u><br>USA<br><u>Method of Collection:</u><br>Questionnaire completed pre and post intervention.<br><u>Analysis:</u><br>Liner mixed-effect models, descriptive statistics	Doctors personality traits mean (CI) by scale:<br>- extraversion 5.7 (5.4-6.0)<br>- agreeableness 7.3 (7.1-7.6)<br>- conscientiousness 6.5 (6.2-6.7)<br>- emotional stability 6.1 (5.8-6.3)<br>- openness 6.8 (6.6-7.1)	57.14%
**Kutluturkan (2016) Resilience and burnout status among nurses working in oncology.**	Oncology Nurses (n=140)<br>Males (n=unknown)<br>Females (n=unknown)	Personality	Resilience Scale for Adults	<u>Country:</u><br>Turkey<br><u>Method of Collection:</u><br>Experienced nursed in hematology and chemotherapy administration unit and policlinics distributed surveys though department.<br><u>Analysis:</u><br>Data collected analysed by percentage ratios, mean and median values, Kruskal-Wallis test, Mann-Whitney U test, correlation analysis and multistep wise linear regression	Oncology nurses <u>resilience</u> median (IQR) score of 134.00 (122.0;146.0) and the median subscale scores:<br>- structural style 16.00<br>- perception of future 16.00<br>- family cohesion 24.00<br>- self-perception 25.00<br>- social competence 23.00<br>- social resources 31.00<br><br>To increase nurses resilience, nurses should be supported in structured style, perception of future, and perception of self. For less experience of burnout, nurses need to experience less emotional exhaustion and should increase personal accomplishment.	85.71%

**Kwarta (2016) Personality traits and styles of coping with stress in physicians.**	Physicians (n=50)<br>Males (n=25)<br>Females (n=25)	Personality	Five Factor Inventory of Personality (NEO FFI)	<u>Country:</u><br>Poland<br><u>Method of Collection:</u><br>Physicians at Medical university teaching hospital across multiple specialities were eligible.<br><u>Analysis:</u><br>t-tests, Mann-Whitney U test, Pearson's Chi-square test. Correlations via Spearman coefficients.	Physicians mean (SD) of personality for each subscale:<br>- Neuroticism 8.4 (1.2)<br>- Extraversion 9.7 (0.7)<br>- Openness 8.7 (1.1)<br>- Agreeableness 8.3 (1.5)<br>- Conscientiousness 8.6 (1.1)<br>High level of extraversion, other variables similar to one another.<br>Higher levels of extraversion compared to pop norms.<br><br>Break down by gender as per categorised data spreadsheet. Males significantly higher levels of agreeableness.	85.71%

**Kyaw (2022) Personality differences between internal medicine and surgical residents in an Asian population**	Surgical Residents (n=70)<br>Males (n=39)<br>Females (n=31)<br><br>Medical (n=35)<br>Surgical (n=35)	Personality	NEO Personality Inventory Revised (NEO-PI-R)	<u>Country:</u><br>Singapore<br><u>Method of Collection:</u><br>Residents from the National University Hospital System, completed survey at home or private workspace.<br><u>Analysis:</u><br>Descriptive statistics, Cohen's D,	Personality traits mean scores by resident speciality:<br><br><u>Medical</u> Residents<br>- neuroticism 50.0<br>- extraversion 45.4<br>- openness 52.6<br>- agreeableness 47.7<br>- conscientiousness 50.1<br><br><u>Surgical</u> Residents<br>- neuroticism 54.7<br>- extraversion 52.4<br>- openness 49.4<br>- agreeableness 40.5<br>- conscientiousness 51.5<br><br>Medical residents had higher scores in the area of overall Agreeableness	100.00%

**Lalonde (2017) Preceptor characteristics and the socialization outcomes of new graduate nurses during a preceptorship programme**	Nursing Preceptors (n=41)<br>Males (n=3)<br>Females (n=38)	Emotional Intelligence<br>Personality	Nursing Emotional Intelligence Scale (NEIS)<br>International Personality Item Pool (IPIP)	Country:<br>Canada<br>Method of Collection:<br>New graduates and their preceptors at 5 large hospitals in greater Toronto area between 2012-2013 recruited by unit managers and educators. Questionnaires circulated by email.<br>Analysis:<br>Pearson correlational analysis to determine relationships.	Nursing preceptors Personality mean (SD) (range) by trait:<br>- agreeableness 4.36 (0.57) (1.80-5.00)<br>- conscientiousness 4.10 (0.55) (2.30-5.00)<br>- openness 3.63 (0.50) (2.00-4.70)<br>- emotional stability 3.35 (0.65) (2.00-4.70)<br><br>Nursing preceptors EI mean (SD) (range) of 100 (15) (63.6-125.1).<br><br>Preceptor personality traits of openness, conscientiousness and emotional stability were significantly related to new graduate nurses who reported greater turnover intent, job dissatisfaction, role conflict and ambiguity.<br>No significant relationship between preceptor EI and outcome of new graduate nurses.	71.43%

**Langemo (1990) Impact of work stress on female nurse educators.**	Nurse educators (n=287)<br>Males (n=0)<br>Females (n=287)	Personality	Hardiness of Personality Inventory (HPI)	Country:<br>USA<br>Method of Collection:<br>Nurse educators from 18 nursing school programs now working full time and > 1 year experience randomly selected<br>Analysis:<br>Stepwise multiple regression analysis exploring relationships.	Nurse educators hardiness mean (SD) of 77.10 (7.80)<br><br>higher hardiness scores = less emotional exhaustion and depersonalization and more personal accomplishment.	57.14%
**Lartey (2021) Emotional intelligence and perceived organizational support as predictors of emotional exhaustion among nurses and midwives**	Nurses and Midwifes (n=342)	Emotional Intelligence	Schuttle self-report emotional intelligence test (SSEIT)	Country:<br>Ghana<br>Method of Collection:<br>Nursing and midwifery managers contacted and left with survey for staff if they wanted to participate. Data collection occurred over 6 weeks.<br>Analysis:<br>Descriptive statistics, Pearson's r test, hierarchical multiple regressions.	Nurse total EI mean (SD) score of 126.67 (12.83).<br><br>EI was found not to have significant predictive effect on emotional exhaustion.	85.71%

**Laurence (2016) Personality characteristics and attributes of international medical graduates in general practice training: Implications for supporting this valued Australian workforce.**	General Medical Graduates (n=452)<br>Males (n=186)<br>Females (n=266)<br><br>International medical graduates (IMG) (n=102)<br>Males (n=50)<br>Females (n=52)<br><br>Australian medical graduates (AMG) (n=350)<br>Males (n=136)<br>Females (n=214)	Personality	Temperament and Character Inventory (TCI-R140)<br>Resilience Scale	Country:<br>Australia<br>Method of Collection:<br>Survey distributed to ACRRM independent pathway (IP) and Australian General Practice Training (AGPT) rural and general pathways across three states in Australia between 2011 and 2013.<br>Analysis:<br>Univariate analysis of exploring differences in traits between IMG and AMG registrars.	Medical Graduates personality mean (CI) of Novelty seeking:<br>- IMG 2.58 (2.49-2.69)<br>- AMG 2.69 (2.68-2.80)<br>Medical Graduates personality mean (CI) of Harm Avoidance:<br>- IMG 2.71 (2.59-2.90)<br>- AMG 2.71 (2.56-2.75)<br>Medical Graduates personality mean (CI) of Reward Dependence:<br>- IMG 3.42 (3.27-3.52)<br>- AMG 3.49 (3.34-3.49)<br>Medical Graduates personality mean (CI) of Persistence:<br>- IMG 3.47 (3.31-3.56)<br>- AMG 3.59 (3.54-3.69)<br>Medical Graduates personality mean (CI) of Self-Directedness:<br>- IMG 3.76 (3.54-3.80)<br>- AMG 3.85 (3.75-3.90)<br>Medical Graduates personality mean (CI) of Cooperativeness:<br>- IMG 3.83 (3.70-3.91)<br>- AMG 4.07 (3.99-4.12)<br>Medical Graduates personality mean (CI) of Self-Transcendence:<br>- IMG 2.68 (2.51-2.85)<br>- AMG 2.64 (2.60-2.80)<br>Regardless of training program,	85.71%

		IMG and AMG have similarities in personality - with average harm avoidance, high reward dependence, high in self-directedness, and low in self-transcendence.<br>Differences with IMG lover in novelty seeking, lower in self-directedness and lower cooperativeness compared to AMG, likely associated with cultural norms within country of origin.<br><br>Medical Graduates total resilience mean (CI):<br>- IMG 141.3 (136.28-144.69)<br>- AMG 144.5 (143.20-148.23)

**Lawal (2017) The role of emotional intelligence and organisational support on work stress of nurses in Ibadan, Nigeria**	Nurses (n=228)<br>Males (n=41)<br>Females (n=187)	Emotional Intelligence	Wong and Law Emotional Intelligence Scale (WLEIS)	Country:<br>Nigeria<br>Method of Collection:<br>Nurses in University College Hospital in Ibadan non-probability sampling technique. Data collected by research assistants.<br>Analysis:<br>Descriptive statistics, intercorrelations analysis to determine relationships.	Nurses EI mean 74.41 (higher EI) and mean (SD) by subscale:<br>- self emotions appraisal 19.53 (3.29)<br>- others emotions appraisal 18.10 (3.93)<br>- use of emotion 20.77 (3.25)<br>- regulation of emotion 19.82 (3.30)<br><br>Self-emotion appraisal, others' emotional appraisal, use of emotion and regulation of emotion have joint contributions to explaining work stress among nurses. Others' emotion appraisal, use of emotion and perceived organisational support have independent relationships with work stress.	85.71%

**Lee (2021) Analysis of the Relationship between the Psychological Well-Being, Emotional Intelligence, Willpower, and Job-Efficacy of Clinical Nurses: A Structural Model Application**	Clinical Nurses (n=300)<br>Males (n=13)<br>Females (n=287)	Emotional Intelligence	Korean Emotional Intelligence Scale	<u>Country:</u><br>Korea<br><u>Method of Collection:</u><br>Nurses in general hospital in Seoul in May 2020<br><u>Analysis:</u><br>descriptive statistics, Pearson's correlation coefficient, CFA, AGFI, RMSR, AGFI, RFI, IFI, TLI, CFI.	Nurses EI mean (SD) by subscale:<br>- self-emotional appraisal 3.89 (0.54)<br>- others emotional appraisal 3.71 (0.61)<br>- regulation of emotion 3.52 (0.60)<br>- use of emotion 3.14 (0.70)	85.71%
**Lewis (1994) Personality, stress, coping, and sense of coherence among nephrology nurses in dialysis settings**	Nurses (n=49)<br>Males (n=0)<br>Females (n=49)	Personality	Sense of Coherence<br>Myers–Briggs Type Indicator (MBTI)	<u>Country:</u><br>Mexico<br><u>Method of Collection:</u><br>Administrators contacted via phone to request send survey to nurses in unit.<br><u>Analysis:</u><br>descriptive statistics, correlation analysis.	Nurses sense of coherence mean (SD) of 5.13 (0.63) (max score of 7) - higher score indicating stronger sense of coherence.<br><br>Nurses personality trait % and comparison to norm population:<br>- introversion 55% (norm 52.43%)<br>- extroversion 45% (norm 47.57%)<br>- Intuition 51% (norm 43.96%)<br>- Sensing 49% (56.04%)<br>- Thinking 55% (36.22%)<br>- Feeling 45% (63.78%)	85.71%

					- Judging 65% (63.58%)<br>- Perception 35% (36.42%)	
**Li (2014) Exploring the relationship between personality features and teaching self-efficacy in clinical nursing preceptors.**	Clinical Nurse Preceptors (n=116)<br>Males (n=0)<br>Females (n=116)	Personality	Eysenck Personality Questionnaire (EPQ)	<u>Country:</u><br>Taiwan<br><u>Method of Collection:</u><br>Survey of senior RN's working in university affiliated hospital in 2009.<br><u>Analysis:</u><br>Descriptive statistics, multivariate linear model regression determine relationships.	Nurses personality mean (SD) total 24.4 (4.6) and by subscale:<br>- extroversion 5.3 (2.9)<br>- neuroticism 6.0 (3.0)<br>- psychoticism 6.4 (1.4)<br>- lie scale 6.8 (2.0)<br><br>Higher extraversion = higher scores for maturity, teaching self-efficacy beliefs, professional skill, teaching strategy effectiveness, and objective teaching evaluation.<br>Higher neuroticism = negatively associated with maturity.	85.71%

**Lin (2016) Emotional Intelligence as a Predictor of Resident Well-Being**	Residents (n=73)<br>Males (n=42)<br>Females (n=31)	Emotional Intelligence	Trait Emotional Intelligence Questionnaire-Short Form (TEIQue-SF)	<u>Country:</u><br>USA<br><u>Method of Collection:</u><br>Survey completed electronically between April 2013 - May 2014.<br><u>Analysis:</u><br>Descriptive statistics, multivariate linear regression analysis	Resident EI mean (SD) of 5.18 (0.81).<br><br>Emotional intelligence is a strong predictor of resident well-being.	100.00%
**Liu (2018) Mediating role of emotional labour in the association between emotional intelligence and fatigue among Chinese doctors: a cross-sectional study.**	Doctors (n=740)<br>Males (n=299)<br>Females (n=441)	Emotional Intelligence	Won and Law Emotional Intelligence Scale (WLEIS)<br>Emotional Labour	<u>Country:</u><br>China<br><u>Method of Collection:</u><br>Self-administered questionnaire to ten large general hospital doctors between March - April 2014. Questionnaires completed at the end of a shift in a private room.<br><u>Analysis:</u><br>Hierarchical linear regression analysis exploring relationship between EI and emotional about strategies.	Chinese doctors <u>emotional intelligence</u> mean (SD) of 81.29 (14.76)<br><br>Chinese doctors <u>emotional labour</u> mean (SD) subscale:<br>- Surface acting 19.40 (5.91)<br>- Deep acting 12.72 (3.12)<br>- Natural acting 10.60 (2.33)<br><br>EI had a negative association with fatigue.	100.00%

**Looff (2019) Burnout symptoms in forensic mental health nurses: Results from a longitudinal study**	Mental Health Nurses (n= 432)	Emotional Intelligence	Dutch Bar-On Emotional Quotient Inventory	<u>Country:</u><br>Netherlands<br><u>Method of Collection:</u><br>Mental health nurses in forensic psychiatric hospitals, Four waves of data collection over a 2 year period.<br><u>Analysis:</u><br>longitudinal multilevel model	Nurses total EI mean (SD) scores of 106.22 (11.26)<br><br>Nursing staff who experience severe aggression and who have relatively low levels of emotional intelligence.	85.71%
**Lu (2022) Do type A personality and neuroticism moderate the relationships of occupational stressors, job satisfaction and burnout among Chinese older nurses? A cross-sectional survey**	Nurses (n=527)	Personality	Big Five Inventory (BFI)	<u>Country:</u><br>China<br><u>Method of Collection:</u><br>Convenient sample of >40 years old. Survey completed online through WeChat link or paper version. Incentivised recruitment through gift/ lucky money.<br><u>Analysis:</u><br>descriptive statistics, Pearson correlation.	Nurses neuroticism mean (SD) of 2.63 (0.75).	85.71%

**Lv (2018) Team autonomy amplifies the positive effects of proactive personality on work engagement**	Nurses (n=464)<br>Males (3.4%)<br>Females (96.6%)	Personality	Proactive Personality Scale	<u>Country:</u><br>China<br><u>Method of Collection:</u><br>Nurses with 75 teams in 4 hospitals<br><u>Analysis:</u><br>multi-level modelling	Nurses personality mean (SD) of 5.02 (0.78).<br><br>Proactive personality is positively related to work engagement and altruism of employees.<br>- proactive personality and work engagement is stronger with a higher level of team autonomy<br>- proactive personality and altruism is not significantly moderated by degree of team autonomy.	71.43%
**Lydon (2015) Medical speciality choice: does personality matter?**	Doctors (n=200)<br>Males (n=unknown)<br>Females (n=unknown)	Personality	NEO Five-Factor Inventory (NEO FFI) - 5 point scale	<u>Country:</u><br>Ireland<br><u>Method of Collection:</u><br>Doctors were emailed to participate, once consent survey distributed.<br><u>Analysis:</u><br>Descriptive statistics, t-tests, chi-square	Doctors personality mean (SD) by trait:<br>- neuroticism 31.3 (6.9) - average<br>- extraversion 31.3 (6.9) - average<br>- openness 32.3 (7.7) - high<br>- agreeableness 32.6 (6.4) - average<br>- conscientiousness 33.8 (6.5) - average	71.43%

**Lysack (2001) Job choice and personality: a profile of Michigan occupational and physical therapists.**	Occupational Therapists and Physiotherapists (n=294)<br>Males (n=53)<br>Females (n=241)<br><br>Occupational Therapists (n=128)<br><br>Physiotherapists (n=166)	Personality	Keirsey-Bates Personality Inventory	Country:<br>USA<br>Method of Collection:<br>Random sample from both Michigan Occupational Therapy Association (MOTA) and Michigan Physical Therapy Association (MPTA) membership database in 1998 mailed questionnaires.<br>Analysis:<br>Frequency counts and percentages used to compare and summaries OT and PT personality.	Health professional personality temperament percentage in Intuitive-Feeling:<br>- OT 27%<br>- PT 5%<br>- General population 12%<br>Health professional personality temperament percentage in Intuitive-thinking:<br>- OT 19%<br>- PT 11%<br>- General population 12%<br>Health professional personality temperament percentage in Sensing-judging:<br>- OT 5%<br>- PT 66%<br>- General population 38%<br>Health professional personality temperament percentage in Sensing-perceiving:<br>- OT 49%<br>- PT 18%<br>- General population 38%<br>PT more likely to have Sensing-Judging temperament and OT are far more likely to be Sensing-perceiving.<br><br>Health professional personality type percentage of INFP:	85.71%

- OT 4.7%
- PT 1.2%
- Total 2.7%

Health professional personality type percentage of ENFP:

- OT 14.7%
- PT 1.8%
- Total 7.5%

Health professional personality type percentage of INFJ:

- OT 3.1%
- PT 6.0%
- Total 4.7%

Health professional personality type percentage of ENFJ:

- OT 4.7%
- PT 9%
- Total 3.1%

Health professional personality type percentage of ISFP:

- OT 11.6%
- PT 4.2%
- Total 7.5%

Health professional personality type percentage of ESFP:

- OT 14.0%
- PT 9.0%
- Total 11.2%

Health professional personality type percentage of ISFJ:

- OT 0%

- PT 3.6%
- Total 2.0%

Health professional personality type percentage of ESFJ:
- OT 0.8%
- PT 16.9%
- Total 9.8%

Health professional personality type percentage of ENFP:
- OT 3.9%
- PT 0.6%
- Total 2.0%

Health professional personality type percentage of INTP:
- OT 11.6%
- PT 0%
- Total 5.1%

Health professional personality type percentage of INTJ:
- OT 1.6%
- PT 4.2%
- Total 3.1%

Health professional personality type percentage of ENTJ:
- OT 0.8%
- PT 0%
- Total 0.33%

Health professional personality type percentage of ISTP:
- OT 7.8%
- PT 1.8%

			- Total 4.4%<br>Health professional personality type percentage of ESTP:<br>- OT 16.3%<br>- PT 4.2%<br>- Total 9.5%<br>Health professional personality type percentage of ISTJ:<br>- OT 0.8%<br>- PT 12.7%<br>- Total 7.5%<br>Health professional personality type percentage of ESTJ:<br>- OT 3.1%<br>- PT 31.9%<br>- Total 19.3%

**Mackay (2012) A UK-wide analysis of trait emotional intelligence within the radiography profession**	Radiologists (n=1997)<br>Males (n=307)<br>Females (n=1690)<br><br>Diagnostic Radiographer (n= 1537)<br>Angiographer (n=28)<br>Trauma (n=36)<br>Mammographer (n=193)<br>General (n=55)<br>Magnetic Resonance (n=117)<br>Ultra sonographer (n=113)<br>Computerised Tomography (n=61)<br>Nuclear Medicine (n=63)<br><br>Therapeutic Radiographer (n=244)	Emotional Intelligence	Trait Emotional Intelligence (TEI)	Country:<br>United Kingdom<br>Method of Collection:<br>Random sample from list of registered to practice radiography. Recruited using a range of activities, including articles in the national radiography press, conferences, and professional networks. Questionnaire distributed by website and paper.<br>Analysis:	Radiologist Global EI mean (SD) by gender:<br>- Males 5.23 (0.70) / slightly higher than pop norm<br>- Females 5.29 (0.63) / slightly higher than pop norm<br><br>Significant differences in EI found between diagnostic radiographers specialties, not between therapeutic radiographers.<br><br>Diagnostic radiography emotional intelligence global mean (SD):<br>Angiographer 5.60 (0.46)<br>Trauma 5.35 (0.64)<br>MR 5.35 (0.55)<br>General 5.35 (0.52)<br>Mammographer 5.34 (0.58)<br>Ultra sonographer 5.32 (0.62)<br>Computerised Tomography 5.22 (0.85)<br>Nuclear Medicine 5.14 (0.73)<br><br>Diagnostic radiography emotional intelligence mean (SD) for subscale well-being:<br>Angiographer 6.12 (0.68)<br>Trauma 5.89 (0.76)	100.00%

Mammographer 5.86 (0.72)
General 5.85 (0.72)
Magnetic Resonance 5.79 (0.79)
Ultra sonographer 5.72 (0.82)
Computerised Tomography 5.67 (1.09)
Nuclear Medicine 5.46 (1.04)
Diagnostic radiography emotional intelligence mean (SD) for subscale emotionality:
Angiographer 5.62 (0.61)
Mammographer 5.58 (0.74)
Magnetic Resonance 5.49 (0.75)
General 5.45 (0.70)
Ultra sonographer 5.38 (0.83)
Computerised Tomography 5.36 (0.93)
Trauma 5.27 (0.83)
Nuclear Medicine 5.12 (0.90)

**Magee (1998) Personality profiles of male and female positive role models in medicine**	Doctors (n=188)<br>Males (n=164)<br>Females (n= 24)	Personality	Revised NEO Personality Inventory (NEO PI-R)	<u>Country:</u><br>USA<br><u>Method of Collection:</u><br>Direct mail campaign and announcements through CEO of hospitals and nationwide medical institutions.<br><u>Analysis:</u><br>t-tests, Bonferroni's corrections, one-way multivariate analysis, univariate analysis and Buncan post hoc comparison tests.	Doctors personality mean (SD) by each trait:<br>- Extraversion 121 (19.5)<br>- Openness 120.3 (19.8)<br>- Agreeableness 132.2 (16.6)<br>- Conscientiousness 135 (17.5)<br>- Neuroticism 66.2 (19.4)<br><br>Doctors have lower levels of neuroticism compared to population norms, but above level agreeableness, conscientiousness, extraversion and openness compared to population norms.	85.71%
**Mahoney (2020) Turnover, Burnout, and Job Satisfaction of Certified Registered Nurse Anaesthetists in the United States: Role of Job Characteristics and Personality**	Nurse Anaesthetists (n=246)<br>Males (n=98)<br>Females (n=148)	Personality	Ten-Item Personality Inventory (Big Five personality dimensions)	<u>Country:</u><br>USA<br><u>Method of Collection:</u><br>All survey responses were collected by researcher<br><u>Analysis:</u><br>Descriptive statistics, relationships determined via structural equation model statistics.	Nursing Anaesthetists Personality mean (SD) by trait:<br>- extraversion 9.17 (3.08)<br>- Conscientiousness 13.24 (1.22)<br>- Agreeableness 11.46 (2.07)<br>- Stability 11.88 (2.04)<br>- Openness 10.78 (1.92)<br><br>Burnout was negatively associated with agreeableness, stability and openness.	85.71%

**Maillet (2021) Work Environment Characteristics and Emotional Intelligence as Correlates of Nurses' Compassion Satisfaction and Compassion Fatigue: A Cross-Sectional Survey Study**	Nurses (n=1271<br>Males (n=81)<br>Females (n=1180)<br>Not reported (n=10)	Emotional Intelligence	Schutte Self-Report Emotional Intelligence Test (SSEIT)	Country:<br>Canada<br>Method of Collection:<br>RNs invited through regulatory body and vis social media to complete survey online. Data collected between Sept - Dec 2016.<br>Analysis:<br>confirmatory factor analysis, Cronbach's alpha coefficients	Nurse Total EI mean (SD) 124.56 (13.16) (score range 33-165) and by subscale:<br>- perceptions of emotions 37.80(4.88)<br>- managing own emotions 34.26 (4.76)<br>- managing others emotions 29.79 (3.81)<br>- utilisation of emotions 22.66 (3.15)<br><br>emotional intelligence (EI) were significantly correlated with nurses' compassion satisfaction and compassion fatigue, except for two EI subscales	71.43%

**Mao (2021) Promoting resilience and lower stress in nurses and improving inpatient experience through emotional intelligence training in China: A randomized controlled trial**	Nurses (n=103)<br>Males (n=<br>Females (n=<br><br>Intervention (n=43)<br>Control (n=45)	Emotional Intelligence	Wong and Law Emotional Intelligence Scale (WLEIS) - 5 point scale	Country:<br>China<br>Method of Collection:<br>Random selection of nurses from 20 wards in general hospital in Changsha, between Jan 2019 - Jan 2020. Collection of survey via paper format prior to intervention.<br>Analysis:<br>Descriptive statistics, t-tests, chi-square test, Pearson's correlation coefficients, ANOVA, Wald Chi-squared test.	Nurses EI mean (SD) by each subject group and subscale:<br><br>Intervention baseline global EI of 54.67 (1.11)<br>- self-emotional appraisal 15.63 (0.37)<br>- others-emotional appraisal 12.58 (0.44)<br>- use of emotions 13.16 (0.34)<br>- regulation of emotions 13.30 (0.37)<br><br>Control baseline global EI of 58.24 (1.08)<br>- self-emotional appraisal 17.02 (0.36)<br>- others-emotional appraisal 15.40 (0.39)<br>- use of emotions 15.23 (0.38)<br>- regulation of emotions 15.16 (0.40)	85.71%

**Marcisz-Dyla (2022) Personality Traits, Strategies of Coping with Stress and Psychophysical Wellbeing of Surgical and Non-Surgical Doctors in Poland**	Doctors (n=655)<br>Males (n=254)<br>Females (n=401)	Personality Behaviour	Type D Personality Scale (DS-14)<br>Framingham Type A Scale<br>Resiliency Assessment Scale	<u>Country:</u><br>Poland<br><u>Method of Collection:</u><br>Doctors employed at 30 randomly selected hospitals that consented to participate were hand distributed surveys.<br><u>Analysis:</u><br>descriptive statistics, Shapiro-Wilk test, Mann-Whitney U test, logistic regression models.	Doctors prevalence of Type A behaviour is 33.1% and Type B behaviour is 33.8% and remaining 33.1 % intermediate style.<br><br>Doctors personality by Type D mean (SD) is 21.05 (10.58)<br><br>Doctor personality resilience total mean (SD) 75.52 (13.84) for each subscale:<br>- persistence and determination 14.76 (3.28)<br>- openness and sense of humour 15.52 (2.77)<br>- ability to cope and tolerance of negative emotion 14.58 (3.28)<br>- tolerance of failure and viewing life as a challenge 14.82 (3.33)<br>- optimistic and ability to mobilise one's self 13.85 (3.40)	100.00%

**Martinussen (2011) Burnout and engagement among physiotherapists**	Physiotherapists (n=244)<br>Males (n=57)<br>Females (n=186)<br>Not reported (n=1)	Personality	Revised Jenkins Activity Survey	<u>Country:</u><br>Norway<br><u>Method of Collection:</u><br>Questionnaire mailed to physiotherapists from Norwegian Physiotherapy Association anonymously, and reminder sent at 2 weeks.<br><u>Analysis:</u><br>descriptive statistics, regression analysis.	Physiotherapist Type A behaviour mean (AD) by subscale:<br>- Achievement strivings 3.5 (0.44)<br>- Impatience-irritability 2.4 (0.57)	85.71%
**Martos Martinez (2021) Empathy, Affect and Personality as Predictors of Engagement in Nursing Professionals**	Nurses (n= 1268)<br>Males (n=<br>Females (n=	Personality	Big Five Inventory (BFI-10)	<u>Country:</u><br>Spain<br><u>Method of Collection:</u><br>Computer-aided web interviewing survey platform via snowballing sampling through social media platforms.<br><u>Analysis:</u><br>descriptive statistics, independent t-tests, Cohen's d	Nursing Personality mean (SD) by factor:<br>- Extraversion 3.50 (0.80)<br>- Agreeableness 4.01 (0.58)<br>- Conscientiousness 3.87 (0.67)<br>- Neuroticism 2.67 (0.81)<br>- Openness to experience 3.51 (0.74)<br><br>Positive correlation between cognition empathy and agreeableness, conscientiousness and open to experience.<br>Affective empathy positive correlation with neuroticism.	85.71%

**Masmouei (2020) Relationship Between Personality Traits and Nursing Professionalism**	Nurses (n=1015)<br>Males (n=unknown)<br>Females (n=unknown)	Personality	NEO Personality Inventory	<u>Country:</u><br>Iran<br><u>Method of Collection:</u><br>Nurses working in teaching hospitals in Khorramabad City.<br><u>Analysis:</u><br>Kolmogorov-Smirnov test, Pearson correlations, descriptive statistics.	Nurses personality mean (SD) by trait for each of the scoring levels.<br><br><u>Low</u> levels<br>- neuroticism 20.64 (6.12)<br>- openness 24 (0)<br><br><u>Medium</u> levels<br>- neuroticism 35.20 (5.881)<br>- extraversion 41.0 (4.369)<br>- openness 35.5 (4.264)<br>- agreeableness 40.14 (5.115)<br>- conscientiousness 42.69 (3.779)<br><br><u>High</u> levels<br>- neuroticism 50.33 (1.528)<br>- extraversion 50.96 (2.010)<br>- openness 50.50 (2.121)<br>- agreeableness 51.68 (2.317)<br>- conscientiousness 53.05 (3.132)<br><br>Big Five personality traits strong relationship to nursing professionalism;<br>with conscientiousness and extraversion as the most pronounced traits.	85.71%

**Matthews (1980) Pattern A, achievement striving, and scientific merit: does pattern A help or hinder?**	Psychologists (n= 118)<br>Males (n=118)	Behaviour	Jenkins Activity Survey	Country:<br>USA<br>Method of Collection:<br>Members from Society of Experimental Social Psychology (SESP) were sent to all male members in 1975.<br>Analysis:<br>Not specified.	Male psychology scientists type A behaviour mean (SD) of 10.3 (3.4) - shown to be somewhat higher than the means scores of student groups.	57.14%
**MazzellaEbstein (2019) The relationships between coping, occupational stress, and emotional intelligence in newly hired oncology nurses**	Oncology Nurses (n=114)<br>Males (n=5)<br>Females (n=108)<br>Unknown (n=1)	Emotional Intelligence	Bar-On's Emotional Quotient Inventory 2.0 (EQ-I 2.0)	Country:<br>USA<br>Method of Collection:<br>Newly hired nurses completed survey between 8th and 12th weeks post hire.<br>Analysis:<br>Descriptive statistics, bivariate statistical analysis, regression models.	Nurses EI mean (SD) of 105.24 (13.02)	100.00%
**Mazzella-Ebstein (2021) The Emotional Intelligence, Occupational Stress, and Coping Characteristics by Years of Nursing Experiences of**	Oncology Nurses (n=114)<br>Males (n=5)<br>Females (n=108)<br><br>New grad (n=60)<br>1-5 years (n=37)<br>>5 years (n=17)	Emotional Intelligence	Bar-On's Emotional Quotient Inventory 2.0 (EQ-I 2.0)	Country:<br>USA<br>Method of Collection:<br>Secondary analysis of data collected from newly hired nurses in previous study.<br>Analysis:<br>Descriptive statistics, ANOVA, Tukey honest post hoc	Nurse EI mean (SD) by years of experience:<br>- new graduate 104.83 (12.53)<br>- 1-5 years 104.41 (12.62)<br>- >5 years 108.47 (15.70)	100.00%

**Newly Hired Oncology Nurses**						
**McCranie (1987) Work stress, hardiness, and burnout among hospital staff nurses**	Nurses (n=107)<br>Males (n=5)<br>Females (n=102)	Personality	Abridged Hardiness Scale	<u>Country:</u><br>USA<br><u>Method of Collection:</u><br>Nil details re: method of delivery<br><u>Analysis:</u><br>Descriptive statistics, hierarchical regression	Higher the score, the less the individual is characterised by commitment, control and challenge.	85.71%
**McCranie (1989) Personality antecedents of burnout among middle-aged physicians**	Doctors (n=440)<br>Males (n=unknown)<br>Females (n=unknown)	Personality	Minnesota Multiphasic Personality Inventory (MMPI)	<u>Country:</u><br>USA<br><u>Method of Collection:</u><br>Alumni rom 8 classes of Medical College of Georgia between years 1953-1961, were mailed survey, with two follow up mailings.<br><u>Analysis:</u><br>descriptive statistics, t-scores,	Doctors personality traits mean (SD) by scale:<br>- neuroticism 11.59 (7.76)<br>- psychoticism / infrequency 3.88 (2.67)<br>- masculinity v femininity 29.54 (3.36)<br>- extraversion 16.85 (3.54)<br>- religious orthodoxy 10.94 (4.39)<br>- somatic complaints 2.02 (1.95)	85.71%

					- inadequacy 4.55 (3.50)<br>- cynicism 9.17 (4.89)<br>- intellectual interest 8.67 (1.45)	
**McCulloch (2005) Tolerance of uncertainty, extroversion, neuroticism and attitudes to randomized controlled trials among surgeons and physicians**	Consultant Surgeons and Physicians (n=724)<br><br><u>General Surgeons</u> (n=346)<br>Sex ratio (M:F) 92:8<br><br><u>Physicians</u> (n=378)<br>Sex ratio (M:F) 79:21	Personality	Eysenck personality dimensions	<u>Country:</u><br>United Kingdom<br><u>Method of Collection:</u><br>Random selection of 100 members from royal college of physicians (London) and association of surgeons of Great Britain and Ireland.<br><u>Analysis:</u><br>Descriptive statistics, Mann-Whitney U, t-tests, Pearson's correlations and multiple linear regressions.	Personality of Physicians mean (SD) by subscale:<br>- Extroversion 4.06 (1.48)<br>- Neuroticism 3.06 (1.67)<br><br>Personality of Surgeons mean (SD) by subscale:<br>- Extroversion 4.57 (1.37)<br>- Neuroticism 2.44 (1.46)<br><br>Surgeons are more extroverted and less neurotic than physicians.	71.43%

**McKenna (2020) A UK-wide analysis of trait emotional intelligence in occupational therapists**	Occupational Therapists (n=808)<br>Males (n=52)<br>Females (n=756)	Emotional Intelligence	Trait Emotional Intelligence Questionnaire-Short Form (TEIQue-SF)	Country:<br>UK<br>Method of Collection:<br>UK wide online survey to all OT practicing in UK in 2014/15.<br>Analysis:<br>descriptive statistics, t-tests, Mann Whitney and Kruskal-Wallis	Nurses global mean (SD) of 5.46 (5.11) and by subfactor:<br>- well-being 5.79 (0.82)<br>- self-control 4.93 (0.88)<br>- emotionality 5.76 (0.73)<br>- sociability 5.07 (0.78)<br><br>Occupational therapists EI score higher than the population norms for both global EI and each of the four subfactors.	85.71%
**McKinley (2015) A multi-institutional study of the emotional intelligence of resident physicians**	Resident Doctors (n=<br>Males (n=<br>Females (n=	Emotional Intelligence	Trait Emotional Intelligence Questionnaire (TEIQue)	Country:<br>USA<br>Method of Collection:<br>Surgical residents at 3 large academic medical centres; and Pathology and paediatric residents at one of these centres were eligible and invited via email to participate. Data collected via REDCap.<br>Analysis:<br>Fischer exact test, Wilcoxon 2-sample tests, MANOVA, post hoc pairwise comparisons	Resident doctors global EI mean (SD) of 101 (8.1).<br><br>Global EI of resident doctors is comparable with, but less variable than, the general population, but not statistically different between specialities.	71.43%

**McPhail (2002) The nursing profession, personality types and leadership**	Nurses (n=94)<br>Males (n=0)<br>Females (n=94)	Personality	Keirsey Temperament Sorter	<u>Country:</u><br>Canada<br><u>Method of Collection:</u><br>Random selection of active practicing nurses from Registered Nurses of Manitoba distributed and collected surveys by mail across 2001 - 2002.<br><u>Analysis:</u><br>descriptive statistics	Nursing personality frequency by subscale:<br>- NF (intuition/feeling) 21.1% (higher norm)<br>- NT (intuition/thinking) 16.67% (high than norm)<br>- SJ (sensing/judging) 57.78% (higher than norm)<br>- SP (sensing/perceiving) 4.40% (lower than norm)	85.71%
**Meesusen (2010) Personality dimensions and their relationship with job satisfaction amongst Dutch nurse anaesthetists.**	Nurse Anaesthetists (n=882)<br>Males (n=451)<br>Females (n=431)	Personality	Myers–Briggs Type Indicator (MBTI)	<u>Country:</u><br>Netherlands<br><u>Method of Collection:</u><br>Surveys were distributed at Dutch nurse anaesthetists congress and distributed by mail in January 2007 and closed in April 2007.<br><u>Analysis:</u><br>Factor and multiple regression analysis	Nurse anaesthetists personality dimensions in order of frequency mean (SD):<br>- orderly 4.03 (0.47)<br>- receptive 3.95 (0.48)<br>- easy going 3.92 (0.55)<br>- compassionate 3.55 (0.56)<br><br>Receptive dimension negatively correlates to other three personality dimensions.<br>Easy going in and orderly positive association to job satisfaction.	71.43%

**Membrive-Jiménez (2022) Explanatory Models of Burnout Diagnosis Based on Personality Factors and Depression in Managing Nurses**	Nurse Managers (n=86)<br>Males (n=unknown)<br>Females (n=unknown)	Personality	NEO Five-Factor Inventory (NEO FFI)	<u>Country:</u><br>Spain<br><u>Method of Collection:</u><br>Multicentre study convenience sample of nurse managers in Madulasia, Spain.<br><u>Analysis:</u><br>Descriptive statistics, categorical-response logistic ordinal regression model	Nurse manager personality mean (SD) by trait:<br>- Neuroticism 26.08 (6.05)<br>- friendliness (agreeableness) 46.85 (4.76)<br>- Responsibility (conscientiousness) 48.62 (5.12)<br>- Extraversion 44.69 (6.63)<br>- openness 38.86 (6.35)	100.00%
**Molavynejad (2019) Relationship between personality traits and burnout in oncology nurses**	Oncology Nurses (n=106)<br>Males (n=39)<br>Females (n=67)	Personality	NEO Five-Factor Inventory (NEO FFI)	<u>Country:</u><br>Iran<br><u>Method of Collection:</u><br>Oncology nurses working in Bagheri Hospital in Oct-Nov 2018.<br><u>Analysis:</u><br>descriptive statistics, t-tests, Pearson's correlation	Nursing personal mean (SD) scores by trait:<br>- neuroticism 27.41 (5.51)<br>- extraversion 44.86 (5.81)<br>- agreeableness 43.65 (8.30)<br>- conscientiousness 46.48 (6.75)<br>- openness 41.43 (6.41)	85.71%

**Montasem (2014) Subjective well-being in dentists: the role of intrinsic aspirations.**	Dentists (n=594)<br>Males (n=340)<br>Females (n=254)	Personality	The Aspiration Index	<u>Country:</u><br>United Kingdom<br><u>Method of Collection:</u><br>Random cluster sample of general practitioner dentists send via mail.<br><u>Analysis:</u><br>descriptive statistics, univariate and multiple regression analysis.	Dentists aspiration mean (SD) by subscale:<br>- Intrinsic importance 5.74 (0.78)<br>- Extrinsic importance 3.27 (1.14)<br>- Intrinsic likelihood 4.90 (0.96)<br>- Extrinsic likelihood 3.13 (1.01)<br>Break down for each gender in categorised data spreadsheet.<br><br>Dentists attached greater importance and likelihood ratings to intrinsic as opposed to extrinsic aspirations. Female dentists reported higher levels of both intrinsic and extrinsic aspiration importance, no differences were observed for other variables.	85.71%

**Moradian (2022) Emotional intelligence of nurses caring for COVID-19 patients: A cross-sectional study**	Nurses (n=211)<br>Males (n=98)<br>Females (n=111)	Emotional Intelligence	Bradberry & Greaves Emotional Intelligence Questionnaire	<u>Country:</u><br>Iran<br><u>Method of Collection:</u><br>Web based online questionnaire sent via email and social media, reminder sent at 1 week.<br><u>Analysis:</u><br>descriptive statistics, inferential statistics (t-tests, multinominal logistic regression, Spearman's correlation coefficient).	Nurses EI mean (SD) of 63.19 (8.22) (score range 0-100, where higher score = higher EI) and subscale:<br>- self-awareness 68.83 (11.46)<br>- self-management 56.41 (9.99)<br>- social awareness 63.24 (10.39)<br>- relationship management 66.55 (11.47)	85.71%

**Mullola (2018) Personality traits and career choices among physicians in Finland: employment sector, clinical patient contact, specialty and change of specialty**	Doctors (n=2837)<br>Males (n=999)<br>Females (n=1838)	Personality	Five Factor Model of personality (FFM)	<u>Country:</u><br>Finland<br><u>Method of Collection:</u><br>Doctors from the Finnish Medical Association were emailed to participate in web-based survey.<br><u>Analysis:</u><br>ANCOVA with post hoc, descriptive statistics	Doctors personality mean (SD) by gender for each trait:<br><br><u>Females</u><br>- extraversion 3.37 (0.90)<br>- conscientiousness 3.83 (0.75)<br>- openness 3.18 (0.74)<br>- agreeableness 3.40 (0.68)<br>- neuroticism 2.94 (0.84)<br><br><u>Males</u><br>- extraversion 3.19 (0.87)<br>- conscientiousness 3.62 (0.72)<br>- openness 3.30 (0.74)<br>- agreeableness 3.39 (0.67)<br>- neuroticism 2.62 (0.76)<br><br>Distinctive personality traits to be associated with physicians' career and specialty choices independent of known confounding factors. Openness was the most consistent personality trait associated with physicians' career choices.	85.71%

**Myhren (2013) Job Satisfaction and Burnout among Intensive Care Unit Nurses and Physicians**	Intensive Care Nurses and Doctors (n=145)<br>Males (n=23)<br>Females (n=122)<br><br>Nurses (n=129)<br>Doctors (n=16)	Personality	Basic Character Inventory (BCI)	Country:<br>Norway<br>Method of Collection:<br>Nurses at 3 ICU's at Oslo University Hospital. Questionnaire delivered by mail to eligible staff, with 1 reminder follow up. Completed surveys returned in a staff box in their resting room.<br>Analysis:<br>Descriptive statistics, t-tests, correlations, linear regression analyses.	Nurses personality mean (CI) by trait:<br>- neuroticism 3.1 (2.7-3.4)<br>- extroversion 5.7 (5.2-6.1)<br>- control 3.9 (3.6-4.2)<br><br>Doctors personality mean (CI) by trait:<br>- neuroticism 2.8 (1.8-3.8)<br>- extroversion 6.1 (4.7-7.4)<br>- control 2.9 (1.7-4.1)<br><br>*score ranges from 0 (low) - 9 (high)	71.43%
**Nagel (2016) The emotional intelligence of registered nurses commencing critical care nursing.**	Critical Care Nurses (n=30)<br>Males (n=unknown)<br>Females (n=unknown)	Emotional Intelligence	TEIQUe-SF	Country:<br>South Africa<br>Method of Collection:<br>Rn's commencing work in critical care in 7 private hospitals in Gauteng, between Sept 20212 - July 2013. Hand distributed and collected questionnaires.<br>Analysis:<br>Descriptive statistics.	Critical care nurses global EI mean (SD) (range) of 148.97 (30.46) (94-197).<br>Wide dispersion of scores.<br>Subscale mean scores:<br>- well-being 5.27<br>- emotionality factor 5.24<br>- self-control 4.89<br>- sociability 4.10<br><br>Higher range of EI demonstrated by critical care nurses, with well-being factor scoring highest, followed by the emotionality factor, self-	85.71%

					control, and sociability scoring lowest.	
**Narumoto (2008) Relationships among burnout, coping style and personality: study of Japanese professional caregivers for elderly.**	Nursing Home Healthcare workers (n=72)<br>Males (n=15)<br>Females (n=57)<br><br>Nurses (n=19)<br>Nursing Assistants (n=49)<br>Social Worker (n=2)<br>Occupational Therapist (n=1)<br>Physiotherapist (n=1)	Personality	NEO Five-Factor Inventory (NEO-FFI)<br>Maslach Burnout Inventory (MBI)	Country:<br>Japan<br>Method of Collection:<br>All healthcare professionals at two nursing homes.<br>Analysis:<br>Multivariate ANOVA, stepwise regressions and structural equation modelling examining relationships.	Nurses lower personality neuroticism 23.9 (7.0) compared to nursing assistants 27.3 (6.5).	85.71%

**Nash (2009) Personality, gender and medico-legal matters in medical practice.**	General Practitioners (n=566)<br>Males (65%)<br>Females (35%)	Personality	Eysenck Personality Questionnaire	Country:<br>Australia<br>Method of Collection:<br>Sample selected from a list of all GPs<br>who were insured in 2006 with the Australian medical defence organization, UNITED Medical Protection ('UNITED'), the largest medical insurer in Australia at<br>that time, distributed by mail.<br>Analysis:<br>Analysis between groups and covariates completed with t-tests, one-way analysis, multivariate analysis and turkey HSD.	General practitioner personality mean (SD) for subscale psychotism:<br>- Part-time males 2.44 (1.66)<br>- Part-time females 1.78 (1.17)<br>- Full-time males 2.51 (1.45)<br>- Full-time females 1.76 (1.32)<br>- Over-time males 2.38 (1.46)<br>- Over-time females 2.36 (1.84)<br>General practitioner personality mean (SD) for subscale extraversion:<br>- Part-time males 4.25 (3.00)<br>- Part-time females 6.03 (3.36)<br>- Full-time males 6.17 (3.62)<br>- Full-time females 7.04 (3.51)<br>- Over-time males 5.79 (3.45)<br>- Over-time females 6.05 (3.42)<br>General practitioner personality mean (SD) for subscale neuroticism:<br>- Part-time males 3.89 (3.60)<br>- Part-time females 4.47 (3.00)<br>- Full-time males 3.57 (3.36)<br>- Full-time females 4.00 (3.00)<br>- Over-time males 3.62 (2.90)<br>- Over-time females 4.24 (3.75)<br>Male respondents had significantly higher psychoticism scores than females, and females had	85.71%

		significantly higher neuroticism scores than males, as in community samples.<br>Male working part-time are more introverted than males working full-time or greater.<br>Females who work more than 48 hours per week demonstrate no differences in personality traits compared to males working the same number of hours.

**Needleman (2011) Personality types of paediatric dentists: comparative analysis and associated factors.**	Paediatric Dentists (n=214)<br>Males (n=125)<br>Females (n=89)	Personality	Myers-Briggs Type Indicator	<u>Country:</u><br>USA<br><u>Method of Collection:</u><br>Random selection of paediatric dentists listed on professional registration list mailed questionnaire.<br><u>Analysis:</u><br>Univariate and multivariate logistic regressions establish predictivity of personality dichotomy.	6 most prevalent personality types among paediatric dentists accounted for 73% of the entire sample:<br>(1) ISTJ (16%)<br>(2) ISFJ (14%)<br>(3) ESTJ (13%)<br>(4) ESFJ (13%)<br>(5) ENFJ (9%)<br>(6) ENFP (9%)<br><br>Paediatric personality traits percentage:<br>- extraversion 52%<br>- introversion 48%<br>- sensing 62%<br>- intuition 38%<br>- thinking 43%<br>- feeling 57%<br>- judging 79%<br>- perceiving 21%<br><br>Paediatric dentists increased sensing when compared to paediatricians; increased feeling compared to dental specialists, general dentists, and paediatricians; judging rather than perceiving compared to paediatrician and the general population.	71.43%

**NizarAbdulMajeed (2019) Association of Occupational Stress and Emotional Intelligence among Physiotherapists in Malaysia: A Cross-sectional Study**	Physiotherapists (n=171)<br>Males (n=54)<br>Females (n=117)	Emotional Intelligence	Genos Emotional Intelligence Inventory – Concise Questionnaire (GEII)	Country:<br>Malaysia<br>Method of Collection:<br>Convenience sample of physiotherapists at working at government and private settings in Malaysia.<br>Analysis:<br>Descriptive statistics, Pearson correlation, t-tests, one-way ANOVA.	Physiotherapists have a moderate level of EI mean (SD) score of 129.36 (18.314).	57.14%

**Nooryan (2012) Controlling anxiety in physicians and nurses working in intensive care units using emotional intelligence items as an anxiety management tool in Iran**	Intensive Doctors and Nurses (n=150)<br>Males (n=unknown)<br>Females (n=unknown)<br><br>Nurses (n=120)<br>Doctors (n=30)	Emotional Intelligence	Bar-on emotional intelligence	Country:<br>Iran<br>Method of Collection:<br>Intensive care doctors and nurses in hospitals in Iran in 2010,<br>Analysis:	Doctors total EI mean (SD) of 495.46 (35.75) and but subscale:<br>- self-regard 35.63 (5.28)<br>- self-awareness 28.10 (3.03)<br>- assertiveness 25.96 (3.16)<br>- independence 25.4 (2.51)<br>- self-actualisation 34.00)2.85)<br>- empathy 32.30 (2.54)<br>- social responsibility 36.76 (3.66)<br>- interpersonal 42.66 (5.94)<br>- stress tolerance 33.96 (3.58)<br>- impulse control 35.70 (3.42)<br>- reality testing 36.66 (2.44)<br>- flexibility 29.56 (3.72)<br>- problem-solving 32.89 (2.54)<br>- optimism 30.26 (3.45)<br>- happiness 35.60 (4.61)<br><br>Nurses total EI mean (SD) of 479.65 (31.58) and but subscale:<br>- self-regard 34.91 (4.81)<br>- self-awareness 27.16 (4.17)<br>- assertiveness 24.97 (23.86)<br>- independence 24.23 (3.03)<br>- self-actualisation 33.49 (2.72)<br>- empathy 31.48 (3.43)<br>- social responsibility 35.89 (3.90)	57.14%

- interpersonal 41.24 (5.34)
- stress tolerance 31.75 (4.03)
- impulse control 32.62 (6.00)
- reality testing 35.81 (3.94)
- flexibility 29.10 (4.54)
- problem-solving 31.17 (3.26)
- optimism 31.02 (3.28)
- happiness 34.76 (3.25)

Mean emotional intelligence scores are higher in physicians than nurses.

**Ntantana (2017) Burnout and job satisfaction of intensive care personnel and the relationship with personality and religious traits: An observational, multicentre, cross-sectional study.**	ICU Physicians and Nurses (n=469)<br>Males (n=121)<br>Females (n=323)<br><br>Physicians (n=149)<br>Nurses (n=320)	Personality	Maslach Burnout Inventory<br>Eysenck Personality Questionnaire	<u>Country:</u><br>Greece<br><u>Method of Collection:</u><br>Physicians and nurses working full-time in 18 Greek ICU departments from June to December 2015.<br><u>Analysis:</u><br>Descriptive statistics, Chi-square, Mann-Whitney U, Kruskal-Wallis, Spearman correlation, univariate and multivariate logistic regression.	ICU Physician personality scores by subscale:<br>- neuroticism: 9<br>- psychoticism: 3<br>- extraversion: 15<br>ICU Nurses personality scores by subscale:<br>- neuroticism: 10<br>- psychoticism: 3<br>- extraversion: 15<br>Neuroticism positive and extraversion negative predictor of exhaustion.	85.71%
**O'Mahony (2018) Compassion Fatigue Among Palliative Care Clinicians: Findings on Personality Factors and Years of Service**	Healthcare workers (n=70)<br>Males (n=12)<br>Females (n=58)<br><br>Doctors (n=22)<br>Nurses (n=18)<br>Social workers (n=11)<br>chaplains (n=8)<br>Nurse Practitioner (n=6)<br>Doctor assistant (n=1)	Personality	Mini-International Personality Item Pool (Mini-IPIP)	<u>Country:</u><br>USA<br><u>Method of Collection:</u><br>Participants attending continuing education program on palliative care medicine, completed survey prior to participation. Data collected in Sept 2015.<br><u>Analysis:</u><br>Descriptive statistics, Spearman correlations, multiple regressions models	Doctors lower levels of neuroticism mean (SD) of 10.31 (2.8)<br>Social workers lower level of neuroticism mean (SD) of 11.55 (2.16)	100.00%

**Ogunyemi (2011) Associations between DISC assessment and performance in obstetrics and gynaecology residents**	Residents (n=46)<br>Males (n=<br>Females (n=	Behaviour	DiSC	Country:<br>USA<br>Method of Collection:<br>Self-administered as an educational needs assessment to residents between 2007-2009.<br>Analysis:<br>Descriptive statistics, t-tests, ANOVA, Spearman's correlations	Residents with negative nurse comments behaviour mean (SD) by style:<br>- Dominance 3.06 (0.43)<br>- influence 3.63 (0.51)<br>- Steadiness 4.94 (0.45)<br>- Conscientiousness 4.94 (0.44)<br><br>Residents with favourable nurse comments behaviour mean (SD) by style:<br>- Dominance 2.83 (0.37)<br>- influence 3.63 (0.41)<br>- Steadiness 4.93 (0.51)<br>- Conscientiousness 5.2 (0.34)	85.71%
**Ohlson (2015) Ability emotional intelligence of nurse managers in the Midwestern United States**	Nurse Managers (n=87)<br>Males (n=10)<br>Females (n=77)	Emotional Intelligence	Mayer-Salovey-Caruso Emotional Intelligence Test (MSCEIT)	Country:<br>USA<br>Method of Collection:<br>Nurse managers from 8 acute care hospitals completed paper-form completion of survey.<br>Analysis:<br>Descriptive statistics inferential statistics	Nurse managers global EI mean (SD) of 98.1 (13.1) indicating closely reflects normal population.	71.43%

**Okumura (2022) Personality traits affect critical care nursing competence: A multicentre cross-sectional study**	Intensive Care Nurses (n=211)<br>Males (n=47)<br>Females (n=164)	Personality	Ten-Item Personality Inventory - Japanese (TIPI-J)	<u>Country:</u><br>Japan<br><u>Method of Collection:</u><br>Nurses in 4 tertiary level ICU's and 2 university hospitals in Japan between Aug 2017 - Dec 2018. Paper forms distributed and collected.<br><u>Analysis:</u><br>descriptive statistics, correlations and comparisons	Nurses personality mean (SD) by trait:<br>- extraversion 4.42 (1.38)<br>- agreeableness 4.78 (0.96)<br>- conscientiousness 3.74 (0.99)<br>- neuroticism 4.25 (1.19)<br>- openness 3.79 (1.06)<br><br>Neuroticism had a direct or indirect negative effect on all four nursing competencies.	100.00%
**Ordu (2022) The Relationship Between Nurses' Emotional Intelligence Skills and Positive Mental Health**	Nurses (n=331)<br>Males (n=36)<br>Females (n=295)	Emotional Intelligence	Emotional Intelligence Assessment Scale (EIA)	<u>Country:</u><br>Turkey<br><u>Method of Collection:</u><br>Nurses at university hospital in Aegean region of Turkey surveyed between Sept - Dec 2019.<br><u>Analysis:</u><br>descriptive statistics, Spearman's correlations analysis	Nurses global EI mean (SD) score of 145.22 (24.71) (Score of >155 = high level of EI, 130 - 154 = medium level; <129 = low level).; subscale scores:<br>- self-awareness 28.10 (6.23)<br>- self-regulation 29.20 (5.51)<br>- self-motivation 29.22 (5.37)<br>- empathy 29.27 (5.56)<br>- social skills 29.42 (5.55)	85.71%

**Ortega-Campos (2019) A Multicentre Study of Psychological Variables and the Prevalence of Burnout among Primary Health Care Nurses**	Nurses (n=338)<br>Males (n=142)<br>Females (n=196)	Personality	NEO Five-Factor Inventory (NEO FFI) - 5 point scale	Country:<br>Spain<br>Method of Collection:<br>Generalist or specialists nurses across multisite in Andalusia Public Health Service in Spain.<br>Analysis:<br>descriptive statistics, Pearson's correlation coefficients	Nursing personality mean (SD) by personality trait:<br>- Neuroticism 27.96 (8.33) (min-max 12-53)<br>- Extraversion 42.19 (7.90) (min-max 20-60)<br>- Openness 38.84 (6.68) (min-max 20-57)<br>- Agreeableness 44.80 (7.54) (min-max 19-60)<br>- Conscientiousness 46.73 (7.37) (min-max 20-60)<br><br>High levels of neuroticism and low ones of agreeableness and extraversion, higher prevalence of burnout.	85.71%

**Pajonk (2012) Trauma and Stress-Related Disorders in German Emergency Physicians: The Predictive Role of Personality Factors**	Emergency Doctors (n=487)<br>Males (n=369)<br>Females (n=118)	Personality	Hamburg Personality Inventory (HPI-K84)	Country:<br>Germany<br>Method of Collection:<br>44 major centre for emergency medicine in Germany, sent questionnaires.<br>Analysis:<br>descriptive statistics, post hoc with Bonferroni correction, Chi-squared test or Exact-Fischer tests.	38.1% of doctors considered anxious type - mod/high scores for neuroticism, slightly above average for agreeableness and low scores risk taking.<br><br>20.2% of doctors considered the unconventional type - characterised by openness, creativity and lower levels of conscientiousness.<br><br>30.1% of doctors are the crisis-manger type - risk-taking, resilient, controlled and considerate. Mod/high scores for extraversion and conscientiousness and slightly below average neuroticism.<br><br>11.5% of doctors are the cool type - very low scores for extraversion, openness and agreeableness.	85.71%

**Pålsson (1996) Burnout, empathy and sense of coherence among Swedish district nurses before and after systematic clinical supervision.**	District Nurses (n=33)<br>Males (n=unknown)<br>Females (n=unknown)<br><br>Supervisory Group (n=21)<br>Comparison Group (n=12)	Personality	Karolinska Scales of Personality	Country:<br>Sweden<br>Method of Collection:<br>District nurses across 10 primary health care district who participated in training programme were eligible. Non-random allocation of participants to supervision after training and others in comparison with no supervision.<br>Analysis:	Nursing Personality (supervisory group) at baseline median for anxiety proneness subscales:<br>- somatic anxiety 16.5<br>- psychic anxiety 21.5<br>- muscular tension 15<br>- psychasthenia 22.5<br>Nursing Personality (supervisory group) at baseline median for extraversion subscales:<br>- impulsiveness 23<br>- monotony avoidance 21<br>- detachment 20.5<br>- socialisation 69<br>Nursing Personality (supervisory group) at baseline median for aggression subscales:<br>- aggression 34<br>- hostility 21<br>- inhibition of aggression 25.5<br><br>Nursing Personality (comparison group) at baseline median for anxiety proneness subscales:<br>- somatic anxiety 13.5<br>- psychic anxiety 19.5<br>- muscular tension 15.5	85.71%

- psychasthenia 20.5

Nursing Personality (comparison group) at baseline median for extraversion subscales:

- impulsiveness 22
- monotony avoidance 22
- detachment 17
- socialisation 68.5

Nursing Personality (comparison group) at baseline median for aggression subscales:

- aggression 31.5
- hostility 19
- inhibition of aggression 22

No differences were found at baseline between the supervisory group (n=21) and the comparison group (n = 12) personality traits, except on two of the KSP subscales-detachment and inhibition of aggression which showed significantly lower scores in the comparison group.

**Papanagnou (2017) An assessment of emotional intelligence in emergency medicine resident physicians**	Medical Residents (n=35)<br>Males (n=21)<br>Females (n=14)	Emotional Intelligence	Bar-On's Emotional Quotient Inventory 2.0 (EQ-I 2.0)	Country:<br>USA<br>Method of Collection:<br>electronic distribution to residents in Thomas Jefferson University Hospital resident program.<br>Analysis:<br>Descriptive statistics	Resident EI mean (SD) of 103.3 (13.5), higher EI than general pop mean (SD) 100 (15).	85.71%
**Pascual (2021) Type A Behaviour Patterns Among Neurosurgeons and Neurosurgical Trainees**	Neurosurgeons (n=41)<br>Males (n=34)<br>Females (n=7)	Behaviour	Modified Bortner Measure of Type A behaviour	Country:<br>USA<br>Method of Collection:<br>Survey completed via Google Forms.<br>Analysis:<br>descriptive statistics, multiple regression analysis	Neurosurgeons Type A mean 86.44.<br>Score range from 14-154, neutral score of 84, higher score more to have Type A behaviour.	57.14%
**Pau (2015) Relationship between emotional intelligence and job satisfaction in newly qualified Malaysian dentists.**	Dentists (n=342)<br>Males (n=80)<br>Females (n=132)	Emotional Intelligence	Schutte Emotional Intelligence Scale	Country:<br>Malaysia<br>Method of Collection:<br>Malaysian dentists employed in 3-year compulsory service posted survey between April - Jun 2008.<br>Analysis:<br>Linear regression analysis	Dentist mean (SD) (95% CI) EI of 122.01 (11.86) (120.8-123.2) and score by gender:<br>- Male 120.9 (118.2-123.5)<br>- Female 122.3 (11.0-123.7)<br><br>EI was statistically significantly associated with JS with patient relationships, peer support, professional development, quality of care, supporting staff and overall job satisfaction.	71.43%

**Pérez-Fuentes (2018) The Role of Emotional Intelligence in Engagement in Nurses**	Nurses (n=2126)<br>Males (n=321)<br>Females (n=1805)	Emotional Intelligence	Reduced Emotional Intelligence Inventory for Adults (EQ-i-20M)	<u>Country:</u><br>Spain<br><u>Method of Collection:</u><br>Random sample from various health centres.<br><u>Analysis:</u><br>Comparisons with t-tests, and linear regressions to determine relationships. Nonlinear predicative CHAID regression to determine predictor variable and relation to criterion based on median score.	<u>Total</u> nursing EI mean (SD) by subscale:<br>- Intrapersonal 2.62 (0.698)<br>- Interpersonal 3.06 (0.501)<br>- Stress Management 3.25 (0.567)<br>- Adaptability 2.91 (0.526)<br>- General Mood 3.08 (0.599)<br><br><u>Male</u> nursing EI mean (SD) by subscale:<br>- Intrapersonal 2.46 (0.690)<br>- Interpersonal 2.94 (0.530)<br>- Stress Management 3.26 (0.569)<br>- Adaptability 2.96 (0.527)<br>- General Mood 3.11 (0.607)<br><br><u>Female</u> nursing EI mean (SD) by subscale:<br>- Intrapersonal 2.65 (0.696)<br>- Interpersonal 3.08 (0.493)<br>- Stress Management 3.20 (0.567)<br>- Adaptability 2.90 (0.526)<br>- General Mood 3.08 (0.598)<br><br>Women exhibited higher levels of emotional intelligence in some emotional intelligence components (interpersonal and	85.71%

				intrapersonal).<br>Age negatively correlated with EI interpersonal factor and positively correlated with stress management.<br>Nurses with higher levels of emotional intelligence also scored more highly in engagement, with the interpersonal factor being the greatest predictor of engagement.

**Pérez-Fuentes (2019) Explanatory Value of General Self-Efficacy, Empathy and Emotional Intelligence in Overall Self-Esteem of Healthcare Professionals**	Health Professionals (n=386)<br>Males (n=65)<br>Females (n=321)	Emotional Intelligence	Brief Emotional Intelligence Inventory for Senior Citizens (EQ-i-20M)	Country:<br>Spain<br>Method of Collection:<br>Questionnaires completed via web platform.<br>Analysis:<br>descriptive statistics, Pearson's correlation coefficient.	Health Professional EI mean (SD by subscale:<br>- intrapersonal 10.37 (2.73)<br>- interpersonal 11.87 (2.02)<br>- stress management 6.75 (1.77)<br>- adaptability 11.2 (2.05)<br>- general mood 12.35 (2.27)	71.43%
**Placek (2019) A Cross-Sectional Study of Emotional Intelligence in Military General Surgery Residents**	Surgical Residents (n=46)<br>Males (n=27)<br>Females (n=19)	Emotional Intelligence	Trait Emotional Intelligence Questionnaire (TEIQue)	Country:<br>USA<br>Method of Collection:<br>Surgery residents in all year levels were surveyed a beginning of the year in June 2016.<br>Analysis:<br>Descriptive statistics, t-tests, Fisher's exact test, linear regressions, Pearson's correlations.	Female Residents total EI mean (SD) 5.14 (0.36), and subscale:<br>- emotionality 4.94 (0.58)<br>- sociability 4.77 (0.55)<br>- well-being 5.59 (0.59)<br>- self-control 5.12 (0.60)<br><br>Male Residents total EI mean (SD) 5.06 (0.48), and subscale:<br>- emotionality 5.16 (0.50)<br>- sociability 4.88 (0.70)<br>- well-being 5.51 (0.57)<br>- self-control 5.13 (0.51)<br><br>Females show higher global EI, and both males and females scored higher in the self-control factor than the normative population.	100.00%

**Popa-Velea (2019) Factors Associated with Burnout in Medical Academia: An Exploratory Analysis of Romanian and Moldavian Physicians**	Academic Doctors (n=200)<br>Males (n=80)<br>Females (n=120)	Emotional Intelligence	Schutte Self-Report Emotional Intelligence Test (SSEIT)	Country:<br>Romania<br>Method of Collection:<br>Academic doctors employed at university of Medicine and Pharmacy in Romania and Moldova.<br>Analysis:<br>Descriptive statistics, Mann-Whitney U tests, hierarchical regressions	Academic doctors EI mean (SD) across two facilities were 125.38 (14.14) and 100.46 (27.1) respectively.	85.71%
**Por Pan (2020) Self-perception of leadership style of dentists: heads of dental departments in community hospitals, Southern Thailand**	Head of Department Dentists (n=133)<br>Males (n=44)<br>Females (n=89)	Emotional Intelligence	Emotional Intelligence Screening Test	Country:<br>Thailand<br>Method of Collection:<br>All Dental department heads at community hospitals in Southern Thailand with dental departments during November 2016 - February 2017 were sent the questionnaire and returned by mail.<br>Analysis:	Total EI mean (SD) (range) of 172.31 (16.40) (132-205).<br><br>EI in normal range for general Thai population. Though also found to be a associated with a significantly increased the transformational leadership score.	85.71%

**Prado-Gascó (2019) The influence of nurse education and training on communication, emotional intelligence, and empathy**	Nurses (n=483)<br>Males (n=101)<br>Females (n=313)<br><br>Degree (n=295)<br>Masters (n=66)<br>Doctorate (n=9)	Emotional Intelligence	Trait Meta Mood Scale (TMMS-24)	Country:<br>Spain<br>Method of Collection:<br>Nurses who provided direct care in 7 public hospitals in Valencia. Nil specific details re data collection method.<br>Analysis:<br>Descriptive statistics, one-way ANOVA, Pearson's correlations, hierarchical multiple linear regression	Nurses with degree mean (SD) by subscale:<br>- emotional attention 3.57 (0.76)<br>- emotional clarity 3.80 (0.69)<br>- emotional repair 3.83 (0.73)<br><br>Nurses with masters mean (SD) by subscale:<br>- emotional attention 3.68 (0.80)<br>- emotional clarity 4.01 (0.64)<br>- emotional repair 3.86 (0.83)<br><br>Nurses with Doctorate mean (SD) by subscale:<br>- emotional attention 3.69 (0.79)<br>- emotional clarity 4.17 (0.50)<br>- emotional repair 4.18 (0.50)<br><br>Nurses with only a degree and less specialized training had lower empathy and emotional intelligence.	71.43%

**Prins (2019) The relationship between burnout, personality traits, and medical specialty. A national study among Dutch residents**	Residents (n=1231)<br>Males (n=325)<br>Females (n=906)	Personality	Five Factor Model of personality (FFM)	Country:<br>Netherlands<br>Method of Collection:<br>Nationwide survey emailed to residents on the Dutch Registration Commission of Medical Specialities in Sept 2015.<br>Analysis:<br>descriptive statistics, ANOVA, Pearson's correlations, t-tests, multivariable logistic regressions	Residents personality mean (SD) by trait:<br>- Openness 3.41 (0.47)<br>- extraversion 3.57 (0.59)<br>- neuroticism 2.51 (0.58)<br>- agreeableness 3.90 (0.41)<br>- conscientiousness 3.90 (0.46)<br><br>Residents scoring high on neuroticism reported more burnout.<br>Extraverted residents less susceptible to burnout.	85.71%

**Prufeta (2017) Emotional Intelligence of Nurse Managers: An Exploratory Study.**	Nurse Managers (n=38)<br>Males (n=6)<br>Females (n=32)	Emotional Intelligence	MSCEIT	<u>Country:</u><br>USA<br><u>Method of Collection:</u><br>Convenient sample at large urban academic medical centre in northwest emailed online survey.<br><u>Analysis:</u><br>descriptive statistics, t-tests, ANOVA, Tukey post hoc analysis.	Nurse managers total EI mean (SD) score of 96.65 (15.11), consider low average.<br>Frequency of scores for each score interpretation:<br>- consider development 5.3%<br>- consider improvement 26.3%<br>- low average 21.1%<br>- high average 34.2%<br>- competent 5.3%<br>- strength 7.9%<br>Nurse managers total EI mean (SD) scores by gender:<br>- Male 94.68 (low average)<br>- Female 97.58 (low average)<br><br>Nurse managers with <2 years of experience had significant lower "using emotions" score and strategic EI. Nurse managers with a masters' degree in nursing scored higher in "using emotions" score than did those with a masters 'degree in a related field.	85.71%

**Radonsky (1980) Personality Characteristics of the Published and Nonpublished Occupational Therapist**	Occupational Therapists (n=62)<br>Males (n=<br>Females (n=<br><br>Publishing (n=19)<br>non-publishing (n=43)	Personality	Myers–Briggs Type Indicator (MBTI)	Country:<br>USA<br>Method of Collection:<br>Random sample of AOTA, sent MBTI forms and returned to researchers.<br>Analysis:<br>t-tests, regression analyses, chi-square tests.	Publishing OT's were ENTJ personality type, whilst non-publishing OT's were ISFJ, representing 31% and 69% respectively of the total participants.	85.71%
**Raeissi (2019) Relationship between communication skills and emotional intelligence among nurses**	Nurses (n=253)<br>Males (83.4%)<br>Females (16.6%)	Emotional Intelligence	Goleman's Emotional Intelligence Scale	Country:<br>Iran<br>Method of Collection:<br>Nursing working at 5 hospitals affiliated to university recruited using census method. Those that returned questionnaires were included.<br>Analysis:<br>One-way analysis of variance, independent t-test and correlation tests (Pearson and Spearman)	Total nursing EI mean (SD) scores of 78.31 (12.88) and subscale scores:<br>- self-awareness 20.83 (3.94)<br>- self-management 18.19 (4.05)<br>- social awareness 19.40 (3.25)<br>- social skills 19.89 (4.03)<br><br>Highest and lowest scores related to self-awareness (20.83) and self-management (18.19) dimensions respectively.<br>Positive relationship between nurses' EI and communication skills.	71.43%

**Rakhshani (2018) The Relationship between Emotional Intelligence and Job Stress among Nurses in Shiraz, Iran**	Nurses (n=500)<br>Males (n=90)<br>Females (n=397)<br>Unspecified (n=13)	Emotional Intelligence	Siberia Schering's Emotional Intelligence Standard Questionnaire	<u>Country:</u><br>Iran<br><u>Method of Collection:</u><br>Multistage cluster method of 5 public and 5 private hospitals and random sample of 50 nurses from each hospital. Hand delivery of questionnaires and collected the next day.<br><u>Analysis:</u><br>t-test, one-way ANOVA, Pearson correlations, and simple and multiple regression.	Nurse global EI mean (SD) f 113.59 (14.70), and subscale:<br>- self-awareness 29.01 (4.70)<br>- self-control 22.98 (4.16)<br>- self-motivation 23.08 (3.25)<br>- social consciousness 20.62 (3.62)<br>- social skills 17.04 (3.14)<br><br>Relatively strong inverse relationship between the nurses' emotional intelligence and job stress.	100.00%
**Ramsey-Haynes (2021) Emotional intelligence and workplace incivility among oncology RNs**	Oncology Nurses (n=100)<br>Males (n=10)<br>Females (n=90)	Emotional Intelligence	Trait Emotional Intelligence Questionnaire-Short Form (TEIQue-SF)	<u>Country:</u><br>USA<br><u>Method of Collection:</u><br>Survey completed online anonymously.<br><u>Analysis:</u><br>Descriptive statistics	Nurses global EI mean (SD) of 169.01 (16.64) (total possible score of 30-210 - higher scores higher EI), subscale scores:<br>- self-control 32.42 (4.51)<br>- sociability 30.13 (4.83)<br>- well-being 37.21 (3.20)<br>- emotionality 45.43 (5.40)	71.43%

**Ramzan Shahid (2018) Promoting wellness and stress management in residents through emotional intelligence training**	Residents (n=31)<br>Males (n=9)<br>Females (n=22)	Emotional Intelligence	Bar-On Emotional Quotient Inventory 2.0, EQ-i 2.0	Country:<br>USA<br>Method of Collection:<br>Residents from a paediatric and med-paeds residency programs at a university volunteered to complete online EI survey in May 2015.<br>Analysis:<br>compared median score reports for the residents as a cohort before and after the intervention.	Total EI median (IQR) score at baseline of 110 (102-115) and composite and sub-composite scores of:<br>- Self-perception composite 106 (102-114)<br>- Self-regard 104 (95-106)<br>- self-actualisation 107 (104-117)<br>- emotional self-awareness 111 (101-122)<br>- self-expression composite 106 (94-113)<br>- emotional expression 108 (103-118)<br>- assertiveness 102 (91-109)<br>- independence 101 (90-110)<br>- interpersonal composite 114 (108-119)<br>- interpersonal relationships 105 (100-113)<br>- empathy 113 (110-121)<br>- social responsibility 112 (101-117)<br>- decision making composite 112 (101-117)<br>- problem solving 105 (90-110)<br>- reality testing 107 (100-117)<br>- impulse control 114 (105-	71.43%

123)
- stress management composite 105 (98-111)
- flexibility 105 (96-113)
- stress tolerance 104 (99-110)
- optimism 103 (95-113)
- wellness 104 (94-113)

Education intervention increases EI scores as well as several other components of EI. Teaching skills in self-awareness, self-management, social awareness, and social skills can improve stress management, promote wellness and prevent burnout.

**Ranjha (2010) Emotional Intelligence and Psychological Adjustment of Nurses Serving in Emergency and Non-emergency Wards**	Emergency and Non-emergency nurses (n=200)<br>Males (n=0)<br>Females (n=200)<br><br>< 5 years' experience (n=105)<br>>5 years' experience (n=95)	Emotional Intelligence	Bar-On Emotional Quotient Inventory	Country:<br>Pakistan<br>Method of Collection:<br>Nurses at 2x government hospitals in Lahore and Sargodha personally approached to participate and complete questionnaires.<br>Analysis:<br>Correlation, regression and t tests.	EI of nurses mean (SD) by wards:<br>- emergency wards 433.2 (57.9)<br>- non-emergency wards 436.1 (57.4)<br><br>EI of nurses mean (SD) by experience:<br>- < 5 years 442.6 (59.5)<br>- > 5 years 425.9 (54.2)<br>Low professional experience were high on EI than nursed with high professional experience.<br><br>EI is significantly correlated with Psychological Adjustment and was a predictor of EI. Wards did not significantly predict EI.	85.71%
**Reemts (2015) Emotional intelligence levels in baccalaureate-prepared early career registered nurses**	Nurses (n=149)<br>Males (n=unknown)<br>Females (n=unknown)	Emotional Intelligence	Mayer-Salovey-Caruso Emotional Intelligence Test (MSCEIT)	Country:<br>USA<br>Method of Collection:<br>Web-based survey distribution via email to nursing participants from graduate years of 2007, 2008, 2009, 2010<br>Analysis:<br>descriptive statistics,	Nurses EI mean (SD) of 99.08 (11.39) (competent) and subscale scores of:<br>- perceiving emotions 100.11 (12.35) - competent<br>- using emotions 97.77 (12.83) - competent<br>- understanding emotions 98.64 (9.88) - competent	85.71%

	- managing emotions<br>99.08(11.39) - competent

**Reeve (1980) Personality characteristics of a sample of anaesthetists.**	Anaesthetists (n=231)<br>Males (n=171)<br>Females (n=60)<br><br>Consultants (n=84)<br>Senior Registrars (n=57)<br>Registrars (n=76)<br>SHOs (n=14)	Personality	Cattell's 16 PF Form C	<u>Country:</u><br>United Kingdom<br><u>Method of Collection:</u><br>Data collected in three stages, nil details on who recruited or collected.<br><u>Analysis:</u><br>Descriptive statistics and frequencies.	Anaesthetists mean personality profile by subscale:<br>- (A) Aloof - Warm 4.9<br>- (B) Dim - Bright 6.6<br>- (C) Unstable - Stable 5.5<br>- (E) Docile - Dominant 6<br>- (F) Serious - Happy-go-lucky 4.6<br>- (G) Expedient - Persistent 5.4<br>- (H) Shy - Bold 4.9<br>- (I) Tough - Sensitive 5.7<br>- (L) Trusting - Suspicious 5.3<br>- (M) Careful - Casual 5.5<br>- (N) Open - Shrewd 5.6<br>- (O) Confident - Unsure 6<br>- (Q1) Tolerant - Critical 5.2<br>- (Q2) Dependent - Independent 7.1<br>- (Q3) Lax - Controlled 5.6<br>- (Q4) Calm - Tense 6<br><br>In comparison with the general adult population, anaesthetists is less outgoing (A, H), brighter (B), more ascendant and dominant (E), more serious (F), more self-reproaching, unsure (0), more self-sufficient (Q2) and more tense and frustrated (Q4).	85.71%

**Rich (1987) Personality hardiness and burnout in female staff nurses.**	Nurses (n=100)<br>Males (n=0)<br>Females (n=100)	Personality	Hardiness<br>Staff Burnout Scale for Health Professionals (SBS-HP)	<u>Country:</u><br>USA<br><u>Method of Collection:</u><br>Nurses were from an acute care, full-service hospital in western Pennsylvania with at least one year's experience. Questionnaire were distributed and returned within a one-week period.<br><u>Analysis:</u><br>Comparison between hardiness and burnout using regression analysis.	Nurses <u>burnout</u> mean (SD) of 49.95 (17.76).<br><br>Nurses <u>hardiness</u> mean (SD) subscales:<br>- Alienation work 2.96 (2.69)<br>- Alienation self-1.71 (1.72)<br>- Security 19.45 (5.96)<br>- Powerlessness 8.79 (5.98)<br>- Locus of control 9.15 (4.23)<br><br>Nurses mean <u>burnout</u> relative to hardiness of <u>younger age</u>:<br>- High hardiness 51.93<br>- Low hardiness 60.48<br>Nurses mean <u>burnout</u> relative to hardiness of <u>older age</u>:<br>- High hardiness 40.48<br>- Low hardiness 50.94<br><br>On the hardiness measures, in comparing the scores of previous research, female nurses scored more externally in locus of control than males and females show greater externality.<br>Young nurses are particularly prone to burnout.	71.43%

**Rolander (2008) Relationships between psychosocial work environmental factors, personality, physical work demands and workload in a group of Swedish dentists**	Dentists (n=77)<br>Males (n=36)<br>Females (n=41)	Personality	Eysenck Personality Questionnaire (EPQ)	<u>Country:</u><br>Sweden<br><u>Method of Collection:</u><br>Questionnaire distributed via program esmaker Nx online.<br><u>Analysis:</u><br>descriptive statistics, correlations	Dentist personality mean (SD) by trait:<br>- neuroticism 7.82 (score range 0-24)<br>- extraversion 12.22 (score range 0-23)	85.71%
**Rosenthal (2015) Relationship between personality traits and pharmacist performance in a pharmacy practice research trial: CPJRPC**	Pharmacists (n=23)<br>Males (n=unknown)<br>Females (n=unknown)	Personality	Big Five Inventory (BFI)	<u>Country:</u><br>Canada<br><u>Method of Collection:</u><br>Alberta community pharmacists, who obtained additional prescribing authorisation and consented to participate provide electronic of paper questionnaire.<br><u>Analysis:</u><br>Descriptive statistics, t-tests, exploratory post hoc testing, and linear regression analysis.	Pharmacists mean (SD) by personality traits:<br>- extraversion 3.56 (0.92)<br>- agreeableness 4.21 (0.46)<br>- conscientiousness 4.01 (0.71)<br>- neuroticism 2.51 (0.76)<br>- openness 3.67 (0.65)<br><br>Pharmacist more likely to exhibit behaviour in line with the traits of extroversion, agreeableness, conscientiousness and openness.	71.43%

**Sabanciogullari (2020) Comparison of Newly Graduated Nurses' and Doctors' Opinions About Spiritual Care and Their Emotional Intelligence Levels**	Newly Graduate Nurses and Doctors (n=100)<br>Males (n=)<br>Females (n=X)<br><br>Nurses (n=50)<br>Males (20%)<br>Females (80%)<br><br>Doctors (n=50)<br>Males (54%)<br>Females (46%)	Emotional Intelligence	Schutte's Emotional Intelligence Test (SSEIT)	Country:<br>Turkey<br>Method of Collection:<br>All newly graduates nurses and doctors from same university eligible. Samples were matched in size, of those who agreed to participate. Questionnaires were collected during interview process prior to leaving the university.<br>Analysis:<br>Descriptive statistics, Chi-square tests and person correlations for relationships.	EI newly graduate nurses mean (SD) total score of 156.56 (18.69) and subscale:<br>- optimism/emotion regulation 46.68 (6.11)<br>- benefiting from emotions 22.64 (3.49)<br>- emotion expression 38.02 (5.56)<br><br>EI newly graduate doctors mean (SD) total score of 145.44 (13.29) and subscale:<br>- optimism/emotion regulation 44.10 (6.16)<br>- benefiting from emotions 21.52 (1.92)<br>- emotion expression 34.60 (5.27)<br><br>EI total and subscale of emotion evaluation mean scores of the nurses were found to be higher than that of the doctors.	71.43%

**Sabzevar (2016) The effect of emotional intelligence training on employed nurses**	Nurses (n=135)<br>Males (n=27)<br>Females (n=108)<br><br>Control (n=73)<br>Intervention (n=62)	Emotional Intelligence	Bar-On's Emotional Quotient Inventory (EQ-I) - 90 item	<u>Country:</u><br>Iran<br><u>Method of Collection:</u><br>Random selection of RN's in Sabzevar hospitals,<br><u>Analysis:</u><br>t-tests, non-parametrical tests	Nurses mean (SD) EI scores of 327.02 (33.44)	85.71%
**Saksvik-Lehouillier (2012) Personality factors predicting changes in shift work tolerance: A longitudinal study among nurses working rotating shifts**	Nurses (n=642)<br>Males (n=unknown)<br>Females (n=unknown)	Personality	Dispositional Resilience (hardiness) Scale-Revised (DRS15R)	<u>Country:</u><br>Norway<br><u>Method of Collection:</u><br>Questionnaires distributed via mail at three time points: Non 2008- March 2009, Jan-May 2010, Feb-Fall 2011. Two reminders if nil response.<br><u>Analysis:</u><br>descriptive statistics, hierarchal regression analyses	Nurses hardiness mean (SD) of 31.7 (4.1)	85.71%

**Saksvik-Lehouillier (2016) Hardiness, psychosocial factors and shift work tolerance among nurses - a 2-year follow-up study**	Nurses (n=2965)<br>Males (n=296)<br>Females (n=2669)	Personality	Dispositional Resilience (hardiness) Scale-Revised (DRS15R)	<u>Country:</u><br>Norway<br><u>Method of Collection:</u><br>Questionnaires distributed via mail at three time points: Non 2008- March 2009, Jan-May 2010, Feb-Fall 2011. Two reminders if nil response.<br><u>Analysis:</u><br>Descriptive statistics, correlation analyses.	Nurses Hardiness mean (SD) of 31.51 (4.59)	85.71%
**Scheepers (2016) How personality traits affect clinician-supervisors' work engagement and subsequently their teaching performance in residency training.**	Doctor Clinician Supervisors (n=636)<br>Males (n=384)<br>Females (n=252)	Personality	Big Five Inventory (BFI-10)	<u>Country:</u><br>Netherlands<br><u>Method of Collection:</u><br>Participants recruited from 61 different training programs, across 18 medical centres between May 2012 and January 2013.<br><u>Analysis:</u><br>Explored relationship of personality traits on doctors work engagement, teacher work engagement and teaching performance.	Doctor clinician supervisors personality traits mean (SD):<br>- Conscientiousness 4.26 (0.56)<br>- Agreeableness 3.89 (0.61)<br>- Extraversion 3.46 (0.80)<br>- Emotional stability 3.87 (0.67)<br>- Openness 3.45 (0.79)<br><br>Extraverted, agreeable and particularly conscientious supervisors are more likely to be engaged with their teacher work, and their high levels of teacher work engagement ultimately made them better teachers in the eyes of residents.	71.43%

**Sciacchitano (2001) Stress, burnout and hardiness in R.T.s**	Radiologists (n=95)<br>Males (n=18)<br>Females (n=77)	Personality	The Personal Views Survey II	Country:<br>USA<br>Method of Collection:<br>Survey sent to eligible participants with postage-paid return envelopes.<br>Analysis:<br>Descriptive statistics, correlations	Radiology personality hardiness mean (SD) 89.9 (11.9) (score range 0-150, where higher score = higher hardiness).	57.14%
**Shabany (2018) Emotional intelligence and depression among hospital nurses of Tehran university of medical sciences**	Nurses (n=113)<br>Males (n=66)<br>Females (n=47)	Emotional Intelligence	Bar-On's Emotional Quotient Inventory (EQ-I) - 90 item	Country:<br>Iran<br>Method of Collection:<br>RN's at Tehran University of Medical Sciences between Aug 2015 - Aug 2016, asked to fill in survey.<br>Analysis:<br>descriptive statistics, t-tests.	Nurses EI mean (SD) by gender:<br>- Male 228.1 (36.4)<br>- Female 227.8 (30.5)	85.71%
**Shah (2022) WLEIS as a Measure of Emotional Intelligence of Healthcare Professionals: A Confirmatory Factor Analysis**	Doctors (n=98)<br>Males (n=unknown)<br>Females (n=unknown)	Emotional Intelligence	Wong and Law Emotional Intelligence Scale (WLEIS)	Country:<br>India<br>Method of Collection:<br>Doctors in Ahmedabad region of Gujarat,<br>Analysis:<br>descriptive statistics, regression analysis.	Doctor global EI mean (SD) of 4.766 (0.917) higher than average score and by subscale:<br>- self-motional appraisal 4.770 (1.627)<br>- others-emotional appraisal 4.706 (1.545)<br>- use of emotion 4.711 (1.580)<br>- regulation of emotion 4.869 (1.518)	57.14%

**Sharif (2013) Teaching emotional intelligence to intensive care unit nurses and their general health: a randomized clinical trial.**	ICU Nurses (n=52)<br>Intervention (n=25)<br>Control (n=27)	Emotional Intelligence	Bar-on emotional intelligence	<u>Country:</u><br>Iran<br><u>Method of Collection:</u><br>ICU nurses affiliated to Shiraz University or Medical Sciences working at Namazi and Shadi Faghihi hospitals, randomly allocated to each group.<br><u>Analysis:</u><br>Explored effect of teaching EI on general health of ICU nurses.	ICU baseline EI mean (SD) by study group:<br>- Intervention 319.0 (33.2)<br>- Control 324.7 (27.5)<br>- not statistically significant.<br><br>Teaching emotional intelligence improved the general health of intensive care nurses.	85.71%
**Sharmila (2014) An emotional intelligence assessment among radiologist**	Radiologists (n=658)<br>Males (n=unknown)<br>Females (n=unknown)	Emotional Intelligence	Self-Emotional Intelligence Scale	<u>Country:</u><br>India<br><u>Method of Collection:</u><br>Radiologist in North India.<br><u>Analysis:</u><br>exploratory data analysis	Radiologist global EI mean (SD) of 3.5302 (0.32718), by subscale:<br>- self emotion appraisal 3.83 (0.584)<br>- others emotion appraisal 3.77 (0.486)<br>- use of emotion 3.40 (0.506)<br>- regulation of emotion 3.64 (0.475)<br><br>Radiologists exhibit medium to average levels of EI.	85.71%

**Shimizutani (2008) Relationship of nurse burnout with personality characteristics and coping behaviours**	Nurses (n=707)<br>Males (n=0)<br>Females (n=707)	Personality	Copenhagen Burnout Inventory<br>Eysenck Personality Questionnaire	Country:<br>unknown<br>Method of Collection:<br>Self-administer to nurses in March 20024 via NUM, returned by sealed envelopes.<br>Analysis:<br>multiple regression analysis, bivariate correlations, ANOVA, covariance structure analysis.	Nurses personality mean (SD) by subscale:<br>- neuroticism 7.8 (2.5)<br>- extroversion 5.7 (3.0)<br><br>Neuroticism was more closely related to personal, work-related, and client related burnout than extroversion.	85.71%
**Sims (2017) Exploring an Emotional Intelligence Model with Psychiatric Mental Health Nurses**	Psychiatric Mental Health Nurses (PMHN) (n=131)<br>Males (n=12)<br>Females (n=118)<br>Transgender (n=1)	Emotional Intelligence	Mayer-Salovey Caruso Emotional Intelligence Test (MSCEIT)<br>Self-Rated Emotional Intelligence Scale (SREIS)	Country:<br>USA<br>Method of Collection:<br>Email send to three PMHN associations, with online survey link.<br>Analysis:<br>Descriptive statistics; Pearson and spearman rank order correlations to examine relationship between two EI measures.	Total PMHN MSCEIT scores ranged from 77-130, with mean (SD) of 102.57 (12.270) and subbranch scores of:<br>- perceiving branch 99.66 (16.362)<br>- using branch 100.90 (14.493)<br>- understanding branch 102.39 (8.456)<br>- managing branch 102.33 (7.592)<br>Higher EI levels compared to normal means comparisons.<br><br>Total PMHN SREIS scores mean (SD) of 4.0227 (0.35957) and subbranch scores of:<br>- perceiving branch 4.1691 (0.48383)<br>- using branch 3.3449	85.71%

(0.40018)
- understanding branch 4.0924 (0.73247)
- managing branch 4.1676 (0.4190)

**Sladek (2010) Do doctors, nurses and managers have different thinking styles?**	Doctors, Nurses and Managers (n=152)<br><br>Medical Consultants (n=49)<br>Males (77.6%)<br>Females (22.4%)<br><br>Senior Nurses (n=50)<br>Males (12%)<br>Females (88%)<br><br>Health Managers (n=53)<br>Males (66%)<br>Females (34%)	Personality	Myers–Briggs Type Indicator	Country:<br>Australia<br>Method of Collection:<br>Personal letter to staff at two public teaching hospitals with questionnaire.<br>Analysis:<br>ANOVA with adjustment for sex. Pairwise comparisons - Turkey's least significant difference post hoc.	Nurses % personality type frequency:<br>- ISTJ 18%<br>- ESTJ 12%<br>- INTJ 12%<br>- ENTJ 12%<br>- ESFJ 10%<br>- ENFJ 8%<br>- ENTP 6%<br>- ENFP 6%<br>- INFJ 6%<br>- ISTP 6%<br>- ISFJ 4%<br>- ESFP 4%<br>- ENFJ 2%<br>- ISFP 2%<br>- ESTP 2%<br>- INFP 0%<br><br>Managers % personality type frequency:<br>- ISTJ 17%<br>- ESTJ 9.4%<br>- INTJ 11.3%<br>- ENTJ 5.7%<br>- ESFJ 0%<br>- ENFJ 3.8%<br>- ENTP 15.1%<br>- ENFP 9.4%<br>- INFJ 3.8%<br>- ISTP 1.9%	85.71%

	- ISFJ 0%<br>- ESFP 1.9%<br>- ENFJ 3.8%<br>- ISFP 1.9%<br>- ESTP 9.4%<br>- INFP 5.7%<br><br>Consultants % personality type frequency:<br>- ISTJ 16.3%<br>- ESTJ 12.2%<br>- INTJ 12..2%<br>- ENTJ 12.2%<br>- ESFJ 4.1%<br>- ENFJ 2%<br>- ENTP 6.1%<br>- ENFP 0%<br>- INFJ 10.2%<br>- ISTP 4.1%<br>- ISFJ 6.1%<br>- ESFP 2%<br>- ENFJ 2%<br>- ISFP 0%<br>- ESTP 2%<br>- INFP 4.1%<br><br>Managers reported a higher preference for 'rational' reasoning than nurses, whereas medical consultants reported a lower preference

				for 'experiential' reasoning than both managers and nurses. Cognitive style was largely homogenous.

**Spano-Szekely (2016) Emotional Intelligence and Transformational Leadership in Nurse Managers.**	Nursing Managers (n=148)<br>Males (n=unknown)<br>Females (n=unknown)	Emotional Intelligence	Trait Emotional Intelligence Questionnaire Short Form (TEIQue-SF)	<u>Country:</u><br>USA<br><u>Method of Collection:</u><br>Convenient sample of nurse managers with > 1 years' experience at 2014 Magnet conference.<br><u>Analysis:</u><br>correlation analysis, independent t-tests with Bonferroni post hoc tests.	Nursing Managers EI total mean (SD) of 170.9 (16.10)<br><br>EI was significantly positively correlated with transformational leadership.	85.71%
**Srivastava (2021) Boosting Job Satisfaction Through Emotional Intelligence: A Study on Health Care Professionals**	Health Professionals (n=260)<br>Males (n=106)<br>Females (n=154)	Emotional Intelligence	Wong and Law Emotional Intelligence Scale (WLEIS)	<u>Country:</u><br>India<br><u>Method of Collection:</u><br>Nil specific collection strategy provided.<br><u>Analysis:</u><br>Descriptive statistics	Health professionals emotional intelligence mean (SD) 60.61 (7.36) and by subscale:<br>- self-emotion appraisal 15.97 (2.55)<br>- others emotional appraisal 13.91 (2.65)<br>- use of emotion 15.41 (2.21)<br>- regulation of emotion 15.31 (2.36)	85.71%

**Stienen (2018) Different but similar: personality traits of surgeons and internists-results of a cross-sectional observational study.**	Surgeons and Interns (n=5148)<br>Males (n=2427)<br>Females (n=2721)<br><br>Specialists (n=2345)<br>Males (n=1358)<br>Females (n=987)<br><br>Residents (n=1453)<br>Males (n=618)<br>Females (n=835)<br><br>Students (n=1350)<br>Males (n=451)<br>Females (n=899)	Personality	Ten-Item Personality Inventory (TIPI)	Country:<br>Austria, Belgium, France, Canada, Germany and Switzerland.<br>Method of Collection:<br>Online survey distributed among physicians and medical students in Austria, Belgium, France, Canada, Germany and Switzerland via the management of larger public hospitals between February 2016 - May 2016.<br>Analysis:<br>MANOVA.	Normal population and board-certified physicians' personality profiles differed, with doctors scoring higher on conscientiousness, extraversion and agreeableness, but lower on neuroticism an no difference in openness to experience.<br>Surgeons score higher on extraversion and openness to experience, but lower on neuroticism. There was no difference in agreeableness and conscientiousness	71.43%

**Stovall (2021) Personality Traits and Traumatic Outcome Symptoms in Registered Nurses in the Aftermath of a Patient Safety Incident**	Nurses (n=139)	Personality	Neuroticism Scale	Country:<br>USA<br>Method of Collection:<br>RN's recruited from state board registries from Oregon and New York, who have worked over the past 5 years, involved in PSI. First 200 were incentivised with eGift cards. Data collected via REDCap through emails sent to eligible participants.<br>Analysis:<br>descriptive statistics, r2 coefficient of determination, multiple regression models.	Nurses Neuroticism mean (SD) score of 18.34 (5.80)	85.71%
**Sun (2021) Correlation between emotional intelligence and negative emotions of front-line nurses during the COVID-19 epidemic: A cross-sectional study**	Nurses (n=202)<br>Males (n=13)<br>Females (n=157)	Emotional Intelligence	Wong and Law Emotional Intelligence Scale (WLEIS-C) - Chinese version	Country:<br>China<br>Method of Collection:<br>Survey distributed by trained staff to eligible participants.<br>Analysis:<br>Descriptive statistics, single factor analysis, correlation analysis	Nurses global EI mean (SD) 69.81 (12.65) and subscale from high to low:<br>- regulation of emotion 17.57 (3.92)<br>- use of emotion 17.57 (3.38)<br>- self-emotional appraisal 17.51 (3.28)<br>- others-emotional appraisal 17.11 (3.82)<br><br>Emotional intelligence of the front-line nurses was in the	100.00%

					upper middle<br>range.	
**Surbeck (2020) Neurologists, neurosurgeons, and psychiatrists' personality traits: a comparison**	Doctors (n=3798)<br>Males (n=1976)<br>Females (n=1822)<br><br>Certified specialists (n=2345)<br>Residents(n=1453)	Personality	Ten-Item Personality Inventory (TIPI)	<u>Country:</u><br>Austria, Belgium, Canada, Germany and Switzerland<br><u>Method of Collection:</u><br>Survey completed online<br><u>Analysis:</u><br>multivariate analysis of variance, canonical linear discrimination analysis, z-scores	<u>Certifies Specialists</u> z-scores (Sd) by personality trait:<br>- agreeableness 0.25 (0.89)<br>- conscientiousness 0.80 (0.66)<br>- extraversion 0.33 (0.90)<br>- neuroticism -0.47 (0.91)<br>- openness -0.01 (0.92)<br><br><u>Resident</u> z-scores (Sd) by personality trait:<br>- agreeableness 0.45 (0.89)<br>- conscientiousness 0.87 (0.70)<br>- extraversion 0.38 (0.92)<br>- neuroticism -0.57 (0.84)<br>- openness -0.16 (0.97)	71.43%

**Swami (2013) Emotional intelligence, perceived stress and burnout among resident doctors: An assessment of the relationship**	Resident Doctors (n=56)<br>Males (n=unknown)<br>Females (n=unknown)<br><br>Medical (n=29)<br>Surgical (n=27)	Emotional Intelligence	Trait Emotional Intelligence Questionnaire-Short Form (TEIQue-SF)	Country:<br>India<br>Method of Collection:<br>residents across two departments in single institution<br>Analysis:<br>Descriptive statistics, Pearson's correlations	Medical resident EI mean (SD) of 146.72 (19.67)<br>Surgical resident EI mean (SD) of 141.52 (16.47)	71.43%
**Symer (2018) The Surgical Personality: Does Surgery Resident Motivation Predict Attrition?**	Surgical Resident Doctors (n=801)<br>Males (n=<br>Females (n=	Personality	Behaviour Inhibitory System/Behaviour Approach System (BIS/BAS)	Country:<br>USA<br>Method of Collection:<br>Surgical residents of US general surgery interns on the American Board of Surgery (ABS) in 2007, with 8 year follow up.<br>Analysis:<br>descriptive statistics, ANOVA, Cohen's d analyses.	Resident personality mean (SD) by category:<br>- BAS drive 12.2 (2.1) (score range 4-16)<br>- BAS fun seeking 11.8 (2.1) (score range 4-16)<br>- BAS reward response 17.4 (1.8) (score range 4-16)<br>- BIS 19.9 (3.2) (score range 7-28)<br><br>General surgery interns appear to be characterized by a high degree of drive, determination, and persistence.	100.00%

**Tagoe (2017) The relationship between emotional intelligence and job satisfaction among nurses in Accra**	Nurses (n=120)<br>Males (n=37)<br>Females (n=83)	Emotional Intelligence	Schutte Self-Report Emotional Intelligence Inventory	<u>Country:</u><br>Ghana<br><u>Method of Collection:</u><br>Convenient sample of registered general nurses from 3 public hospital in Accra in 2015.<br><u>Analysis:</u><br>descriptive and inferential. Pearson's correlations, independent t-test.	Nurses total EI mean (SD) score of 125.06 (14.02), by gender:<br>- Female 146.67 (21.02)<br>- Male 143.21 (18.02)<br><br>Positive correlation between emotional intelligence and job satisfaction among the nurses. No significant gender difference in emotional intelligence and job satisfaction.	100.00%
**Tajigharajeh (2021) Determining the relationship between emotional intelligence and interpersonal sensitivity with quality of work life in nurses**	Nurses (n=256)<br>Males (n=84)<br>Females (n=172)	Emotional Intelligence	Shrink's Emotional Intelligence Questionnaire	<u>Country:</u><br>Iran<br><u>Method of Collection:</u><br>Nurses working in 5 government hospitals in Tehran city,<br><u>Analysis:</u><br>descriptive statistics, t-tests, person's correlations	Nurses EI mean of 91.32 (average), and mean by subscale:<br>- self-motivating 18.74 (average)<br>- self-awareness 23.25 (average)<br>- self-control 16.24 (weak)<br>- empathy 18.63 (average)<br>- social skills 14.46 (average)	71.43%

**Takase (2018a) Effects of nurses' personality traits and their environmental characteristics on their workplace learning and nursing competence.**	Nurses (n=315)<br>Males (8.3%)<br>Females (91.7%)	Personality	Big Five Scale	<u>Country:</u><br>Japan<br><u>Method of Collection:</u><br>Nurse currently working selected three hospitals, directly involved in patient care received questionnaire via NUM between January and February 2016.<br><u>Analysis:</u><br>Descriptive statistics and multiple regression analysis to determine relationships.	Nurses personality mean (SD) by trait:<br>- extraversion 4.74 (0.82)<br>- conscientiousness 3.84 (0.87)<br>- openness to experience 4.80 (0.61)<br><br>Positive personality traits, such as extraversion, conscientiousness, and openness to experience could enhance workplace learning and nursing competence.	71.43%
**Takase (2018b) The factors related to self-other agreement/disagreement in nursing competence assessment: Comparative and correlational study.**	Nurses (n=207)<br>Males (n=50)<br>Females (n=185)	Personality	Big Five Scale	<u>Country:</u><br>Japan<br><u>Method of Collection:</u><br>Nurse currently working selected three hospitals, directly involved in patient care received questionnaire via NUM between January and February 2016.<br><u>Analysis:</u><br>Descriptive statistics and multiple regression analysis to determine relationships.	Nurse personalities mean (SD) by trait:<br>- agreeable 4.613 (0.902)<br>- neuroticism 4.509 (1.285)<br>- conscientiousness 3.868 (0.835)<br>- openness to experience 4.826 (0.617)<br>- extraversion 4.761 (0.821)<br><br>nurses' personality traits of conscientiousness and extraversion were statistically significantly related to the agreement in self-other competence assessment.	85.71%

**Talha (2022) Myers-Briggs Type Indicator Personality Types of Female Intern Doctors and Their Specialty Preference**	Doctors (n=92)<br>Males (n=<br>Females (n=	Personality	Myers–Briggs Type Indicator (MBTI)	<u>Country:</u><br>Bangladesh<br><u>Method of Collection:</u><br>Interns between Sept - Nov 2021, invited in college auditorium.<br><u>Analysis:</u><br>Descriptive statistics, Chi-square test	Interns personality by trait:<br>- ENFJ 4.34%<br>- ENFP 3.26%<br>- ENTP 5.43%<br>- ESFJ 6.52%<br>- ESFP 4.35%<br>- ESTY 4.35%<br>- ESTP 14.13%<br>- INFJ 5.43%<br>- INFP 4.34%<br>- INTJ 5.43%<br>- INTP 4.34%<br>- ISFJ 5.43%<br>- ISFP 8.69%<br>- ISTJ 9.78%<br>- ISTP 14.13%	71.43%
**Taylan (2021) Caring behaviours, moral sensitivity, and emotional intelligence in intensive care nurses: A descriptive study**	Intensive Cares Nurses (n=156)<br>Males (n=<br>Females (n=	Emotional Intelligence	Schutte Self-Report Emotional Intelligence Test (SSEIT)	<u>Country:</u><br>Turkey<br><u>Method of Collection:</u><br>Questionnaire filled in by eligible nurses between oct 2018 - April 2019, in the nurse rooms of the ICU unit.<br><u>Analysis:</u><br>descriptive statistics, Kolmogorov-Smirnov test, Mann-Whitney U test, Kruskal-Wallis test, linear regression	Nurses EI global EI mean (SD) of 150.57 (19.10) and subscale:<br>- optimism/emotion regulation 39.67 (5.48)<br>- utilising emotions 22.26 (5.01)<br>- appraisal of emotions 41.84 (1.51)	100.00%

**Tierney (1997) An investigation into modification of personality hardiness in staff nurses.**	Nurses (n=62)<br>Male (n=unknown)<br>Female (n=unknown)<br><br>Group 1: Hardiness (n=21)<br>Group 2: Time management (n=19)<br>Group 3: Control (n=22)	Personality	Third Generation Personal Views (Hardiness) questionnaire	Country:<br>?<br>Method of Collection:<br>Nurses between 6 months and 2.5 years at suburban community hospital asked to completed questionnaire and randomly allocated to 3 groups.<br>Analysis:<br>Explore the effect of hardiness training and time management training on nursing staff hardiness at three time frames post intervention and change from baseline data.	Nurses baseline personality (hardiness) total mean (SD):<br>- Group 1 (hardiness class) 73.95 (7.06)<br>- Group 2 (time management class) 78.60 (6.45)<br>- Group 3 (control) 74.74 (8.30)<br>Nurses baseline personality (hardiness) mean (SD) for commitment by study group:<br>- Group 1 (hardiness class) 38.81 (3.70)<br>- Group 2 (time management class) 41.26 (4.05)<br>- Group 3 (control) 37.67 (5.95)<br>Nurses baseline personality (hardiness) mean (SD) for control by study group:<br>- Group 1 (hardiness class) 40.14 (4.36)<br>- Group 2 (time management class) 42.47 (2.63)<br>- Group 3 (control) 39.54 (4.92)<br>Nurses baseline personality (hardiness) mean (SD) for challenge by study group:<br>- Group 1 (hardiness class) 31.76 (5.98)<br>- Group 2 (time management class) 42.47 (5.48)<br>- Group 3 (control) 34.77 (3.22)	71.43%

		No significant difference between groups at baseline. Hardiness class increased hardiness immediately post, but not sustained at 6 months. Time management class decreased hardiness immediately post and sustained at 6 months.

**Tofighi (2015) Relationship between emotional intelligence and organizational citizenship behaviour in critical and emergency nurses in southeast of Iran.**	Critical care and emergency nurses (n=150)<br>Males (n=9)<br>Females (n=141)<br><br>Emergency (n=43)<br>ICU (n=59)<br>CCU (n=35)<br>Dialysis (n=13)	Emotional Intelligence	Bradbury-Graves's emotional intelligence inventory	<u>Country:</u><br>Iran<br><u>Method of Collection:</u><br>Random sample of nurses working in teaching hospitals supervised by Kerman Medical University by letter.<br><u>Analysis:</u><br>Correlations between EI and organisational citizenship behaviour using person's correlations coefficients., On-way ANOVA and independent t-tests.	Nurses total EI mean (SD) of 121.08 (17.56). Scores ranged from 94-160, with high score indication greater level of EI.<br><br>Nurses <u>EI</u> mean (SD) by subcategory:<br>- Self-awareness 26.73 (4.55)<br>- self-management 28.51 (5.35)<br>- Social awareness 30.4 (4.31)<br>- Relationship management 35.80 (9.06)	100.00%
**Tofighi (2022) Time Management Behaviours and Emotional Intelligence in Head Nurses in Emergency and Intensive Care Units**	Nurse Managers (n=38)<br>Males (n=5)<br>Females (n=33)	Emotional Intelligence	Bradbury-Graves EI Questionnaire	<u>Country:</u><br>Iran<br><u>Method of Collection:</u><br>Head of department nurses across 9 educational hospitals, had given surveys once consent to study.<br><u>Analysis:</u><br>Nil specific details.	Nurse global EI mean (SD) of 128 (15.80) and subcategory:<br>- self-awareness 27.84 (4.42)<br>- self-management 30.57 (4.62)<br>- social awareness 20.76 (2.21)<br>- relationship management 37.39 (5.87)<br><br>Nurses have high (121-168) EI scores.	85.71%

**Topf (1989) Personality hardiness, occupational stress, and burnout in critical care nurses.**	Critical Care Nurses (n=100)<br>Males (9%)<br>Females (91%)	Personality	Hardiness<br>Maslach Burnout Inventory (MBI)	Country:<br>USA<br>Method of Collection:<br>Self-reported questionnaire distributed to nurses from 2 large university affiliated hospital<br>Analysis:	Critical Care nurses hardiness (lack of) composite mean (SD) of 0.00 (1.00)<br><br>Critical care nurses burnout mean (SD) by subscale:<br>- emotional exhaustion 20.88 (9.77)<br>- depersonalisation 6.16 (5.24)<br>- personal accomplishment 33.68 (9.01)<br>- staff burnout scale 56.32 (17.48)<br><br>Hardiness (lack of) composite scores were associated with greater occupational stress scores.	71.43%
**Tuman (2022) The effect of type D personality on anxiety, depression and fear of COVID-19 disease in healthcare workers**	Healthcare Workers (n=194)<br>Males (n=74)<br>Females (n=120)<br><br>Doctors (n=53)<br>Dentists (n=19)<br>Nurses (n=76)<br>Auxiliary Personnel (n=46)	Personality	Type D Personality Scale (DS-14)	Country:<br>Turkey<br>Method of Collection:<br>Healthcare workers, working in Mediplo University Esenler Hospital during the pandemic period.<br>Analysis:<br>Shapiro Wilk Test, Mann-Whitney U, Kruskal Wallis and Dunn multiple comparison tests	35.6% of health care workers had D type personality style.	85.71%

**Tyczkowski (2015) Emotional intelligence (EI) and nursing leadership styles among nurse managers.**	Nurse Managers (n=146)<br>Males (n=13)<br>Females (n=133)	Emotional Intelligence	Emotional Quotient Inventory (EQ-i 2.0)	Country:<br>USA<br>Method of Collection:<br>Convenient sample of 6 large midwestern health systems.<br>Analysis:<br>descriptive statistics	Nurse managers total EI mean (SD) of 107.76 (11.26) and subscale:<br>- Stress Management 107.18 (11.59)<br>- decision making 107.4 (11.18)<br>- interpersonal 108.02 (10.19)<br>- self-expression 105.98 (12.18)<br>- self-perception 104.83 (11.80)<br>- happiness 107.64 (15.09)<br><br>Positive relationships between EI and transformational leadership and the outcomes of leadership (extra effort, effectiveness, and satisfaction). No statistically significant relationships between EI and transactional or laissez-faire leadership styles.	85.71%

**Uguz (2022) Relationships between personality traits and nomophobia: Research on nurses working in public hospitals**	Nurses (n=669)<br>Males (n=115)<br>Females (n=554)	Personality	Big Five Inventory (BFI)	Country:<br>Istanbul<br>Method of Collection:<br>6 hospitals with the highest nursing numbers within the Ministry of Health, convenience sample.<br>Analysis:<br>descriptive statistics, t-tests, one-way analysis of variation, Mann-Whitney U, Kruskal-Wallis, Pearson's correlation coefficient	Nurses personality mean (SD) by trait:<br>- Extraversion 3.45 (0.63)<br>- Agreeableness 3.75 (0.55)<br>- Conscientiousness 3.77 (0.58)<br>- Neuroticism 2.78 (0.65)<br>- Openness 3.58 (0.58)<br><br>personality traits of the people do not have a strong explanatory on the levels of nomophobia	85.71%

**Uzonwanne (2016) Practising Male, in a "Woman's World": Gender, Age and Dimensions of Emotional Intelligence among Nurse Leaders in Northern Nigeria**	Nurse Leaders (n=335)<br>Males (n=101)<br>Females (n=234)	Emotional Intelligence	Wong and Law Emotional Intelligence (WLEIS)	Country:<br>Northern Nigeria<br>Method of Collection:<br>Male and female nurses from several hospitals in Kaduna and Kano state. Participants were selected from different wards, units, and departments<br>Analysis:<br>MANOVA and ANOVA statistics and descriptive statistical analysis to compare gender and age.	Nursing EI total mean (SD) 62.17 (9.35).<br>Nurses EI total mean (SD) by gender:<br>- Male 60.88 (10.05)<br>- Female 62.75 (8.97)<br><br>Nurses mean (SD) of EI subscale of self-emotional appraisal:<br>- Male 15.42 (3.34)<br>- Female 15.99 (2.81)<br>Nurses mean (SD) of EI subscale of others emotional appraisal:<br>- Male 14.20 (3.24)<br>- Female 14.98 (2.95)<br>Nurses mean (SD) of EI subscale of use of emotion:<br>- Male 16.21 (3.20)<br>- Female 16.85 (2.83)<br>Nurses mean (SD) of EI subscale of regulation of emotion:<br>- Male 14.93 (3.12)<br>- Female 15.13 (3.47)<br><br>There is a significant gender difference in the Emotional Intelligence sub-scales (Self emotion Appraisal, Others	85.71%

			Emotion Appraisal, Use of Emotion and Regulation of Emotions) of male and female nurses in Northern Nigeria. There is a significant difference in the Emotional Intelligence of the Nurses based on their age.

**Vallone (2020) Work-related stress and wellbeing among nurses: Testing a multi-dimensional model**	Nurses (n=450)<br>Males (n=206)<br>Females (n=244)	Personality Behaviour	Type D Scale-14<br>Bortner Measure of Type A behaviour	Country:<br>Italy<br>Method of Collection:<br>May 2016 - June 2017 in 5 hospitals of Italian Public Health Service.<br>Analysis:<br>descriptive statistics, logistic regression analysis, person's correlations.	Type A behavioural pattern mean (SD) 88.17 (24.61)<br>Type D personality mean (SD) 1.29 (0.45)	85.71%
**Van Dusseldorp (2011) Emotional intelligence of mental health nurses.**	Mental Health Nurses (n=98)<br>Males (n=48)<br>Females (n=50)<br><br>Inpatient care (n= 50)<br>Males (n=23)<br>Females (n=27)<br><br>Outpatient care (n=48)<br>Males (n=25)<br>Females (n=23)	Emotional Intelligence	Bar-on Emotional Quotient Inventory	Country:<br>Netherlands<br>Method of Collection:<br>Nurses from one large psychiatric institute. Survey distributed via letter and returned between February - March 2007.<br>Analysis:<br>Descriptive statistics, t-test, Mann-Whitney U tests and one way ANOVA comparison between groups.	Total EI mean (SD) (range) scores of 108.76 (11.58) (80-136).<br><br>Details of subscale mean (SD) by gender detailed on qualitative compiled spreadsheet.<br><br>Emotional intelligence of MH Nurses is statistically significantly higher than the emotional intelligence of the general population.<br>Female nurses score higher than men on the subscales Empathy, Social Responsibility, Interpersonal Relationship, Emotional Self-awareness, Self-	85.71%

					Actualisation and Assertiveness.	
**Van Mol (2018) Counterbalancing work-related stress? Work engagement among intensive care professionals**	Intensive Care professionals (n=193)<br>Males (n=61)<br>Females (n=132)<br><br>Nurses (n=146)<br>Doctors (n=32)<br>Other (n=15)<br><br>*data only analysed n=186*	Personality	Revised NEO Personality Inventory	Country:<br>Netherlands<br>Method of Collection:<br>Online survey distributed to ICU unit at largest adult ICU in the Netherlands via email. Survey responses collected over a 4 week period.<br>Analysis:<br>descriptive statistics, Pearson's and Spearman's correlations, linear regression models, ANCOVA.	Intensive care personality for nurses mean (SD) by subscale:<br>- neuroticism: 2.30 (0.6)<br>- extraversion 3.64 (0.5)<br>- openness 3.76 (0.5)<br>- agreeableness 3.95 (0.4)<br>- conscientiousness 3.99 (0.4)<br><br>Intensive care personality for doctors mean (SD) by subscale:<br>- neuroticism 2.32 (0.5)<br>- extraversion 3.61 (0.6)<br>- openness 4.11 (0.6)<br>- agreeableness 3.87 (0.5)<br>- conscientiousness 4.00 (0.4)	85.71%

**vanderWal (2016) Psychological distress, burnout and personality traits in Dutch anaesthesiologists: A survey**	Anaesthetic Doctors (n=655)<br>Males (n=388)<br>Females (n=267)<br><br>certified specialist (n=514)<br>residents (n=141)	Personality	Big Five Inventory (BFI)	Country:<br>Netherlands<br>Method of Collection:<br>Questionnaires sent between Jul 2012 - Dec 2012, with electronic reminder after 3 months of initial email.<br>Analysis:<br>descriptive statistics, Mann-Whitney U test, multivariable logistic regression	Doctor personality traits mean (SD)<br>- neuroticism 2.36 (0.60)<br>- extraversion 3.43 (0.60)<br>- openness 3.54 (0.54)<br>- agreeableness 3.75 (0.47)<br>- conscientiousness 3.89 (0.47)	71.43%
**VanServellen (1994) Personality hardiness, work-related stress, and health in hospital nurses.**	Nurses (n=237)<br>Males (n=unknown)<br>Females (n=unknown)<br><br>Nursing Units:<br>AIDS (n=4)<br>Oncology (n=4)<br>ICU (n=3)<br>General medical (n=7)	Personality	Hardiness	Country:<br>USA<br>Method of Collection:<br>Nurses employed in 7 hospital and 18 units, which were employed minimum 3 months. Questionnaire was completed at personal residence and returned to workplace in sealed envelope.<br>Analysis:<br>Comparison of nurses who work in units of known higher stress compared to known lower stress units.	Nurses total hardiness mean (SD) of 72.11 (10.16) and subscale mean (SD) of:<br>- Commitment 37.22 (6.10)<br>- Control 38.93 (4.93)<br>- Challenge 31.85 (6.45)<br><br>Significant associations were found between all three hardiness subscale, work-stress, and exhaustion scores, with the exception of the challenge subscale and stress. Hardier nurses tended to report less work-related stress, less emotional exhaustion, and fewer health problems.	71.43%

**vanZyl (2017) The effect of work stress and emotional intelligence on self-leadership among nurses in leadership positions in the Lesotho Ministry of Health and Social Welfare**	Nurse leaders (n=159)<br>Males (n=unknown)<br>Females (n=unknown)	Emotional Intelligence	Emotional Intelligence Index (EQI)	<u>Country:</u><br>Africa<br><u>Method of Collection:</u><br>Nurse leaders employed at Lesotho Ministry of Health and Social Welfare.<br><u>Analysis:</u><br>descriptive statistics, t-tests, Cronbach's alphas	Nurses EI mean (SD) of 83.3003 (39.07226) indicating low level of EI.	85.71%
**Wagner (2002) Physicians' emotional intelligence and patient satisfaction**	Doctors (n=30)<br>Males (n=18)<br>Females (n=12)<br><br>Faculty (n=14)<br>Residents (n=16)	Emotional Intelligence	Bar-On's Emotional Quotient Inventory (EQ-I) - 133 item	<u>Country:</u><br>USA<br><u>Method of Collection:</u><br>Nil specific details re collection process.<br><u>Analysis:</u><br>descriptive statistics, Spearman's correlations, t-tests	Doctors mean EI of 100.27, and by subscale:<br>- intrapersonal 100.13<br>- interpersonal 102.43<br>- adaptability 98.77<br>- stress management 98.2<br>- general mood 100.37	71.43%

**Wan (2019) A big-five personality model-based study of empathy behaviours in clinical nurses.**	Nurses (n=471)<br>Males (1.8%)<br>Females (98.2%)	Personality	Chinese Big Five Personality Inventory brief version (CBF-PI-B)	Country:<br>China<br>Method of Collection:<br>Clinical nurses at 10 hospitals in Sichuan province, questionnaires completes on site and retrieved by researcher in sealed envelope.<br>Analysis:<br>Descriptive statistics, Pearson's correlations and multiple hierarchical regressions.	Nurses personality mean (SD) scores by trait:<br>- agreeableness 38.62 (5.39)<br>- conscientiousness 37.93 (6.50)<br>- openness 33.03 (7.33)<br>- extraversion 29.93 (6.44)<br>- neuroticism 25.32 (8.71)<br><br>Empathy was positively associated with conscientiousness and agreeableness, negatively associated with neuroticism.	85.71%
**Weng (2008) Does the physician's emotional intelligence matter? Impacts of the physician's emotional intelligence on the trust, patient-physician relationship, and satisfaction.**	Physicians (n=39)<br>Males (n=35)<br>Females (n=4)<br><br>Nursing Directors (n=3)<br>Males (n=unknown)<br>Females (n=unknown)	Emotional Intelligence	Wong and Law Emotional Intelligence Scale (WLEIS)	Country:<br>Taiwan<br>Method of Collection:<br>Individually approached physicians to consent to study and completed questionnaire. Physician patients also recruited to compare EI to patient satisfaction. Nursing directors recruited to provide an external perspective.<br>Analysis:<br>Descriptive statistics, Pearson's correlations	Physicians EI mean (SD) of 5.67 (0.68).<br>Nursing directors EI mean (SD) of 5.26 (1.21).<br><br>Older clinicians had better control on emotional regulation.	71.43%

**Weng (2011a) Specialty differences in the association between health care climate and patient trust.**	Interns and Surgeons (n=211)<br>Males (n=unknown)<br>Females (n=unknown)<br><br>Interns (n=110)<br>Males (n=94)<br>Females (n=16)<br><br>Surgeons (n=101)<br>Males (n=99)<br>Females (n=2)	Emotional Intelligence	Wong and Law Emotional Intelligence Scale (WLEIS)	Country:<br>Taiwan<br>Method of Collection:<br>Attending physicians in to teaching hospitals from July 2006 - August 2007, who were teaching residents or students.<br>Analysis:<br>Hierarchical linear modelling and multi-level regression analysis.	EI mean (SD) scores by speciality:<br>- Interns 4.79 (0.63)<br>- Surgeons 4.66 (0.71)<br>No significant difference between interns and surgeon EI scores.<br><br>Positive correlation between Doctor EI and patient trust for all patients.	85.71%
**Weng (2011b) Associations between emotional intelligence and doctor burnout, job satisfaction and patient satisfaction.**	Interns (n=110)<br>Males (n=unknown)<br>Females (n=unknown)	Emotional Intelligence	Wong and Law Emotional Intelligence Scale (WLEIS)	Country:<br>Taiwan<br>Method of Collection:<br>Interns working between July 2006 - August 2007 completed questionnaires<br>Analysis:<br>Descriptive statistics and correlations.	Intern EI mean (SD) by subscale:<br>- self emotion appraisal 5.94 (0.81)<br>- other's emotion appraisal 5.10 (0.92)<br>- use of emotion 5.44 (0.80)<br>- regulation of emotion 5.22 (0.97)<br><br>Higher self-rated EI was significantly associated with less burnout and higher job satisfaction.	85.71%

**Weng (2011c) The effect of surgeon empathy and emotional intelligence on patient satisfaction**	Surgeons (n=32)<br>Males (n=unknown)<br>Females (n=unknown)	Emotional Intelligence	Wong and Law Emotional Intelligence Scale (WLEIS) - 7 point scale	<u>Country:</u><br>Taiwan<br><u>Method of Collection:</u><br>Surgeons who were performing surgeon on patients between July 2006 - Aug 2007.<br><u>Analysis:</u><br>Descriptive statistics,	Surgeons EI mean (SD) by trait:<br>- self-emotional appraisal 4.47 (1.00)<br>- others emotional appraisal 4.18 (1.00)<br>- use of emotion 4.4 (1.17)<br>- regulation of emotion 4.31 (1.18)	85.71%
**Whitworth (2005) Is there a relationship between personality type and preferred conflict-handling styles? An exploratory study of registered nurses in southern Mississippi**	Nurses (n=97)<br>Females (n=97)	Personality	Myers–Briggs Type Indicator (MBTI)	<u>Country:</u><br>USA<br><u>Method of Collection:</u><br>Female nurses employed in Mississippi administered survey physically and returned in envelope to maintain confidentiality.<br><u>Analysis:</u><br>Correlation analysis, Pearson's product, multiple regressions	Nurses personality types by percentage frequency for each indicator:<br>- Extroversion 42.3%<br>- Introversion 21.6%<br>- Sensing 64.9%<br>- Intuition 19.6%<br>- Thinking 20.6%<br>- Feeling 56.7%<br>- Judging 42.3%<br>- Perceiving 39.2%<br><br>There is no relationship between registered nurses personality factors and methods used to deal with conflict.	71.43%

**Williams (2009) Do nurses really care? Confirming the stereotype with a case control study.**	Nurses (n=174)<br>Males (n=0)<br>Females (n=174)	Personality	Ten-Item Personality Inventory (TIPI)	Country:<br>USA<br>Method of Collection:<br>Copies of questionnaire were left in staff rooms of medical and surgical wards at a university hospital. Consent implied base on anonymous return of completed questionnaire.<br>Analysis:<br>unpaired t-tests	Nurses personality mean (SD) by trait:<br>- extraversion 5.1 (1.1)<br>- agreeableness 5.6 (1.0)<br>- conscientiousness 6.2 (0.9)<br>- openness to new experience 5.5 (1.0)<br>- emotional stability 5.3 (1.2)<br><br>In direct comparison to general population adult females, staff nurses are significantly more caring, conscientious and resilient individuals.	71.43%

**Winters (2019) The Relationship Between Personality Characteristics, Tenure, and Intent to Leave Among Emergency Nurses: JEN**	Emergency Nurses (n=406)<br>Males (n=unknown)<br>Females (n=unknown)<br><br>Low tenure (< 2 years) and intent to leave < 1 year (n=31)<br>High tenure (> 10 years) (n=107)	Personality	HEXACO-PI-R	<u>Country:</u><br>USA<br><u>Method of Collection:</u><br>Emergency nurses recruited via email to researchers in response to flyers circulated to 7 hospitals; as well as Facebook and twitter posts.<br><u>Analysis:</u><br>MANOVA, ANOVA, frequency distributions and t-tests.	Nurse with low tenure (< 2 years) and intent to leave <1 year personality mean (SD) by trait:<br>- honesty/humility 3.5786 (0.51)<br>- emotionality 3.1109 (0.55)<br>- extraversion 3.5585 (0.54)<br>- agreeableness 2.9456 (0.56)<br>- openness 3.5000 (0.66)<br>- conscientiousness 3.6351 (0.48)<br><br>Nurse with high tenure (> 10 years) personality mean (SD) by trait:<br>- honesty/humility 3.9048 (0.46)<br>- emotionality 2.8242 (0.54)<br>- extraversion 3.5585 (0.54)<br>- agreeableness 2.9200 (0.56)<br>- openness 3.3411 (0.56)<br>- conscientiousness 3.6828 (0.42)<br><br>Nurses with tenure longer than 10 years have significantly higher levels of Honesty/Humility and significantly lower levels of Emotionality compared with	85.71%

	those emergency nurses who have less than 2 years of tenure and intent to leave within 1 year.

**Wolf (2022) "How obsessive are dentists?"--A personality styles & disorder inventory based prospective, controlled study**	Dentists (n=580)<br>Males (n=unknown)<br>Females (n=unknown)	Personality	Personality Styles and Disorder Inventory (PSDI)	<u>Country:</u><br>Germany<br><u>Method of Collection:</u><br>German registered dentists contacted via email.<br><u>Analysis:</u><br>descriptive statistics, t-tests, Bonferroni correction	Dentist Personality traits mean (SD)<br>**- Willful 44.72 (9.79)**<br>- **Spontaneous 46.44 (7.31)**<br>- **Reserved 44.78 (10.50)**<br>- **Ambitious 45.47 (8.27)**<br>- **Loyal 47.51 (8.77)**<br>- Critical 49.05 (8.61)<br>- **Intuitive 53.09 (10.54)**<br>- **Unselfish 52.39 (10.23)**<br>- Self-critical 50.23 (9.33)<br>- **Passive 48.85 (8.25)**<br>- Assertive 49.28 (9.29)<br>- Charming 49.79 (9.48)<br>- **Optimistic 53.60 (9.08)**<br>- Conscientious 58.84 (7.76)<br><br>Dentists differed in 10 out of 14 personality styles from the normalization sample (NORM).	85.71%

**Wright (1993a) Personality profiles of nurses: a comparison between Australian and US research findings**	Nurses (n=445)<br>Males (n=unknown)<br>Females (n=unknown)	Personality	Edwards Personal Preference Schedule (EPPS)	<u>Country:</u><br>USA<br><u>Method of Collection:</u><br>Nurses at 27 hospitals<br><u>Analysis:</u><br>Descriptive statistics, ANOVA,	Nurses personality mean (SD) by trait:<br>- Achievement 15.25 (4.18)<br>- Deference 13.41 (3.33)<br>- Order 13.63 (5.02)<br>- Exhibition 12.14 (3.78)<br>- Autonomy 11.85 (4.17)<br>- Affiliation 15.1 (3.56)<br>- Interception 16.42 (4.1)<br>- Succourance 13.2 (4.19)<br>- Dominance 12.65 (4.71)<br>- Abasement 12.89 (5.25)<br>- Nurturance 16.65 (4.31)<br>- Change 16.62 (4.63)<br>- Endurance 15.97 (4.59)<br>- Heterosexuality 12.77 (6.35)<br>- Aggression 10.78 (3.8)	85.71%

**Wright (1993b) Hardiness, stress, and burnout among intensive care nurses**	Nurses (n= 31)<br>Males (n=unknown)<br>Females (n=unknown)	Personality	The Hardiness Test	Country:<br>USA<br>Method of Collection:<br>RN's employed in 3 ICU units in south-eastern USA. Questionnaires mailed to all eligible participants, with follow up phone calls at 2 weeks if nil response. 4 week total collection period.<br>Analysis:<br>Regression studies, Hierarchical multiple regression analyses	Nursing hardiness mean (SD) of 72.00 (7.51) and by professional group:<br>- Medical/cardiac intensive care 67.42 (6.86)<br>- Neurotrauma/surgical intensive care 73.44 (5.41)<br>- Burns 74.52 (9.52)<br>(Maximum hardiness scores of 100)<br><br>Potent relationship exists between personal hardiness and burnout; it also has buffering effects in relationship between work stress and burnout.	85.71%
**Xie (2021) Mindfulness, emotional intelligence and occupational burnout in intensive care nurses: A mediating effect model**	Intensive Care Nurses (n=883)<br>Males (n=90)<br>Females (n=793)	Emotional Intelligence	Emotional Intelligence Scale (EIS)	Country:<br>China<br>Method of Collection:<br>ICU nurses at 29 ICU's survey via face-to-face session and collected by researchers.<br>Analysis:<br>Descriptive statistics, Pearson's correlations.	ICU nurses global EI mean (SD) of 122.4 (13.5) and by subscale:<br>- management of others emotions 22.9 (3.1)<br>- emotional self-management 30.1 (3.5)<br>- emotional perception 42.0 (5.2)<br>- use of emotion 27.4 (3.7)	100.00%

**Yarbeigi (2021) Investigating the relationship between demographic variables and emotional intelligence and spiritual health in the nurses working in the teaching hospitals of ilam**	Nurses (n=130)<br>Males (n=79)<br>Females (n=51)	Emotional Intelligence	Bar-On's Emotional Quotient Inventory (EQ-I) - 90 item	Country:<br>Iran<br>Method of Collection:<br>Nurses selected by census<br>Analysis:<br>Descriptive statistics, t-tests, one-way ANOVA	The total score of EI was 313.34:<br>- Moderate (181-270) = 7 (5.4%)<br>- High (271-360) = 115 (88.5%)<br>- Very High (361-450) = 8 (6.2%)<br><br>EI mean (SD) by gender:<br>- Male 313.3 (27.2)<br>- Female 313.4 (33.1)	71.43%
**Yazdanian (2016) Association between nurses' personality characteristics and their attitude toward the older adults**	Nurses (n=261)<br>Males (n=68)<br>Females (n=193)	Personality	Big Five Inventory (BFI)	Country:<br>Iran<br>Method of Collection:<br>Random stratified sampling of nurses at Isfahan hospital in 2014.<br>Analysis:<br>descriptive and inferential statistics	Nurses personality mean (SD) by trait:<br>- extroversion 25.13 (4.06)<br>- consciousness 32.29 (4.10)<br>- neuroticism 20.73 (5.27)<br>- openness 34.09 (5.07)<br>- agreeableness 33.71 (4.23)<br><br>Nurses with higher agreeableness and lower neuroticism can be an appropriate personality traits when caring for aged people.	71.43%

**Yeh (2016) The moderating effect of leadership on the relationship between personality and performance.**	Nurses (n=1488)<br>Males (n=14)<br>Females (n=1474)<br><br><u>Head Nurses</u> (n=135)<br>Males (n=0)<br>Females (n=135)<br><br><u>Registered nurses</u> (n=1353)<br>Males (n=14)<br>Females (n=1339)	Personality	Big Five Inventory (BFI)	<u>Country:</u><br>Taiwan<br><u>Method of Collection:</u><br>Nurses working in 161 nursing wards from nine hospitals between June and July 2014 working full time for >6 months. Self-reported questionnaire completed and returned in sealed envelope.<br><u>Analysis:</u><br>Tobit regression analysis to compare relationship between personality and leadership.	Nurses personality traits mean (SD) by subscale:<br>- Conscientiousness 36.53 (3.90)<br>- Agreeableness 33.71 (4.79)<br>- Extraversion 27.40 (4.33)<br>- Neuroticism 21.32 (4.79)<br>- Openness 33.29 (4.71)<br><br>Only Conscientiousness and Neuroticism were significantly related to efficiency. Those who reported high Conscientiousness and low Neuroticism were more likely to have higher efficiency.<br>Four personality traits (Conscientiousness, Extraversion, Agreeableness and Openness) are related to higher efficiency unless a strong initiating structure leadership style is also present. Neuroticism is associated with lower efficiency under the initiating structure leadership style.	85.71%

**Yildirim (2012) Nursing academicians' attitudes towards work life and their personality traits**	Academic Nurses (n=287)<br>Males (n=unknown)<br>Females (n=unknown)	Personality	Temperament and Character Inventory (TCI-240)	<u>Country:</u><br>Turkey<br><u>Method of Collection:</u><br>Nurses from 14 school of nursing universities in Turkey, between Oct 2007 - Feb 2009.<br><u>Analysis:</u><br>Descriptive statistics	Nurses temperament and character trait mean (SD):<br>- Novelty seeking 18.1 (4.4)<br>- Harm avoidance 15.3 (6.2)<br>- Reward Dependence 15.2 (3.2)<br>- Persistence 5.7 (1.8)<br>- self-directedness 35.5 (6.0)<br>- cooperativeness 32.4 (4.5)<br>- Self-transcendence 17.9 (5.4)	85.71%
**Ying (2018) Dark triad personalities and counterproductive work behaviours among physicians in China**	Doctors (n=168)<br>Males (n=unknown)<br>Females (n=unknown)	Personality	Dark Triad Personality (DTP)	<u>Country:</u><br>China<br><u>Method of Collection:</u><br>Paper surveys randomly distributed to 200 doctors at Beijing Children's Hospital, return of the form indicated consent.<br><u>Analysis:</u><br>Descriptive statistics, regression analysis, confirmatory factor analysis	Doctor narcissism mean (SD) of 2.50 (1.34)	100.00%

**Young-Ritchie (2019) The effects of emotionally intelligent leadership behaviour on emergency staff nurses' workplace empowerment and organizational commitment**	Nurses (n=283)<br>Males (n=unknown)<br>Females (n=unknown)	Emotional Intelligence	The Emotional Competency Inventory (ECI 2.0)	<u>Country:</u><br>UK<br><u>Method of Collection:</u><br>Non-experimental survey design. Random sample of R's working in emergency departments for at least 6 months from the colleges of nurses register.<br><u>Analysis:</u><br>Descriptive statistics, path analysis,	Nurse global EI mean (SD) of 3.43 (0.70), indicating they are somewhat EI.<br><br>Nurses perceptions of supervisors EI leadership behaviour has a strong effect on feelings of empowerment and organisational commitment.	100.00%
**YousifAli (2020) Emotional intelligence dimensions as predictors of coping reactions to stress in nursing practitioners**	Nurse Practitioners (n=201)<br>Males (n=115)<br>Females (n=86)	Emotional Intelligence	Brief Emotional Intelligence Scale (BEIS-10)	<u>Country:</u><br>Iraq<br><u>Method of Collection:</u><br>Nurse practitioners working in public hospitals between Nov 2017 - Jan 2018 in Duhok city in Iraqi Kurdistan. Survey administered in paper at single point of time.<br><u>Analysis:</u><br>Descriptive statistic	Nurses EI mean (SD) by subscale:<br>- appraisal of own emotions 1.99 (0.78)<br>- appraisal of others' emotions 2.19 (0.81)<br>- regulation of own emotions 2.33 (0.75)<br>- regulation of others' emotions 2.24 (0.71)<br>- utilisation of emotions 1.91 (0.73)	85.71%

**Zaid (2022) Relation between decision-making styles among head nurses and their personality traits**	Head Nurses (n=137)<br>Males (n=11)<br>Females (n=126)	Personality	Personality-trait questionnaire	<u>Country:</u><br>Egypt<br><u>Method of Collection:</u><br>All head nurses across 9 hospitals.<br><u>Analysis:</u><br>descriptive statistics, Pearson's correlation	Head nurses personality mean (SD) by factor:<br>- psychoticism 21.41 (0.86) (score range 0-25)<br>- extraversion 16.55 (2.37) (score range 0-20)<br>- neuroticism 17.40 (3.51) (score range 0-23)<br>- lie 14.38 (1.66) (score range 0-23)	100.00%
**Zeb (2021) Work-Family Conflict, Emotional Intelligence, and General Self-Efficacy Among Medical Practitioners During the COVID-19 Pandemic**	Doctors (n=140)<br>Males (n=65)<br>Females (n=75)	Emotional Intelligence	Wong and Law Emotional Intelligence Scale (WLEIS) - 7 point scale	<u>Country:</u><br>Pakistan<br><u>Method of Collection:</u><br>Online survey through purposive sampling technique.<br><u>Analysis:</u><br>Descriptive statistics, Pearson bivariate correlation, regression analysis	Doctors EI global EI mean (SD) of 5.42 (0.95), subscale scores of:<br>- self emotional appraisal 5.40 (1.19)<br>- others emotional appraisal 4.96 (1.38)<br>- regulation of emotion 5.60 (1.03)<br>- use of emotion 5.70 (1.18)	85.71%

**Zeidner (2013) Personal factors related to compassion fatigue in health professionals**	Health Practitioners (n=182)<br>Males (n=unknown<br>Females (n=unknown)<br><br>Mental Health Care Group<br>Psychologists (n=25)<br>Social Workers (n=55)<br>Psychiatrists (n=9)<br><br>Doctors (n=93)	Personality	Schutte Self-Report Emotional Intelligence Test (SSEIT)	<u>Country:</u><br>Israel<br><u>Method of Collection:</u><br>Survey completed during work time in an assessment session (60-80 minutes).<br><u>Analysis:</u><br>descriptive statistics	Mental Health professional mean (SD) by gender:<br>- Male 3.79 (0.80)<br>- Female 3.81 (0.38)<br><br>Doctors mean (SD) by gender:<br>- Male 3.63 (0.41)<br>- Female 3.74 (0.46)	85.71%
**Zhang (2013) The personality profile of excellent nurses in China: the 16PF.**	Nurses (n=159)<br>Males (n=unknown)<br>Females (n=unknown)	Personality	Cattell's Sixteen Personality Factor Questionnaire (16PF)	<u>Country:</u><br>China<br><u>Method of Collection:</u><br>convenient sample of nurses from 3 hospitals. Surveys distributed via the nursing department directors.<br><u>Analysis:</u><br>independent t-tests.	Mean (SD) for each personality subscale as per categorised spreadsheet.<br><br>Excellent nurses possess higher social boldness, openness to change, self-reliance, perfectionism, and lower dominance, vigilance, shrewdness than average nurses.	71.43%

**Zhu (2015) The impact of emotional intelligence on work engagement of registered nurses: the mediating role of organisational justice.**	Nurses (n=511)<br>Males (n=30)<br>Females (n=481)	Emotional Intelligence	Wong and Law Emotional Intelligence Scale (WLEIS)	Country:<br>China<br>Method of Collection:<br>Multistage sampling of nurses from 4 public hospitals in Jinan city, China who have worked > 6 months during April 2014 - June 2014.<br>Analysis:<br>Structural equation modelling	Nursing EI mean (SD) total of 3.8 (0.50) and score by subscale:<br>- self emotion appraisal 3.96 (0.51)<br>- regulation of emotion 3.69 (0.67)<br>- use of emotion 3.94 (0.55)<br>- other's emotion appraisal 3.68 (0.60)<br><br>Emotional intelligence were significant predictors of nurses' work engagement.	85.71%
